# Whole-Genome Analyses Identifies Multiple Reassortant Rotavirus Strains in Rwanda Post-Vaccine Introduction

**DOI:** 10.3390/v13010095

**Published:** 2021-01-12

**Authors:** Sebotsana Rasebotsa, Jeannine Uwimana, Milton T. Mogotsi, Kebareng Rakau, Nonkululeko B. Magagula, Mapaseka L. Seheri, Jason M. Mwenda, M. Jeffrey Mphahlele, Saheed Sabiu, Richard Mihigo, Leon Mutesa, Martin M. Nyaga

**Affiliations:** 1 Next Generation Sequencing Unit and Division of Virology, Faculty of Health Sciences, University of the Free State, Bloemfontein 9300, South Africa; sebotsanarasebotsa@gmail.com (S.R.); tmogotsi16@gmail.com (M.T.M.); SabiuS@dut.ac.za (S.S.); 2 Department of Laboratory, Clinical Biology, Kigali University Teaching Hospital, P.O. Box 4285, Kigali, Rwanda; jeannineuw@gmail.com; 3 Diarrheal Pathogens Research Unit, Faculty of Health Sciences, Sefako Makgatho Health Sciences University, Medunsa, Pretoria 0204, South Africa; kebarengrakau@gmail.com (K.R.); mdinonb@gmail.com (N.B.M.); mapaseka.seheri@smu.ac.za (M.L.S.); jeffrey.mphahlele@mrc.ac.za (M.J.M.); 4 World Health Organization, Regional Office for Africa, P.O. Box 06, Brazzaville, Congo; mwendaj@who.int (J.M.M.); mihigor@who.int (R.M.); 5 South African Medical Research Council, 1 Soutpansberg Road, Pretoria 0001, South Africa; 6 Centre for Human Genetics, University of Rwanda, College of Medicine and Health Sciences, P.O. Box 4285, Kigali, Rwanda; lmutesa@gmail.com

**Keywords:** rotavirus, reassortment, whole-genome based surveillance, epitopes, vaccine

## Abstract

Children in low-and middle-income countries, including Rwanda, experience a greater burden of rotavirus disease relative to developed countries. Evolutionary mechanisms leading to multiple reassortant rotavirus strains have been documented over time which influence the diversity and evolutionary dynamics of novel rotaviruses. Comprehensive rotavirus whole-genome analysis was conducted on 158 rotavirus group A (RVA) samples collected pre- and post-vaccine introduction in children less than five years in Rwanda. Of these RVA positive samples, five strains with the genotype constellations G4P[4]-I1-R2-C2-M2-A2-N2-T1-E1-H2 (n = 1), G9P[4]-I1-R2-C2-M2-A1-N1-T1-E1-H1 (n = 1), G12P[8]-I1-R2-C2-M1-A1-N2-T1-E2-H3 (n = 2) and G12P[8]-I1-R1-C1-M1-A2-N2-T2-E1-H1 (n = 1), with double and triple gene reassortant rotavirus strains were identified. Phylogenetic analysis revealed a close relationship between the Rwandan strains and cognate human RVA strains as well as the RotaTeq^®^ vaccine strains in the VP1, VP2, NSP2, NSP4 and NSP5 gene segments. Pairwise analyses revealed multiple differences in amino acid residues of the VP7 and VP4 antigenic regions of the RotaTeq^®^ vaccine strain and representative Rwandan study strains. Although the impact of such amino acid changes on the effectiveness of rotavirus vaccines has not been fully explored, this analysis underlines the potential of rotavirus whole-genome analysis by enhancing knowledge and understanding of intergenogroup reassortant strains circulating in Rwanda post vaccine introduction.

## 1. Introduction

Group A rotaviruses (RVA) are the most common cause of severe virus-induced diarrhea in infants and children less than five years of age in both developed and developing countries, accounting for over 128,500 deaths globally in 2016, of which 104,733 occurred in sub-Saharan Africa [1]. Children living in low-and middle-income countries have a higher disease burden due to poor living conditions, inadequate sanitation and limited supply of clean drinking water, among other factors [2,3]. Prior to the introduction of the vaccine RotaTeq^®^ (RV5, Merck & Co. Inc., Kenilworth, NJ, USA) in Rwanda, approximately 3500 RVA related mortality was reported in children annually, accounting for 8.8% of all mortality rate in children less than five years of age [4]. RotaTeq^®^ was introduced into the Rwandan National Immunization Program in May 2012 with a vaccine coverage of 99% within a year after introduction [5,6]. During the first three years post- RotaTeq^®^ introduction, the proportion of total diarrheal hospitalization due to rotavirus declined by 25–44% among children less than five years in the Eastern Province of Rwanda suggesting a positive vaccine impact on the population [7].

Structurally, rotaviruses are triple-layered with icosahedral symmetry and consist of an 11-segmented double-stranded RNA (dsRNA) genome encoding six structural (VP1-VP4, VP6, VP7) and five to six non-structural (NSP1-NSP5/NSP6) proteins [8,9]. Although, the binary classification is widely used to classify RVA into G (VP7) and P (VP4) viral genotypes based on the antigenicity of the two outer capsid proteins, whole-genome characterization has proven to be extensively essential and has been well facilitated with next generation sequencing (NGS) techniques to fully characterize circulating RVA strains [10,11,12]. According to the whole-genome-based classification system, there are three human RVA genogroups; the Wa-like and DS-1-like (major genogroups) and the AU-1-like (minor genogroup) [13,14,15,16]. Majority of strains that possess the Wa-like (G1-P[8]-I1-R1-C1-M1-A1-N1-T1-E1-H1) constellation are from porcine origin, while DS-1-like (G2-P[4]-I2-R2-C2-M2-A2-N2-T2-E2-H2) and AU-1-like (G3-P[9]-I3-R3-C3-M3-A3-N3-T3-E3-H3) strains are of bovine and canine/feline origin, respectively [15].

To date, 35 African countries have introduced rotavirus vaccines into their national immunization program to curb RVA infections in children less than five years [6]. Based on safety and efficacy studies, four RVA vaccines, Rotarix^®^ (RV1, GlaxoSmithKline Biologicals, Rixensart, Belgium), RotaTeq^®^ (RV5, Merck & Co. Inc., Kenilworth, NJ, USA), Rotavac^®^ (nHRV, Bharat Biotech International Limited India, Telangana, India) and Rotasil^®^ (BRV-PV, Serum Institute of India, Pune, India), have been prequalified by the World Health organization (WHO) for global use [17]. RotaTeq^®^ is a live-attenuated pentavalent vaccine composed of both bovine (WC3 and G6P[5]) and human reassortant strains (G1 [W179-9], G2 [SC2-9], G3 [W178-8], G4 [BrB-9] and P[8] [W179-4] [18]. In Africa, RotaTeq^®^ vaccine was introduced in few countries such as; Burkina Faso (2013), Côte d’Ivoire (2017), Gambia (2013), Libya (2013), Rwanda (2012) and São Tomé and Príncipe (2016) [19]. A wide range of G and P genotype combinations have been reported to circulate among African countries over the years, with G1P[8], G2P[4], G3P[8], G9P[8], G1P[6], G2P[6], G3P[6], G8P[4] and G9P[6] being the most prevalent combinations [20,21,22]. In Rwanda, G1P[8] was the predominant strain circulating in 2011 (pre-vaccine year). There was an immediate shift to G8P[4] (53% in 2013), G12P[8] (39.4% in 2014) and G4P[8] (36.6% in 2014) and were subsequently replaced by the re-emergence of G1P[8] (51.6%) in 2015 [23].

Genome reassortment between human RVA strains have been occasionally reported among circulating strains globally [24,25,26,27,28,29,30]. Such strains are suspected to influence the low effectiveness of rotavirus vaccines in regions with a significant strain diversity [31]. The RVA strains derived from intergenogroup reassortment are normally influenced by co-infection with multiple RVA strains, possibly generating strains with novel genome constellations [8,25,32,33,34]. However, such strains are reported to exhibit decreased evolutionary fitness compared to strains having a pure Wa-like or pure DS-1-like genotype constellations and thus transmit less rapidly across human populations [26,29]. In an effort to contribute to the understanding of dynamics of intergenogroup reassortment of RVA strains, the present study reports on five intergenogroup reassortant strains observed post-vaccine introduction in Rwanda between 2013 and 2015 using a whole-genome sequencing approach.

## 2. Materials and Methods

### 2.1. Ethics Statement

This study was reviewed and approved by the Health Sciences Research Ethics Committee of the UFS, Bloemfontein on the 15 October 2019 and assigned an ethics number (UFS-HSD2019/1601/2810). The diarrheal stool samples were collected as a routine diagnostic clinical sample when the parents brought their child to the hospital for clinical management, requiring no written informed consent. The archived RVA-positive samples were anonymized and utilized for strain characterization.

### 2.2. Sample Collection

Stool samples (n = 158) were collected from hospitalized children presenting with acute gastroenteritis between 2011 and 2016 in Rwanda as part of the WHO/AFRO supported rotavirus surveillance program. These samples were retrieved from “African stool repository” established to archive stool samples as part of the WHO African surveillance network, maintained at the Diarrheal Pathogens Research Unit, WHO Rotavirus Regional Reference Laboratory (Pretoria, South Africa. Five samples had the reassortant genome constellations, which meet the criteria for this study. Two samples were collected from unvaccinated children aged 14 months (female) and 36 months (male) from the South and East Province of Rwanda, respectively. The remaining three samples were collected from children (males) who received all three doses of the RotaTeq^®^ vaccine, one aged 12 months (from the North Province) and two aged 24 months (both from the East Province). The samples were whole-genome sequenced and analyzed at the UFS-NGS Unit. The Rwandan strains described in this study were deposited in GenBank database under accession numbers MT163179-MT163266.

### 2.3. Preparation of Purified dsRNA and cDNA for Rotavirus Whole-Genome Sequencing

The dsRNA was extracted using a protocol previously described by Nyaga et al.[35], and purified using the Qiagen MinElute gel extraction kit (Qiagen, Hilden, Germany). The quality of the purified dsRNA was thereafter verified by 1% agarose gel electrophoresis prior to quantification using a BioDrop-μLITE spectrophotometer (Biodrop, Cambridge, UK). For the cDNA synthesis, the Maxima H Minus Double-Stranded cDNA Synthesis Kit (ThermoFisher Scientific, Waltham, MA, USA) was used in accordance to manufacturer’s instructions with minor modifications. The modification included the denaturation of the dsRNA at 95 °C as a replacement for 65 °C for five minutes prior to synthesis of the first strand for 2 h at 50 °C in a thermocycler (Merck, Darmstadt, Germany).

### 2.4. DNA Library Preparations and Whole-Genome Sequencing

The Nextera^®^ XT DNA library preparation kit (Illumina Inc., San Diego, CA, USA) was used to construct DNA libraries for whole-genome sequencing, following the manufacturer’s instructions. The libraries were dual-barcoded using Nextera index kit (Illumina Inc.) and purified using AMPure XP magnetic beads (Beckman Coulter, Indianapolis, IN, USA) while simultaneously selecting for ~300 bp DNA fragments and removing short library fragments. Fragment sizes were confirmed using a High Sensitivity DNA Kit (Agilent Technologies, Waldbronn, Germany) and run on a Bioanalyzer 2100 (Agilent Technologies). The libraries were quantified on a Qubit 3.0 Fluorimeter (Invitrogen, Carlsbad, CA, USA). Whole-genome sequencing was performed on an Illumina MiSeq (Illumina Inc., San Diego, CA, USA) platform for 600 cycles (301 × 2 paired ends) at a final concentration of 8pM and a PhiX (20pM) spike of 20% was used as a positive control.

### 2.5. Computational Analysis

The raw reads were assembled in Geneious Prime v11.1.5 [36] (Biomatters, https://www.geneious.com/) and CLC Genomics Workbench v11 (CLC Bio, Qiagen; (https://www.qiagenbioinformatics.com/) as complementary tools. A reference based assembly was carried out for the samples and the resulting contigs were used as query sequences in the RotaC v2.0 (http://rotac.regatools.be/) and the Nucleotide Basic Local Alignment Search Tool (BLASTn; https://blast.ncbi.nlm.nih.gov/) to determine the genotype of each gene and their full-length nucleotide sequence. Phylogenetic trees were constructed using MEGA 6 software package [37] (http://www.megasoftware.net/) with Maximum Likelihood method-based model supported by bootstrap analysis with 1000 replicates. The *p*-distance algorithm was used to calculate the nucleotide distances. The VP7 and VP4 protein structure was constructed using Swiss-model protein structure server [38] (https://swissmodel.expasy.org) and PyMOL v2. The validity of the structures were confirmed using YASARA [39] and verify 3D (https://servicesn.mbi.ucla.edu/Verify3D/).

## 3. Results

### 3.1. Genome Constellations

In order to gain insight into the genetic variability among the study strains and their genetic relatedness with selected global reference RVA strains, the full-genome sequences of 158 Rwandan samples sequenced from the pre- (2011) and post- (2012–2016) vaccination period were determined. From that dataset pool, only five samples from the pool possessed reassortant constellations (Table 1). The full or near full-length nucleotide sequences reads were assembled for all the 11 gene segments of the five surveillance strains. The length size of contigs and number of reads post-assembly are given (Table 1). All the five reassortant strains were observed between 2013 and 2015 from children presenting with diarrhea and vomiting episodes. Strains RVA/Human-wt/RWA/UFS-NGS:MRC-DPRU6235/2014/G4P[4] and RVA/Human-wt/RWA/UFS-NGS:MRC-DPRU566/2013/G9P[4] from non-vaccinated children exhibited both the Wa-like and DS-1-like genotype constellation: G4P[4]-I1-R2-C2-M2-A2-N2-T1-E1-H2 and G9P[4]-I1-R2-C2-M2-A1-N1-T1-E1-H1, respectively. Two G12P[8] strains (RVA/Human-wt/RWA/UFS-NGS:MRC-DPRU8020/2015/G12P[8] and RVA/Human-wt/RWA/UFS-NGS:MRC-DPRU9995/2015/G12P[8]) possessed an identical genome constellation across the backbone (G12P[8]-I1-R2-C2-M1-A1-N2-T1-E2-H3), comprised of typical Wa-like, DS-1-like and AU-1-like genotype constellation. On the other hand, strain RVA/Human-wt/RWA/UFS-NGS:MRC-DPRU6212/2014/G12P[8] exhibited both the Wa-like and DS-1-like genotype constellation G12P[8]-I1-R1-C1-M1-A2-N2-T2-E1-H1. All the G12P[8] strains were from vaccinated children.

### 3.2. The VP4 and VP7 Antigenic Region Analyses

**Figure 1:**
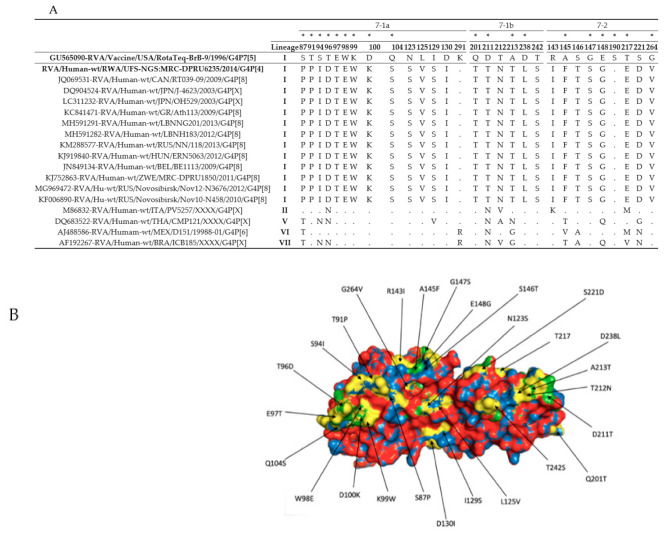
(**A**) The alignment of the G4 VP7 component of the Rotateq vaccine strain and G4 Rwandan study strain based on the three surface exposed epitope regions (7-1a, 7-1b and 7-2). The asterisk (*) represents amino acid position of residues associated with escape neutralization with monoclonal antibodies. (**B**) Surface representation of the VP7 protein. The structure has the root mean square deviation (RMSD) of 0.044 Å. The structure of the RotaTeq^®^ vaccine strain is represented with the red colour and overlapped with the structure of the Rwandan study strain indicated with the blue colour. The green colour represents the amino acid changes observed on the Rwandan study strain as compared to the RotaTeq^®^ vaccine strain (yellow).

Amino acid changes at the VP7 and VP4 antigenic epitopes of circulating RVA strains and RVA vaccine strains can affect the ability of antibodies to neutralize virus infectivity and could also undermine vaccine effectiveness [40]. In this regard, we compared the antigenic differences between RotaTeq^®^ vaccine and the Rwandan study strains by analyzing the amino acid sequence of the VP7 and VP4. The VP7 gene contains two structurally defined antigenic epitope regions: 7-1 and 7-2 made up of 29 amino acid residues [41,42]. The 7-1 epitope is further subdivided into 7-1a and 7-1b. The VP7 epitopes of the Rwandan G4 strain was compared to the G4 VP7 protein of strain RVA/Vaccine/USA/RotaTeq-BrB-9/1996/G4P75, which showed 27 amino acid differences distributed across the VP7 epitope regions (Figure 1A,B). Only two residues in position 190 (within the 7-2 region) and 291 (within the 7-1a region) were conserved between the RotaTeq^®^ vaccine strains and the Rwandan study strain (Figure 1A). Amino acid substitutions from uncharged polar molecules to charged polar molecules were observed in three positions, T96D, T217E and S221D. Furthermore, amino acid substitution from charged polar molecule to uncharged polar molecule was also observed in two positions E97T and D211T. The polarity of the other amino acids was not affected by either of the substitutions.

**Figure 2:**
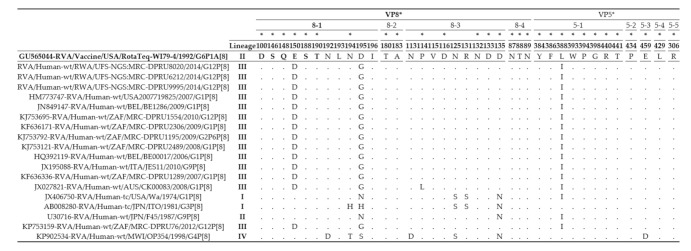
The alignment of the P[8] VP4 component of the Rotateq vaccine strain and P[8] Rwandan study strain based on the two VP4 domains, the VP8* (8-1 to 8-4) and VP5 (5-1 to 5-5). The asterisk (*) represents amino acid position of residues associated with escape neutralization with monoclonal antibodies.

In the presence of trypsin, the VP4 is cleaved into two domains, the VP8* (8-1 to 8-4) and the VP5* (5-1 to 5-5) made up of 37 amino acid residues in the antigenic epitope regions [40,43]. The VP4 epitopes of the Rwandan strains was compared to the P[8] VP4 protein of strain RVA/Vaccine/USA/RotaTeq-WI79-4/1992/G6P1A8 (Figure 2). The Rwandan study strains differed from the RotaTeq^®^ vaccine strain in only three positions, E150D and D195G at 8-1 epitope region and L388I at 5-1 epitope region, while the rest of the residues in the epitope region were conserved (Figure 2). At position 150, the amino acid changed from a glutamic acid to aspartic acid. While the change at position 195 was from an aspartic acid (charged polar molecule) to a glycine molecule (nonpolar molecule) and the change at position 388 was from leucine to isoleucine.

### 3.3. Phylogenetic Analysis of the VP7 Gene of G4, G9 and G12

**Figure 3:**
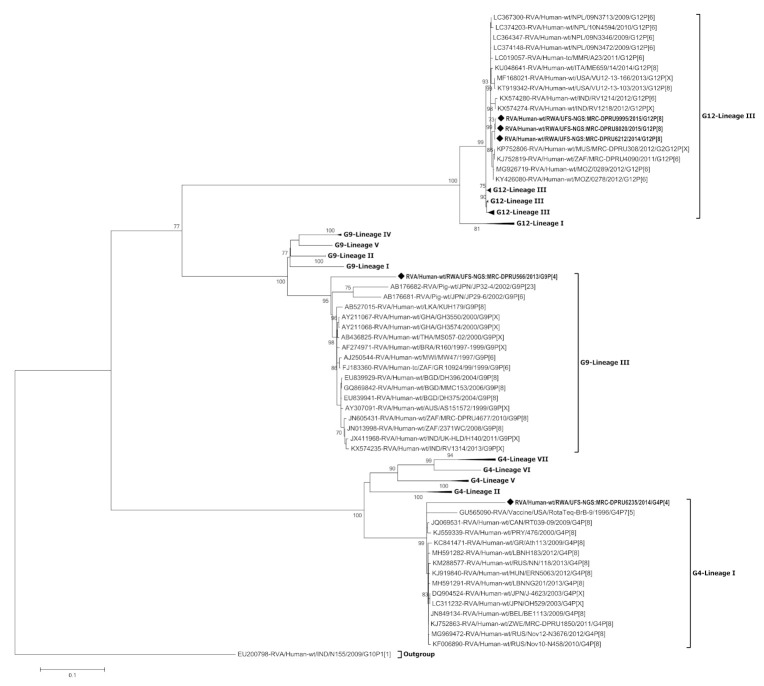
Phylogenetic relatedness of group A species based on VP7 of the study strains from Rwanda, G4, G9 and G12. The study strains are indicated with a black diamond. Bootstrap values ≥70% are indicated at each branch node. Scale bars represents substitutions per nucleotide site.

**Figure 4:**
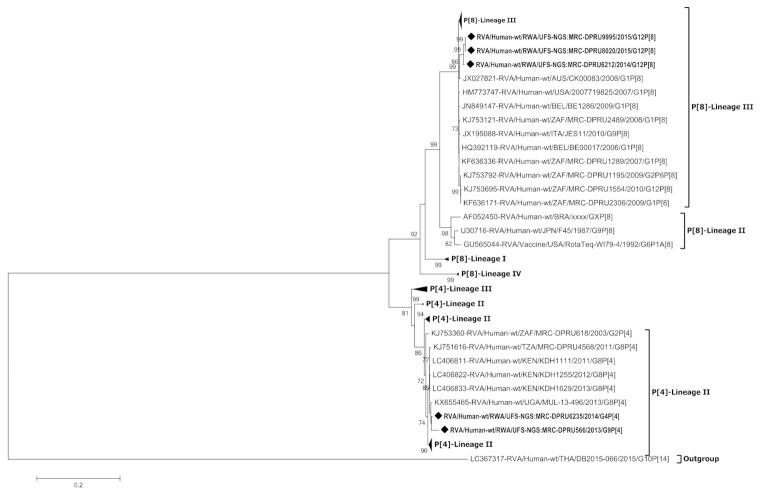
Phylogenetic relatedness of group A species based on VP4 of the study strains from Rwanda, P[4] and P[8]. The study strains are indicated with a black diamond. Bootstrap values ≥ 70% are indicated at each branch node. Scale bars represents substitutions per nucleotide site.

Phylogenetic trees were constructed for each of the 11 genome segments of the five Rwandan RVA reassortant strains in comparison with global reference strains from GenBank (Figure 3, Figure 4 and Figure S1). The three Rwandan G12P[8] strains clustered in G12 lineage III, and shared 87.6–99.3% nucleotide (nt) similarity with other lineage III G12 strains (Figure 3 and Figure S2). The G12 strains clustered together and shared 99.8–100% nt similarity amongst themselves. On the other hand, the Rwandan G9 strain clustered in G9 lineage III distantly from globally circulating G9 strains (81.7–92.1% nt similarity). The G4 strain clustered in G4 lineage I with circulating G4 global strains and exhibited 86.0% nt similarity with a RotaTeq^®^ vaccine strain.

### 3.4. Phylogenetic Analysis of the VP4 Gene of P[4] and P[8]

The VP4 gene of the two Rwandan P[4] strains and three Rwandan P[8] strains clustered in P[4]-lineage II and P[8]-lineage III, respectively (Figure 4). Strain RVA/Human-wt/RWA/UFS-NGS:MRC-DPRU6235/2014/G4P[4] and RVA/Human-wt/RWA/UFS-NGS: MRC-DPRU566/2013/G9P[4] clustered amongst strains from Kenya, South Africa, Tanzania and Uganda that circulated between 2011–2013 and shared 91.9–99.4% nt similarity. In contrast, the three Rwandan P[8] strains shared 99.3–100% nt similarity amongst themselves and clustered separately from other P[8] strains circulating globally.

### 3.5. Phylogenetic Analyses of the VP1-VP3 and VP6 Genes

The VP6 gene of all the five Rwandan study strains clustered separately within lineage I1 and formed multiple sub-clusters (Figure S1). Strain RVA/Human-wt/RWA/UFS-NGS:MRC-DPRU8020/2015/G12P[8], RVA/Human-wt/RWA/UFS-NGS:MRC-DPRU9995/2015/G12P[8] and RVA/Human-wt/RWA/UFS-NGS:MRC-DPRU6235/2014/G4P[4] formed distinct branches that clustered separately from globally circulating strains. Furthermore, the VP1 gene of the two G12P[8] (RVA/Human-wt/RWA/UFS-NGS:MRC-DPRU8020/2015/G12P[8] and RVA/Human-wt/RWA/UFS-NGS:MRC-DPRU9995/2015/G12P[8]), G4P[4] and G9P[4] strains clustered in lineage R2, while strain RVA/Human-wt/RWA/UFS-NGS:MRC-DPRU6212/2014/G12P[8] clustered in lineage R1 (Figure S1). The two G12P[8] strains in lineage R2 clustered amongst human, animal and RotaTeq^®^ vaccine strains and were homologous. The VP2 gene separated into lineage C1 and lineage C2 (Figure S1). RVA/Human-wt/RWA/UFS-NGS:MRC-DPRU8020/2015/G12P[8] and RVA/Human-wt/RWA/UFS-NGS:MRC-DPRU9995/2015/G12P[8] showed a similar clustering pattern as seen with the VP1 gene by clustering with both human and RotaTeq® vaccine strains exhibiting homologous similarity. RVA/Human-wt/RWA/UFS-NGS:MRC-DPRU6212/2014/G12P[8] clustered with strains from Nepal and Myanmar that shared 99.5–99.6% nt similarity, while the G4P[4] and G9P[4] strains formed a distinct branch from strains circulating globally. The VP3 gene of the three G12P[8] strains clustered in lineage M1, while strain G4P[4] and G9P[4] clustered in lineage M2 (Figure S1). RVA/Human-wt/RWA/UFS-NGS:MRC-DPRU566/2013/G9P[4] and RVA/Human-wt/RWA/UFS-NGS:MRC-DPRU 6235/2014/G4P[4] formed distinct branches separate from globally circulating strains, while strain RVA/Human-wt/RWA/UFS-NGS:MRC-DPRU6212/2014/G12P[8] clustered in a distinct sub-cluster to RVA/Human-wt/RWA/UFS-NGS:MRC-DPRU8020/2015/G12P[8] and RVA/Human-wt/RWA/UFS-NGS:MRC-DPRU9995/2015/G12P[8].

### 3.6. Phylogenetic Analyses of the NSP1-NSP5 Genes

The NSP1-NSP5 genes of study strain RVA/Human-wt/RWA/UFS-NGS:MRC-DPRU8020/2015/G12P[8] and RVA/Human-wt/RWA/UFS-NGS:MRC-DPRU9995/2015/G12P[8] phylogenetically clustered closely with reference strains circulating globally in previously established genotypes A1, N2, T1, E2 and H3 (Figure S1). The two G12P[8] strains also clustered among human and RotaTeq® vaccine strains in the NSP2 (homologous), NSP4 (homologous) and NSP5 (99.5–99.8% nt similarity) genes (Figures S1 and S2). Furthermore, the NSP1 gene separated into lineage A1 and lineage A2 (Figure S1). Strains G9P[4] and G4P[4] branched separately from globally circulating strains observed in lineage A1 and lineage A2, respectively. RVA/Human-wt/RWA/UFS-NGS:MRC-DPRU6212/2014/G12P[8] clustered closer to strains from Thailand that shared 99.2% nt similarity in lineage A2. The NSP2 gene of the three G12P[8] strains and the G4P[4] strain clustered in lineage N2, while the G9P[4] strain clustered in lineage N1 (Figure S1). RVA/Human-wt/RWA/UFS-NGS:MRC-DPRU6212/2014/G12P[8] and RVA/Human-wt/RWA/UFS-NGS:MRC-DPRU6235/2014/G4P[4] shared 95.9% nt similarity, while strain RVA/Human-wt/RWA/UFS-NGS:MRC-DPRU566/2013/G9P[4] shared 97.1% nt similarity with a strain from the United States. The NSP3 gene of the two G12P[8] (RVA/Human-wt/RWA/UFS-NGS:MRC-DPRU 8020/2015/G12P[8] and RVA/Human-wt/RWA/UFS-NGS:MRC-DPRU9995/2015/G12P[8]) strains, G4P[4] and G9P[4] clustered in lineage T1, while strain RVA/Human-wt/RWA/UFS-NGS:MRC-DPRU6212/2014/G12P[8] clustered in lineage T2 (Figure S1). RVA/Human-wt/RWA/UFS-NGS:MRC-DPRU6212/2014/G12P[8] was homologous to a Ugandan strain. The NSP4 gene of the G4P[4] strain clustered closely with strains from Denmark, Russia and Japan that showed 99.4–99.6% nt similarity (Figures S1 and S2). The G12P[8] and G9[4] strains that clustered in lineage E1 and shared 99% nt similarity with strains from Japan and India, respectively. The NSP5 gene separated into lineage H1, H2 and H3 (Figure S1). The G12P[8] strain in lineage H1 clustered closely with strains from Nepal and Myanmar and shared 99.1–99.3% nt similarity, while the G9P[4] strain clustered closely with a strain from the United State (99.5% nt similarity). In contrast, the G4P[4] strain shared 99.5% and 99.8% similarity with a South African and Zimbabwean strain, respectively.

## 4. Discussion

In the present study, we analyzed the whole-genome of five Rwandan rotavirus strains that have undergone intergenogroup reassortment identified as part of WHO supported on-going rotavirus sentinel surveillance. All the G12P[8] strains were from vaccinated children while the G9P[4] and G4P[4] strains were from non-vaccinated children. Despite the vaccination status of the children, they all experienced similar symptoms. Strains RVA/Human-wt/RWA/UFS-NGS:MRC-DPRU6235/2014/G4P[4], RVA/Human-wt/RWA/UFS-NGS:MRC-DPRU566/2013/G9P[4] and RVA/Human-wt/RWA/UFS-NGS: MRC-DPRU6212/2014/G12P[8] showed genotype constellations involving the Wa-like and DS-1-like genogroups while RVA/Human-wt/RWA/UFS-NGS:MRC-DPRU 8020/2015/G12P[8] and RVA/Human-wt/RWA/UFS-NGS:MRC-DPRU9995/2015/G12P[8] exhibited triple-gene reassortment of all three human genogroups, Wa-like, DS-1-like and AU-1-like. Bányai et al. [31] stated that most atypical RVA strains are result of natural intergenogroup reassortment between the Wa-like and DS-1-like strains due to the segmented nature of RVA. The emergence of these intergenogroup reassortant strains may be attributed to either, the lack of RNA polymerase proofreading ability or co-infection of multiple strains from various human RVA strains [8,15,44,45,46]. Co-infections have been reported in high frequencies in several RVA strains across Africa [21,22,35,47].

The detection of the unusual G9P[4] strain (RVA/Human-wt/RWA/UFS-NGS:MRC-DPRU566/2013/G9P[4]) in Rwanda is noteworthy as it is more prevalent in South-East Asia, Japan and Central America and detected at very low frequency (2%) in Africa [23,48,49,50,51,52,53,54]. Phylogenetic analysis revealed that the five Rwandan strains clustered mostly with strains circulating globally, with few exceptions that clustered separately. This suggests a direct importation of these variants from abroad rather than local emergence through multiple reassortment events between locally circulating strains. It is evident that the reassortment events of the five Rwandan strains mostly occurred with contemporary human rotavirus strains as they did not show sufficient evidence of animal/human reassortment. Furthermore, RVA/Human-wt/RWA/UFS-NGS:MRC-DPRU8020/2015/G12P[8] and RVA/Human-wt/RWA/UFS-NGS:MRC-DPRU9995/2015/G12P[8] exhibit a high similarity amongst each other across the genome and both human and RotaTeq^®^ vaccine strains in the VP2, NSP2, NSP4 and NSP5 gene segment. This finding suggests a reassortment between both human and RotaTeq^®^ vaccine strains might have also transpired. This observation is consistent with the findings of Rose et al. [55], who have reported that such reassortment events are expected considering the attenuation of RotaTeq^®^ vaccines and the segmented nature of the RVA genome [55].

In the construction of the RotaTeq^®^ vaccine in the 1980s, the G4 and P[8] components were included in the composition of this pentavalent vaccine [18]. When comparing the G4 and P[8] components of the Rwandan strains and the RotaTeq^®^ vaccine strains, we observed that the Rwandan G4 strain clustered distinctly to the RotaTeq^®^ vaccine strain in G4-lineage I while the Rwandan P[8] strains clustered in P[8] lineage III distantly from the RotaTeq® vaccine strain in P[8]-lineage II. This phenomenon can be attributed to the constant evolutionary changes that rotaviruses undergo. Hence the currently circulating RVA strains may cluster in VP7 and VP4 lineages different from the RVA vaccine strains [40]. Such changes may influence a varying selective pressure against these VP7 and VP4 lineages ultimately reducing vaccine effectiveness over time. The VP1, VP3, NSP1 and NSP3 genes of strain RVA/Human-wt/RWA/UFS-NGS:MRC-DPRU6235/2014/G4P[4] were phylogenetically distinct from strains circulating in other places thus suggesting that these gene segments may be unique.

Comparison of the deduced amino acid sequences of the G4P[4] and G12P[8] Rwandan strains and the RotaTeq® vaccine strain showed that none of the Rwandan strains were identical to the RotaTeq® vaccine strain. Amino acid substitutions were observed throughout the VP7 epitope regions excluding position 291 and 190, while the VP4 exhibited only three amino acid substitutions at position 150, 195 and 388. The amino acid substitution at position 96, 97, 211, 217 and 221 on the VP7 epitope region suggests a radical change in polarity. McDonald et al. [29] suggested that such substitutions do not change the genotype specificity of the rotavirus strain. However, they may influence the binding of neutralizing antibodies thus affecting the viral fitness through selection pressure. Despite the distant clustering between the Rwandan P[8] strains and the vaccine strain, only three changes were observed in the VP4 neutralizing epitope regions. Antigenic variations between rotavirus strains and the vaccine strains is often implicated in the decreased effectiveness of rotavirus vaccines in low-income countries such as Rwanda [56,57,58]. There is no evidence that the amino acid changes on the VP4 epitope regions are due to vaccination. The changes could have occurred as a natural evolutionary process as they were observed in other strains circulating globally over the years.

## 5. Conclusions

The detection of five intergenogroup reassortant Rwandan rotavirus strains from the whole-genome analysis further emphasizes the ubiquitous nature and diversity of RVA strains in circulation. Whether vaccine introduction is responsible for the observed reassortment events in vaccinated children or not, remains unknown as several natural factors can be attributed to the evolution of these RVA strains. The amino acid substitutions observed in the antigenic regions in the neutralizing epitope of the VP7 and VP4 proteins of the Rwandan strains when compared with the RotaTeq^®^ vaccine strain related to the reduced effectiveness of rotavirus vaccines in Rwanda as well as other low-income countries in the region. Continuous surveillance of rotavirus, using the whole-genome sequencing analysis, is very important to monitoring the impact of vaccine pressure, on the circulating rotavirus strains in African countries.

## 6. Study Limitations

The children in this study represents a small portion of the population of children that tested positive for rotavirus in Rwanda as part of a WHO whole-genome surveillance pilot study.

## Figures and Tables

**Table 1:** Genome constellations of five reassortant rotavirus strains detected post-vaccine introduction (2013–2015) in Rwanda along with the contigs length and number of reads mapped to each contig.

Strain Nomenclature		VP7	VP4	VP6	VP1	VP2	VP3	NSP1	NSP2	NSP3	NSP4	NSP5
RVA/Human-wt/RWA/UFS-NGS:MRC-DPRU6235/2014/G4P[4] (Non-Vaccinated))	Genome constellations	G4	P[4]	I1	R2	C2	M2	A2	N2	T1	E1	H2
	Contig length	1065	2359	1353	3303	2729	3302	3302	1044	1073	750	673
	Reads mapped to contigs	4013	16,451	6652	17,536	13,647	19,920	7673	3907	5391	3371	1892
	Genome constellations	G9	P[4]	I1	R2	C2	M2	A1	N1	T1	E1	H1
RVA/Human-wt/RWA/UFS-NGS:MRC-DPRU566/2013/G9P[4] (Non-Vaccinated)	Contig length	1061	2359	1352	3302	2726	2591	1567	1059	1074	748	664
	Reads mapped to contigs	4324	7950	7548	19,078	11,493	11,132	4451	3604	4152	3879	722
	Genome constellations	G12	P[8]	I1	R2	C2	M1	A1	N2	T1	E2	H3
RVA/Human-wt/RWA/UFS-NGS:MRC-DPRU8020/2015/G12P[8] (Vaccinated)	Contig length	1062	2359	1360	3304	2707	3302	3302	1059	1074	751	667
	Reads mapped to contigs	3888	6742	5761	20,328	12,815	11,949	12,849	3172	3484	2600	1170
	Genome constellations	G12	P[8]	I1	R2	C2	M1	A1	N2	T1	E2	H3
RVA/Human-wt/RWA/UFS-NGS:MRC-DPRU9995/2015/G12P[8] (Vaccinated)	Contig length	1062	2359	1356	3302	2687	3302	3302	1059	1074	751	668
	Reads mapped to contigs	25,828	45,020	42,925	92,061	65,575	26,672	18,505	20,815	12,752	7205	11,688
	Genome constellations	G12	P[8]	I1	R1	C1	M1	A2	N2	T2	E1	H1
RVA/Human-wt/RWA/UFS-NGS:MRC-DPRU6212/2014/G12P[8] (Vaccinated)	Contig length	1061	2359	1356	3301	2735	2591	3302	1059	1066	749	669
	Reads mapped to contigs	22,656	60,092	26,846	62,970	55,548	59,218	26,988	15,855	20,750	24,216	4705

Wa-like genogroups are represented by the green color, DS-1like is represented by the red color and AU-1-like is represented by the yellow color.

## Data Availability

Data is contained within the article and the supplementary materials.

## Supplementary Materials

The following are available online at https://www.mdpi.com/1999-4915/13/1/95/s1, Figure S1: (A–I) Phylogenetic relatedness of group A species based on (A) VP6 (B) VP1 (C) VP2 (D) VP3 (E) NSP1 (F) NSP2 (G) NSP3 (H) NSP4 (I) NSP5 of the study strains from Rwanda. The study strains are indicated with a black diamond. Bootstrap values ≥ 70% are indicated at each branch node. Scale bars represents substitutions per nucleotide site., Figure S2: Nucleotide Homology tables for all the 11 segments based on (A) VP6 (B) VP1 (C) VP2 (D) VP3 (E) NSP1 (F) NSP2 (G) NSP3 (H) NSP4 (I) NSP5 of the study strains from Rwanda.
