# Supplementary material for: Whole-Genome Analyses Identifies Multiple Reassortant Rotavirus Strains in Rwanda Post-Vaccine Introduction
Source: Viruses. 2021 Jan 12;13(1):95. doi: 10.3390/v13010095 (PMC7828107; doi:10.3390/v13010095)
Supplement: Supplementary file 1 [file viruses-13-00095-s001.pdf]

Whole-genome analyses identifies multiple reassortant rotavirus strains in Rwanda post-vaccine introduction

Supplementary data: Phylogenetic trees and homology tables based on the 11 segments of the five reassortant strains from Rwanda

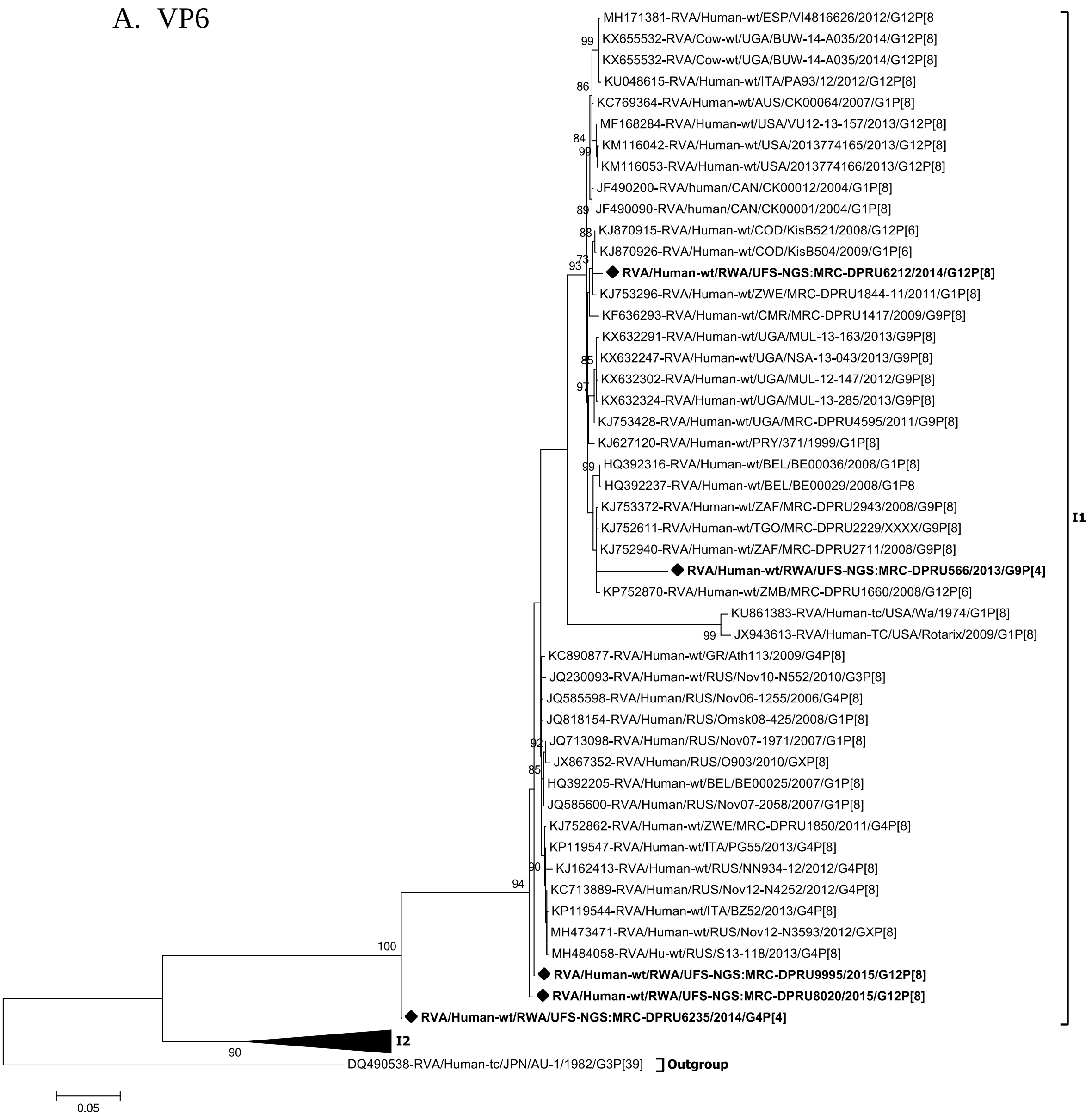

A. VP6
MH171381-RVA/Human-wt/ESP/VI4816626/2012/G12P[8
KX655532-RVA/Cow-wt/UGA/BUW-14-A035/2014/G12P[8]
KX655532-RVA/Cow-wt/UGA/BUW-14-A035/2014/G12P[8]
KU048615-RVA/Human-wt/ITA/PA93/12/2012/G12P[8]
KC769364-RVA/Human-wt/AUS/CK00064/2007/G1P[8]
MF168284-RVA/Human-wt/USA/VU12-13-157/2013/G12P[8]
KM116042-RVA/Human-wt/USA/2013774165/2013/G12P[8]
KM116053-RVA/Human-wt/USA/2013774166/2013/G12P[8]
JF490200-RVA/human/CAN/CK00012/2004/G1P[8]
JF490090-RVA/human/CAN/CK00001/2004/G1P[8]
KJ870915-RVA/Human-wt/COD/KisB521/2008/G12P[6]
KJ870926-RVA/Human-wt/COD/KisB504/2009/G1P[6]
RVA/Human-wt/RWA/UFS-NGS:MRC-DPRU6212/2014/G12P[8]
KJ753296-RVA/Human-wt/ZWE/MRC-DPRU1844-11/2011/G1P[8]
KF636293-RVA/Human-wt/CMR/MRC-DPRU1417/2009/G9P[8]
KX632291-RVA/Human-wt/UGA/MUL-13-163/2013/G9P[8]
KX632247-RVA/Human-wt/UGA/NSA-13-043/2013/G9P[8]
KX632302-RVA/Human-wt/UGA/MUL-12-147/2012/G9P[8]
KX632324-RVA/Human-wt/UGA/MUL-13-285/2013/G9P[8]
KJ753428-RVA/Human-wt/UGA/MRC-DPRU4595/2011/G9P[8]
KJ627120-RVA/Human-wt/PRY/371/1999/G1P[8]
HQ392316-RVA/Human-wt/BEL/BE00036/2008/G1P[8]
HQ392237-RVA/Human-wt/BEL/BE00029/2008/G1P8
KJ753372-RVA/Human-wt/ZAF/MRC-DPRU2943/2008/G9P[8]
KJ752611-RVA/Human-wt/TGO/MRC-DPRU2229/XXXX/G9P[8]
KJ752940-RVA/Human-wt/ZAF/MRC-DPRU2711/2008/G9P[8]
RVA/Human-wt/RWA/UFS-NGS:MRC-DPRU566/2013/G9P[4]
KP752870-RVA/Human-wt/ZMB/MRC-DPRU1660/2008/G12P[6]
KU861383-RVA/Human-tc/USA/Wa/1974/G1P[8]
JX943613-RVA/Human-TC/USA/Rotarix/2009/G1P[8]
KC890877-RVA/Human-wt/GR/Ath113/2009/G4P[8]
JQ230093-RVA/Human-wt/RUS/Nov10-N552/2010/G3P[8]
JQ585598-RVA/Human/RUS/Nov06-1255/2006/G4P[8]
JQ818154-RVA/Human/RUS/Omsk08-425/2008/G1P[8]
JQ713098-RVA/Human/RUS/Nov07-1971/2007/G1P[8]
JX867352-RVA/Human/RUS/O903/2010/GXP[8]
HQ392205-RVA/Human-wt/BEL/BE00025/2007/G1P[8]
JQ585600-RVA/Human/RUS/Nov07-2058/2007/G1P[8]
KJ752862-RVA/Human-wt/ZWE/MRC-DPRU1850/2011/G4P[8]
KP119547-RVA/Human-wt/ITA/PG55/2013/G4P[8]
KJ162413-RVA/Human-wt/RUS/NN934-12/2012/G4P[8]
KC713889-RVA/Human/RUS/Nov12-N4252/2012/G4P[8]
KP119544-RVA/Human-wt/ITA/BZ52/2013/G4P[8]
MH473471-RVA/Human-wt/RUS/Nov12-N3593/2012/GXP[8]
MH484058-RVA/Hu-wt/RUS/S13-118/2013/G4P[8]
RVA/Human-wt/RWA/UFS-NGS:MRC-DPRU9995/2015/G12P[8]
RVA/Human-wt/RWA/UFS-NGS:MRC-DPRU8020/2015/G12P[8]
RVA/Human-wt/RWA/UFS-NGS:MRC-DPRU6235/2014/G4P[4]
I1
I2
DQ490538-RVA/Human-tc/JPN/AU-1/1982/G3P[39]
Outgroup
99
86
84
99
89
88
73
93
85
97
99
99
92
85
90
94
100
90
0.05

B. VP1

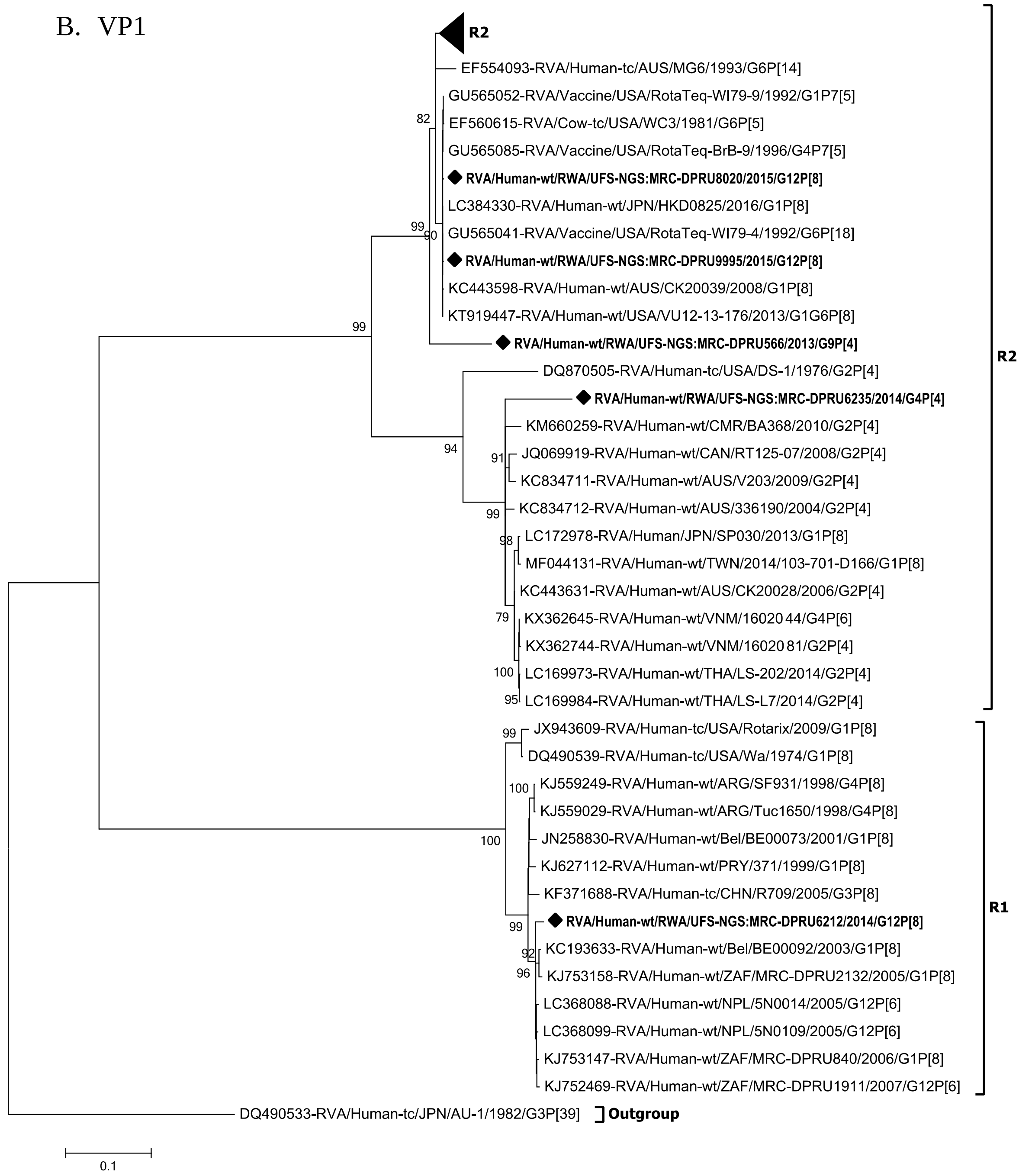
R2
EF554093-RVA/Human-tc/AUS/MG6/1993/G6P[14]
GU565052-RVA/Vaccine/USA/RotaTeq-WI79-9/1992/G1P7[5]
EF560615-RVA/Cow-tc/USA/WC3/1981/G6P[5]
GU565085-RVA/Vaccine/USA/RotaTeq-BrB-9/1996/G4P7[5]
RVA/Human-wt/RWA/UFS-NGS:MRC-DPRU8020/2015/G12P[8]
LC384330-RVA/Human-wt/JPN/HKD0825/2016/G1P[8]
GU565041-RVA/Vaccine/USA/RotaTeq-WI79-4/1992/G6P[18]
RVA/Human-wt/RWA/UFS-NGS:MRC-DPRU9995/2015/G12P[8]
KC443598-RVA/Human-wt/AUS/CK20039/2008/G1P[8]
KT919447-RVA/Human-wt/USA/VU12-13-176/2013/G1G6P[8]
RVA/Human-wt/RWA/UFS-NGS:MRC-DPRU566/2013/G9P[4]
DQ870505-RVA/Human-tc/USA/DS-1/1976/G2P[4]
RVA/Human-wt/RWA/UFS-NGS:MRC-DPRU6235/2014/G4P[4]
KM660259-RVA/Human-wt/CMR/BA368/2010/G2P[4]
JQ069919-RVA/Human-wt/CAN/RT125-07/2008/G2P[4]
KC834711-RVA/Human-wt/AUS/V203/2009/G2P[4]
KC834712-RVA/Human-wt/AUS/336190/2004/G2P[4]
LC172978-RVA/Human/JPN/SP030/2013/G1P[8]
MF044131-RVA/Human-wt/TWN/2014/103-701-D166/G1P[8]
KC443631-RVA/Human-wt/AUS/CK20028/2006/G2P[4]
KX362645-RVA/Human-wt/VNM/16020 44/G4P[6]
KX362744-RVA/Human-wt/VNM/16020 81/G2P[4]
LC169973-RVA/Human-wt/THA/LS-202/2014/G2P[4]
LC169984-RVA/Human-wt/THA/LS-L7/2014/G2P[4]
R2
JX943609-RVA/Human-tc/USA/Rotarix/2009/G1P[8]
DQ490539-RVA/Human-tc/USA/Wa/1974/G1P[8]
KJ559249-RVA/Human-wt/ARG/SF931/1998/G4P[8]
KJ559029-RVA/Human-wt/ARG/Tuc1650/1998/G4P[8]
JN258830-RVA/Human-wt/Bel/BE00073/2001/G1P[8]
KJ627112-RVA/Human-wt/PRY/371/1999/G1P[8]
KF371688-RVA/Human-tc/CHN/R709/2005/G3P[8]
RVA/Human-wt/RWA/UFS-NGS:MRC-DPRU6212/2014/G12P[8]
KC193633-RVA/Human-wt/Bel/BE00092/2003/G1P[8]
KJ753158-RVA/Human-wt/ZAF/MRC-DPRU2132/2005/G1P[8]
LC368088-RVA/Human-wt/NPL/5N0014/2005/G12P[6]
LC368099-RVA/Human-wt/NPL/5N0109/2005/G12P[6]
KJ753147-RVA/Human-wt/ZAF/MRC-DPRU840/2006/G1P[8]
KJ752469-RVA/Human-wt/ZAF/MRC-DPRU1911/2007/G12P[6]
R1
DQ490533-RVA/Human-tc/JPN/AU-1/1982/G3P[39]
Outgroup
0.1

C. VP2

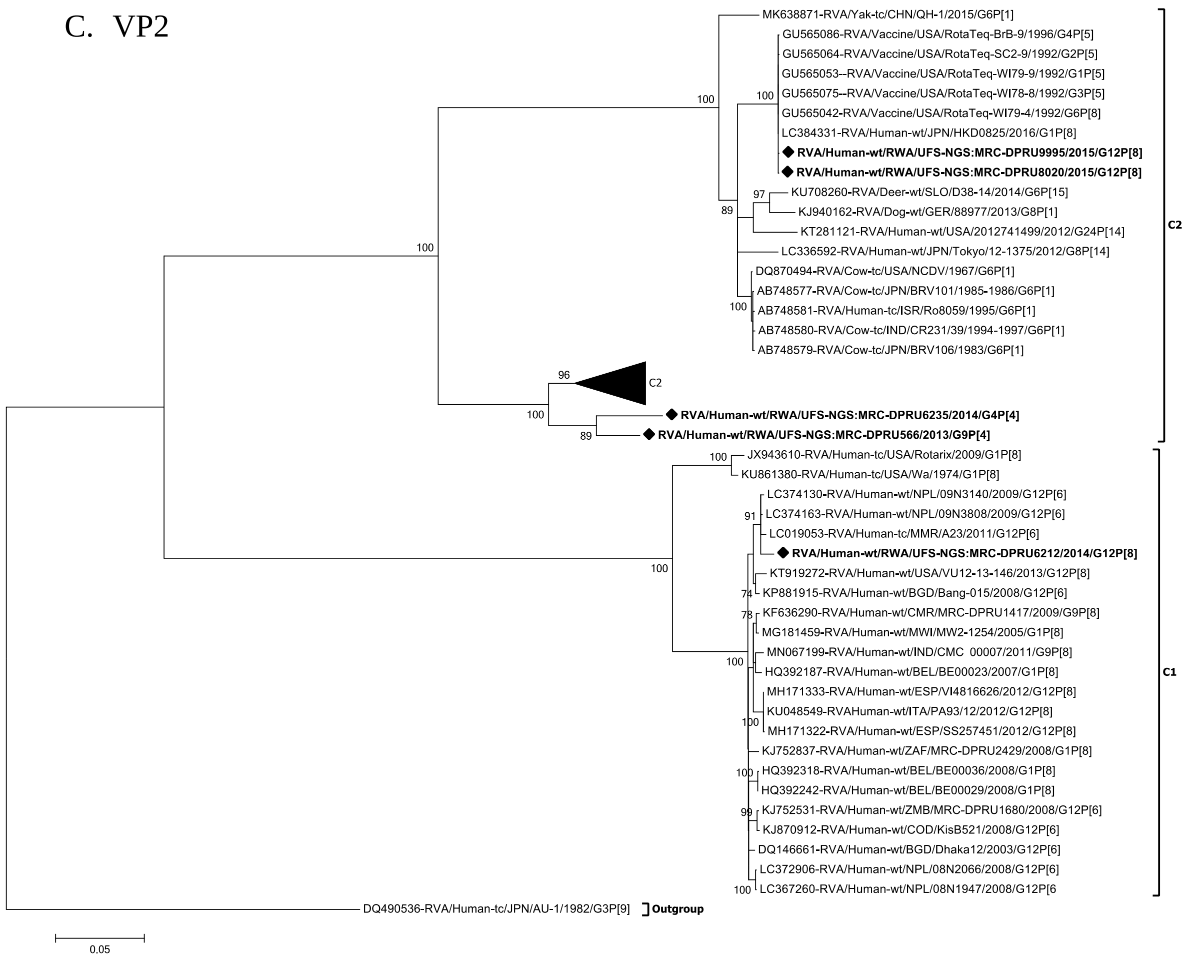

MK638871-RVA/Yak-tc/CHN/QH-1/2015/G6P[1]
GU565086-RVA/Vaccine/USA/RotaTeq-BrB-9/1996/G4P[5]
GU565064-RVA/Vaccine/USA/RotaTeq-SC2-9/1992/G2P[5]
GU565053--RVA/Vaccine/USA/RotaTeq-WI79-9/1992/G1P[5]
GU565075--RVA/Vaccine/USA/RotaTeq-WI78-8/1992/G3P[5]
GU565042-RVA/Vaccine/USA/RotaTeq-WI79-4/1992/G6P[8]
LC384331-RVA/Human-wt/JPN/HKD0825/2016/G1P[8]
RVA/Human-wt/RWA/UFS-NGS:MRC-DPRU9995/2015/G12P[8]
RVA/Human-wt/RWA/UFS-NGS:MRC-DPRU8020/2015/G12P[8]
KU708260-RVA/Deer-wt/SLO/D38-14/2014/G6P[15]
KJ940162-RVA/Dog-wt/GER/88977/2013/G8P[1]
KT281121-RVA/Human-wt/USA/2012741499/2012/G24P[14]
LC336592-RVA/Human-wt/JPN/Tokyo/12-1375/2012/G8P[14]
DQ870494-RVA/Cow-tc/USA/NCDV/1967/G6P[1]
AB748577-RVA/Cow-tc/JPN/BRV101/1985-1986/G6P[1]
AB748581-RVA/Human-tc/ISR/Ro8059/1995/G6P[1]
AB748580-RVA/Cow-tc/IND/CR231/39/1994-1997/G6P[1]
AB748579-RVA/Cow-tc/JPN/BRV106/1983/G6P[1]
C2
RVA/Human-wt/RWA/UFS-NGS:MRC-DPRU6235/2014/G4P[4]
RVA/Human-wt/RWA/UFS-NGS:MRC-DPRU566/2013/G9P[4]
JX943610-RVA/Human-tc/USA/Rotarix/2009/G1P[8]
KU861380-RVA/Human-tc/USA/Wa/1974/G1P[8]
LC374130-RVA/Human-wt/NPL/09N3140/2009/G12P[6]
LC374163-RVA/Human-wt/NPL/09N3808/2009/G12P[6]
LC019053-RVA/Human-tc/MMR/A23/2011/G12P[6]
RVA/Human-wt/RWA/UFS-NGS:MRC-DPRU6212/2014/G12P[8]
KT919272-RVA/Human-wt/USA/VU12-13-146/2013/G12P[8]
KP881915-RVA/Human-wt/BGD/Bang-015/2008/G12P[6]
KF636290-RVA/Human-wt/CMR/MRC-DPRU1417/2009/G9P[8]
MG181459-RVA/Human-wt/MWI/MW2-1254/2005/G1P[8]
MN067199-RVA/Human-wt/IND/CMC 00007/2011/G9P[8]
HQ392187-RVA/Human-wt/BEL/BE00023/2007/G1P[8]
MH171333-RVA/Human-wt/ESP/VI4816626/2012/G12P[8]
KU048549-RVAHuman-wt/ITA/PA93/12/2012/G12P[8]
MH171322-RVA/Human-wt/ESP/SS257451/2012/G12P[8]
KJ752837-RVA/Human-wt/ZAF/MRC-DPRU2429/2008/G1P[8]
HQ392318-RVA/Human-wt/BEL/BE00036/2008/G1P[8]
HQ392242-RVA/Human-wt/BEL/BE00029/2008/G1P[8]
KJ752531-RVA/Human-wt/ZMB/MRC-DPRU1680/2008/G12P[6]
KJ870912-RVA/Human-wt/COD/KisB521/2008/G12P[6]
DQ146661-RVA/Human-wt/BGD/Dhaka12/2003/G12P[6]
LC372906-RVA/Human-wt/NPL/08N2066/2008/G12P[6]
LC367260-RVA/Human-wt/NPL/08N1947/2008/G12P[6
DQ490536-RVA/Human-tc/JPN/AU-1/1982/G3P[9]
Outgroup
C2
C1
0.05

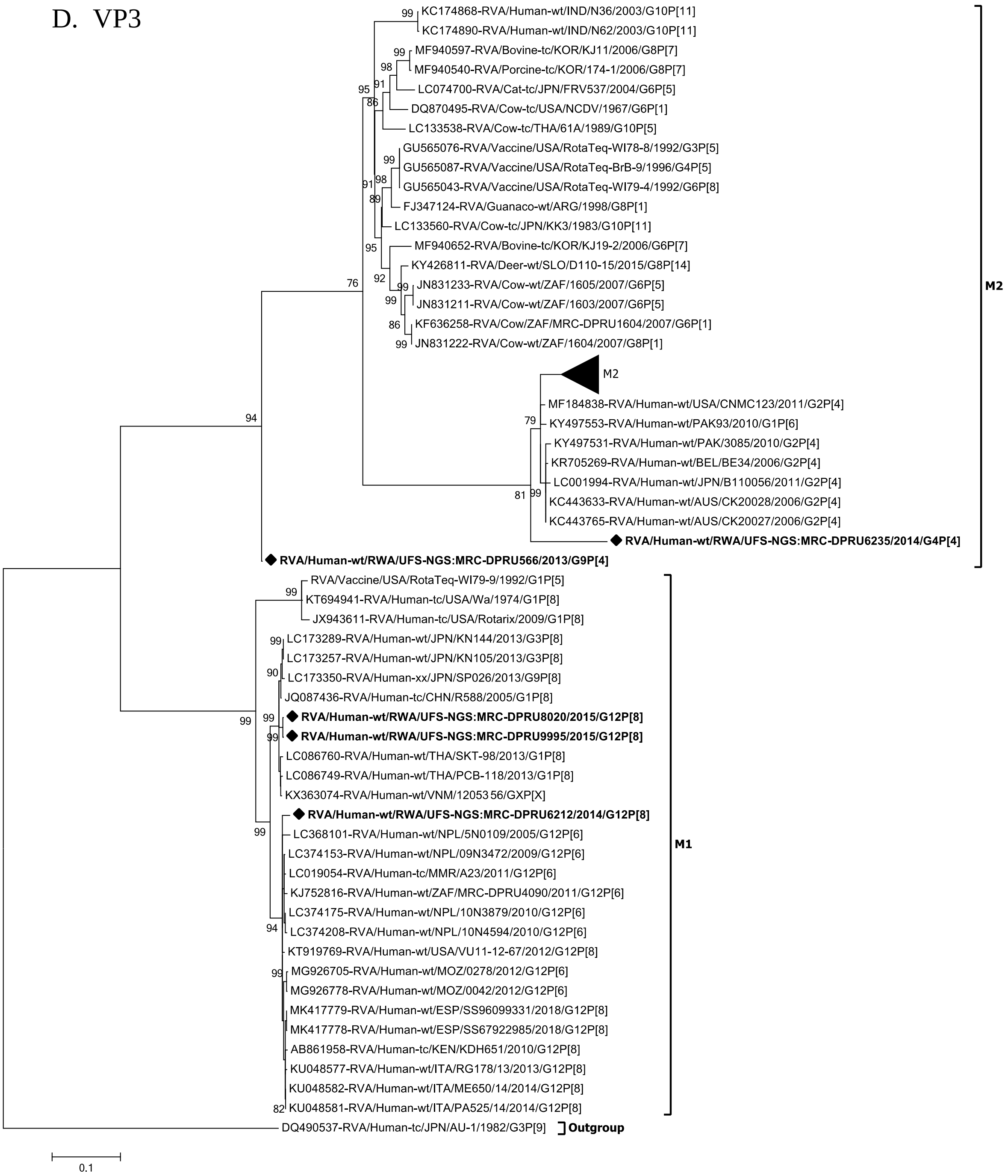
D. VP3
KC174868-RVA/Human-wt/IND/N36/2003/G10P[11]
KC174890-RVA/Human-wt/IND/N62/2003/G10P[11]
MF940597-RVA/Bovine-tc/KOR/KJ11/2006/G8P[7]
MF940540-RVA/Porcine-tc/KOR/174-1/2006/G8P[7]
LC074700-RVA/Cat-tc/JPN/FRV537/2004/G6P[5]
DQ870495-RVA/Cow-tc/USA/NCDV/1967/G6P[1]
LC133538-RVA/Cow-tc/THA/61A/1989/G10P[5]
GU565076-RVA/Vaccine/USA/RotaTeq-WI78-8/1992/G3P[5]
GU565087-RVA/Vaccine/USA/RotaTeq-BrB-9/1996/G4P[5]
GU565043-RVA/Vaccine/USA/RotaTeq-WI79-4/1992/G6P[8]
FJ347124-RVA/Guanaco-wt/ARG/1998/G8P[1]
LC133560-RVA/Cow-tc/JPN/KK3/1983/G10P[11]
MF940652-RVA/Bovine-tc/KOR/KJ19-2/2006/G6P[7]
KY426811-RVA/Deer-wt/SLO/D110-15/2015/G8P[14]
JN831233-RVA/Cow-wt/ZAF/1605/2007/G6P[5]
JN831211-RVA/Cow-wt/ZAF/1603/2007/G6P[5]
KF636258-RVA/Cow/ZAF/MRC-DPRU1604/2007/G6P[1]
JN831222-RVA/Cow-wt/ZAF/1604/2007/G8P[1]
M2
MF184838-RVA/Human-wt/USA/CNMC123/2011/G2P[4]
KY497553-RVA/Human-wt/PAK93/2010/G1P[6]
KY497531-RVA/Human-wt/PAK/3085/2010/G2P[4]
KR705269-RVA/Human-wt/BEL/BE34/2006/G2P[4]
LC001994-RVA/Human-wt/JPN/B110056/2011/G2P[4]
KC443633-RVA/Human-wt/AUS/CK20028/2006/G2P[4]
KC443765-RVA/Human-wt/AUS/CK20027/2006/G2P[4]
RVA/Human-wt/RWA/UFS-NGS:MRC-DPRU6235/2014/G4P[4]
RVA/Human-wt/RWA/UFS-NGS:MRC-DPRU566/2013/G9P[4]
M2
RVA/Vaccine/USA/RotaTeq-WI79-9/1992/G1P[5]
KT694941-RVA/Human-tc/USA/Wa/1974/G1P[8]
JX943611-RVA/Human-tc/USA/Rotarix/2009/G1P[8]
LC173289-RVA/Human-wt/JPN/KN144/2013/G3P[8]
LC173257-RVA/Human-wt/JPN/KN105/2013/G3P[8]
LC173350-RVA/Human-xx/JPN/SP026/2013/G9P[8]
JQ087436-RVA/Human-tc/CHN/R588/2005/G1P[8]
RVA/Human-wt/RWA/UFS-NGS:MRC-DPRU8020/2015/G12P[8]
RVA/Human-wt/RWA/UFS-NGS:MRC-DPRU9995/2015/G12P[8]
LC086760-RVA/Human-wt/THA/SKT-98/2013/G1P[8]
LC086749-RVA/Human-wt/THA/PCB-118/2013/G1P[8]
KX363074-RVA/Human-wt/VNM/12053 56/GXP[X]
RVA/Human-wt/RWA/UFS-NGS:MRC-DPRU6212/2014/G12P[8]
LC368101-RVA/Human-wt/NPL/5N0109/2005/G12P[6]
LC374153-RVA/Human-wt/NPL/09N3472/2009/G12P[6]
LC019054-RVA/Human-tc/MMR/A23/2011/G12P[6]
KJ752816-RVA/Human-wt/ZAF/MRC-DPRU4090/2011/G12P[6]
LC374175-RVA/Human-wt/NPL/10N3879/2010/G12P[6]
LC374208-RVA/Human-wt/NPL/10N4594/2010/G12P[6]
KT919769-RVA/Human-wt/USA/VU11-12-67/2012/G12P[8]
MG926705-RVA/Human-wt/MOZ/0278/2012/G12P[6]
MG926778-RVA/Human-wt/MOZ/0042/2012/G12P[6]
MK417779-RVA/Human-wt/ESP/SS96099331/2018/G12P[8]
MK417778-RVA/Human-wt/ESP/SS67922985/2018/G12P[8]
AB861958-RVA/Human-tc/KEN/KDH651/2010/G12P[8]
KU048577-RVA/Human-wt/ITA/RG178/13/2013/G12P[8]
KU048582-RVA/Human-wt/ITA/ME650/14/2014/G12P[8]
KU048581-RVA/Human-wt/ITA/PA525/14/2014/G12P[8]
M1
DQ490537-RVA/Human-tc/JPN/AU-1/1982/G3P[9]
Outgroup
0.1

E. NSP1

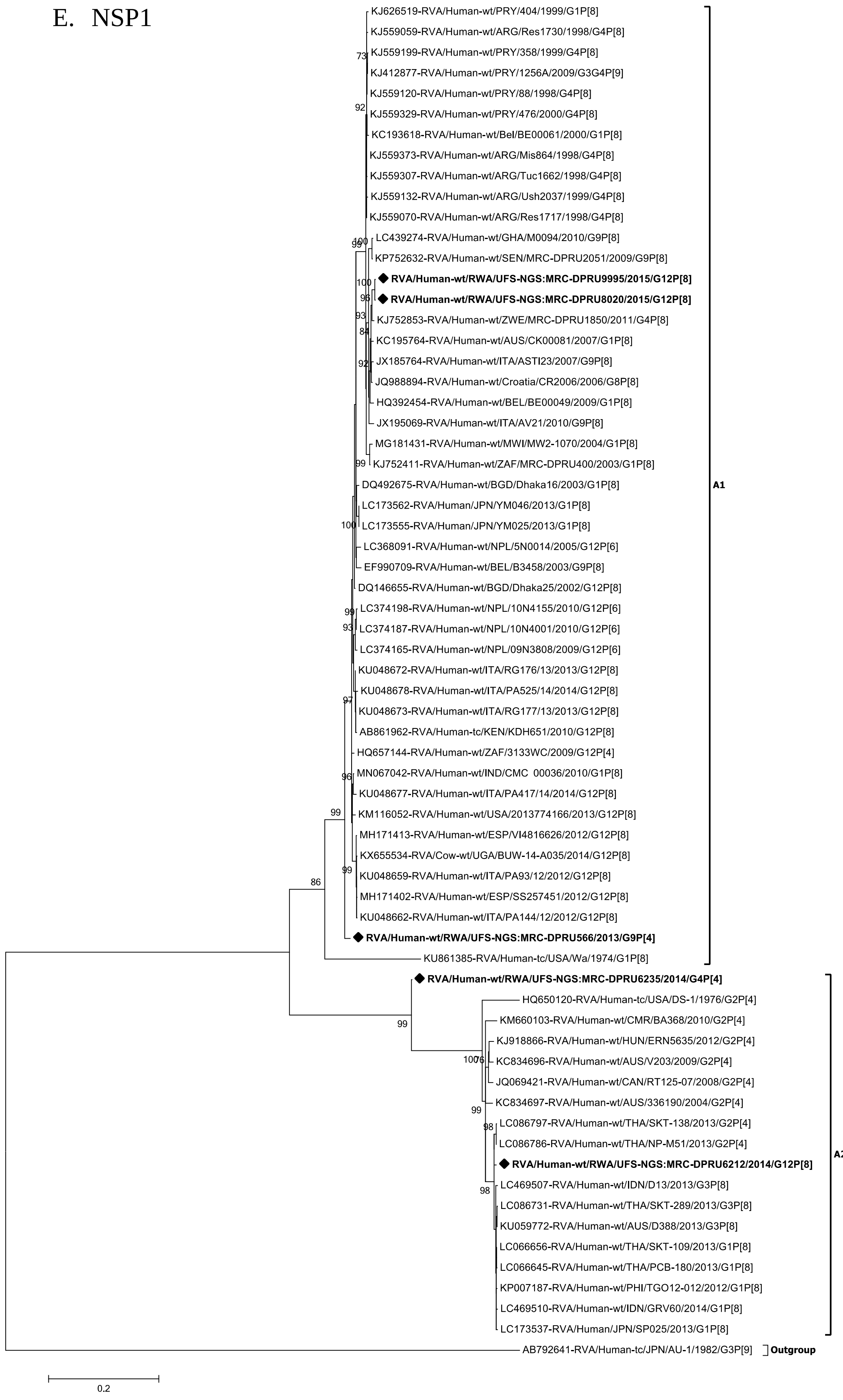
KJ626519-RVA/Human-wt/PRY/404/1999/G1P[8]
KJ559059-RVA/Human-wt/ARG/Res1730/1998/G4P[8]
KJ559199-RVA/Human-wt/PRY/358/1999/G4P[8]
KJ412877-RVA/Human-wt/PRY/1256A/2009/G3G4P[9]
KJ559120-RVA/Human-wt/PRY/88/1998/G4P[8]
KJ559329-RVA/Human-wt/PRY/476/2000/G4P[8]
KC193618-RVA/Human-wt/Bel/BE00061/2000/G1P[8]
KJ559373-RVA/Human-wt/ARG/Mis864/1998/G4P[8]
KJ559307-RVA/Human-wt/ARG/Tuc1662/1998/G4P[8]
KJ559132-RVA/Human-wt/ARG/Ush2037/1999/G4P[8]
KJ559070-RVA/Human-wt/ARG/Res1717/1998/G4P[8]
LC439274-RVA/Human-wt/GHA/M0094/2010/G9P[8]
KP752632-RVA/Human-wt/SEN/MRC-DPRU2051/2009/G9P[8]
RVA/Human-wt/RWA/UFS-NGS:MRC-DPRU9995/2015/G12P[8]
RVA/Human-wt/RWA/UFS-NGS:MRC-DPRU8020/2015/G12P[8]
KJ752853-RVA/Human-wt/ZWE/MRC-DPRU1850/2011/G4P[8]
KC195764-RVA/Human-wt/AUS/CK00081/2007/G1P[8]
JX185764-RVA/Human-wt/ITA/ASTI23/2007/G9P[8]
JQ988894-RVA/Human-wt/Croatia/CR2006/2006/G8P[8]
HQ392454-RVA/Human-wt/BEL/BE00049/2009/G1P[8]
JX195069-RVA/Human-wt/ITA/AV21/2010/G9P[8]
MG181431-RVA/Human-wt/MWI/MW2-1070/2004/G1P[8]
KJ752411-RVA/Human-wt/ZAF/MRC-DPRU400/2003/G1P[8]
DQ492675-RVA/Human-wt/BGD/Dhaka16/2003/G1P[8]
LC173562-RVA/Human/JPN/YM046/2013/G1P[8]
LC173555-RVA/Human/JPN/YM025/2013/G1P[8]
LC368091-RVA/Human-wt/NPL/5N0014/2005/G12P[6]
EF990709-RVA/Human-wt/BEL/B3458/2003/G9P[8]
DQ146655-RVA/Human-wt/BGD/Dhaka25/2002/G12P[8]
LC374198-RVA/Human-wt/NPL/10N4155/2010/G12P[6]
LC374187-RVA/Human-wt/NPL/10N4001/2010/G12P[6]
LC374165-RVA/Human-wt/NPL/09N3808/2009/G12P[6]
KU048672-RVA/Human-wt/ITA/RG176/13/2013/G12P[8]
KU048678-RVA/Human-wt/ITA/PA525/14/2014/G12P[8]
KU048673-RVA/Human-wt/ITA/RG177/13/2013/G12P[8]
AB861962-RVA/Human-tc/KEN/KDH651/2010/G12P[8]
HQ657144-RVA/Human-wt/ZAF/3133WC/2009/G12P[4]
MN067042-RVA/Human-wt/IND/CMC 00036/2010/G1P[8]
KU048677-RVA/Human-wt/ITA/PA417/14/2014/G12P[8]
KM116052-RVA/Human-wt/USA/2013774166/2013/G12P[8]
MH171413-RVA/Human-wt/ESP/VI4816626/2012/G12P[8]
KX655534-RVA/Cow-wt/UGA/BUW-14-A035/2014/G12P[8]
KU048659-RVA/Human-wt/ITA/PA93/12/2012/G12P[8]
MH171402-RVA/Human-wt/ESP/SS257451/2012/G12P[8]
KU048662-RVA/Human-wt/ITA/PA144/12/2012/G12P[8]
RVA/Human-wt/RWA/UFS-NGS:MRC-DPRU566/2013/G9P[4]
KU861385-RVA/Human-tc/USA/Wa/1974/G1P[8]
A1
RVA/Human-wt/RWA/UFS-NGS:MRC-DPRU6235/2014/G4P[4]
HQ650120-RVA/Human-tc/USA/DS-1/1976/G2P[4]
KM660103-RVA/Human-wt/CMR/BA368/2010/G2P[4]
KJ918866-RVA/Human-wt/HUN/ERN5635/2012/G2P[4]
KC834696-RVA/Human-wt/AUS/V203/2009/G2P[4]
JQ069421-RVA/Human-wt/CAN/RT125-07/2008/G2P[4]
KC834697-RVA/Human-wt/AUS/336190/2004/G2P[4]
LC086797-RVA/Human-wt/THA/SKT-138/2013/G2P[4]
LC086786-RVA/Human-wt/THA/NP-M51/2013/G2P[4]
RVA/Human-wt/RWA/UFS-NGS:MRC-DPRU6212/2014/G12P[8]
LC469507-RVA/Human-wt/IDN/D13/2013/G3P[8]
LC086731-RVA/Human-wt/THA/SKT-289/2013/G3P[8]
KU059772-RVA/Human-wt/AUS/D388/2013/G3P[8]
LC066656-RVA/Human-wt/THA/SKT-109/2013/G1P[8]
LC066645-RVA/Human-wt/THA/PCB-180/2013/G1P[8]
KP007187-RVA/Human-wt/PHI/TGO12-012/2012/G1P[8]
LC469510-RVA/Human-wt/IDN/GRV60/2014/G1P[8]
LC173537-RVA/Human/JPN/SP025/2013/G1P[8]
A2
AB792641-RVA/Human-tc/JPN/AU-1/1982/G3P[9]
Outgroup
0.2

F. NSP2

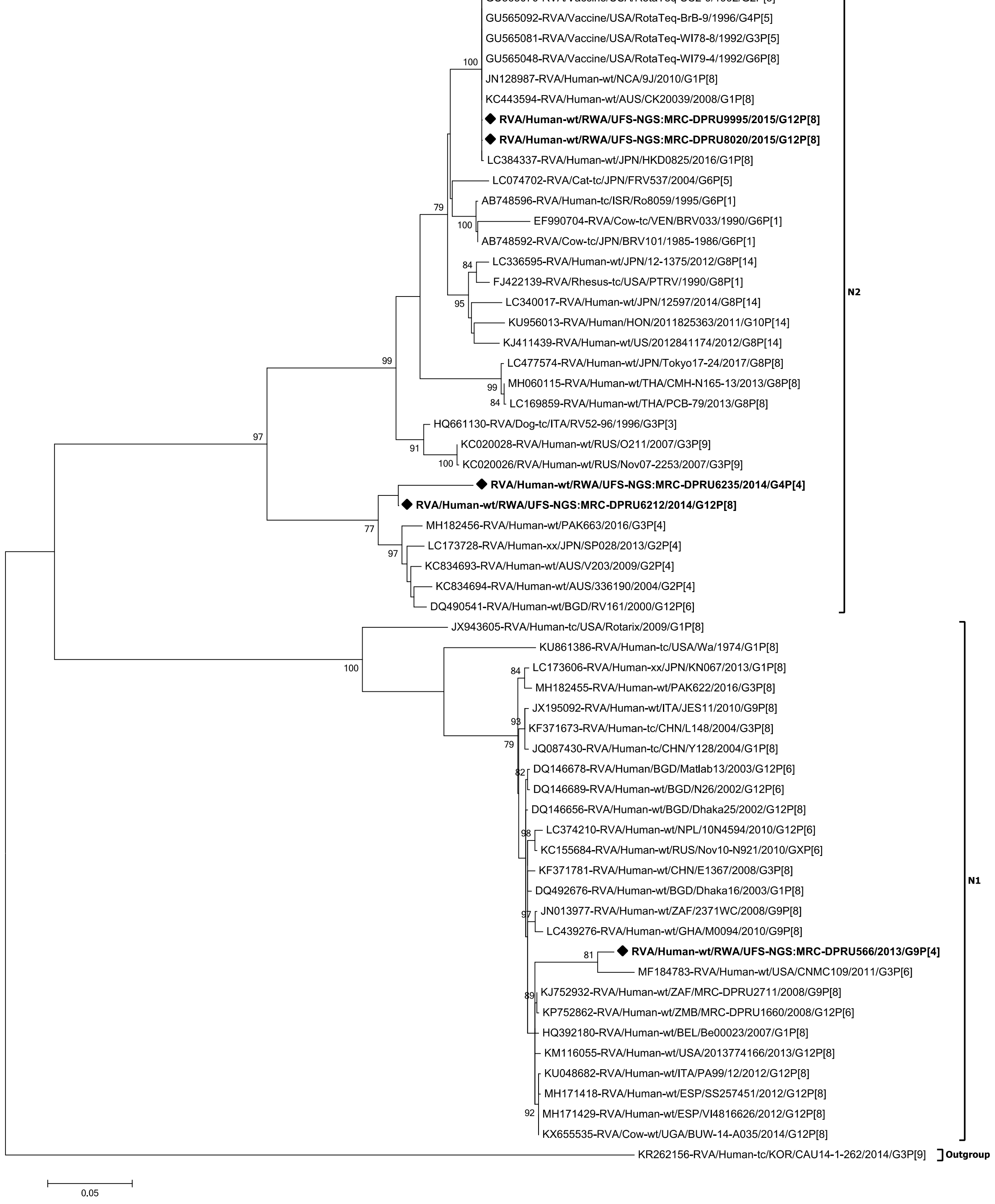
GU565059-RVA/Vaccine/USA/RotaTeq-WI79-9/1992/G1P[5]
GU565070-RVA/Vaccine/USA/RotaTeq-SC2-9/1992/G2P[5]
GU565092-RVA/Vaccine/USA/RotaTeq-BrB-9/1996/G4P[5]
GU565081-RVA/Vaccine/USA/RotaTeq-WI78-8/1992/G3P[5]
GU565048-RVA/Vaccine/USA/RotaTeq-WI79-4/1992/G6P[8]
JN128987-RVA/Human-wt/NCA/9J/2010/G1P[8]
KC443594-RVA/Human-wt/AUS/CK20039/2008/G1P[8]
RVA/Human-wt/RWA/UFS-NGS:MRC-DPRU9995/2015/G12P[8]
RVA/Human-wt/RWA/UFS-NGS:MRC-DPRU8020/2015/G12P[8]
LC384337-RVA/Human-wt/JPN/HKD0825/2016/G1P[8]
LC074702-RVA/Cat-tc/JPN/FRV537/2004/G6P[5]
AB748596-RVA/Human-tc/ISR/Ro8059/1995/G6P[1]
EF990704-RVA/Cow-tc/VEN/BRV033/1990/G6P[1]
AB748592-RVA/Cow-tc/JPN/BRV101/1985-1986/G6P[1]
LC336595-RVA/Human-wt/JPN/12-1375/2012/G8P[14]
FJ422139-RVA/Rhesus-tc/USA/PTRV/1990/G8P[1]
LC340017-RVA/Human-wt/JPN/12597/2014/G8P[14]
KU956013-RVA/Human/HON/2011825363/2011/G10P[14]
KJ411439-RVA/Human-wt/US/2012841174/2012/G8P[14]
LC477574-RVA/Human-wt/JPN/Tokyo17-24/2017/G8P[8]
MH060115-RVA/Human-wt/THA/CMH-N165-13/2013/G8P[8]
LC169859-RVA/Human-wt/THA/PCB-79/2013/G8P[8]
HQ661130-RVA/Dog-tc/ITA/RV52-96/1996/G3P[3]
KC020028-RVA/Human-wt/RUS/O211/2007/G3P[9]
KC020026/RVA/Human-wt/RUS/Nov07-2253/2007/G3P[9]
RVA/Human-wt/RWA/UFS-NGS:MRC-DPRU6235/2014/G4P[4]
RVA/Human-wt/RWA/UFS-NGS:MRC-DPRU6212/2014/G12P[8]
MH182456-RVA/Human-wt/PAK663/2016/G3P[4]
LC173728-RVA/Human-xx/JPN/SP028/2013/G2P[4]
KC834693-RVA/Human-wt/AUS/V203/2009/G2P[4]
KC834694-RVA/Human-wt/AUS/336190/2004/G2P[4]
DQ490541-RVA/Human-wt/BGD/RV161/2000/G12P[6]
JX943605-RVA/Human-tc/USA/Rotarix/2009/G1P[8]
KU861386-RVA/Human-tc/USA/Wa/1974/G1P[8]
LC173606-RVA/Human-xx/JPN/KN067/2013/G1P[8]
MH182455-RVA/Human-wt/PAK622/2016/G3P[8]
JX195092-RVA/Human-wt/ITA/JES11/2010/G9P[8]
KF371673-RVA/Human-tc/CHN/L148/2004/G3P[8]
JQ087430-RVA/Human-tc/CHN/Y128/2004/G1P[8]
DQ146678-RVA/Human/BGD/Matlab13/2003/G12P[6]
DQ146689-RVA/Human-wt/BGD/N26/2002/G12P[6]
DQ146656-RVA/Human-wt/BGD/Dhaka25/2002/G12P[8]
LC374210-RVA/Human-wt/NPL/10N4594/2010/G12P[6]
KC155684-RVA/Human-wt/RUS/Nov10-N921/2010/GXP[6]
KF371781-RVA/Human-wt/CHN/E1367/2008/G3P[8]
DQ492676-RVA/Human-wt/BGD/Dhaka16/2003/G1P[8]
JN013977-RVA/Human-wt/ZAF/2371WC/2008/G9P[8]
LC439276-RVA/Human-wt/GHA/M0094/2010/G9P[8]
RVA/Human-wt/RWA/UFS-NGS:MRC-DPRU566/2013/G9P[4]
MF184783-RVA/Human-wt/USA/CNMC109/2011/G3P[6]
KJ752932-RVA/Human-wt/ZAF/MRC-DPRU2711/2008/G9P[8]
KP752862-RVA/Human-wt/ZMB/MRC-DPRU1660/2008/G12P[6]
HQ392180-RVA/Human-wt/BEL/Be00023/2007/G1P[8]
KM116055-RVA/Human-wt/USA/2013774166/2013/G12P[8]
KU048682-RVA/Human-wt/ITA/PA99/12/2012/G12P[8]
MH171418-RVA/Human-wt/ESP/SS257451/2012/G12P[8]
MH171429-RVA/Human-wt/ESP/VI4816626/2012/G12P[8]
KX655535-RVA/Cow-wt/UGA/BUW-14-A035/2014/G12P[8]
KR262156-RVA/Human-tc/KOR/CAU14-1-262/2014/G3P[9]
N2
N1
Outgroup
0.05

G. NSP3

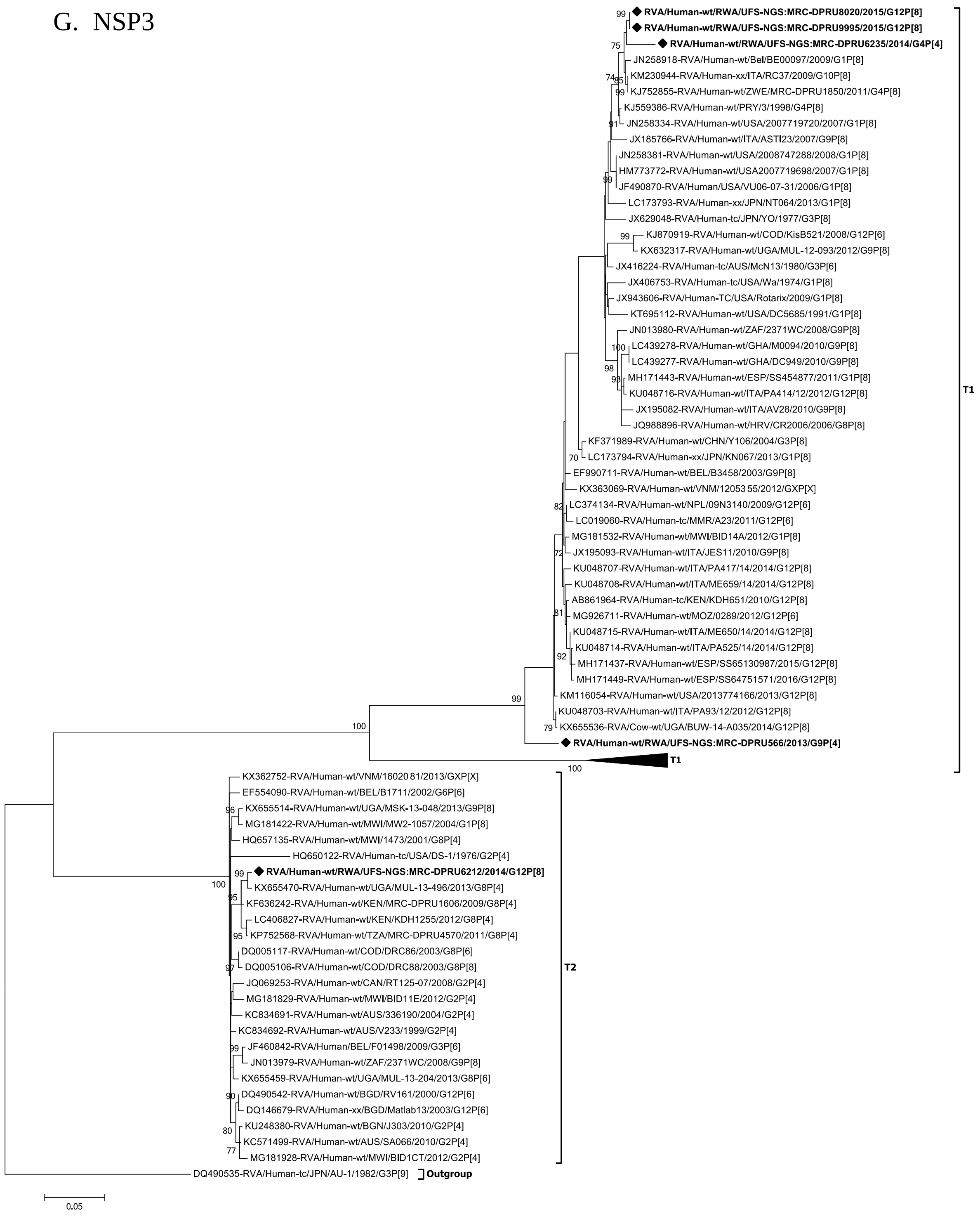

RVA/Human-wt/RWA/UFS-NGS:MRC-DPRU8020/2015/G12P[8]
RVA/Human-wt/RWA/UFS-NGS:MRC-DPRU9995/2015/G12P[8]
RVA/Human-wt/RWA/UFS-NGS:MRC-DPRU6235/2014/G4P[4]
JN258918-RVA/Human-wt/Bel/BE00097/2009/G1P[8]
KM230944-RVA/Human-xx/ITA/RC37/2009/G10P[8]
KJ752855-RVA/Human-wt/ZWE/MRC-DPRU1850/2011/G4P[8]
KJ559386-RVA/Human-wt/PRY/3/1998/G4P[8]
JN258334-RVA/Human-wt/USA/2007719720/2007/G1P[8]
JX185766-RVA/Human-wt/ITA/ASTI23/2007/G9P[8]
JN258381-RVA/Human-wt/USA/2008747288/2008/G1P[8]
HM773772-RVA/Human-wt/USA2007719698/2007/G1P[8]
JF490870-RVA/Human/USA/VU06-07-31/2006/G1P[8]
LC173793-RVA/Human-xx/JPN/NT064/2013/G1P[8]
JX629048-RVA/Human-tc/JPN/YO/1977/G3P[8]
KJ870919-RVA/Human-wt/COD/KisB521/2008/G12P[6]
KX632317-RVA/Human-wt/UGA/MUL-12-093/2012/G9P[8]
JX416224-RVA/Human-tc/AUS/McN13/1980/G3P[6]
JX406753-RVA/Human-tc/USA/Wa/1974/G1P[8]
JX943606-RVA/Human-TC/USA/Rotarix/2009/G1P[8]
KT695112-RVA/Human-wt/USA/DC5685/1991/G1P[8]
JN013980-RVA/Human-wt/ZAF/2371WC/2008/G9P[8]
LC439278-RVA/Human-wt/GHA/M0094/2010/G9P[8]
LC439277-RVA/Human-wt/GHA/DC949/2010/G9P[8]
MH171443-RVA/Human-wt/ESP/SS454877/2011/G1P[8]
KU048716-RVA/Human-wt/ITA/PA414/12/2012/G12P[8]
JX195082-RVA/Human-wt/ITA/AV28/2010/G9P[8]
JQ988896-RVA/Human-wt/HRV/CR2006/2006/G8P[8]
KF371989-RVA/Human-wt/CHN/Y106/2004/G3P[8]
LC173794-RVA/Human-xx/JPN/KN067/2013/G1P[8]
EF990711-RVA/Human-wt/BEL/B3458/2003/G9P[8]
KX363069-RVA/Human-wt/VNM/12053 55/2012/GXP[X]
LC374134-RVA/Human-wt/NPL/09N3140/2009/G12P[6]
LC019060-RVA/Human-tc/MMR/A23/2011/G12P[6]
MG181532-RVA/Human-wt/MWI/BID14A/2012/G1P[8]
JX195093-RVA/Human-wt/ITA/JES11/2010/G9P[8]
KU048707-RVA/Human-wt/ITA/PA417/14/2014/G12P[8]
KU048708-RVA/Human-wt/ITA/ME659/14/2014/G12P[8]
AB861964-RVA/Human-tc/KEN/KDH651/2010/G12P[8]
MG926711-RVA/Human-wt/MOZ/0289/2012/G12P[6]
KU048715-RVA/Human-wt/ITA/ME650/14/2014/G12P[8]
KU048714-RVA/Human-wt/ITA/PA525/14/2014/G12P[8]
MH171437-RVA/Human-wt/ESP/SS65130987/2015/G12P[8]
MH171449-RVA/Human-wt/ESP/SS64751571/2016/G12P[8]
KM116054-RVA/Human-wt/USA/2013774166/2013/G12P[8]
KU048703-RVA/Human-wt/ITA/PA93/12/2012/G12P[8]
KX655536-RVA/Cow-wt/UGA/BUW-14-A035/2014/G12P[8]
RVA/Human-wt/RWA/UFS-NGS:MRC-DPRU566/2013/G9P[4]
T1
T1
KX362752-RVA/Human-wt/VNM/16020 81/2013/GXP[X]
EF554090-RVA/Human-wt/BEL/B1711/2002/G6P[6]
KX655514-RVA/Human-wt/UGA/MSK-13-048/2013/G9P[8]
MG181422-RVA/Human-wt/MWI/MW2-1057/2004/G1P[8]
HQ657135-RVA/Human-wt/MWI/1473/2001/G8P[4]
HQ650122-RVA/Human-tc/USA/DS-1/1976/G2P[4]
RVA/Human-wt/RWA/UFS-NGS:MRC-DPRU6212/2014/G12P[8]
KX655470-RVA/Human-wt/UGA/MUL-13-496/2013/G8P[4]
KF636242-RVA/Human-wt/KEN/MRC-DPRU1606/2009/G8P[4]
LC406827-RVA/Human-wt/KEN/KDH1255/2012/G8P[4]
KP752568-RVA/Human-wt/TZA/MRC-DPRU4570/2011/G8P[4]
DQ005117-RVA/Human-wt/COD/DRC86/2003/G8P[6]
DQ005106-RVA/Human-wt/COD/DRC88/2003/G8P[8]
JQ069253-RVA/Human-wt/CAN/RT125-07/2008/G2P[4]
MG181829-RVA/Human-wt/MWI/BID11E/2012/G2P[4]
KC834691-RVA/Human-wt/AUS/336190/2004/G2P[4]
KC834692-RVA/Human-wt/AUS/V233/1999/G2P[4]
JF460842-RVA/Human/BEL/F01498/2009/G3P[6]
JN013979-RVA/Human-wt/ZAF/2371WC/2008/G9P[8]
KX655459-RVA/Human-wt/UGA/MUL-13-204/2013/G8P[6]
DQ490542-RVA/Human-wt/BGD/RV161/2000/G12P[6]
DQ146679-RVA/Human-xx/BGD/Matlab13/2003/G12P[6]
KU248380-RVA/Human-wt/BGN/J303/2010/G2P[4]
KC571499-RVA/Human-wt/AUS/SA066/2010/G2P[4]
MG181928-RVA/Human-wt/MWI/BID1CT/2012/G2P[4]
T2
DQ490535-RVA/Human-tc/JPN/AU-1/1982/G3P[9]
Outgroup
0.05

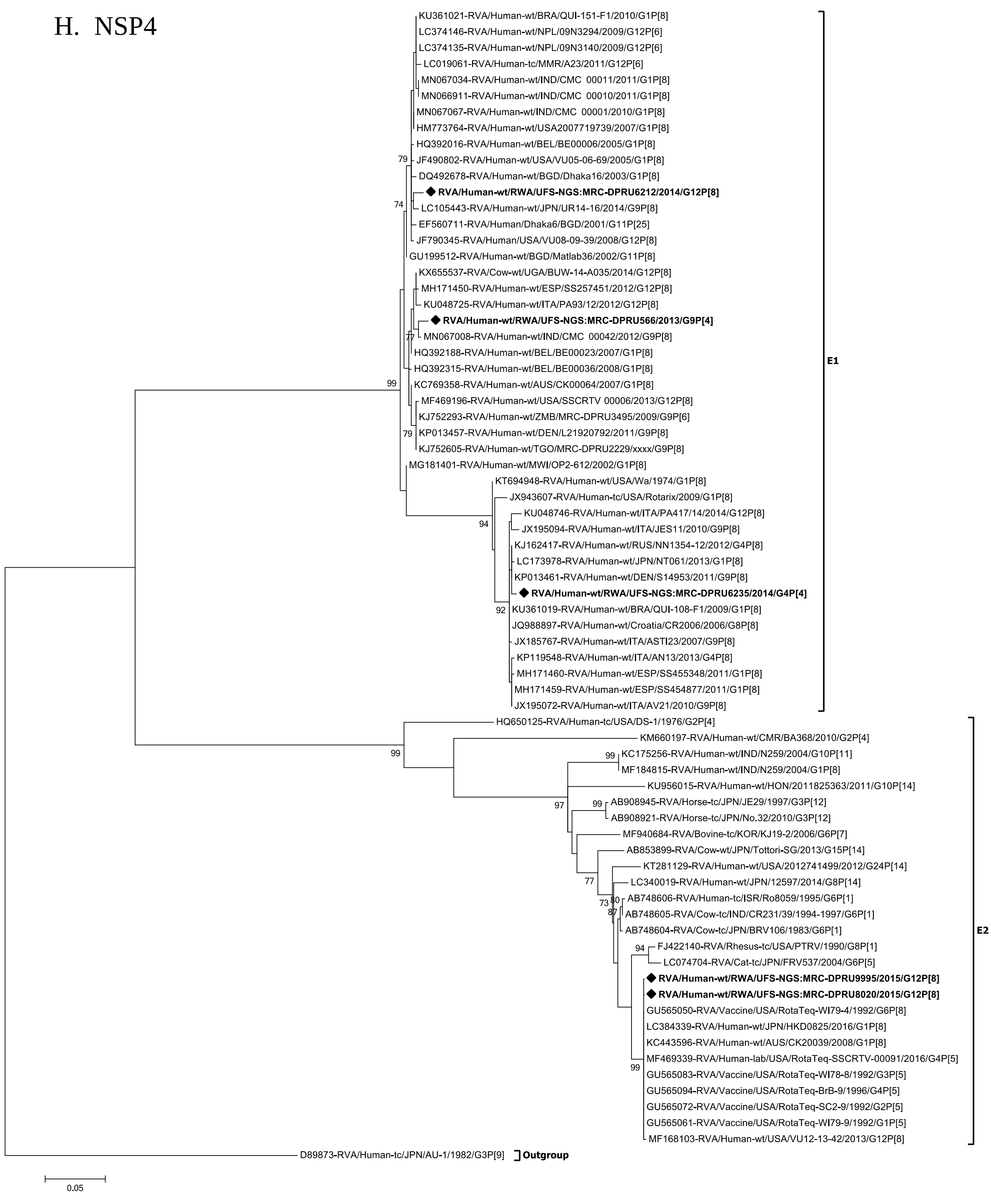
H. NSP4
KU361021-RVA/Human-wt/BRA/QUI-151-F1/2010/G1P[8]
LC374146-RVA/Human-wt/NPL/09N3294/2009/G12P[6]
LC374135-RVA/Human-wt/NPL/09N3140/2009/G12P[6]
LC019061-RVA/Human-tc/MMR/A23/2011/G12P[6]
MN067034-RVA/Human-wt/IND/CMC 00011/2011/G1P[8]
MN066911-RVA/Human-wt/IND/CMC 00010/2011/G1P[8]
MN067067-RVA/Human-wt/IND/CMC 00001/2010/G1P[8]
HM773764-RVA/Human-wt/USA2007719739/2007/G1P[8]
HQ392016-RVA/Human-wt/BEL/BE00006/2005/G1P[8]
JF490802-RVA/Human-wt/USA/VU05-06-69/2005/G1P[8]
DQ492678-RVA/Human-wt/BGD/Dhaka16/2003/G1P[8]
RVA/Human-wt/RWA/UFS-NGS:MRC-DPRU6212/2014/G12P[8]
LC105443-RVA/Human-wt/JPN/UR14-16/2014/G9P[8]
EF560711-RVA/Human/Dhaka6/BGD/2001/G11P[25]
JF790345-RVA/Human/USA/VU08-09-39/2008/G12P[8]
GU199512-RVA/Human-wt/BGD/Matlab36/2002/G11P[8]
KX655537-RVA/Cow-wt/UGA/BUW-14-A035/2014/G12P[8]
MH171450-RVA/Human-wt/ESP/SS257451/2012/G12P[8]
KU048725-RVA/Human-wt/ITA/PA93/12/2012/G12P[8]
RVA/Human-wt/RWA/UFS-NGS:MRC-DPRU566/2013/G9P[4]
MN067008-RVA/Human-wt/IND/CMC 00042/2012/G9P[8]
HQ392188-RVA/Human-wt/BEL/BE00023/2007/G1P[8]
HQ392315-RVA/Human-wt/BEL/BE00036/2008/G1P[8]
KC769358-RVA/Human-wt/AUS/CK00064/2007/G1P[8]
MF469196-RVA/Human-wt/USA/SSCRTV 00006/2013/G12P[8]
KJ752293-RVA/Human-wt/ZMB/MRC-DPRU3495/2009/G9P[6]
KP013457-RVA/Human-wt/DEN/L21920792/2011/G9P[8]
KJ752605-RVA/Human-wt/TGO/MRC-DPRU2229/xxxx/G9P[8]
MG181401-RVA/Human-wt/MWI/OP2-612/2002/G1P[8]
KT694948-RVA/Human-wt/USA/Wa/1974/G1P[8]
JX943607-RVA/Human-tc/USA/Rotarix/2009/G1P[8]
KU048746-RVA/Human-wt/ITA/PA417/14/2014/G12P[8]
JX195094-RVA/Human-wt/ITA/JES11/2010/G9P[8]
KJ162417-RVA/Human-wt/RUS/NN1354-12/2012/G4P[8]
LC173978-RVA/Human-wt/JPN/NT061/2013/G1P[8]
KP013461-RVA/Human-wt/DEN/S14953/2011/G9P[8]
RVA/Human-wt/RWA/UFS-NGS:MRC-DPRU6235/2014/G4P[4]
KU361019-RVA/Human-wt/BRA/QUI-108-F1/2009/G1P[8]
JQ988897-RVA/Human-wt/Croatia/CR2006/2006/G8P[8]
JX185767-RVA/Human-wt/ITA/ASTI23/2007/G9P[8]
KP119548-RVA/Human-wt/ITA/AN13/2013/G4P[8]
MH171460-RVA/Human-wt/ESP/SS455348/2011/G1P[8]
MH171459-RVA/Human-wt/ESP/SS454877/2011/G1P[8]
JX195072-RVA/Human-wt/ITA/AV21/2010/G9P[8]
E1
79
74
77
99
79
94
92
HQ650125-RVA/Human-tc/USA/DS-1/1976/G2P[4]
KM660197-RVA/Human-wt/CMR/BA368/2010/G2P[4]
KC175256-RVA/Human-wt/IND/N259/2004/G10P[11]
MF184815-RVA/Human-wt/IND/N259/2004/G1P[8]
KU956015-RVA/Human-wt/HON/2011825363/2011/G10P[14]
AB908945-RVA/Horse-tc/JPN/JE29/1997/G3P[12]
AB908921-RVA/Horse-tc/JPN/No.32/2010/G3P[12]
MF940684-RVA/Bovine-tc/KOR/KJ19-2/2006/G6P[7]
AB853899-RVA/Cow-wt/JPN/Tottori-SG/2013/G15P[14]
KT281129-RVA/Human-wt/USA/2012741499/2012/G24P[14]
LC340019-RVA/Human-wt/JPN/12597/2014/G8P[14]
AB748606-RVA/Human-tc/ISR/Ro8059/1995/G6P[1]
AB748605-RVA/Cow-tc/IND/CR231/39/1994-1997/G6P[1]
AB748604-RVA/Cow-tc/JPN/BRV106/1983/G6P[1]
FJ422140-RVA/Rhesus-tc/USA/PTRV/1990/G8P[1]
LC074704-RVA/Cat-tc/JPN/FRV537/2004/G6P[5]
RVA/Human-wt/RWA/UFS-NGS:MRC-DPRU9995/2015/G12P[8]
RVA/Human-wt/RWA/UFS-NGS:MRC-DPRU8020/2015/G12P[8]
GU565050-RVA/Vaccine/USA/RotaTeq-WI79-4/1992/G6P[8]
LC384339-RVA/Human-wt/JPN/HKD0825/2016/G1P[8]
KC443596-RVA/Human-wt/AUS/CK20039/2008/G1P[8]
MF469339-RVA/Human-lab/USA/RotaTeq-SSCRTV-00091/2016/G4P[5]
GU565083-RVA/Vaccine/USA/RotaTeq-WI78-8/1992/G3P[5]
GU565094-RVA/Vaccine/USA/RotaTeq-BrB-9/1996/G4P[5]
GU565072-RVA/Vaccine/USA/RotaTeq-SC2-9/1992/G2P[5]
GU565061-RVA/Vaccine/USA/RotaTeq-WI79-9/1992/G1P[5]
MF168103-RVA/Human-wt/USA/VU12-13-42/2013/G12P[8]
E2
99
99
97
99
77
73
80
87
94
99
D89873-RVA/Human-tc/JPN/AU-1/1982/G3P[9]
Outgroup
0,05

I. NSP5

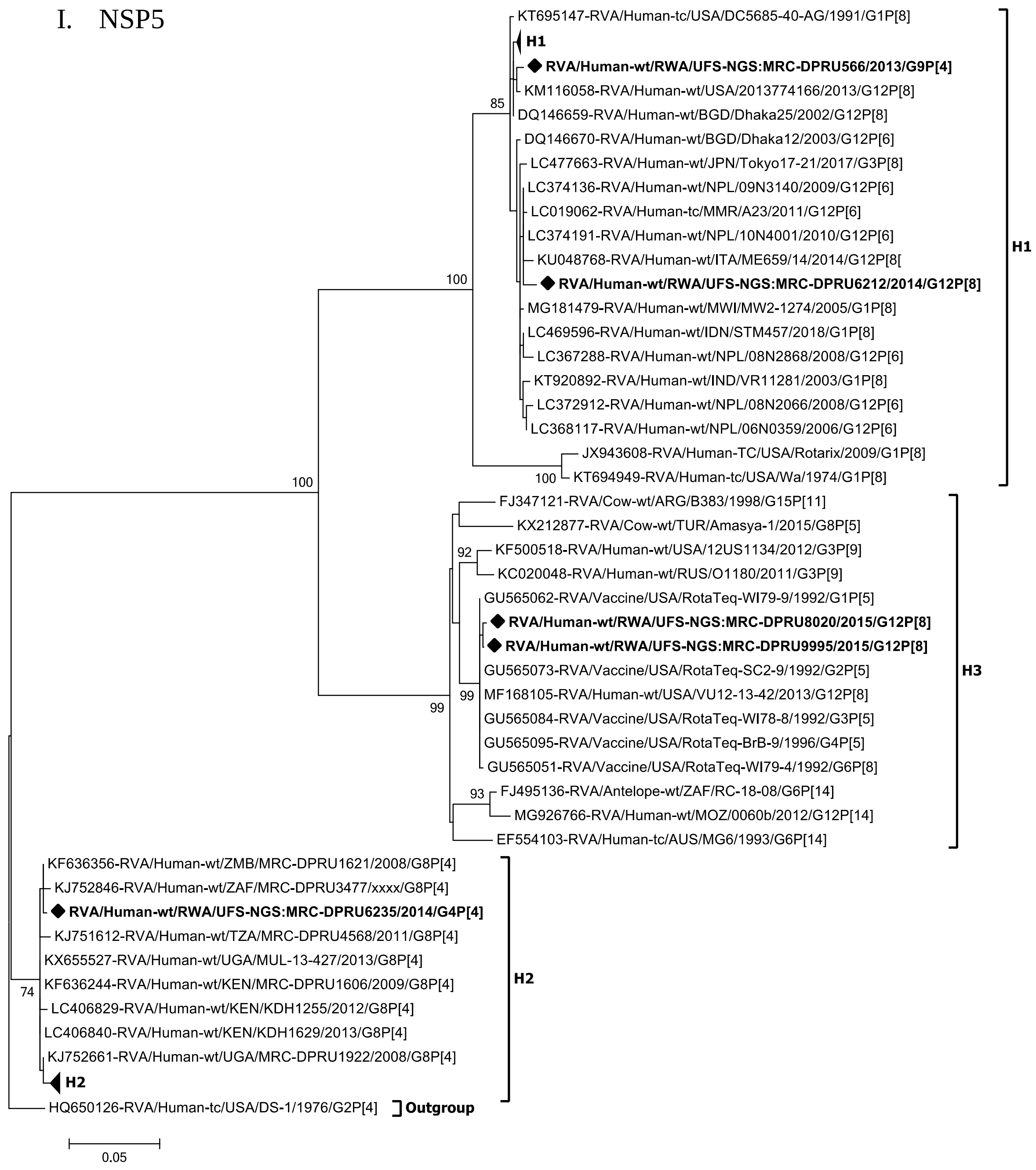

Supplementary 1 (S1): A) – I) Phylogenetic relatedness of group A species based on A) VP6 B) VP1 C) VP2 D) VP3 E) NSP1 F) NSP2 G) NSP3 H) NSP4 I) NSP5 of the study strains from Rwanda. The study strains are indicated with a black diamond. Bootstrap values ≥ 70% are indicated at each branch node. Scale bars represents substitutions per nucleotide site.

A. VP6

	**RVA/Human-wt/RWA/UFS-NGS:MRC-DPRU566/2013/G9P[4]**	
1	KJ752940-RVA/Human-wt/ZAF/MRC-DPRU2711/2008/G9P[8]	95,0
2	KJ753372-RVA/Human-wt/ZAF/MRC-DPRU2943/2008/G9P[8]	94,9
3	KJ752611-RVA/Human-wt/TGO/MRC-DPRU2229/XXXX/G9P[8]	94,9
4	KP752870-RVA/Human-wt/ZMB/MRC-DPRU1660/2008/G12P[6]	94,7
5	HQ392316-RVA/Human-wt/BEL/BE00036/2008/G1P[8]	94,5
6	KJ753428-RVA/Human-wt/UGA/MRC-DPRU4595/2011/G9P[8]	94,4
7	HQ392237-RVA/Human-wt/BEL/BE00029/2008/G1P[8]	94,4
8	JF490090-RVA/human/CAN/CK00001/2004/G1P[8]	94,4
9	JF490200-RVA/human/CAN/CK00012/2004/G1P[8]	94,4
10	KC769364-RVA/Human-wt/AUS/CK00064/2007/G1P[8]	94,3
11	KJ870926-RVA/Human-wt/COD/KisB504/2009/G1P[6]	94,2
12	KX632324-RVA/Human-wt/UGA/MUL-13-285/2013/G9P[8]	94,2
13	KX632247-RVA/Human-wt/UGA/NSA-13-043/2013/G9P[8]	94,2
14	MF168284-RVA/Human-wt/USA/VU12-13-157/2013/G12P[8]	94,2
15	KJ627120-RVA/Human-wt/PRY/371/1999/G1P[8]	94,2
16	KJ870915-RVA/Human-wt/COD/KisB521/2008/G12P[6]	94,1
17	KX632302-RVA/Human-wt/UGA/MUL-12-147/2012/G9P[8]	94,1
18	KM116053-RVA/Human-wt/USA/2013774166/2013/G12P[8]	94,1
19	KX632291-RVA/Human-wt/UGA/MUL-13-163/2013/G9P[8]	94,0
20	KJ753296-RVA/Human-wt/ZWE/MRC-DPRU1844-11/2011/G1P[	94,0
21	KM116042-RVA/Human-wt/USA/2013774165/2013/G12P[8]	94,0
22	KF636293-RVA/Human-wt/CMR/MRC-DPRU1417/2009/G9P[8]	93,8
23	KX655532-RVA/Cow-wt/UGA/BUW-14-A035/2014/G12P[8]	93,8
24	KX655532-RVA/Cow-wt/UGA/BUW-14-A035/2014/G12P[8]	93,8
25	MH171381-RVA/Human-wt/ESP/VI4816626/2012/G12P[8]	93,7
26	KU048615-RVA/Human-wt/ITA/PA93/12/2012/G12P[8]	93,6
27	**RVA/Human-wt/RWA/UFS-NGS:MRC-DPRU6212/2014/G12P[8**	**93,4**
28	JQ818154-RVA/Human/RUS/Omsk08-425/2008/G1P[8]	91,2
29	**RVA/Human-wt/RWA/UFS-NGS:MRC-DPRU9995/2015/G12P[8**	**91,1**
30	KC890877-RVA/Human-wt/GR/Ath113/2009/G4P[8]	91,0
31	KP119547-RVA/Human-wt/ITA/PG55/2013/G4P[8]	91,0
32	JQ585598-RVA/Human/RUS/Nov06-1255/2006/G4P[8]	91,0
33	KJ752862-RVA/Human-wt/ZWE/MRC-DPRU1850/2011/G4P[8]	91,0
34	JQ230093-RVA/Human-wt/RUS/Nov10-N552/2010/G3P[8]	90,9
35	JQ585600-RVA/Human/RUS/Nov07-2058/2007/G1P[8]	90,9
36	HQ392205-RVA/Human-wt/BEL/BE00025/2007/G1P[8]	90,9
37	KC713889-RVA/Human/RUS/Nov12-N4252/2012/G4P[8]	90,9
38	MH473471-RVA/Human-wt/RUS/Nov12-N3593/2012/GXP[8]	90,9
39	KP119544-RVA/Human-wt/ITA/BZ52/2013/G4P[8]	90,9
40	MH484058-RVA/Hu-wt/RUS/S13-118/2013/G4P[8]	90,8
41	**RVA/Human-wt/RWA/UFS-NGS:MRC-DPRU8020/2015/G12P[8**	**90,8**
42	KJ162413-RVA/Human-wt/RUS/NN934-12/2012/G4P[8]	90,8
43	JQ713098-RVA/Human/RUS/Nov07-1971/2007/G1P[8]	90,7
44	JX867352-RVA/Human/RUS/O903/2010/GXP[8]	90,3
45	JX943613-RVA/Human-TC/USA/Rotarix/2009/G1P[8]	85,3
46	KU861383-RVA/Human-tc/USA/Wa/1974/G1P[8]	85,1
47	**RVA/Human-wt/RWA/UFS-NGS:MRC-DPRU6235/2014/G4P[4]**	**84,1**
48	KC834700-RVA/Human-wt/AUS/336190/2004/G2P[4]	77,1
49	DQ870507-RVA/Human-tc/USA/DS-1/1976/G2P[4]	77,0
50	JQ069584-RVA/Human-wt/CAN/RT125-07/2008/G2P[4]	76,9
51	KJ919810-RVA/Human-wt/HUN/ERN5635/2012/G2P[4]	76,9
52	GU565045-RVA/Vaccine/USA/RotaTeq-WI79-4/1992/G6P1[8]	76,8
53	GU565089-RVA/Vaccine/USA/RotaTeq-BrB-9/1996/G4P7[5]	76,7
54	GU565067-RVA/Vaccine/USA/RotaTeq-SC2-9/1992/G2P7[5]	76,7
55	GU565056-RVA/Vaccine/USA/RotaTeq-WI79-9/1992/G1P7[5]	76,7
56	GU565078-RVA/Vaccine/USA/RotaTeq-WI78-8/1992/G3P7[5]	76,6
57	KM660383-RVA/Human-wt/CMR/BA368/2010/G2P[4]	76,4
58	KC834699-RVA/Human-wt/AUS/V203/2009/G2P[4]	76,2
59	DQ490538-RVA/Human-tc/JPN/AU-1/1982/G3P3[9]	71,5

	**RVA/Human-wt/RWA/UFS-NGS:MRC-DPRU6235/2014/G4P[4]**	
1	KC890877-RVA/Human-wt/GR/Ath113/2009/G4P[8]	91,6
2	**RVA/Human-wt/RWA/UFS-NGS:MRC-DPRU8020/2015/G12P[8]**	**91,4**
3	**RVA/Human-wt/RWA/UFS-NGS:MRC-DPRU9995/2015/G12P[8]**	**91,2**
4	JQ818154-RVA/Human/RUS/Omsk08-425/2008/G1P[8]	91,1
5	JQ585598-RVA/Human/RUS/Nov06-1255/2006/G4P[8]	91,1
6	JQ585600-RVA/Human/RUS/Nov07-2058/2007/G1P[8]	91,0
7	HQ392205-RVA/Human-wt/BEL/BE00025/2007/G1P[8]	91,0
8	JQ230093-RVA/Human-wt/RUS/Nov10-N552/2010/G3P[8]	91,0
9	KP119547-RVA/Human-wt/ITA/PG55/2013/G4P[8]	91,0
10	KJ752862-RVA/Human-wt/ZWE/MRC-DPRU1850/2011/G4P[8]	91,0
11	KC713889-RVA/Human/RUS/Nov12-N4252/2012/G4P[8]	90,9
12	MH473471-RVA/Human-wt/RUS/Nov12-N3593/2012/GXP[8]	90,9
13	MH484058-RVA/Hu-wt/RUS/S13-118/2013/G4P[8]	90,9
14	KP119544-RVA/Human-wt/ITA/BZ52/2013/G4P[8]	90,9
15	JQ713098-RVA/Human/RUS/Nov07-1971/2007/G1P[8]	90,8
16	JX867352-RVA/Human/RUS/O903/2010/GXP[8]	90,6
17	KJ162413-RVA/Human-wt/RUS/NN934-12/2012/G4P[8]	90,6
18	KJ627120-RVA/Human-wt/PRY/371/1999/G1P[8]	89,1
19	KC769364-RVA/Human-wt/AUS/CK00064/2007/G1P[8]	88,8
20	KJ753296-RVA/Human-wt/ZWE/MRC-DPRU1844-11/2011/G1P[8]	88,7
21	JF490090-RVA/human/CAN/CK00001/2004/G1P[8]	88,7
22	KJ870915-RVA/Human-wt/COD/KisB521/2008/G12P[6]	88,6
23	JF490200-RVA/human/CAN/CK00012/2004/G1P[8]	88,6
24	HQ392316-RVA/Human-wt/BEL/BE00036/2008/G1P[8]	88,6
25	KJ753428-RVA/Human-wt/UGA/MRC-DPRU4595/2011/G9P[8]	88,6
26	KJ870926-RVA/Human-wt/COD/KisB504/2009/G1P[6]	88,5
27	KF636293-RVA/Human-wt/CMR/MRC-DPRU1417/2009/G9P[8]	88,5
28	HQ392237-RVA/Human-wt/BEL/BE00029/2008/G1P[8]	88,5
29	MF168284-RVA/Human-wt/USA/VU12-13-157/2013/G12P[8]	88,4
30	KP752870-RVA/Human-wt/ZMB/MRC-DPRU1660/2008/G12P[6]	88,4
31	KX632247-RVA/Human-wt/UGA/NSA-13-043/2013/G9P[8]	88,4
32	KX655532-RVA/Cow-wt/UGA/BUW-14-A035/2014/G12P[8]	88,4
33	KX655532-RVA/Cow-wt/UGA/BUW-14-A035/2014/G12P[8]	88,4
34	KJ752940-RVA/Human-wt/ZAF/MRC-DPRU2711/2008/G9P[8]	88,3
35	KX632302-RVA/Human-wt/UGA/MUL-12-147/2012/G9P[8]	88,3
36	KX632291-RVA/Human-wt/UGA/MUL-13-163/2013/G9P[8]	88,3
37	MH171381-RVA/Human-wt/ESP/VI4816626/2012/G12P[8]	88,3
38	KM116053-RVA/Human-wt/USA/2013774166/2013/G12P[8]	88,3
39	KM116042-RVA/Human-wt/USA/2013774165/2013/G12P[8]	88,3
40	KX632324-RVA/Human-wt/UGA/MUL-13-285/2013/G9P[8]	88,3
41	KJ753372-RVA/Human-wt/ZAF/MRC-DPRU2943/2008/G9P[8]	88,2
42	KU048615-RVA/Human-wt/ITA/PA93/12/2012/G12P[8]	88,2
43	KJ752611-RVA/Human-wt/TGO/MRC-DPRU2229/XXXX/G9P[8]	88,2
44	**RVA/Human-wt/RWA/UFS-NGS:MRC-DPRU6212/2014/G12P[8]**	**87,9**
45	**RVA/Human-wt/RWA/UFS-NGS:MRC-DPRU566/2013/G9P[4]**	**84,1**
46	KU861383-RVA/Human-tc/USA/Wa/1974/G1P[8]	83,1
47	JX943613-RVA/Human-TC/USA/Rotarix/2009/G1P[8]	83,1
48	KC834700-RVA/Human-wt/AUS/336190/2004/G2P[4]	82,9
49	JQ069584-RVA/Human-wt/CAN/RT125-07/2008/G2P[4]	82,1
50	KC834699-RVA/Human-wt/AUS/V203/2009/G2P[4]	81,6
51	KM660383-RVA/Human-wt/CMR/BA368/2010/G2P[4]	81,5
52	GU565045-RVA/Vaccine/USA/RotaTeq-WI79-4/1992/G6P1[8]	80,7
53	GU565089-RVA/Vaccine/USA/RotaTeq-BrB-9/1996/G4P7[5]	80,5
54	GU565067-RVA/Vaccine/USA/RotaTeq-SC2-9/1992/G2P7[5]	80,5
55	GU565056-RVA/Vaccine/USA/RotaTeq-WI79-9/1992/G1P7[5]	80,5
56	GU565078-RVA/Vaccine/USA/RotaTeq-WI78-8/1992/G3P7[5]	80,4
57	KJ919810-RVA/Human-wt/HUN/ERN5635/2012/G2P[4]	79,6
58	DQ870507-RVA/Human-tc/USA/DS-1/1976/G2P[4]	77,4
59	DQ490538-RVA/Human-tc/JPN/AU-1/1982/G3P3[9]	70,4

	**RVA/Human-wt/RWA/UFS-NGS:MRC-DPRU8020/2015/G12P[8]**	
1	**RVA/Human-wt/RWA/UFS-NGS:MRC-DPRU9995/2015/G12P[8**	**99,4**
2	JQ818154-RVA/Human/RUS/Omsk08-425/2008/G1P[8]	98,7
3	JQ585598-RVA/Human/RUS/Nov06-1255/2006/G4P[8]	98,7
4	JQ585600-RVA/Human/RUS/Nov07-2058/2007/G1P[8]	98,6
5	HQ392205-RVA/Human-wt/BEL/BE00025/2007/G1P[8]	98,6
6	KC890877-RVA/Human-wt/GR/Ath113/2009/G4P[8]	98,5
7	JQ713098-RVA/Human/RUS/Nov07-1971/2007/G1P[8]	98,5
8	JQ230093-RVA/Human-wt/RUS/Nov10-N552/2010/G3P[8]	98,5
9	KP119547-RVA/Human-wt/ITA/PG55/2013/G4P[8]	98,5
10	KJ752862-RVA/Human-wt/ZWE/MRC-DPRU1850/2011/G4P[8]	98,5
11	KC713889-RVA/Human/RUS/Nov12-N4252/2012/G4P[8]	98,4
12	MH473471-RVA/Human-wt/RUS/Nov12-N3593/2012/GXP[8]	98,4
13	KP119544-RVA/Human-wt/ITA/BZ52/2013/G4P[8]	98,4
14	MH484058-RVA/Hu-wt/RUS/S13-118/2013/G4P[8]	98,3
15	JX867352-RVA/Human/RUS/O903/2010/GXP[8]	98,1
16	KJ162413-RVA/Human-wt/RUS/NN934-12/2012/G4P[8]	98,1
17	JF490200-RVA/human/CAN/CK00012/2004/G1P[8]	95,3
18	JF490090-RVA/human/CAN/CK00001/2004/G1P[8]	95,2
19	KJ627120-RVA/Human-wt/PRY/371/1999/G1P[8]	95,2
20	KJ753428-RVA/Human-wt/UGA/MRC-DPRU4595/2011/G9P[8]	95,0
21	KC769364-RVA/Human-wt/AUS/CK00064/2007/G1P[8]	95,0
22	KX632247-RVA/Human-wt/UGA/NSA-13-043/2013/G9P[8]	94,9
23	KJ870915-RVA/Human-wt/COD/KisB521/2008/G12P[6]	94,8
24	KX632302-RVA/Human-wt/UGA/MUL-12-147/2012/G9P[8]	94,8
25	KX632324-RVA/Human-wt/UGA/MUL-13-285/2013/G9P[8]	94,8
26	MF168284-RVA/Human-wt/USA/VU12-13-157/2013/G12P[8]	94,7
27	KJ752940-RVA/Human-wt/ZAF/MRC-DPRU2711/2008/G9P[8]	94,7
28	KJ870926-RVA/Human-wt/COD/KisB504/2009/G1P[6]	94,7
29	KX632291-RVA/Human-wt/UGA/MUL-13-163/2013/G9P[8]	94,7
30	KJ753296-RVA/Human-wt/ZWE/MRC-DPRU1844-11/2011/G1P[	94,7
31	KF636293-RVA/Human-wt/CMR/MRC-DPRU1417/2009/G9P[8]	94,7
32	KM116053-RVA/Human-wt/USA/2013774166/2013/G12P[8]	94,6
33	KM116042-RVA/Human-wt/USA/2013774165/2013/G12P[8]	94,6
34	KJ753372-RVA/Human-wt/ZAF/MRC-DPRU2943/2008/G9P[8]	94,6
35	KP752870-RVA/Human-wt/ZMB/MRC-DPRU1660/2008/G12P[6]	94,6
36	KJ752611-RVA/Human-wt/TGO/MRC-DPRU2229/XXXX/G9P[8]	94,6
37	HQ392316-RVA/Human-wt/BEL/BE00036/2008/G1P[8]	94,6
38	KX655532-RVA/Cow-wt/UGA/BUW-14-A035/2014/G12P[8]	94,5
39	KX655532-RVA/Cow-wt/UGA/BUW-14-A035/2014/G12P[8]	94,5
40	HQ392237-RVA/Human-wt/BEL/BE00029/2008/G1P[8]	94,5
41	MH171381-RVA/Human-wt/ESP/VI4816626/2012/G12P[8]	94,4
42	**RVA/Human-wt/RWA/UFS-NGS:MRC-DPRU6212/2014/G12P[8**	**94,3**
43	KU048615-RVA/Human-wt/ITA/PA93/12/2012/G12P[8]	94,3
44	**RVA/Human-wt/RWA/UFS-NGS:MRC-DPRU6235/2014/G4P[4]**	**91,4**
45	**RVA/Human-wt/RWA/UFS-NGS:MRC-DPRU566/2013/G9P[4]**	**90,8**
46	JX943613-RVA/Human-TC/USA/Rotarix/2009/G1P[8]	87,5
47	KU861383-RVA/Human-tc/USA/Wa/1974/G1P[8]	87,5
48	DQ870507-RVA/Human-tc/USA/DS-1/1976/G2P[4]	72,8
49	GU565045-RVA/Vaccine/USA/RotaTeq-WI79-4/1992/G6P1[8]	72,1
50	KJ919810-RVA/Human-wt/HUN/ERN5635/2012/G2P[4]	72,0
51	GU565089-RVA/Vaccine/USA/RotaTeq-BrB-9/1996/G4P7[5]	71,9
52	GU565067-RVA/Vaccine/USA/RotaTeq-SC2-9/1992/G2P7[5]	71,9
53	GU565056-RVA/Vaccine/USA/RotaTeq-WI79-9/1992/G1P7[5]	71,9
54	GU565078-RVA/Vaccine/USA/RotaTeq-WI78-8/1992/G3P7[5]	71,8
55	JQ069584-RVA/Human-wt/CAN/RT125-07/2008/G2P[4]	71,6
56	KC834700-RVA/Human-wt/AUS/336190/2004/G2P[4]	71,2
57	KC834699-RVA/Human-wt/AUS/V203/2009/G2P[4]	71,0
58	KM660383-RVA/Human-wt/CMR/BA368/2010/G2P[4]	70,9
59	DQ490538-RVA/Human-tc/JPN/AU-1/1982/G3P3[9]	68,3

	**RVA/Human-wt/RWA/UFS-NGS:MRC-DPRU9995/2015/G12P[8]**	
1	**RVA/Human-wt/RWA/UFS-NGS:MRC-DPRU8020/2015/G12P[8]**	**99,4**
2	JQ818154-RVA/Human/RUS/Omsk08-425/2008/G1P[8]	99,3
3	JQ585598-RVA/Human/RUS/Nov06-1255/2006/G4P[8]	99,3
4	JQ585600-RVA/Human/RUS/Nov07-2058/2007/G1P[8]	99,2
5	HQ392205-RVA/Human-wt/BEL/BE00025/2007/G1P[8]	99,2
6	KC890877-RVA/Human-wt/GR/Ath113/2009/G4P[8]	99,2
7	JQ713098-RVA/Human/RUS/Nov07-1971/2007/G1P[8]	99,1
8	JQ230093-RVA/Human-wt/RUS/Nov10-N552/2010/G3P[8]	99,1
9	KP119547-RVA/Human-wt/ITA/PG55/2013/G4P[8]	99,1
10	KJ752862-RVA/Human-wt/ZWE/MRC-DPRU1850/2011/G4P[8]	99,1
11	KC713889-RVA/Human/RUS/Nov12-N4252/2012/G4P[8]	99,0
12	MH473471-RVA/Human-wt/RUS/Nov12-N3593/2012/GXP[8]	99,0
13	KP119544-RVA/Human-wt/ITA/BZ52/2013/G4P[8]	99,0
14	MH484058-RVA/Hu-wt/RUS/S13-118/2013/G4P[8]	98,9
15	JX867352-RVA/Human/RUS/O903/2010/GXP[8]	98,7
16	KJ162413-RVA/Human-wt/RUS/NN934-12/2012/G4P[8]	98,7
17	JF490200-RVA/human/CAN/CK00012/2004/G1P[8]	96,0
18	JF490090-RVA/human/CAN/CK00001/2004/G1P[8]	95,9
19	KJ627120-RVA/Human-wt/PRY/371/1999/G1P[8]	95,9
20	KJ753428-RVA/Human-wt/UGA/MRC-DPRU4595/2011/G9P[8]	95,7
21	KC769364-RVA/Human-wt/AUS/CK00064/2007/G1P[8]	95,6
22	KX632247-RVA/Human-wt/UGA/NSA-13-043/2013/G9P[8]	95,5
23	KJ870915-RVA/Human-wt/COD/KisB521/2008/G12P[6]	95,4
24	KX632302-RVA/Human-wt/UGA/MUL-12-147/2012/G9P[8]	95,4
25	KX632324-RVA/Human-wt/UGA/MUL-13-285/2013/G9P[8]	95,4
26	MF168284-RVA/Human-wt/USA/VU12-13-157/2013/G12P[8]	95,3
27	KJ752940-RVA/Human-wt/ZAF/MRC-DPRU2711/2008/G9P[8]	95,3
28	KJ870926-RVA/Human-wt/COD/KisB504/2009/G1P[6]	95,3
29	KX632291-RVA/Human-wt/UGA/MUL-13-163/2013/G9P[8]	95,3
30	KJ753296-RVA/Human-wt/ZWE/MRC-DPRU1844-11/2011/G1P[	95,3
31	KF636293-RVA/Human-wt/CMR/MRC-DPRU1417/2009/G9P[8]	95,3
32	KM116053-RVA/Human-wt/USA/2013774166/2013/G12P[8]	95,2
33	KM116042-RVA/Human-wt/USA/2013774165/2013/G12P[8]	95,2
34	KJ753372-RVA/Human-wt/ZAF/MRC-DPRU2943/2008/G9P[8]	95,2
35	KP752870-RVA/Human-wt/ZMB/MRC-DPRU1660/2008/G12P[6]	95,2
36	KJ752611-RVA/Human-wt/TGO/MRC-DPRU2229/XXXX/G9P[8]	95,2
37	HQ392316-RVA/Human-wt/BEL/BE00036/2008/G1P[8]	95,2
38	KX655532-RVA/Cow-wt/UGA/BUW-14-A035/2014/G12P[8]	95,2
39	KX655532-RVA/Cow-wt/UGA/BUW-14-A035/2014/G12P[8]	95,2
40	HQ392237-RVA/Human-wt/BEL/BE00029/2008/G1P[8]	95,1
41	MH171381-RVA/Human-wt/ESP/VI4816626/2012/G12P[8]	95,1
42	**RVA/Human-wt/RWA/UFS-NGS:MRC-DPRU6212/2014/G12P[8]**	**95,0**
43	KU048615-RVA/Human-wt/ITA/PA93/12/2012/G12P[8]	95,0
44	**RVA/Human-wt/RWA/UFS-NGS:MRC-DPRU6235/2014/G4P[4]**	**91,2**
45	**RVA/Human-wt/RWA/UFS-NGS:MRC-DPRU566/2013/G9P[4]**	**91,1**
46	JX943613-RVA/Human-TC/USA/Rotarix/2009/G1P[8]	88,0
47	KU861383-RVA/Human-tc/USA/Wa/1974/G1P[8]	87,9
48	DQ870507-RVA/Human-tc/USA/DS-1/1976/G2P[4]	73,6
49	GU565045-RVA/Vaccine/USA/RotaTeq-WI79-4/1992/G6P1[8]	71,9
50	KJ919810-RVA/Human-wt/HUN/ERN5635/2012/G2P[4]	71,8
51	GU565089-RVA/Vaccine/USA/RotaTeq-BrB-9/1996/G4P7[5]	71,7
52	GU565067-RVA/Vaccine/USA/RotaTeq-SC2-9/1992/G2P7[5]	71,7
53	GU565056-RVA/Vaccine/USA/RotaTeq-WI79-9/1992/G1P7[5]	71,7
54	GU565078-RVA/Vaccine/USA/RotaTeq-WI78-8/1992/G3P7[5]	71,6
55	JQ069584-RVA/Human-wt/CAN/RT125-07/2008/G2P[4]	71,4
56	KC834700-RVA/Human-wt/AUS/336190/2004/G2P[4]	71,1
57	KM660383-RVA/Human-wt/CMR/BA368/2010/G2P[4]	70,9
58	KC834699-RVA/Human-wt/AUS/V203/2009/G2P[4]	70,8
59	DQ490538-RVA/Human-tc/JPN/AU-1/1982/G3P3[9]	68,8

	**RVA/Human-wt/RWA/UFS-NGS:MRC-DPRU6212/2014/G12P[8]**	
1	KJ870915-RVA/Human-wt/COD/KisB521/2008/G12P[6]	99,1
2	KJ870926-RVA/Human-wt/COD/KisB504/2009/G1P[6]	99,0
3	KJ753296-RVA/Human-wt/ZWE/MRC-DPRU1844-11/2011/G1P[	99,0
4	KX632302-RVA/Human-wt/UGA/MUL-12-147/2012/G9P[8]	98,6
5	KX632247-RVA/Human-wt/UGA/NSA-13-043/2013/G9P[8]	98,5
6	KJ753428-RVA/Human-wt/UGA/MRC-DPRU4595/2011/G9P[8]	98,5
7	KX632324-RVA/Human-wt/UGA/MUL-13-285/2013/G9P[8]	98,4
8	KF636293-RVA/Human-wt/CMR/MRC-DPRU1417/2009/G9P[8]	98,3
9	JF490090-RVA/human/CAN/CK00001/2004/G1P[8]	98,3
10	KX632291-RVA/Human-wt/UGA/MUL-13-163/2013/G9P[8]	98,3
11	KJ627120-RVA/Human-wt/PRY/371/1999/G1P[8]	98,3
12	KC769364-RVA/Human-wt/AUS/CK00064/2007/G1P[8]	98,2
13	JF490200-RVA/human/CAN/CK00012/2004/G1P[8]	98,2
14	HQ392316-RVA/Human-wt/BEL/BE00036/2008/G1P[8]	98,2
15	KJ752940-RVA/Human-wt/ZAF/MRC-DPRU2711/2008/G9P[8]	98,1
16	KX655532-RVA/Cow-wt/UGA/BUW-14-A035/2014/G12P[8]	98,1
17	KX655532-RVA/Cow-wt/UGA/BUW-14-A035/2014/G12P[8]	98,1
18	KU048615-RVA/Human-wt/ITA/PA93/12/2012/G12P[8]	98,1
19	HQ392237-RVA/Human-wt/BEL/BE00029/2008/G1P[8]	98,1
20	KJ753372-RVA/Human-wt/ZAF/MRC-DPRU2943/2008/G9P[8]	98,0
21	MH171381-RVA/Human-wt/ESP/VI4816626/2012/G12P[8]	98,0
22	KJ752611-RVA/Human-wt/TGO/MRC-DPRU2229/XXXX/G9P[8]	98,0
23	MF168284-RVA/Human-wt/USA/VU12-13-157/2013/G12P8[3]	97,9
24	KP752870-RVA/Human-wt/ZMB/MRC-DPRU1660/2008/G12P[6	97,9
25	KM116053-RVA/Human-wt/USA/2013774166/2013/G12P[8]	97,9
26	KM116042-RVA/Human-wt/USA/2013774165/2013/G12P[8]	97,8
27	JQ818154-RVA/Human/RUS/Omsk08-425/2008/G1P[8]	95,3
28	KC890877-RVA/Human-wt/GR/Ath113/2009/G4P[8]	95,2
29	JQ585598-RVA/Human/RUS/Nov06-1255/2006/G4P[8]	95,2
30	JQ230093-RVA/Human-wt/RUS/Nov10-N552/2010/G3P[8]	95,1
31	JQ585600-RVA/Human/RUS/Nov07-2058/2007/G1P[8]	95,1
32	HQ392205-RVA/Human-wt/BEL/BE00025/2007/G1P[8]	95,1
33	**RVA/Human-wt/RWA/UFS-NGS:MRC-DPRU9995/2015/G12P[8**	**95,0**
34	KP119547-RVA/Human-wt/ITA/PG55/2013/G4P[8]	95,0
35	KJ752862-RVA/Human-wt/ZWE/MRC-DPRU1850/2011/G4P[8]	95,0
36	JQ713098-RVA/Human/RUS/Nov07-1971/2007/G1P[8]	94,9
37	KC713889-RVA/Human/RUS/Nov12-N4252/2012/G4P[8]	94,9
38	MH473471-RVA/Human-wt/RUS/Nov12-N3593/2012/GXP[8]	94,9
39	KP119544-RVA/Human-wt/ITA/BZ52/2013/G4P[8]	94,9
40	MH484058-RVA/Hu-wt/RUS/S13-118/2013/G4P[8]	94,8
41	JX867352-RVA/Human/RUS/O903/2010/GXP[8]	94,5
42	KJ162413-RVA/Human-wt/RUS/NN934-12/2012/G4P[8]	94,4
43	**RVA/Human-wt/RWA/UFS-NGS:MRC-DPRU8020/2015/G12P[8**	**94,3**
44	**RVA/Human-wt/RWA/UFS-NGS:MRC-DPRU566/2013/G9P[4]**	**93,4**
45	JX943613-RVA/Human-TC/USA/Rotarix/2009/G1P[8]	88,2
46	**RVA/Human-wt/RWA/UFS-NGS:MRC-DPRU6235/2014/G4P[4]**	**87,9**
47	KU861383-RVA/Human-tc/USA/Wa/1974/G1P[8]	87,9
48	DQ870507-RVA/Human-tc/USA/DS-1/1976/G2P[4]	73,4
49	GU565045-RVA/Vaccine/USA/RotaTeq-WI79-4/1992/G6P1[8]	72,3
50	GU565089-RVA/Vaccine/USA/RotaTeq-BrB-9/1996/G4P7[5]	72,1
51	GU565067-RVA/Vaccine/USA/RotaTeq-SC2-9/1992/G2P7[5]	72,1
52	GU565056-RVA/Vaccine/USA/RotaTeq-WI79-9/1992/G1P7[5]	72,1
53	KJ919810-RVA/Human-wt/HUN/ERN5635/2012/G2P[4]	72,1
54	GU565078-RVA/Vaccine/USA/RotaTeq-WI78-8/1992/G3P7[5]	72,0
55	KM660383-RVA/Human-wt/CMR/BA368/2010/G2P[4]	70,8
56	DQ490538-RVA/Human-tc/JPN/AU-1/1982/G3P3[9]	70,5
57	KC834699-RVA/Human-wt/AUS/V203/2009/G2P[4]	70,3
58	KC834700-RVA/Human-wt/AUS/336190/2004/G2P[4]	70,3
59	JQ069584-RVA/Human-wt/CAN/RT125-07/2008/G2P[4]	69,9

B. VP1

	**RVA/Human-wt/RWA/UFS-NGS:MRC-DPRU8020/2015/G12P[8]**	
1	LC384330-RVA/Human-wt/JPN/HKD0825/2016/G1P[8]	99,9
2	GU565041-RVA/Vaccine/USA/RotaTeq-WI79-4/1992/G6P1[8]	99,9
3	KT919447-RVA/Human-wt/USA/VU12-13-176/2013/G1G6P[8]	99,9
4	GU565085-RVA/Vaccine/USA/RotaTeq-BrB-9/1996/G4P7[5]	99,9
5	**RVA/Human-wt/RWA/UFS-NGS:MRC-DPRU9995/2015/G12P[8]**	**99,9**
6	GU565052-RVA/Vaccine/USA/RotaTeq-WI79-9/1992/G1P7[5]	99,9
7	KC443598-RVA/Human-wt/AUS/CK20039/2008/G1P[8]	99,9
8	EF560615-RVA/Cow-tc/USA/WC3/1981/G6P[5]	99,8
9	AB748573-RVA/Cow-tc/JPN/BRV105/1983/G6P[1]	96,9
10	EF554093-RVA/Human-tc/AUS/MG6/1993/G6P[14]	96,9
11	AB853890-RVA/Cow-wt/JPN/Tottori-SG/2013/G15P[14]	96,8
12	AB748576-RVA/Human-tc/ISR/Ro8059/1995/G6P[1]	96,7
13	LC095957-RVA/Human-wt/VNM/NT0082/2007/G10P[14]	96,7
14	MF940641-RVA/Bovine-tc/KOR/KJ19-2/2006/G6P[7]	96,6
15	LC133547-RVA/Cow-tc/USA/B223/1983/G10P[11]	96,6
16	FJ422131-RVA/Rhesus-tc/USA/PTRV/1990/G8[1]	96,5
17	EF583006-RVA/Simian-tc/USA/RRV/1975/G3P[3]	96,5
18	KP006506-RVA/Human-wt/GTM/2009726790/2009/G8P[14]	96,4
19	KU956006-RVA/Human/HON/2011825363/2011/G10P[14]	96,3
20	KF541281-RVA/Human-wt/US/09US7118/2009/G3P[24]	96,3
21	KR705300-RVA/Human-wt/BEL/BE41/2007/G2P[4]	96,0
22	LC133536-RVA/Cow-tc/THA/61A/1989/G10P[5]	95,8
23	JF693026-bovine-tc/USA/NCDV/1971/G6P[1]	95,7
24	KU708259-RVA/Deer-wt/SLO/D38-14/2014/G6P[15]	95,6
25	LC336591-RVA/Human-wt/JPN/12-1375/2012/G8P[14]	95,6
26	KJ919388-RVA/Human-wt/HUN/ERN5635/2012/G2P[4]	95,5
27	LC133558-RVA/Cow-tc/JPN/KK3/1983/G10P[11]	95,2
28	**RVA/Human-wt/RWA/UFS-NGS:MRC-DPRU566/2013/G9P[4]**	**94,1**
29	JQ069919-RVA/Human-wt/CAN/RT125-07/2008/G2P[4]	85,0
30	KC834712-RVA/Human-wt/AUS/336190/2004/G2P[4]	84,9
31	KC834711-RVA/Human-wt/AUS/V203/2009/G2P[4]	84,7
32	KC443631-RVA/Human-wt/AUS/CK20028/2006/G2P[4]	84,6
33	KM660259-RVA/Human-wt/CMR/BA368/2010/G2P[4]	84,5
34	LC172978-RVA/Human/JPN/SP030/2013/G1P[8]	84,5
35	MF044131-RVA/Human-wt/TWN/2014/103-701-D166/G1P[8]	84,4
36	KX362645-RVA/Human-wt/VNM/16020_44/G4P[6]	84,3
37	LC169973-RVA/Human-wt/THA/LS-202/2014/G2P[4]	84,3
38	KX362744-RVA/Human-wt/VNM/16020_81/G2P[4]	84,3
39	LC169984-RVA/Human-wt/THA/LS-L7/2014/G2P[4]	84,2
40	DQ870505-RVA/Human-tc/USA/DS-1/1976/G2P[4]	84,2
41	**RVA/Human-wt/RWA/UFS-NGS:MRC-DPRU6235/2014/G4P[4]**	**82,5**
42	DQ490533-RVA/Human-tc/JPN/AU-1/1982/G3P3[9]	75,2
43	KF371688-RVA/Human-tc/CHN/R709/2005/G3P[8]	73,0
44	KJ753147-RVA/Human-wt/ZAF/MRC-DPRU840/2006/G1P[8]	73,0
45	LC368099-RVA/Human-wt/NPL/5N0109/2005/G12P[6]	73,0
46	LC368088-RVA/Human-wt/NPL/5N0014/2005/G12P[6]	72,9
47	KJ627112-RVA/Human-wt/PRY/371/1999/G1P[8]	72,8
48	KJ752469-RVA/Human-wt/ZAF/MRC-DPRU1911/2007/G12P[6]	72,8
49	KJ753158-RVA/Human-wt/ZAF/MRC-DPRU2132/2005/G1P[8]	72,7
50	**RVA/Human-wt/RWA/UFS-NGS:MRC-DPRU6212/2014/G12P[8]**	**72,7**
51	JN258830-RVA/Human-wt/Bel/BE00073/2001/G1P[8]	72,7
52	KC193633-RVA/Human-wt/Bel/BE00092/2003/G1P[8]	72,6
53	KJ559249-RVA/Human-wt/ARG/SF931/1998/G4P[8]	72,6
54	KJ559029-RVA/Human-wt/ARG/Tuc1650/1998/G4P[8]	72,5
55	JX943609-RVA/Human-tc/USA/Rotarix/2009/G1P[8]	72,5
56	DQ490539-RVA/Human-tc/USA/Wa/1974/G1P[8]	72,5

	**RVA/Human-wt/RWA/UFS-NGS:MRC-DPRU6235/2014/G4P[4]**	
1	KX362645-RVA/Human-wt/VNM/16020_44/G4P[6]	94,9
2	KC443631-RVA/Human-wt/AUS/CK20028/2006/G2P[4]	94,8
3	LC169973-RVA/Human-wt/THA/LS-202/2014/G2P[4]	94,8
4	LC169984-RVA/Human-wt/THA/LS-L7/2014/G2P[4]	94,8
5	MF044131-RVA/Human-wt/TWN/2014/103-701-D166/G1P[8]	94,8
6	KX362744-RVA/Human-wt/VNM/16020_81/G2P[4]	94,8
7	LC172978-RVA/Human/JPN/SP030/2013/G1P[8]	94,7
8	KC834711-RVA/Human-wt/AUS/V203/2009/G2P[4]	93,3
9	KC834712-RVA/Human-wt/AUS/336190/2004/G2P[4]	93,1
10	JQ069919-RVA/Human-wt/CAN/RT125-07/2008/G2P[4]	93,0
11	KM660259-RVA/Human-wt/CMR/BA368/2010/G2P[4]	92,8
12	DQ870505-RVA/Human-tc/USA/DS-1/1976/G2P[4]	86,4
13	LC095957-RVA/Human-wt/VNM/NT0082/2007/G10P[14]	83,0
14	KP006506-RVA/Human-wt/GTM/2009726790/2009/G8P[14]	82,7
15	JF693026-bovine-tc/USA/NCDV/1971/G6P[1]	82,7
16	LC133558-RVA/Cow-tc/JPN/KK3/1983/G10P[11]	82,7
17	FJ422131-RVA/Rhesus-tc/USA/PTRV/1990/G8P[1]	82,7
18	KR705300-RVA/Human-wt/BEL/BE41/2007/G2P[4]	82,7
19	KU708259-RVA/Deer-wt/SLO/D38-14/2014/G6P[15]	82,7
20	KU956006-RVA/Human/HON/2011825363/2011/G10P[14]	82,7
21	AB853890-RVA/Cow-wt/JPN/Tottori-SG/2013/G15P[14]	82,7
22	LC133536-RVA/Cow-tc/THA/61A/1989/G10P[5]	82,6
23	KJ919388-RVA/Human-wt/HUN/ERN5635/2012/G2P[4]	82,6
24	LC384330-RVA/Human-wt/JPN/HKD0825/2016/G1P[8]	82,6
25	GU565041-RVA/Vaccine/USA/RotaTeq-WI79-4/1992/G6P1[8]	82,6
26	KT919447-RVA/Human-wt/USA/VU12-13-176/2013/G1G6P[8]	82,6
27	EF560615-RVA/Cow-tc/USA/WC3/1981/G6P[5]	82,6
28	EF583006-RVA/Simian-tc/USA/RRV/1975/G3P[3]	82,6
29	KF541281-RVA/Human-wt/US/09US7118/2009/G3P[24]	82,6
30	GU565085-RVA/Vaccine/USA/RotaTeq-BrB-9/1996/G4P7[5]	82,6
31	**RVA/Human-wt/RWA/UFS-NGS:MRC-DPRU9995/2015/G12P[8]**	**82,6**
32	GU565052-RVA/Vaccine/USA/RotaTeq-WI79-9/1992/G1P7[5]	82,5
33	KC443598-RVA/Human-wt/AUS/CK20039/2008/G1P[8]	82,5
34	**RVA/Human-wt/RWA/UFS-NGS:MRC-DPRU8020/2015/G12P[8]**	**82,5**
35	LC336591-RVA/Human-wt/JPN/12-1375/2012/G8P[14]	82,5
36	LC133547-RVA/Cow-tc/USA/B223/1983/G10P[11]	82,5
37	EF554093-RVA/Human-tc/AUS/MG6/1993/G6P[14]	82,5
38	MF940641-RVA/Bovine-tc/KOR/KJ19-2/2006/G6P[7]	82,4
39	AB748573-RVA/Cow-tc/JPN/BRV105/1983/G6P[1]	82,3
40	AB748576-RVA/Human-tc/ISR/Ro8059/1995/G6P[1]	82,1
41	**RVA/Human-wt/RWA/UFS-NGS:MRC-DPRU566/2013/G9P[4]**	**81,5**
42	DQ490539-RVA/Human-tc/USA/Wa/1974/G1P[8]	80,8
43	JX943609-RVA/Human-tc/USA/Rotarix/2009/G1P[8]	80,7
44	KJ753147-RVA/Human-wt/ZAF/MRC-DPRU840/2006/G1P[8]	80,1
45	LC368088-RVA/Human-wt/NPL/5N0014/2005/G12P[6]	80,1
46	KJ753158-RVA/Human-wt/ZAF/MRC-DPRU2132/2005/G1P[8]	80,0
47	LC368099-RVA/Human-wt/NPL/5N0109/2005/G12P[6]	80,0
48	KJ752469-RVA/Human-wt/ZAF/MRC-DPRU1911/2007/G12P[6]	80,0
49	**RVA/Human-wt/RWA/UFS-NGS:MRC-DPRU6212/2014/G12P[8]**	**79,9**
50	KC193633-RVA/Human-wt/Bel/BE00092/2003/G1P[8]	79,8
51	KJ627112-RVA/Human-wt/PRY/371/1999/G1P[8]	79,8
52	JN258830-RVA/Human-wt/Bel/BE00073/2001/G1P[8]	79,7
53	KJ559249-RVA/Human-wt/ARG/SF931/1998/G4P[8]	79,7
54	KJ559029-RVA/Human-wt/ARG/Tuc1650/1998/G4P[8]	79,7
55	KF371688-RVA/Human-tc/CHN/R709/2005/G3P[8]	79,6
56	DQ490533-RVA/Human-tc/JPN/AU-1/1982/G3P3[9]	75,8

	**RVA/Human-wt/RWA/UFS-NGS:MRC-DPRU566/2013/G9P[4]**	
1	GU565085-RVA/Vaccine/USA/RotaTeq-BrB-9/1996/G4P7[5]	94,2
2	GU565052-RVA/Vaccine/USA/RotaTeq-WI79-9/1992/G1P7[5]	94,2
3	LC384330-RVA/Human-wt/JPN/HKD0825/2016/G1P[8]	94,2
4	GU565041-RVA/Vaccine/USA/RotaTeq-WI79-4/1992/G6P1[8]	94,2
5	KT919447-RVA/Human-wt/USA/VU12-13-176/2013/G1G6P[8]	94,2
6	**RVA/Human-wt/RWA/UFS-NGS:MRC-DPRU9995/2015/G12P[8]**	**94,1**
7	KC443598-RVA/Human-wt/AUS/CK20039/2008/G1P[8]	94,1
8	EF560615-RVA/Cow-tc/USA/WC3/1981/G6P[5]	94,1
9	**RVA/Human-wt/RWA/UFS-NGS:MRC-DPRU8020/2015/G12P[8]**	**94,1**
10	AB853890-RVA/Cow-wt/JPN/Tottori-SG/2013/G15P[14]	91,8
11	EF554093-RVA/Human-tc/AUS/MG6/1993/G6P[14]	91,6
12	MF940641-RVA/Bovine-tc/KOR/KJ19-2/2006/G6P[7]	91,5
13	AB748573-RVA/Cow-tc/JPN/BRV105/1983/G6P[1]	91,5
14	FJ422131-RVA/Rhesus-tc/USA/PTRV/1990/G8P[1]	91,5
15	LC095957-RVA/Human-wt/VNM/NT0082/2007/G10P[14]	91,5
16	KP006506-RVA/Human-wt/GTM/2009726790/2009/G8P[14]	91,5
17	EF583006-RVA/Simian-tc/USA/RRV/1975/G3P[3]	91,3
18	AB748576-RVA/Human-tc/ISR/Ro8059/1995/G6P[1]	91,3
19	LC133547-RVA/Cow-tc/USA/B223/1983/G10P[11]	91,3
20	KF541281-RVA/Human-wt/US/09US7118/2009/G3P[24]	91,2
21	KU956006-RVA/Human/HON/2011825363/2011/G10P[14]	91,0
22	LC133536-RVA/Cow-tc/THA/61A/1989/G10P[5]	90,9
23	KU708259-RVA/Deer-wt/SLO/D38-14/2014/G6P[15]	90,8
24	KR705300-RVA/Human-wt/BEL/BE41/2007/G2P[4]	90,7
25	LC336591-RVA/Human-wt/JPN/12-1375/2012/G8P[14]	90,6
26	JF693026-bovine-tc/USA/NCDV/1971/G6P[1]	90,6
27	KJ919388-RVA/Human-wt/HUN/ERN5635/2012/G2P[4]	90,3
28	LC133558-RVA/Cow-tc/JPN/KK3/1983/G10P[11]	90,2
29	JQ069919-RVA/Human-wt/CAN/RT125-07/2008/G2P[4]	82,9
30	KC834711-RVA/Human-wt/AUS/V203/2009/G2P[4]	82,7
31	LC172978-RVA/Human/JPN/SP030/2013/G1P[8]	82,6
32	KM660259-RVA/Human-wt/CMR/BA368/2010/G2P[4]	82,5
33	KC834712-RVA/Human-wt/AUS/336190/2004/G2P[4]	82,5
34	KC443631-RVA/Human-wt/AUS/CK20028/2006/G2P[4]	82,5
35	MF044131-RVA/Human-wt/TWN/2014/103-701-D166/G1P[8]	82,4
36	KX362645-RVA/Human-wt/VNM/16020_44/G4P[6]	82,2
37	LC169973-RVA/Human-wt/THA/LS-202/2014/G2P[4]	82,2
38	LC169984-RVA/Human-wt/THA/LS-L7/2014/G2P[4]	82,2
39	KX362744-RVA/Human-wt/VNM/16020_81/G2P[4]	82,1
40	DQ870505-RVA/Human-tc/USA/DS-1/1976/G2P[4]	81,5
41	**RVA/Human-wt/RWA/UFS-NGS:MRC-DPRU6235/2014/G4P[4]**	**81,5**
42	KF371688-RVA/Human-tc/CHN/R709/2005/G3P[8]	80,7
43	JN258830-RVA/Human-wt/Bel/BE00073/2001/G1P[8]	80,7
44	KJ753158-RVA/Human-wt/ZAF/MRC-DPRU2132/2005/G1P[8]	80,6
45	KJ627112-RVA/Human-wt/PRY/371/1999/G1P[8]	80,6
46	KJ559249-RVA/Human-wt/ARG/SF931/1998/G4P[8]	80,6
47	LC368099-RVA/Human-wt/NPL/5N0109/2005/G12P[6]	80,6
48	KJ753147-RVA/Human-wt/ZAF/MRC-DPRU840/2006/G1P[8]	80,5
49	LC368088-RVA/Human-wt/NPL/5N0014/2005/G12P[6]	80,5
50	KJ559029-RVA/Human-wt/ARG/Tuc1650/1998/G4P[8]	80,5
51	KC193633-RVA/Human-wt/Bel/BE00092/2003/G1P[8]	80,5
52	KJ752469-RVA/Human-wt/ZAF/MRC-DPRU1911/2007/G12P[6]	80,4
53	**RVA/Human-wt/RWA/UFS-NGS:MRC-DPRU6212/2014/G12P[8]**	**80,2**
54	DQ490539-RVA/Human-tc/USA/Wa/1974/G1P[8]	79,9
55	JX943609-RVA/Human-tc/USA/Rotarix/2009/G1P[8]	79,8
56	DQ490533-RVA/Human-tc/JPN/AU-1/1982/G3P3[9]	74,9

	**RVA/Human-wt/RWA/UFS-NGS:MRC-DPRU9995/2015/G12P[8]**	
1	LC384330-RVA/Human-wt/JPN/HKD0825/2016/G1P[8]	100,0
2	GU565041-RVA/Vaccine/USA/RotaTeq-WI79-4/1992/G6P1[8]	100,0
3	KT919447-RVA/Human-wt/USA/VU12-13-176/2013/G1G6P[8]	100,0
4	GU565085-RVA/Vaccine/USA/RotaTeq-BrB-9/1996/G4P7[5]	99,9
5	GU565052-RVA/Vaccine/USA/RotaTeq-WI79-9/1992/G1P7[5]	99,9
6	KC443598-RVA/Human-wt/AUS/CK20039/2008/G1P[8]	99,9
7	**RVA/Human-wt/RWA/UFS-NGS:MRC-DPRU8020/2015/G12P[8]**	**99,9**
8	EF560615-RVA/Cow-tc/USA/WC3/1981/G6P[5]	99,8
9	AB748573-RVA/Cow-tc/JPN/BRV105/1983/G6P[1]	96,9
10	EF554093-RVA/Human-tc/AUS/MG6/1993/G6P[14]	96,9
11	AB853890-RVA/Cow-wt/JPN/Tottori-SG/2013/G15P[14]	96,9
12	LC095957-RVA/Human-wt/VNM/NT0082/2007/G10P[14]	96,8
13	AB748576-RVA/Human-tc/ISR/Ro8059/1995/G6P[1]	96,7
14	MF940641-RVA/Bovine-tc/KOR/KJ19-2/2006/G6P[7]	96,7
15	LC133547-RVA/Cow-tc/USA/B223/1983/G10P[11]	96,6
16	FJ422131-RVA/Rhesus-tc/USA/PTRV/1990/G8P[1]	96,6
17	EF583006-RVA/Simian-tc/USA/RRV/1975/G3P[3]	96,6
18	KP006506-RVA/Human-wt/GTM/2009726790/2009/G8P[14]	96,4
19	KU956006-RVA/Human/HON/2011825363/2011/G10P[14]	96,3
20	KF541281-RVA/Human-wt/US/09US7118/2009/G3P[24]	96,3
21	KR705300-RVA/Human-wt/BEL/BE41/2007/G2P[4]	96,0
22	LC133536-RVA/Cow-tc/THA/61A/1989/G10P[5]	95,8
23	JF693026-bovine-tc/USA/NCDV/1971/G6P[1]	95,8
24	KU708259-RVA/Deer-wt/SLO/D38-14/2014/G6P[15]	95,7
25	LC336591-RVA/Human-wt/JPN/12-1375/2012/G8P[14]	95,6
26	KJ919388-RVA/Human-wt/HUN/ERN5635/2012/G2P[4]	95,6
27	LC133558-RVA/Cow-tc/JPN/KK3/1983/G10P[11]	95,2
28	**RVA/Human-wt/RWA/UFS-NGS:MRC-DPRU566/2013/G9P[4]**	**94,1**
29	JQ069919-RVA/Human-wt/CAN/RT125-07/2008/G2P[4]	85,1
30	KC834712-RVA/Human-wt/AUS/336190/2004/G2P[4]	84,9
31	KC834711-RVA/Human-wt/AUS/V203/2009/G2P[4]	84,7
32	KC443631-RVA/Human-wt/AUS/CK20028/2006/G2P[4]	84,6
33	KM660259-RVA/Human-wt/CMR/BA368/2010/G2P[4]	84,6
34	LC172978-RVA/Human/JPN/SP030/2013/G1P[8]	84,5
35	MF044131-RVA/Human-wt/TWN/2014/103-701-D166/G1P[8]	84,4
36	KX362645-RVA/Human-wt/VNM/16020_44/G4P[6]	84,3
37	LC169973-RVA/Human-wt/THA/LS-202/2014/G2P[4]	84,3
38	KX362744-RVA/Human-wt/VNM/16020_81/G2P[4]	84,3
39	LC169984-RVA/Human-wt/THA/LS-L7/2014/G2P[4]	84,3
40	DQ870505-RVA/Human-tc/USA/DS-1/1976/G2P[4]	84,2
41	**RVA/Human-wt/RWA/UFS-NGS:MRC-DPRU6235/2014/G4P[4]**	**82,6**
42	DQ490533-RVA/Human-tc/JPN/AU-1/1982/G3P3[9]	75,2
43	KF371688-RVA/Human-tc/CHN/R709/2005/G3P[8]	73,0
44	LC368099-RVA/Human-wt/NPL/5N0109/2005/G12P[6]	73,0
45	KJ753147-RVA/Human-wt/ZAF/MRC-DPRU840/2006/G1P[8]	73,0
46	LC368088-RVA/Human-wt/NPL/5N0014/2005/G12P[6]	72,9
47	KJ627112-RVA/Human-wt/PRY/371/1999/G1P[8]	72,8
48	KJ752469-RVA/Human-wt/ZAF/MRC-DPRU1911/2007/G12P[6]	72,8
49	KJ753158-RVA/Human-wt/ZAF/MRC-DPRU2132/2005/G1P[8]	72,7
50	**RVA/Human-wt/RWA/UFS-NGS:MRC-DPRU6212/2014/G12P[8]**	**72,7**
51	JN258830-RVA/Human-wt/Bel/BE00073/2001/G1P[8]	72,7
52	KC193633-RVA/Human-wt/Bel/BE00092/2003/G1P[8]	72,6
53	KJ559249-RVA/Human-wt/ARG/SF931/1998/G4P[8]	72,6
54	JX943609-RVA/Human-tc/USA/Rotarix/2009/G1P[8]	72,6
55	KJ559029-RVA/Human-wt/ARG/Tuc1650/1998/G4P[8]	72,5
56	DQ490539-RVA/Human-tc/USA/Wa/1974/G1P[8]	72,5

	**RVA/Human-wt/RWA/UFS-NGS:MRC-DPRU6212/2014/G12P[8]**	
1	LC368088-RVA/Human-wt/NPL/5N0014/2005/G12P[6]	99,0
2	LC368099-RVA/Human-wt/NPL/5N0109/2005/G12P[6]	98,9
3	KJ753147-RVA/Human-wt/ZAF/MRC-DPRU840/2006/G1P[8]	98,9
4	KJ752469-RVA/Human-wt/ZAF/MRC-DPRU1911/2007/G12P[6]	98,8
5	KC193633-RVA/Human-wt/Bel/BE00092/2003/G1P[8]	98,6
6	KJ753158-RVA/Human-wt/ZAF/MRC-DPRU2132/2005/G1P[8]	98,5
7	KJ559249-RVA/Human-wt/ARG/SF931/1998/G4P[8]	97,8
8	KJ559029-RVA/Human-wt/ARG/Tuc1650/1998/G4P[8]	97,8
9	KJ627112-RVA/Human-wt/PRY/371/1999/G1P[8]	97,7
10	JN258830-RVA/Human-wt/Bel/BE00073/2001/G1P[8]	97,6
11	KF371688-RVA/Human-tc/CHN/R709/2005/G3P[8]	97,3
12	DQ490539-RVA/Human-tc/USA/Wa/1974/G1P[8]	94,8
13	JX943609-RVA/Human-tc/USA/Rotarix/2009/G1P[8]	94,3
14	**RVA/Human-wt/RWA/UFS-NGS:MRC-DPRU566/2013/G9P[4]**	**80,2**
15	**RVA/Human-wt/RWA/UFS-NGS:MRC-DPRU6235/2014/G4P[4]**	**79,9**
16	LC172978-RVA/Human/JPN/SP030/2013/G1P[8]	75,2
17	MF044131-RVA/Human-wt/TWN/2014/103-701-D166/G1P[8]	75,1
18	DQ490533-RVA/Human-tc/JPN/AU-1/1982/G3P3[9]	74,9
19	KC834711-RVA/Human-wt/AUS/V203/2009/G2P[4]	74,9
20	KX362645-RVA/Human-wt/VNM/16020_44/G4P[6]	74,8
21	KM660259-RVA/Human-wt/CMR/BA368/2010/G2P[4]	74,7
22	KX362744-RVA/Human-wt/VNM/16020_81/G2P[4]	74,7
23	LC169973-RVA/Human-wt/THA/LS-202/2014/G2P[4]	74,7
24	LC169984-RVA/Human-wt/THA/LS-L7/2014/G2P[4]	74,7
25	KC443631-RVA/Human-wt/AUS/CK20028/2006/G2P[4]	74,6
26	KC834712-RVA/Human-wt/AUS/336190/2004/G2P[4]	74,6
27	JQ069919-RVA/Human-wt/CAN/RT125-07/2008/G2P[4]	74,5
28	DQ870505-RVA/Human-tc/USA/DS-1/1976/G2P[4]	73,5
29	KP006506-RVA/Human-wt/GTM/2009726790/2009/G8P[14]	73,2
30	AB853890-RVA/Cow-wt/JPN/Tottori-SG/2013/G15P[14]	73,1
31	KU708259-RVA/Deer-wt/SLO/D38-14/2014/G6P[15]	73,0
32	FJ422131-RVA/Rhesus-tc/USA/PTRV/1990/G8P[1]	73,0
33	EF554093-RVA/Human-tc/AUS/MG6/1993/G6P[14]	72,9
34	KU956006-RVA/Human/HON/2011825363/2011/G10P[14]	72,9
35	LC095957-RVA/Human-wt/VNM/NT0082/2007/G10P[14]	72,8
36	JF693026-bovine-tc/USA/NCDV/1971/G6P[1]	72,8
37	LC133536-RVA/Cow-tc/THA/61A/1989/G10P[5]	72,8
38	EF583006-RVA/Simian-tc/USA/RRV/1975/G3P[3]	72,8
39	LC336591-RVA/Human-wt/JPN/12-1375/2012/G8P[14]	72,8
40	LC384330-RVA/Human-wt/JPN/HKD0825/2016/G1P[8]	72,8
41	GU565041-RVA/Vaccine/USA/RotaTeq-WI79-4/1992/G6P1[8]	72,8
42	KT919447-RVA/Human-wt/USA/VU12-13-176/2013/G1G6P[8]	72,8
43	KJ919388-RVA/Human-wt/HUN/ERN5635/2012/G2P[4]	72,7
44	LC133558-RVA/Cow-tc/JPN/KK3/1983/G10P[11]	72,7
45	GU565085-RVA/Vaccine/USA/RotaTeq-BrB-9/1996/G4P7[5]	72,7
46	**RVA/Human-wt/RWA/UFS-NGS:MRC-DPRU9995/2015/G12P[8]**	**72,7**
47	**RVA/Human-wt/RWA/UFS-NGS:MRC-DPRU8020/2015/G12P[8]**	**72,7**
48	EF560615-RVA/Cow-tc/USA/WC3/1981/G6P[5]	72,7
49	GU565052-RVA/Vaccine/USA/RotaTeq-WI79-9/1992/G1P7[5]	72,7
50	KC443598-RVA/Human-wt/AUS/CK20039/2008/G1P[8]	72,7
51	KR705300-RVA/Human-wt/BEL/BE41/2007/G2P[4]	72,6
52	MF940641-RVA/Bovine-tc/KOR/KJ19-2/2006/G6P[7]	72,5
53	LC133547-RVA/Cow-tc/USA/B223/1983/G10P[11]	72,4
54	AB748573-RVA/Cow-tc/JPN/BRV105/1983/G6P[1]	72,4
55	AB748576-RVA/Human-tc/ISR/Ro8059/1995/G6P[1]	72,1
56	KF541281-RVA/Human-wt/US/09US7118/2009/G3P[24]	71,9

C. VP2

	**RVA/Human-wt/RWA/UFS-NGS:MRC-DPRU8020/2015/G12P[8]**	
1	LC384330-RVA/Human-wt/JPN/HKD0825/2016/G1P[8]	99,9
2	GU565041-RVA/Vaccine/USA/RotaTeq-WI79-4/1992/G6P1[8]	99,9
3	KT919447-RVA/Human-wt/USA/VU12-13-176/2013/G1G6P[8]	99,9
4	GU565085-RVA/Vaccine/USA/RotaTeq-BrB-9/1996/G4P7[5]	99,9
5	**RVA/Human-wt/RWA/UFS-NGS:MRC-DPRU9995/2015/G12P[8]**	**99,9**
6	GU565052-RVA/Vaccine/USA/RotaTeq-WI79-9/1992/G1P7[5]	99,9
7	KC443598-RVA/Human-wt/AUS/CK20039/2008/G1P[8]	99,9
8	EF560615-RVA/Cow-tc/USA/WC3/1981/G6P[5]	99,8
9	AB748573-RVA/Cow-tc/JPN/BRV105/1983/G6P[1]	96,9
10	EF554093-RVA/Human-tc/AUS/MG6/1993/G6P[14]	96,9
11	AB853890-RVA/Cow-wt/JPN/Tottori-SG/2013/G15P[14]	96,8
12	AB748576-RVA/Human-tc/ISR/Ro8059/1995/G6P[1]	96,7
13	LC095957-RVA/Human-wt/VNM/NT0082/2007/G10P[14]	96,7
14	MF940641-RVA/Bovine-tc/KOR/KJ19-2/2006/G6P[7]	96,6
15	LC133547-RVA/Cow-tc/USA/B223/1983/G10P[11]	96,6
16	FJ422131-RVA/Rhesus-tc/USA/PTRV/1990/G8[1]	96,5
17	EF583006-RVA/Simian-tc/USA/RRV/1975/G3P[3]	96,5
18	KP006506-RVA/Human-wt/GTM/2009726790/2009/G8P[14]	96,4
19	KU956006-RVA/Human/HON/2011825363/2011/G10P[14]	96,3
20	KF541281-RVA/Human-wt/US/09US7118/2009/G3P[24]	96,3
21	KR705300-RVA/Human-wt/BEL/BE41/2007/G2P[4]	96,0
22	LC133536-RVA/Cow-tc/THA/61A/1989/G10P[5]	95,8
23	JF693026-bovine-tc/USA/NCDV/1971/G6P[1]	95,7
24	KU708259-RVA/Deer-wt/SLO/D38-14/2014/G6P[15]	95,6
25	LC336591-RVA/Human-wt/JPN/12-1375/2012/G8P[14]	95,6
26	KJ919388-RVA/Human-wt/HUN/ERN5635/2012/G2P[4]	95,5
27	LC133558-RVA/Cow-tc/JPN/KK3/1983/G10P[11]	95,2
28	**RVA/Human-wt/RWA/UFS-NGS:MRC-DPRU566/2013/G9P[4]**	**94,1**
29	JQ069919-RVA/Human-wt/CAN/RT125-07/2008/G2P[4]	85,0
30	KC834712-RVA/Human-wt/AUS/336190/2004/G2P[4]	84,9
31	KC834711-RVA/Human-wt/AUS/V203/2009/G2P[4]	84,7
32	KC443631-RVA/Human-wt/AUS/CK20028/2006/G2P[4]	84,6
33	KM660259-RVA/Human-wt/CMR/BA368/2010/G2P[4]	84,5
34	LC172978-RVA/Human/JPN/SP030/2013/G1P[8]	84,5
35	MF044131-RVA/Human-wt/TWN/2014/103-701-D166/G1P[8]	84,4
36	KX362645-RVA/Human-wt/VNM/16020_44/G4P[6]	84,3
37	LC169973-RVA/Human-wt/THA/LS-202/2014/G2P[4]	84,3
38	KX362744-RVA/Human-wt/VNM/16020_81/G2P[4]	84,3
39	LC169984-RVA/Human-wt/THA/LS-L7/2014/G2P[4]	84,2
40	DQ870505-RVA/Human-tc/USA/DS-1/1976/G2P[4]	84,2
41	**RVA/Human-wt/RWA/UFS-NGS:MRC-DPRU6235/2014/G4P[4]**	**82,5**
42	DQ490533-RVA/Human-tc/JPN/AU-1/1982/G3P3[9]	75,2
43	KF371688-RVA/Human-tc/CHN/R709/2005/G3P[8]	73,0
44	KJ753147-RVA/Human-wt/ZAF/MRC-DPRU840/2006/G1P[8]	73,0
45	LC368099-RVA/Human-wt/NPL/5N0109/2005/G12P[6]	73,0
46	LC368088-RVA/Human-wt/NPL/5N0014/2005/G12P[6]	72,9
47	KJ627112-RVA/Human-wt/PRY/371/1999/G1P[8]	72,8
48	KJ752469-RVA/Human-wt/ZAF/MRC-DPRU1911/2007/G12P[6]	72,8
49	KJ753158-RVA/Human-wt/ZAF/MRC-DPRU2132/2005/G1P[8]	72,7
50	**RVA/Human-wt/RWA/UFS-NGS:MRC-DPRU6212/2014/G12P[8]**	**72,7**
51	JN258830-RVA/Human-wt/Bel/BE00073/2001/G1P[8]	72,7
52	KC193633-RVA/Human-wt/Bel/BE00092/2003/G1P[8]	72,6
53	KJ559249-RVA/Human-wt/ARG/SF931/1998/G4P[8]	72,6
54	KJ559029-RVA/Human-wt/ARG/Tuc1650/1998/G4P[8]	72,5
55	JX943609-RVA/Human-tc/USA/Rotarix/2009/G1P[8]	72,5
56	DQ490539-RVA/Human-tc/USA/Wa/1974/G1P[8]	72,5

	**RVA/Human-wt/RWA/UFS-NGS:MRC-DPRU6235/2014/G4P[4]**	
1	KX362645-RVA/Human-wt/VNM/16020_44/G4P[6]	94,9
2	KC443631-RVA/Human-wt/AUS/CK20028/2006/G2P[4]	94,8
3	LC169973-RVA/Human-wt/THA/LS-202/2014/G2P[4]	94,8
4	LC169984-RVA/Human-wt/THA/LS-L7/2014/G2P[4]	94,8
5	MF044131-RVA/Human-wt/TWN/2014/103-701-D166/G1P[8]	94,8
6	KX362744-RVA/Human-wt/VNM/16020_81/G2P[4]	94,8
7	LC172978-RVA/Human/JPN/SP030/2013/G1P[8]	94,7
8	KC834711-RVA/Human-wt/AUS/V203/2009/G2P[4]	93,3
9	KC834712-RVA/Human-wt/AUS/336190/2004/G2P[4]	93,1
10	JQ069919-RVA/Human-wt/CAN/RT125-07/2008/G2P[4]	93,0
11	KM660259-RVA/Human-wt/CMR/BA368/2010/G2P[4]	92,8
12	DQ870505-RVA/Human-tc/USA/DS-1/1976/G2P[4]	86,4
13	LC095957-RVA/Human-wt/VNM/NT0082/2007/G10P[14]	83,0
14	KP006506-RVA/Human-wt/GTM/2009726790/2009/G8P[14]	82,7
15	JF693026-bovine-tc/USA/NCDV/1971/G6P[1]	82,7
16	LC133558-RVA/Cow-tc/JPN/KK3/1983/G10P[11]	82,7
17	FJ422131-RVA/Rhesus-tc/USA/PTRV/1990/G8P[1]	82,7
18	KR705300-RVA/Human-wt/BEL/BE41/2007/G2P[4]	82,7
19	KU708259-RVA/Deer-wt/SLO/D38-14/2014/G6P[15]	82,7
20	KU956006-RVA/Human/HON/2011825363/2011/G10P[14]	82,7
21	AB853890-RVA/Cow-wt/JPN/Tottori-SG/2013/G15P[14]	82,7
22	LC133536-RVA/Cow-tc/THA/61A/1989/G10P[5]	82,6
23	KJ919388-RVA/Human-wt/HUN/ERN5635/2012/G2P[4]	82,6
24	LC384330-RVA/Human-wt/JPN/HKD0825/2016/G1P[8]	82,6
25	GU565041-RVA/Vaccine/USA/RotaTeq-WI79-4/1992/G6P1[8]	82,6
26	KT919447-RVA/Human-wt/USA/VU12-13-176/2013/G1G6P[8]	82,6
27	EF560615-RVA/Cow-tc/USA/WC3/1981/G6P[5]	82,6
28	EF583006-RVA/Simian-tc/USA/RRV/1975/G3P[3]	82,6
29	KF541281-RVA/Human-wt/US/09US7118/2009/G3P[24]	82,6
30	GU565085-RVA/Vaccine/USA/RotaTeq-BrB-9/1996/G4P7[5]	82,6
31	**RVA/Human-wt/RWA/UFS-NGS:MRC-DPRU9995/2015/G12P[8]**	**82,6**
32	GU565052-RVA/Vaccine/USA/RotaTeq-WI79-9/1992/G1P7[5]	82,5
33	KC443598-RVA/Human-wt/AUS/CK20039/2008/G1P[8]	82,5
34	**RVA/Human-wt/RWA/UFS-NGS:MRC-DPRU8020/2015/G12P[8]**	**82,5**
35	LC336591-RVA/Human-wt/JPN/12-1375/2012/G8P[14]	82,5
36	LC133547-RVA/Cow-tc/USA/B223/1983/G10P[11]	82,5
37	EF554093-RVA/Human-tc/AUS/MG6/1993/G6P[14]	82,5
38	MF940641-RVA/Bovine-tc/KOR/KJ19-2/2006/G6P[7]	82,4
39	AB748573-RVA/Cow-tc/JPN/BRV105/1983/G6P[1]	82,3
40	AB748576-RVA/Human-tc/ISR/Ro8059/1995/G6P[1]	82,1
41	**RVA/Human-wt/RWA/UFS-NGS:MRC-DPRU566/2013/G9P[4]**	**81,5**
42	DQ490539-RVA/Human-tc/USA/Wa/1974/G1P[8]	80,8
43	JX943609-RVA/Human-tc/USA/Rotarix/2009/G1P[8]	80,7
44	KJ753147-RVA/Human-wt/ZAF/MRC-DPRU840/2006/G1P[8]	80,1
45	LC368088-RVA/Human-wt/NPL/5N0014/2005/G12P[6]	80,1
46	KJ753158-RVA/Human-wt/ZAF/MRC-DPRU2132/2005/G1P[8]	80,0
47	LC368099-RVA/Human-wt/NPL/5N0109/2005/G12P[6]	80,0
48	KJ752469-RVA/Human-wt/ZAF/MRC-DPRU1911/2007/G12P[6]	80,0
49	**RVA/Human-wt/RWA/UFS-NGS:MRC-DPRU6212/2014/G12P[8]**	**79,9**
50	KC193633-RVA/Human-wt/Bel/BE00092/2003/G1P[8]	79,8
51	KJ627112-RVA/Human-wt/PRY/371/1999/G1P[8]	79,8
52	JN258830-RVA/Human-wt/Bel/BE00073/2001/G1P[8]	79,7
53	KJ559249-RVA/Human-wt/ARG/SF931/1998/G4P[8]	79,7
54	KJ559029-RVA/Human-wt/ARG/Tuc1650/1998/G4P[8]	79,7
55	KF371688-RVA/Human-tc/CHN/R709/2005/G3P[8]	79,6
56	DQ490533-RVA/Human-tc/JPN/AU-1/1982/G3P3[9]	75,8

	**RVA/Human-wt/RWA/UFS-NGS:MRC-DPRU566/2013/G9P[4]**	
1	GU565085-RVA/Vaccine/USA/RotaTeq-BrB-9/1996/G4P7[5]	94,2
2	GU565052-RVA/Vaccine/USA/RotaTeq-WI79-9/1992/G1P7[5]	94,2
3	LC384330-RVA/Human-wt/JPN/HKD0825/2016/G1P[8]	94,2
4	GU565041-RVA/Vaccine/USA/RotaTeq-WI79-4/1992/G6P1[8]	94,2
5	KT919447-RVA/Human-wt/USA/VU12-13-176/2013/G1G6P[8]	94,2
6	**RVA/Human-wt/RWA/UFS-NGS:MRC-DPRU9995/2015/G12P[8]**	**94,1**
7	KC443598-RVA/Human-wt/AUS/CK20039/2008/G1P[8]	94,1
8	EF560615-RVA/Cow-tc/USA/WC3/1981/G6P[5]	94,1
9	**RVA/Human-wt/RWA/UFS-NGS:MRC-DPRU8020/2015/G12P[8]**	**94,1**
10	AB853890-RVA/Cow-wt/JPN/Tottori-SG/2013/G15P[14]	91,8
11	EF554093-RVA/Human-tc/AUS/MG6/1993/G6P[14]	91,6
12	MF940641-RVA/Bovine-tc/KOR/KJ19-2/2006/G6P[7]	91,5
13	AB748573-RVA/Cow-tc/JPN/BRV105/1983/G6P[1]	91,5
14	FJ422131-RVA/Rhesus-tc/USA/PTRV/1990/G8P[1]	91,5
15	LC095957-RVA/Human-wt/VNM/NT0082/2007/G10P[14]	91,5
16	KP006506-RVA/Human-wt/GTM/2009726790/2009/G8P[14]	91,5
17	EF583006-RVA/Simian-tc/USA/RRV/1975/G3P[3]	91,3
18	AB748576-RVA/Human-tc/ISR/Ro8059/1995/G6P[1]	91,3
19	LC133547-RVA/Cow-tc/USA/B223/1983/G10P[11]	91,3
20	KF541281-RVA/Human-wt/US/09US7118/2009/G3P[24]	91,2
21	KU956006-RVA/Human/HON/2011825363/2011/G10P[14]	91,0
22	LC133536-RVA/Cow-tc/THA/61A/1989/G10P[5]	90,9
23	KU708259-RVA/Deer-wt/SLO/D38-14/2014/G6P[15]	90,8
24	KR705300-RVA/Human-wt/BEL/BE41/2007/G2P[4]	90,7
25	LC336591-RVA/Human-wt/JPN/12-1375/2012/G8P[14]	90,6
26	JF693026-bovine-tc/USA/NCDV/1971/G6P[1]	90,6
27	KJ919388-RVA/Human-wt/HUN/ERN5635/2012/G2P[4]	90,3
28	LC133558-RVA/Cow-tc/JPN/KK3/1983/G10P[11]	90,2
29	JQ069919-RVA/Human-wt/CAN/RT125-07/2008/G2P[4]	82,9
30	KC834711-RVA/Human-wt/AUS/V203/2009/G2P[4]	82,7
31	LC172978-RVA/Human/JPN/SP030/2013/G1P[8]	82,6
32	KM660259-RVA/Human-wt/CMR/BA368/2010/G2P[4]	82,5
33	KC834712-RVA/Human-wt/AUS/336190/2004/G2P[4]	82,5
34	KC443631-RVA/Human-wt/AUS/CK20028/2006/G2P[4]	82,5
35	MF044131-RVA/Human-wt/TWN/2014/103-701-D166/G1P[8]	82,4
36	KX362645-RVA/Human-wt/VNM/16020_44/G4P[6]	82,2
37	LC169973-RVA/Human-wt/THA/LS-202/2014/G2P[4]	82,2
38	LC169984-RVA/Human-wt/THA/LS-L7/2014/G2P[4]	82,2
39	KX362744-RVA/Human-wt/VNM/16020_81/G2P[4]	82,1
40	DQ870505-RVA/Human-tc/USA/DS-1/1976/G2P[4]	81,5
41	**RVA/Human-wt/RWA/UFS-NGS:MRC-DPRU6235/2014/G4P[4]**	**81,5**
42	KF371688-RVA/Human-tc/CHN/R709/2005/G3P[8]	80,7
43	JN258830-RVA/Human-wt/Bel/BE00073/2001/G1P[8]	80,7
44	KJ753158-RVA/Human-wt/ZAF/MRC-DPRU2132/2005/G1P[8]	80,6
45	KJ627112-RVA/Human-wt/PRY/371/1999/G1P[8]	80,6
46	KJ559249-RVA/Human-wt/ARG/SF931/1998/G4P[8]	80,6
47	LC368099-RVA/Human-wt/NPL/5N0109/2005/G12P[6]	80,6
48	KJ753147-RVA/Human-wt/ZAF/MRC-DPRU840/2006/G1P[8]	80,5
49	LC368088-RVA/Human-wt/NPL/5N0014/2005/G12P[6]	80,5
50	KJ559029-RVA/Human-wt/ARG/Tuc1650/1998/G4P[8]	80,5
51	KC193633-RVA/Human-wt/Bel/BE00092/2003/G1P[8]	80,5
52	KJ752469-RVA/Human-wt/ZAF/MRC-DPRU1911/2007/G12P[6]	80,4
53	**RVA/Human-wt/RWA/UFS-NGS:MRC-DPRU6212/2014/G12P[8]**	**80,2**
54	DQ490539-RVA/Human-tc/USA/Wa/1974/G1P[8]	79,9
55	JX943609-RVA/Human-tc/USA/Rotarix/2009/G1P[8]	79,8
56	DQ490533-RVA/Human-tc/JPN/AU-1/1982/G3P3[9]	74,9

	**RVA/Human-wt/RWA/UFS-NGS:MRC-DPRU9995/2015/G12P[8]**	
1	LC384330-RVA/Human-wt/JPN/HKD0825/2016/G1P[8]	100,0
2	GU565041-RVA/Vaccine/USA/RotaTeq-WI79-4/1992/G6P1[8]	100,0
3	KT919447-RVA/Human-wt/USA/VU12-13-176/2013/G1G6P[8]	100,0
4	GU565085-RVA/Vaccine/USA/RotaTeq-BrB-9/1996/G4P7[5]	99,9
5	GU565052-RVA/Vaccine/USA/RotaTeq-WI79-9/1992/G1P7[5]	99,9
6	KC443598-RVA/Human-wt/AUS/CK20039/2008/G1P[8]	99,9
7	**RVA/Human-wt/RWA/UFS-NGS:MRC-DPRU8020/2015/G12P[8]**	**99,9**
8	EF560615-RVA/Cow-tc/USA/WC3/1981/G6P[5]	99,8
9	AB748573-RVA/Cow-tc/JPN/BRV105/1983/G6P[1]	96,9
10	EF554093-RVA/Human-tc/AUS/MG6/1993/G6P[14]	96,9
11	AB853890-RVA/Cow-wt/JPN/Tottori-SG/2013/G15P[14]	96,9
12	LC095957-RVA/Human-wt/VNM/NT0082/2007/G10P[14]	96,8
13	AB748576-RVA/Human-tc/ISR/Ro8059/1995/G6P[1]	96,7
14	MF940641-RVA/Bovine-tc/KOR/KJ19-2/2006/G6P[7]	96,7
15	LC133547-RVA/Cow-tc/USA/B223/1983/G10P[11]	96,6
16	FJ422131-RVA/Rhesus-tc/USA/PTRV/1990/G8P[1]	96,6
17	EF583006-RVA/Simian-tc/USA/RRV/1975/G3P[3]	96,6
18	KP006506-RVA/Human-wt/GTM/2009726790/2009/G8P[14]	96,4
19	KU956006-RVA/Human/HON/2011825363/2011/G10P[14]	96,3
20	KF541281-RVA/Human-wt/US/09US7118/2009/G3P[24]	96,3
21	KR705300-RVA/Human-wt/BEL/BE41/2007/G2P[4]	96,0
22	LC133536-RVA/Cow-tc/THA/61A/1989/G10P[5]	95,8
23	JF693026-bovine-tc/USA/NCDV/1971/G6P[1]	95,8
24	KU708259-RVA/Deer-wt/SLO/D38-14/2014/G6P[15]	95,7
25	LC336591-RVA/Human-wt/JPN/12-1375/2012/G8P[14]	95,6
26	KJ919388-RVA/Human-wt/HUN/ERN5635/2012/G2P[4]	95,6
27	LC133558-RVA/Cow-tc/JPN/KK3/1983/G10P[11]	95,2
28	**RVA/Human-wt/RWA/UFS-NGS:MRC-DPRU566/2013/G9P[4]**	**94,1**
29	JQ069919-RVA/Human-wt/CAN/RT125-07/2008/G2P[4]	85,1
30	KC834712-RVA/Human-wt/AUS/336190/2004/G2P[4]	84,9
31	KC834711-RVA/Human-wt/AUS/V203/2009/G2P[4]	84,7
32	KC443631-RVA/Human-wt/AUS/CK20028/2006/G2P[4]	84,6
33	KM660259-RVA/Human-wt/CMR/BA368/2010/G2P[4]	84,6
34	LC172978-RVA/Human/JPN/SP030/2013/G1P[8]	84,5
35	MF044131-RVA/Human-wt/TWN/2014/103-701-D166/G1P[8]	84,4
36	KX362645-RVA/Human-wt/VNM/16020_44/G4P[6]	84,3
37	LC169973-RVA/Human-wt/THA/LS-202/2014/G2P[4]	84,3
38	KX362744-RVA/Human-wt/VNM/16020_81/G2P[4]	84,3
39	LC169984-RVA/Human-wt/THA/LS-L7/2014/G2P[4]	84,3
40	DQ870505-RVA/Human-tc/USA/DS-1/1976/G2P[4]	84,2
41	**RVA/Human-wt/RWA/UFS-NGS:MRC-DPRU6235/2014/G4P[4]**	**82,6**
42	DQ490533-RVA/Human-tc/JPN/AU-1/1982/G3P3[9]	75,2
43	KF371688-RVA/Human-tc/CHN/R709/2005/G3P[8]	73,0
44	LC368099-RVA/Human-wt/NPL/5N0109/2005/G12P[6]	73,0
45	KJ753147-RVA/Human-wt/ZAF/MRC-DPRU840/2006/G1P[8]	73,0
46	LC368088-RVA/Human-wt/NPL/5N0014/2005/G12P[6]	72,9
47	KJ627112-RVA/Human-wt/PRY/371/1999/G1P[8]	72,8
48	KJ752469-RVA/Human-wt/ZAF/MRC-DPRU1911/2007/G12P[6]	72,8
49	KJ753158-RVA/Human-wt/ZAF/MRC-DPRU2132/2005/G1P[8]	72,7
50	**RVA/Human-wt/RWA/UFS-NGS:MRC-DPRU6212/2014/G12P[8]**	**72,7**
51	JN258830-RVA/Human-wt/Bel/BE00073/2001/G1P[8]	72,7
52	KC193633-RVA/Human-wt/Bel/BE00092/2003/G1P[8]	72,6
53	KJ559249-RVA/Human-wt/ARG/SF931/1998/G4P[8]	72,6
54	JX943609-RVA/Human-tc/USA/Rotarix/2009/G1P[8]	72,6
55	KJ559029-RVA/Human-wt/ARG/Tuc1650/1998/G4P[8]	72,5
56	DQ490539-RVA/Human-tc/USA/Wa/1974/G1P[8]	72,5

	**RVA/Human-wt/RWA/UFS-NGS:MRC-DPRU6212/2014/G12P[8]**	
1	LC368088-RVA/Human-wt/NPL/5N0014/2005/G12P[6]	99,0
2	LC368099-RVA/Human-wt/NPL/5N0109/2005/G12P[6]	98,9
3	KJ753147-RVA/Human-wt/ZAF/MRC-DPRU840/2006/G1P[8]	98,9
4	KJ752469-RVA/Human-wt/ZAF/MRC-DPRU1911/2007/G12P[6]	98,8
5	KC193633-RVA/Human-wt/Bel/BE00092/2003/G1P[8]	98,6
6	KJ753158-RVA/Human-wt/ZAF/MRC-DPRU2132/2005/G1P[8]	98,5
7	KJ559249-RVA/Human-wt/ARG/SF931/1998/G4P[8]	97,8
8	KJ559029-RVA/Human-wt/ARG/Tuc1650/1998/G4P[8]	97,8
9	KJ627112-RVA/Human-wt/PRY/371/1999/G1P[8]	97,7
10	JN258830-RVA/Human-wt/Bel/BE00073/2001/G1P[8]	97,6
11	KF371688-RVA/Human-tc/CHN/R709/2005/G3P[8]	97,3
12	DQ490539-RVA/Human-tc/USA/Wa/1974/G1P[8]	94,8
13	JX943609-RVA/Human-tc/USA/Rotarix/2009/G1P[8]	94,3
14	**RVA/Human-wt/RWA/UFS-NGS:MRC-DPRU566/2013/G9P[4]**	**80,2**
15	**RVA/Human-wt/RWA/UFS-NGS:MRC-DPRU6235/2014/G4P[4]**	**79,9**
16	LC172978-RVA/Human/JPN/SP030/2013/G1P[8]	75,2
17	MF044131-RVA/Human-wt/TWN/2014/103-701-D166/G1P[8]	75,1
18	DQ490533-RVA/Human-tc/JPN/AU-1/1982/G3P3[9]	74,9
19	KC834711-RVA/Human-wt/AUS/V203/2009/G2P[4]	74,9
20	KX362645-RVA/Human-wt/VNM/16020_44/G4P[6]	74,8
21	KM660259-RVA/Human-wt/CMR/BA368/2010/G2P[4]	74,7
22	KX362744-RVA/Human-wt/VNM/16020_81/G2P[4]	74,7
23	LC169973-RVA/Human-wt/THA/LS-202/2014/G2P[4]	74,7
24	LC169984-RVA/Human-wt/THA/LS-L7/2014/G2P[4]	74,7
25	KC443631-RVA/Human-wt/AUS/CK20028/2006/G2P[4]	74,6
26	KC834712-RVA/Human-wt/AUS/336190/2004/G2P[4]	74,6
27	JQ069919-RVA/Human-wt/CAN/RT125-07/2008/G2P[4]	74,5
28	DQ870505-RVA/Human-tc/USA/DS-1/1976/G2P[4]	73,5
29	KP006506-RVA/Human-wt/GTM/2009726790/2009/G8P[14]	73,2
30	AB853890-RVA/Cow-wt/JPN/Tottori-SG/2013/G15P[14]	73,1
31	KU708259-RVA/Deer-wt/SLO/D38-14/2014/G6P[15]	73,0
32	FJ422131-RVA/Rhesus-tc/USA/PTRV/1990/G8P[1]	73,0
33	EF554093-RVA/Human-tc/AUS/MG6/1993/G6P[14]	72,9
34	KU956006-RVA/Human/HON/2011825363/2011/G10P[14]	72,9
35	LC095957-RVA/Human-wt/VNM/NT0082/2007/G10P[14]	72,8
36	JF693026-bovine-tc/USA/NCDV/1971/G6P[1]	72,8
37	LC133536-RVA/Cow-tc/THA/61A/1989/G10P[5]	72,8
38	EF583006-RVA/Simian-tc/USA/RRV/1975/G3P[3]	72,8
39	LC336591-RVA/Human-wt/JPN/12-1375/2012/G8P[14]	72,8
40	LC384330-RVA/Human-wt/JPN/HKD0825/2016/G1P[8]	72,8
41	GU565041-RVA/Vaccine/USA/RotaTeq-WI79-4/1992/G6P1[8]	72,8
42	KT919447-RVA/Human-wt/USA/VU12-13-176/2013/G1G6P[8]	72,8
43	KJ919388-RVA/Human-wt/HUN/ERN5635/2012/G2P[4]	72,7
44	LC133558-RVA/Cow-tc/JPN/KK3/1983/G10P[11]	72,7
45	GU565085-RVA/Vaccine/USA/RotaTeq-BrB-9/1996/G4P7[5]	72,7
46	**RVA/Human-wt/RWA/UFS-NGS:MRC-DPRU9995/2015/G12P[8]**	**72,7**
47	**RVA/Human-wt/RWA/UFS-NGS:MRC-DPRU8020/2015/G12P[8]**	**72,7**
48	EF560615-RVA/Cow-tc/USA/WC3/1981/G6P[5]	72,7
49	GU565052-RVA/Vaccine/USA/RotaTeq-WI79-9/1992/G1P7[5]	72,7
50	KC443598-RVA/Human-wt/AUS/CK20039/2008/G1P[8]	72,7
51	KR705300-RVA/Human-wt/BEL/BE41/2007/G2P[4]	72,6
52	MF940641-RVA/Bovine-tc/KOR/KJ19-2/2006/G6P[7]	72,5
53	LC133547-RVA/Cow-tc/USA/B223/1983/G10P[11]	72,4
54	AB748573-RVA/Cow-tc/JPN/BRV105/1983/G6P[1]	72,4
55	AB748576-RVA/Human-tc/ISR/Ro8059/1995/G6P[1]	72,1
56	KF541281-RVA/Human-wt/US/09US7118/2009/G3P[24]	71,9

D. VP3

	**RVA/Human-wt/RWA/UFS-NGS:MRC-DPRU6235/2014/G4P[4]**	
1	KC443633-RVA/Human-wt/AUS/CK20028/2006/G2P[4]	94,5
2	KC443765-RVA/Human-wt/AUS/CK20027/2006/G2P[4]	94,5
3	KR705269-RVA/Human-wt/BEL/BE34/2006/G2P[4]	94,4
4	KY497531-RVA/Human-wt/PAK/3085/2010/G2P[4]	94,3
5	LC001994-RVA/Human-wt/JPN/B110056/2011/G2P[4]	94,2
6	MF184838-RVA/Human-wt/USA/CNMC123/2011/G2P[4]	94,0
7	KY497553-RVA/Human-wt/PAK93/2010/G1P[6]	93,9
8	FN665690-RVA/Human-wt/HUN/BP1062/2004/G8P[14]	92,0
9	JX416213-RVA/Human-tc/SWE/1076/1983/G2P2A[6]	91,3
10	KJ752620-RVA/Human-wt/SEN/MRC-DPRU2053/2009/G8P[6]	91,2
11	KU356642-RVA/Human-wt/BGN/M292/2013/G2P[4]	90,8
12	LC133527-RVA/Cow-tc/THA/A5-13/1988/G8P[1]	90,8
13	LC227959-RVA/Human-wt/IND/Kol-047/2013/G9P[4]	90,7
14	LC227964-RVA/Human-wt/IND/Kol-066/2013/G9P[4]	90,7
15	KC782521.2-RVA/Human-wt/USA/LB1562/2010/G9P[4]	90,7
16	MH170019-RVA/Human-wt/PAK585/2016/G1P[8]	90,7
17	KP007153-RVA/Human-wt/PHI/TGO12-003/2012/G2P[4]	90,6
18	MH170018-RVA/Human-wt/PAK419/2016/G3P[4]	90,6
19	MG891995-RVA/Human-wt/MOZ/0126/2013/G2P[4]	90,6
20	KU248374-RVA/Human-wt/BGN/J303/2010/G2P[4]	90,6
21	MG926749-RVA/Human-wt/MOZ/0440/2013/G2P[4]	90,6
22	KC571495-RVA/Human-wt/AUS/SA066/2010/G2P[4]	90,6
23	LC477526-RVA/Human-wt/JPN/Tokyo18-42/2018/G2P[4]	90,6
24	JX965141-RVA/Human/AUS/MON008/2010/G2P[4]	90,6
25	LC173352-RVA/Human-xx/JPN/SP028/2013/G2P[4]	90,5
26	KU714446-RVA/Human-wt/MWI/MW2-181_A/2000/G1P[8]	90,5
27	HQ650118-RVA/Human-tc/USA/DS-1/1976/G2P[4]	89,2
28	LC086760-RVA/Human-wt/THA/SKT-98/2013/G1P[8]	88,3
29	KX363074-RVA/Human-wt/VNM/12053_56/GXP[X]	88,3
30	LC086749-RVA/Human-wt/THA/PCB-118/2013/G1P[8]	88,2
31	JQ087436-RVA/Human-tc/CHN/R588/2005/G1P[8]	88,2
32	**RVA/Human-wt/RWA/UFS-NGS:MRC-DPRU9995/2015/G12P[8]**	**88,2**
33	**RVA/Human-wt/RWA/UFS-NGS:MRC-DPRU8020/2015/G12P[8]**	**88,2**
34	LC173289-RVA/Human-wt/JPN/KN144/2013/G3P[8]	88,2
35	LC173257-RVA/Human-wt/JPN/KN105/2013/G3P[8]	88,2
36	LC173350-RVA/Human-xx/JPN/SP026/2013/G9P[8]	88,1
37	MK417778-RVA/Human-wt/ESP/SS67922985/2018/G12P[8]	87,8
38	KU048582-RVA/Human-wt/ITA/ME650/14/2014/G12P[8]	87,8
39	KU048581-RVA/Human-wt/ITA/PA525/14/2014/G12P[8]	87,8
40	LC368101-RVA/Human-wt/NPL/5N0109/2005/G12P[6]	87,7
41	MK417779-RVA/Human-wt/ESP/SS96099331/2018/G12P[8]	87,7
42	KU048577-RVA/Human-wt/ITA/RG178/13/2013/G12P[8]	87,7
43	KT919769-RVA/Human-wt/USA/VU11-12-67/2012/G12P[8]	87,7
44	AB861958-RVA/Human-tc/KEN/KDH651/2010/G12P[8]	87,7
45	MG926778-RVA/Human-wt/MOZ/0042/2012/G12P[6]	87,6
46	**RVA/Human-wt/RWA/UFS-NGS:MRC-DPRU6212/2014/G12P[8]**	**87,5**
47	LC374153-RVA/Human-wt/NPL/09N3472/2009/G12P[6]	87,5
48	MG926705-RVA/Human-wt/MOZ/0278/2012/G12P[6]	87,5
49	LC374175-RVA/Human-wt/NPL/10N3879/2010/G12P[6]	87,5
50	KJ752816-RVA/Human-wt/ZAF/MRC-DPRU4090/2011/G12P[6]	87,4
51	LC019054-RVA/Human-tc/MMR/A23/2011/G12P[6]	87,4
52	LC374208-RVA/Human-wt/NPL/10N4594/2010/G12P[6]	87,4
53	KT694941-RVA/Human-tc/USA/Wa/1974/G1P[8]	86,4
54	RVA/Vaccine/USA/RotaTeq-WI79-9/1992/G1P7[5]	86,3
55	**RVA/Human-wt/RWA/UFS-NGS:MRC-DPRU566/2013/G9P[4]**	**86,1**
56	MF940540-RVA/Porcine-tc/KOR/174-1/2006/G8P[7]	86,1
57	KC174868-RVA/Human-wt/IND/N36/2003/G10P[11]	86,1
58	LC133538-RVA/Cow-tc/THA/61A/1989/G10P[5]	86,0
59	MF940597-RVA/Bovine-tc/KOR/KJ11/2006/G8P[7]	86,0
60	JX943611-RVA/Human-tc/USA/Rotarix/2009/G1P[8]	86,0
61	KC174890-RVA/Human-wt/IND/N62/2003/G10P[11]	86,0
62	LC133560-RVA/Cow-tc/JPN/KK3/1983/G10P[11]	85,9
63	DQ870495-RVA/Cow-tc/USA/NCDV/1967/G6P6[1]	85,9
64	FJ347124-RVA/Guanaco-wt/ARG/1998/G8P[1]	85,6
65	LC074700-RVA/Cat-tc/JPN/FRV537/2004/G6P[5]	85,6
66	KY426811-RVA/Deer-wt/SLO/D110-15/2015/G8P[14]	85,6
67	JN831222-RVA/Cow-wt/ZAF/1604/2007/G8P[1]	85,5
68	KF636258-RVA/Cow/ZAF/MRC-DPRU1604/2007/G6P[1]	85,5
69	MF940652-RVA/Bovine-tc/KOR/KJ19-2/2006/G6P[7]	85,4
70	JN831233-RVA/Cow-wt/ZAF/1605/2007/G6P[5]	85,4
71	JN831211-RVA/Cow-wt/ZAF/1603/2007/G6P[5]	85,4
72	GU565043-RVA/Vaccine/USA/RotaTeq-WI79-4/1992/G6P1A[8]	85,3
73	GU565076-RVA/Vaccine/USA/RotaTeq-WI78-8/1992/G3P7[5]	85,3
74	GU565087-RVA/Vaccine/USA/RotaTeq-BrB-9/1996/G4P7[5]	85,3
75	DQ490537-RVA/Human-tc/JPN/AU-1/1982/G3P3[9]	81,6

	**RVA/Human-wt/RWA/UFS-NGS:MRC-DPRU566/2013/G9P[4]**	
1	GU565043-RVA/Vaccine/USA/RotaTeq-WI79-4/1992/G6P1A[8]	94,1
2	GU565076-RVA/Vaccine/USA/RotaTeq-WI78-8/1992/G3P7[5]	94,1
3	GU565087-RVA/Vaccine/USA/RotaTeq-BrB-9/1996/G4P7[5]	94,1
4	FJ347124-RVA/Guanaco-wt/ARG/1998/G8P[1]	93,0
5	LC133560-RVA/Cow-tc/JPN/KK3/1983/G10P[11]	92,6
6	JN831222-RVA/Cow-wt/ZAF/1604/2007/G8P[1]	91,4
7	KF636258-RVA/Cow/ZAF/MRC-DPRU1604/2007/G6P[1]	91,3
8	JN831233-RVA/Cow-wt/ZAF/1605/2007/G6P[5]	91,2
9	JN831211-RVA/Cow-wt/ZAF/1603/2007/G6P[5]	91,2
10	MF940652-RVA/Bovine-tc/KOR/KJ19-2/2006/G6P[7]	91,1
11	LC074700-RVA/Cat-tc/JPN/FRV537/2004/G6P[5]	91,0
12	KY426811-RVA/Deer-wt/SLO/D110-15/2015/G8P[14]	91,0
13	LC133538-RVA/Cow-tc/THA/61A/1989/G10P[5]	90,9
14	DQ870495-RVA/Cow-tc/USA/NCDV/1967/G6P6[1]	90,8
15	MF940540-RVA/Porcine-tc/KOR/174-1/2006/G8P[7]	90,7
16	KC174868-RVA/Human-wt/IND/N36/2003/G10P[11]	90,7
17	MF940597-RVA/Bovine-tc/KOR/KJ11/2006/G8P[7]	90,6
18	KC174890-RVA/Human-wt/IND/N62/2003/G10P[11]	90,6
19	LC368101-RVA/Human-wt/NPL/5N0109/2005/G12P[6]	88,2
20	AB861958-RVA/Human-tc/KEN/KDH651/2010/G12P[8]	88,2
21	MK417779-RVA/Human-wt/ESP/SS96099331/2018/G12P[8]	88,1
22	KT919769-RVA/Human-wt/USA/VU11-12-67/2012/G12P[8]	88,1
23	LC374153-RVA/Human-wt/NPL/09N3472/2009/G12P[6]	88,1
24	MK417778-RVA/Human-wt/ESP/SS67922985/2018/G12P[8]	88,1
25	LC374175-RVA/Human-wt/NPL/10N3879/2010/G12P[6]	88,1
26	KU048582-RVA/Human-wt/ITA/ME650/14/2014/G12P[8]	88,1
27	KU048581-RVA/Human-wt/ITA/PA525/14/2014/G12P[8]	88,1
28	MG926778-RVA/Human-wt/MOZ/0042/2012/G12P[6]	88,1
29	MG926705-RVA/Human-wt/MOZ/0278/2012/G12P[6]	88,1
30	LC374208-RVA/Human-wt/NPL/10N4594/2010/G12P[6]	88,1
31	KU048577-RVA/Human-wt/ITA/RG178/13/2013/G12P[8]	88,0
32	LC019054-RVA/Human-tc/MMR/A23/2011/G12P[6]	88,0
33	KJ752816-RVA/Human-wt/ZAF/MRC-DPRU4090/2011/G12P[6]	88,0
34	**RVA/Human-wt/RWA/UFS-NGS:MRC-DPRU6212/2014/G12P[8]**	**88,0**
35	JQ087436-RVA/Human-tc/CHN/R588/2005/G1P[8]	87,5
36	LC173289-RVA/Human-wt/JPN/KN144/2013/G3P[8]	87,5
37	LC173257-RVA/Human-wt/JPN/KN105/2013/G3P[8]	87,5
38	KX363074-RVA/Human-wt/VNM/12053_56/GXP[X]	87,4
39	LC086760-RVA/Human-wt/THA/SKT-98/2013/G1P[8]	87,4
40	**RVA/Human-wt/RWA/UFS-NGS:MRC-DPRU9995/2015/G12P[8]**	**87,3**
41	LC173350-RVA/Human-xx/JPN/SP026/2013/G9P[8]	87,3
42	LC086749-RVA/Human-wt/THA/PCB-118/2013/G1P[8]	87,3
43	**RVA/Human-wt/RWA/UFS-NGS:MRC-DPRU8020/2015/G12P[8]**	**87,3**
44	KT694941-RVA/Human-tc/USA/Wa/1974/G1P[8]	86,3
45	**RVA/Human-wt/RWA/UFS-NGS:MRC-DPRU6235/2014/G4P[4]**	**86,1**
46	JX943611-RVA/Human-tc/USA/Rotarix/2009/G1P[8]	86,1
47	LC227964-RVA/Human-wt/IND/Kol-066/2013/G9P[4]	86,1
48	HQ650118-RVA/Human-tc/USA/DS-1/1976/G2P[4]	86,1
49	RVA/Vaccine/USA/RotaTeq-WI79-9/1992/G1P7[5]	86,0
50	LC227959-RVA/Human-wt/IND/Kol-047/2013/G9P[4]	86,0
51	LC173352-RVA/Human-xx/JPN/SP028/2013/G2P[4]	86,0
52	KP007153-RVA/Human-wt/PHI/TGO12-003/2012/G2P[4]	85,9
53	LC477526-RVA/Human-wt/JPN/Tokyo18-42/2018/G2P[4]	85,9
54	MH170018-RVA/Human-wt/PAK419/2016/G3P[4]	85,8
55	KU248374-RVA/Human-wt/BGN/J303/2010/G2P[4]	85,8
56	MG891995-RVA/Human-wt/MOZ/0126/2013/G2P[4]	85,8
57	MG926749-RVA/Human-wt/MOZ/0440/2013/G2P[4]	85,8
58	MH170019-RVA/Human-wt/PAK585/2016/G1P[8]	85,8
59	JX965141-RVA/Human/AUS/MON008/2010/G2P[4]	85,8
60	KC782521.2-RVA/Human-wt/USA/LB1562/2010/G9P[4]	85,7
61	KC571495-RVA/Human-wt/AUS/SA066/2010/G2P[4]	85,7
62	KU356642-RVA/Human-wt/BGN/M292/2013/G2P[4]	85,7
63	JX416213-RVA/Human-tc/SWE/1076/1983/G2P2A[6]	85,6
64	LC001994-RVA/Human-wt/JPN/B110056/2011/G2P[4]	85,6
65	KR705269-RVA/Human-wt/BEL/BE34/2006/G2P[4]	85,4
66	KY497531-RVA/Human-wt/PAK/3085/2010/G2P[4]	85,4
67	KC443633-RVA/Human-wt/AUS/CK20028/2006/G2P[4]	85,4
68	KC443765-RVA/Human-wt/AUS/CK20027/2006/G2P[4]	85,4
69	FN665690-RVA/Human-wt/HUN/BP1062/2004/G8P[14]	85,3
70	LC133527-RVA/Cow-tc/THA/A5-13/1988/G8P[1]	85,3
71	MF184838-RVA/Human-wt/USA/CNMC123/2011/G2P[4]	85,2
72	KY497553-RVA/Human-wt/PAK93/2010/G1P[6]	85,1
73	KJ752620-RVA/Human-wt/SEN/MRC-DPRU2053/2009/G8P[6]	85,1
74	KU714446-RVA/Human-wt/MWI/MW2-181_A/2000/G1P[8]	85,0
75	DQ490537-RVA/Human-tc/JPN/AU-1/1982/G3P3[9]	81,9

	**RVA/Human-wt/RWA/UFS-NGS:MRC-DPRU8020/2015/G12P[8]**	
1	**RVA/Human-wt/RWA/UFS-NGS:MRC-DPRU9995/2015/G12P[8]**	**100,0**
2	KX363074-RVA/Human-wt/VNM/12053_56/GXP[X]	99,5
3	LC086749-RVA/Human-wt/THA/PCB-118/2013/G1P[8]	99,4
4	JQ087436-RVA/Human-tc/CHN/R588/2005/G1P[8]	99,4
5	LC086760-RVA/Human-wt/THA/SKT-98/2013/G1P[8]	99,4
6	LC173289-RVA/Human-wt/JPN/KN144/2013/G3P[8]	99,3
7	LC173257-RVA/Human-wt/JPN/KN105/2013/G3P[8]	99,3
8	LC173350-RVA/Human-xx/JPN/SP026/2013/G9P[8]	99,2
9	LC368101-RVA/Human-wt/NPL/5N0109/2005/G12P[6]	98,1
10	KT919769-RVA/Human-wt/USA/VU11-12-67/2012/G12P[8]	97,9
11	LC374153-RVA/Human-wt/NPL/09N3472/2009/G12P[6]	97,8
12	LC374175-RVA/Human-wt/NPL/10N3879/2010/G12P[6]	97,7
13	AB861958-RVA/Human-tc/KEN/KDH651/2010/G12P[8]	97,7
14	LC019054-RVA/Human-tc/MMR/A23/2011/G12P[6]	97,7
15	KU048582-RVA/Human-wt/ITA/ME650/14/2014/G12P[8]	97,7
16	KU048581-RVA/Human-wt/ITA/PA525/14/2014/G12P[8]	97,7
17	MG926778-RVA/Human-wt/MOZ/0042/2012/G12P[6]	97,7
18	LC374208-RVA/Human-wt/NPL/10N4594/2010/G12P[6]	97,7
19	MG926705-RVA/Human-wt/MOZ/0278/2012/G12P[6]	97,6
20	MK417779-RVA/Human-wt/ESP/SS96099331/2018/G12P[8]	97,6
21	KU048577-RVA/Human-wt/ITA/RG178/13/2013/G12P[8]	97,6
22	MK417778-RVA/Human-wt/ESP/SS67922985/2018/G12P[8]	97,6
23	KJ752816-RVA/Human-wt/ZAF/MRC-DPRU4090/2011/G12P[6]	97,6
24	**RVA/Human-wt/RWA/UFS-NGS:MRC-DPRU6212/2014/G12P[8]**	**97,3**
25	KT694941-RVA/Human-tc/USA/Wa/1974/G1P[8]	94,8
26	RVA/Vaccine/USA/RotaTeq-WI79-9/1992/G1P7[5]	94,6
27	JX943611-RVA/Human-tc/USA/Rotarix/2009/G1P[8]	94,5
28	**RVA/Human-wt/RWA/UFS-NGS:MRC-DPRU6235/2014/G4P[4]**	**88,2**
29	**RVA/Human-wt/RWA/UFS-NGS:MRC-DPRU566/2013/G9P[4]**	**87,3**
30	DQ490537-RVA/Human-tc/JPN/AU-1/1982/G3P3[9]	82,4
31	HQ650118-RVA/Human-tc/USA/DS-1/1976/G2P[4]	82,2
32	KC571495-RVA/Human-wt/AUS/SA066/2010/G2P[4]	82,0
33	KU356642-RVA/Human-wt/BGN/M292/2013/G2P[4]	81,9
34	LC173352-RVA/Human-xx/JPN/SP028/2013/G2P[4]	81,7
35	MH170019-RVA/Human-wt/PAK585/2016/G1P[8]	81,7
36	KJ752620-RVA/Human-wt/SEN/MRC-DPRU2053/2009/G8P[6]	81,6
37	KU248374-RVA/Human-wt/BGN/J303/2010/G2P[4]	81,6
38	KP007153-RVA/Human-wt/PHI/TGO12-003/2012/G2P[4]	81,5
39	LC227959-RVA/Human-wt/IND/Kol-047/2013/G9P[4]	81,5
40	LC227964-RVA/Human-wt/IND/Kol-066/2013/G9P[4]	81,5
41	MG926749-RVA/Human-wt/MOZ/0440/2013/G2P[4]	81,5
42	JX965141-RVA/Human/AUS/MON008/2010/G2P[4]	81,5
43	MG891995-RVA/Human-wt/MOZ/0126/2013/G2P[4]	81,5
44	LC477526-RVA/Human-wt/JPN/Tokyo18-42/2018/G2P[4]	81,5
45	KC782521.2-RVA/Human-wt/USA/LB1562/2010/G9P[4]	81,4
46	MH170018-RVA/Human-wt/PAK419/2016/G3P[4]	81,4
47	LC001994-RVA/Human-wt/JPN/B110056/2011/G2P[4]	81,4
48	JX416213-RVA/Human-tc/SWE/1076/1983/G2P2A[6]	81,4
49	KY497531-RVA/Human-wt/PAK/3085/2010/G2P[4]	81,4
50	KC443633-RVA/Human-wt/AUS/CK20028/2006/G2P[4]	81,4
51	KC443765-RVA/Human-wt/AUS/CK20027/2006/G2P[4]	81,4
52	KY497553-RVA/Human-wt/PAK93/2010/G1P[6]	81,4
53	KR705269-RVA/Human-wt/BEL/BE34/2006/G2P[4]	81,4
54	LC133527-RVA/Cow-tc/THA/A5-13/1988/G8P[1]	81,3
55	FN665690-RVA/Human-wt/HUN/BP1062/2004/G8P[14]	81,3
56	MF184838-RVA/Human-wt/USA/CNMC123/2011/G2P[4]	81,3
57	KU714446-RVA/Human-wt/MWI/MW2-181_A/2000/G1P[8]	81,2
58	LC133538-RVA/Cow-tc/THA/61A/1989/G10P[5]	81,1
59	KC174868-RVA/Human-wt/IND/N36/2003/G10P[11]	81,0
60	KC174890-RVA/Human-wt/IND/N62/2003/G10P[11]	80,9
61	DQ870495-RVA/Cow-tc/USA/NCDV/1967/G6P6[1]	80,8
62	MF940540-RVA/Porcine-tc/KOR/174-1/2006/G8P[7]	80,7
63	JN831222-RVA/Cow-wt/ZAF/1604/2007/G8P[1]	80,7
64	KF636258-RVA/Cow/ZAF/MRC-DPRU1604/2007/G6P[1]	80,6
65	LC074700-RVA/Cat-tc/JPN/FRV537/2004/G6P[5]	80,6
66	KY426811-RVA/Deer-wt/SLO/D110-15/2015/G8P[14]	80,6
67	FJ347124-RVA/Guanaco-wt/ARG/1998/G8P[1]	80,6
68	JN831233-RVA/Cow-wt/ZAF/1605/2007/G6P[5]	80,6
69	LC133560-RVA/Cow-tc/JPN/KK3/1983/G10P[11]	80,6
70	GU565043-RVA/Vaccine/USA/RotaTeq-WI79-4/1992/G6P1A[8]	80,6
71	GU565076-RVA/Vaccine/USA/RotaTeq-WI78-8/1992/G3P7[5]	80,6
72	GU565087-RVA/Vaccine/USA/RotaTeq-BrB-9/1996/G4P7[5]	80,6
73	MF940597-RVA/Bovine-tc/KOR/KJ11/2006/G8P[7]	80,6
74	JN831211-RVA/Cow-wt/ZAF/1603/2007/G6P[5]	80,6
75	MF940652-RVA/Bovine-tc/KOR/KJ19-2/2006/G6P[7]	80,4

	**RVA/Human-wt/RWA/UFS-NGS:MRC-DPRU9995/2015/G12P[8]**	
1	**RVA/Human-wt/RWA/UFS-NGS:MRC-DPRU8020/2015/G12P[8]**	**100,0**
2	KX363074-RVA/Human-wt/VNM/12053_56/GXP[X]	99,5
3	LC086749-RVA/Human-wt/THA/PCB-118/2013/G1P[8]	99,5
4	JQ087436-RVA/Human-tc/CHN/R588/2005/G1P[8]	99,5
5	LC086760-RVA/Human-wt/THA/SKT-98/2013/G1P[8]	99,4
6	LC173289-RVA/Human-wt/JPN/KN144/2013/G3P[8]	99,3
7	LC173257-RVA/Human-wt/JPN/KN105/2013/G3P[8]	99,3
8	LC173350-RVA/Human-xx/JPN/SP026/2013/G9P[8]	99,2
9	LC368101-RVA/Human-wt/NPL/5N0109/2005/G12P[6]	98,1
10	KT919769-RVA/Human-wt/USA/VU11-12-67/2012/G12P[8]	97,9
11	LC374153-RVA/Human-wt/NPL/09N3472/2009/G12P[6]	97,8
12	LC374175-RVA/Human-wt/NPL/10N3879/2010/G12P[6]	97,8
13	AB861958-RVA/Human-tc/KEN/KDH651/2010/G12P[8]	97,7
14	LC019054-RVA/Human-tc/MMR/A23/2011/G12P[6]	97,7
15	KU048582-RVA/Human-wt/ITA/ME650/14/2014/G12P[8]	97,7
16	KU048581-RVA/Human-wt/ITA/PA525/14/2014/G12P[8]	97,7
17	MG926778-RVA/Human-wt/MOZ/0042/2012/G12P[6]	97,7
18	LC374208-RVA/Human-wt/NPL/10N4594/2010/G12P[6]	97,7
19	MK417779-RVA/Human-wt/ESP/SS96099331/2018/G12P[8]	97,7
20	KU048577-RVA/Human-wt/ITA/RG178/13/2013/G12P[8]	97,6
21	MK417778-RVA/Human-wt/ESP/SS67922985/2018/G12P[8]	97,6
22	MG926705-RVA/Human-wt/MOZ/0278/2012/G12P[6]	97,6
23	KJ752816-RVA/Human-wt/ZAF/MRC-DPRU4090/2011/G12P[6]	97,6
24	**RVA/Human-wt/RWA/UFS-NGS:MRC-DPRU6212/2014/G12P[8]**	**97,3**
25	KT694941-RVA/Human-tc/USA/Wa/1974/G1P[8]	94,8
26	RVA/Vaccine/USA/RotaTeq-WI79-9/1992/G1P7[5]	94,6
27	JX943611-RVA/Human-tc/USA/Rotarix/2009/G1P[8]	94,5
28	**RVA/Human-wt/RWA/UFS-NGS:MRC-DPRU6235/2014/G4P[4]**	**88,2**
29	**RVA/Human-wt/RWA/UFS-NGS:MRC-DPRU566/2013/G9P[4]**	**87,3**
30	DQ490537-RVA/Human-tc/JPN/AU-1/1982/G3P3[9]	82,5
31	HQ650118-RVA/Human-tc/USA/DS-1/1976/G2P[4]	82,2
32	KC571495-RVA/Human-wt/AUS/SA066/2010/G2P[4]	82,0
33	KU356642-RVA/Human-wt/BGN/M292/2013/G2P[4]	82,0
34	LC173352-RVA/Human-xx/JPN/SP028/2013/G2P[4]	81,8
35	MH170019-RVA/Human-wt/PAK585/2016/G1P[8]	81,7
36	KJ752620-RVA/Human-wt/SEN/MRC-DPRU2053/2009/G8P[6]	81,6
37	KU248374-RVA/Human-wt/BGN/J303/2010/G2P[4]	81,6
38	KP007153-RVA/Human-wt/PHI/TGO12-003/2012/G2P[4]	81,6
39	LC227959-RVA/Human-wt/IND/Kol-047/2013/G9P[4]	81,6
40	LC227964-RVA/Human-wt/IND/Kol-066/2013/G9P[4]	81,6
41	JX965141-RVA/Human/AUS/MON008/2010/G2P[4]	81,5
42	LC477526-RVA/Human-wt/JPN/Tokyo18-42/2018/G2P[4]	81,5
43	MG891995-RVA/Human-wt/MOZ/0126/2013/G2P[4]	81,5
44	KC782521.2-RVA/Human-wt/USA/LB1562/2010/G9P[4]	81,5
45	MH170018-RVA/Human-wt/PAK419/2016/G3P[4]	81,5
46	MG926749-RVA/Human-wt/MOZ/0440/2013/G2P[4]	81,5
47	LC001994-RVA/Human-wt/JPN/B110056/2011/G2P[4]	81,4
48	JX416213-RVA/Human-tc/SWE/1076/1983/G2P2A[6]	81,4
49	KY497531-RVA/Human-wt/PAK/3085/2010/G2P[4]	81,4
50	KC443633-RVA/Human-wt/AUS/CK20028/2006/G2P[4]	81,4
51	KC443765-RVA/Human-wt/AUS/CK20027/2006/G2P[4]	81,4
52	KY497553-RVA/Human-wt/PAK93/2010/G1P[6]	81,4
53	KR705269-RVA/Human-wt/BEL/BE34/2006/G2P[4]	81,4
54	LC133527-RVA/Cow-tc/THA/A5-13/1988/G8P[1]	81,3
55	FN665690-RVA/Human-wt/HUN/BP1062/2004/G8P[14]	81,3
56	MF184838-RVA/Human-wt/USA/CNMC123/2011/G2P[4]	81,3
57	KU714446-RVA/Human-wt/MWI/MW2-181_A/2000/G1P[8]	81,2
58	LC133538-RVA/Cow-tc/THA/61A/1989/G10P[5]	81,1
59	KC174868-RVA/Human-wt/IND/N36/2003/G10P[11]	81,0
60	KC174890-RVA/Human-wt/IND/N62/2003/G10P[11]	80,9
61	DQ870495-RVA/Cow-tc/USA/NCDV/1967/G6P6[1]	80,8
62	MF940540-RVA/Porcine-tc/KOR/174-1/2006/G8P[7]	80,8
63	JN831222-RVA/Cow-wt/ZAF/1604/2007/G8P[1]	80,7
64	KF636258-RVA/Cow/ZAF/MRC-DPRU1604/2007/G6P[1]	80,7
65	LC074700-RVA/Cat-tc/JPN/FRV537/2004/G6P[5]	80,7
66	KY426811-RVA/Deer-wt/SLO/D110-15/2015/G8P[14]	80,7
67	FJ347124-RVA/Guanaco-wt/ARG/1998/G8P[1]	80,7
68	JN831233-RVA/Cow-wt/ZAF/1605/2007/G6P[5]	80,7
69	LC133560-RVA/Cow-tc/JPN/KK3/1983/G10P[11]	80,6
70	GU565043-RVA/Vaccine/USA/RotaTeq-WI79-4/1992/G6P1A[8]	80,6
71	GU565076-RVA/Vaccine/USA/RotaTeq-WI78-8/1992/G3P7[5]	80,6
72	GU565087-RVA/Vaccine/USA/RotaTeq-BrB-9/1996/G4P7[5]	80,6
73	MF940597-RVA/Bovine-tc/KOR/KJ11/2006/G8P[7]	80,6
74	JN831211-RVA/Cow-wt/ZAF/1603/2007/G6P[5]	80,6
75	MF940652-RVA/Bovine-tc/KOR/KJ19-2/2006/G6P[7]	80,4

	**RVA/Human-wt/RWA/UFS-NGS:MRC-DPRU6212/2014/G12P[8]**	
1	LC374175-RVA/Human-wt/NPL/10N3879/2010/G12P[6]	99,5
2	LC374153-RVA/Human-wt/NPL/09N3472/2009/G12P[6]	99,5
3	LC019054-RVA/Human-tc/MMR/A23/2011/G12P[6]	99,4
4	KJ752816-RVA/Human-wt/ZAF/MRC-DPRU4090/2011/G12P[6]	99,4
5	LC374208-RVA/Human-wt/NPL/10N4594/2010/G12P[6]	99,4
6	KT919769-RVA/Human-wt/USA/VU11-12-67/2012/G12P[8]	99,2
7	AB861958-RVA/Human-tc/KEN/KDH651/2010/G12P[8]	99,2
8	KU048582-RVA/Human-wt/ITA/ME650/14/2014/G12P[8]	99,2
9	KU048581-RVA/Human-wt/ITA/PA525/14/2014/G12P[8]	99,2
10	MK417779-RVA/Human-wt/ESP/SS96099331/2018/G12P[8]	99,1
11	KU048577-RVA/Human-wt/ITA/RG178/13/2013/G12P[8]	99,1
12	MK417778-RVA/Human-wt/ESP/SS67922985/2018/G12P[8]	99,1
13	MG926778-RVA/Human-wt/MOZ/0042/2012/G12P[6]	99,0
14	MG926705-RVA/Human-wt/MOZ/0278/2012/G12P[6]	98,9
15	LC368101-RVA/Human-wt/NPL/5N0109/2005/G12P[6]	98,8
16	JQ087436-RVA/Human-tc/CHN/R588/2005/G1P[8]	97,7
17	LC173289-RVA/Human-wt/JPN/KN144/2013/G3P[8]	97,6
18	LC173257-RVA/Human-wt/JPN/KN105/2013/G3P[8]	97,6
19	LC173350-RVA/Human-xx/JPN/SP026/2013/G9P[]8	97,5
20	KX363074-RVA/Human-wt/VNM/12053_56/GXP[X]	97,5
21	LC086749-RVA/Human-wt/THA/PCB-118/2013/G1P[8]	97,4
22	LC086760-RVA/Human-wt/THA/SKT-98/2013/G1P[8]	97,3
23	**RVA/Human-wt/RWA/UFS-NGS:MRC-DPRU9995/2015/G12P[8]**	**97,3**
24	**RVA/Human-wt/RWA/UFS-NGS:MRC-DPRU8020/2015/G12P[8]**	**97,3**
25	KT694941-RVA/Human-tc/USA/Wa/1974/G1P[8]	94,4
26	RVA/Vaccine/USA/RotaTeq-WI79-9/1992/G1P7[5]	94,2
27	JX943611-RVA/Human-tc/USA/Rotarix/2009/G1P[8]	93,9
28	**RVA/Human-wt/RWA/UFS-NGS:MRC-DPRU566/2013/G9P[4]**	**88,0**
29	**RVA/Human-wt/RWA/UFS-NGS:MRC-DPRU6235/2014/G4P[4]**	**87,5**
30	HQ650118-RVA/Human-tc/USA/DS-1/1976/G2P[4]	82,7
31	DQ490537-RVA/Human-tc/JPN/AU-1/1982/G3P3[9]	82,4
32	KC571495-RVA/Human-wt/AUS/SA066/2010/G2P[4]	82,2
33	KU356642-RVA/Human-wt/BGN/M292/2013/G2P[4]	82,1
34	MH170019-RVA/Human-wt/PAK585/2016/G1P[8]	82,1
35	LC227959-RVA/Human-wt/IND/Kol-047/2013/G9P[4]	81,9
36	LC227964-RVA/Human-wt/IND/Kol-066/2013/G9P[4]	81,9
37	KP007153-RVA/Human-wt/PHI/TGO12-003/2012/G2P[4]	81,9
38	MH170018-RVA/Human-wt/PAK419/2016/G3P[4]	81,9
39	LC173352-RVA/Human-xx/JPN/SP028/2013/G2P[4]	81,8
40	LC477526-RVA/Human-wt/JPN/Tokyo18-42/2018/G2P[4]	81,8
41	MG891995-RVA/Human-wt/MOZ/0126/2013/G2P[4]	81,8
42	KU248374-RVA/Human-wt/BGN/J303/2010/G2P[4]	81,8
43	JX965141-RVA/Human/AUS/MON008/2010/G2P[4]	81,8
44	LC001994-RVA/Human-wt/JPN/B110056/2011/G2P[4]	81,8
45	MG926749-RVA/Human-wt/MOZ/0440/2013/G2P[4]	81,7
46	KY497531-RVA/Human-wt/PAK/3085/2010/G2P[4]	81,7
47	KY497553-RVA/Human-wt/PAK93/2010/G1P[6]	81,7
48	KC782521.2-RVA/Human-wt/USA/LB1562/2010/G9P[4]	81,7
49	KU714446-RVA/Human-wt/MWI/MW2-181_A/2000/G1P[8]	81,7
50	KC443633-RVA/Human-wt/AUS/CK20028/2006/G2P[4]	81,7
51	KC443765-RVA/Human-wt/AUS/CK20027/2006/G2P[4]	81,7
52	KJ752620-RVA/Human-wt/SEN/MRC-DPRU2053/2009/G8P[6]	81,7
53	KR705269-RVA/Human-wt/BEL/BE34/2006/G2P[4]	81,6
54	MF184838-RVA/Human-wt/USA/CNMC123/2011/G2P[4]	81,6
55	LC133527-RVA/Cow-tc/THA/A5-13/1988/G8P[1]	81,5
56	JX416213-RVA/Human-tc/SWE/1076/1983/G2P2A[6]	81,5
57	FN665690-RVA/Human-wt/HUN/BP1062/2004/G8P[14]	81,4
58	KC174868-RVA/Human-wt/IND/N36/2003/G10P[11]	81,4
59	LC133538-RVA/Cow-tc/THA/61A/1989/G10P[5]	81,3
60	KC174890-RVA/Human-wt/IND/N62/2003/G10P[11]	81,3
61	JN831233-RVA/Cow-wt/ZAF/1605/2007/G6P[5]	81,2
62	JN831211-RVA/Cow-wt/ZAF/1603/2007/G6P[5]	81,2
63	LC133560-RVA/Cow-tc/JPN/KK3/1983/G10P[11]	81,1
64	JN831222-RVA/Cow-wt/ZAF/1604/2007/G8P[1]	81,1
65	LC074700-RVA/Cat-tc/JPN/FRV537/2004/G6P[5]	81,1
66	KF636258-RVA/Cow/ZAF/MRC-DPRU1604/2007/G6P[1]	81,1
67	FJ347124-RVA/Guanaco-wt/ARG/1998/G8P[1]	81,0
68	MF940540-RVA/Porcine-tc/KOR/174-1/2006/G8P[7]	81,0
69	GU565043-RVA/Vaccine/USA/RotaTeq-WI79-4/1992/G6P1A[8]	81,0
70	GU565076-RVA/Vaccine/USA/RotaTeq-WI78-8/1992/G3P7[5]	81,0
71	GU565087-RVA/Vaccine/USA/RotaTeq-BrB-9/1996/G4P7[5]	81,0
72	DQ870495-RVA/Cow-tc/USA/NCDV/1967/G6P6[1]	80,9
73	KY426811-RVA/Deer-wt/SLO/D110-15/2015/G8P[14]	80,9
74	MF940597-RVA/Bovine-tc/KOR/KJ11/2006/G8P[7]	80,8
75	MF940652-RVA/Bovine-tc/KOR/KJ19-2/2006/G6P[7]	80,7

E. NSP1

	**RVA/Human-wt/RWA/UFS-NGS:MRC-DPRU6235/2014/G4P[4]**	
1	**RVA/Human-wt/RWA/UFS-NGS:MRC-DPRU6212/2014/G12P[8]**	**90,5**
2	LC086797-RVA/Human-wt/THA/SKT-138/2013/G2P[4]	90,2
3	KU059772-RVA/Human-wt/AUS/D388/2013/G3P[8]	90,2
4	LC086786-RVA/Human-wt/THA/NP-M51/2013/G2P[4]	90,2
5	LC086731-RVA/Human-wt/THA/SKT-289/2013/G3P[8]	90,1
6	LC066656-RVA/Human-wt/THA/SKT-109/2013/G1P[8]	90,0
7	LC066645-RVA/Human-wt/THA/PCB-180/2013/G1P[8]	89,9
8	KP007187-RVA/Human-wt/PHI/TGO12-012/2012/G1P[8]	89,9
9	LC173537-RVA/Human/JPN/SP025/2013/G1P[8]	89,8
10	LC469507-RVA/Human-wt/IDN/D13/2013/G3P[8]	89,8
11	LC469510-RVA/Human-wt/IDN/GRV60/2014/G1P[8]	89,8
12	KC834697-RVA/Human-wt/AUS/336190/2004/G2P[4]	88,9
13	KC834696-RVA/Human-wt/AUS/V203/2009/G2P[4]	88,6
14	KJ918866-RVA/Human-wt/HUN/ERN5635/2012/G2P[4]	88,5
15	JQ069421-RVA/Human-wt/CAN/RT125-07/2008/G2P[4]	88,2
16	KM660103-RVA/Human-wt/CMR/BA368/2010/G2P[4]	87,7
17	HQ650120-RVA/Human-tc/USA/DS-1/1976/G2P[4]	85,5
18	**RVA/Human-wt/RWA/UFS-NGS:MRC-DPRU566/2013/G9P[4]**	**79,4**
19	**RVA/Human-wt/RWA/UFS-NGS:MRC-DPRU8020/2015/G12P[8]**	**79,1**
20	**RVA/Human-wt/RWA/UFS-NGS:MRC-DPRU9995/2015/G12P[8]**	**79,1**
21	KJ752853-RVA/Human-wt/ZWE/MRC-DPRU1850/2011/G4P[8]	78,9
22	JQ988894-RVA/Human-wt/Croatia/CR2006/2006/G8P[8]	78,7
23	KC195764-RVA/Human-wt/AUS/CK00081/2007/G1P[8]	78,6
24	LC439274-RVA/Human-wt/GHA/M0094/2010/G9P[8]	78,5
25	JX185764-RVA/Human-wt/ITA/ASTI23/2007/G9P[8]	78,5
26	KP752632-RVA/Human-wt/SEN/MRC-DPRU2051/2009/G9P[8]	78,5
27	LC173562-RVA/Human/JPN/YM046/2013/G1P[8]	78,4
28	LC173555-RVA/Human/JPN/YM025/2013/G1P[8]	78,4
29	KJ559373-RVA/Human-wt/ARG/Mis864/1998/G4P[8]	78,3
30	KJ559329-RVA/Human-wt/PRY/476/2000/G4P[8]	78,3
31	KJ559070-RVA/Human-wt/ARG/Res1717/1998/G4P[8]	78,3
32	KJ626519-RVA/Human-wt/PRY/404/1999/G1P[8]	78,3
33	DQ492675-RVA/Human-wt/BGD/Dhaka16/2003/G1P[8]	78,3
34	HQ392454-RVA/Human-wt/BEL/BE00049/2009/G1P[8]	78,2
35	KJ559307-RVA/Human-wt/ARG/Tuc1662/1998/G4P[8]	78,2
36	KJ559120-RVA/Human-wt/PRY/88/1998/G4P[8]	78,2
37	KJ559199-RVA/Human-wt/PRY/358/1999/G4P[8]	78,2
38	KJ559059-RVA/Human-wt/ARG/Res1730/1998/G4P[8]	78,2
39	KJ412877-RVA/Human-wt/PRY/1256A/2009/G3G4P[9]	78,2
40	LC368091-RVA/Human-wt/NPL/5N0014/2005/G12P[6]	78,2
41	HQ657144-RVA/Human-wt/ZAF/3133WC/2009/G12P[4]	78,2
42	KC193618-RVA/Human-wt/Bel/BE00061/2000/G1P[8]	78,1
43	KJ752411-RVA/Human-wt/ZAF/MRC-DPRU400/2003/G1P[8]	78,1
44	EF990709-RVA/Human-wt/BEL/B3458/2003/G9P[8]	78,1
45	DQ146655-RVA/Human-wt/BGD/Dhaka25/2002/G12P[8]	78,1
46	KJ559132-RVA/Human-wt/ARG/Ush2037/1999/G4P[8]	78,0
47	JX195069-RVA/Human-wt/ITA/AV21/2010/G9P[8]	78,0
48	KU048672-RVA/Human-wt/ITA/RG176/13/2013/G12P[8]	78,0
49	MN067042-RVA/Human-wt/IND/CMC_00036/2010/G1P[8]	77,9
50	KU048673-RVA/Human-wt/ITA/RG177/13/2013/G12P[8]	77,9
51	KX655534-RVA/Cow-wt/UGA/BUW-14-A035/2014/G12P[8]	77,9
52	KU048678-RVA/Human-wt/ITA/PA525/14/2014/G12P[8]	77,8
53	KM116052-RVA/Human-wt/USA/2013774166/2013/G12P[8]	77,8
54	MH171413-RVA/Human-wt/ESP/VI4816626/2012/G12P[8]	77,8
55	KU048659-RVA/Human-wt/ITA/PA93/12/2012/G12P[8]	77,8
56	LC374165-RVA/Human-wt/NPL/09N3808/2009/G12P[6]	77,7
57	MG181431-RVA/Human-wt/MWI/MW2-1070/2004/G1P[8]	77,7
58	AB861962-RVA/Human-tc/KEN/KDH651/2010/G12P[8]	77,7
59	KU048662-RVA/Human-wt/ITA/PA144/12/2012/G12P[8]	77,7
60	MH171402-RVA/Human-wt/ESP/SS257451/2012/G12P[8]	77,6
61	LC374187-RVA/Human-wt/NPL/10N4001/2010/G12P[6]	77,6
62	KU048677-RVA/Human-wt/ITA/PA417/14/2014/G12P[8]	77,5
63	LC374198-RVA/Human-wt/NPL/10N4155/2010/G12P[6]	77,4
64	KU861385-RVA/Human-tc/USA/Wa/1974/G1P[8]	73,5
65	AB792641-RVA/Human-tc/JPN/AU-1/1982/G3P[9]	39,6

	**RVA/Human-wt/RWA/UFS-NGS:MRC-DPRU566/2013/G9P[4]**	
1	MN067042-RVA/Human-wt/IND/CMC_00036/2010/G1P[8]	97,7
2	HQ657144-RVA/Human-wt/ZAF/3133WC/2009/G12P[4]	97,4
3	KU048672-RVA/Human-wt/ITA/RG176/13/2013/G12P[8]	97,3
4	KX655534-RVA/Cow-wt/UGA/BUW-14-A035/2014/G12P[8]	97,3
5	KM116052-RVA/Human-wt/USA/2013774166/2013/G12P[8]	97,3
6	KU048677-RVA/Human-wt/ITA/PA417/14/2014/G12P[8]	97,3
7	KU048673-RVA/Human-wt/ITA/RG177/13/2013/G12P[8]	97,2
8	DQ146655-RVA/Human-wt/BGD/Dhaka25/2002/G12P[8]	97,2
9	MH171413-RVA/Human-wt/ESP/VI4816626/2012/G12P[8]	97,2
10	KU048659-RVA/Human-wt/ITA/PA93/12/2012/G12P[8]	97,2
11	KU048662-RVA/Human-wt/ITA/PA144/12/2012/G12P[8]	97,1
12	MH171402-RVA/Human-wt/ESP/SS257451/2012/G12P[8]	97,1
13	AB861962-RVA/Human-tc/KEN/KDH651/2010/G12P[8]	97,1
14	LC374187-RVA/Human-wt/NPL/10N4001/2010/G12P[6]	97,0
15	KU048678-RVA/Human-wt/ITA/PA525/14/2014/G12P[8]	97,0
16	LC374198-RVA/Human-wt/NPL/10N4155/2010/G12P[6]	96,9
17	LC374165-RVA/Human-wt/NPL/09N3808/2009/G12P[6]	96,9
18	LC368091-RVA/Human-wt/NPL/5N0014/2005/G12P[6]	96,8
19	DQ492675-RVA/Human-wt/BGD/Dhaka16/2003/G1P[8]	96,8
20	EF990709-RVA/Human-wt/BEL/B3458/2003/G9P[8]	96,7
21	LC173562-RVA/Human/JPN/YM046/2013/G1P[8]	96,6
22	LC173555-RVA/Human/JPN/YM025/2013/G1P[8]	96,6
23	KJ559307-RVA/Human-wt/ARG/Tuc1662/1998/G4P[8]	95,6
24	KJ559373-RVA/Human-wt/ARG/Mis864/1998/G4P[8]	95,5
25	KJ559070-RVA/Human-wt/ARG/Res1717/1998/G4P[8]	95,5
26	KJ559329-RVA/Human-wt/PRY/476/2000/G4P[8]	95,5
27	KJ559120-RVA/Human-wt/PRY/88/1998/G4P[8]	95,5
28	KJ626519-RVA/Human-wt/PRY/404/1999/G1P[8]	95,4
29	KJ559059-RVA/Human-wt/ARG/Res1730/1998/G4P[8]	95,4
30	KJ412877-RVA/Human-wt/PRY/1256A/2009/G3G4P[9]	95,4
31	KC193618-RVA/Human-wt/Bel/BE00061/2000/G1P[8]	95,3
32	KJ559132-RVA/Human-wt/ARG/Ush2037/1999/G4P[8]	95,3
33	KJ559199-RVA/Human-wt/PRY/358/1999/G4P[8]	95,3
34	KJ752411-RVA/Human-wt/ZAF/MRC-DPRU400/2003/G1P[8]	94,9
35	MG181431-RVA/Human-wt/MWI/MW2-1070/2004/G1P[8]	94,9
36	LC439274-RVA/Human-wt/GHA/M0094/2010/G9P[8]	94,8
37	KP752632-RVA/Human-wt/SEN/MRC-DPRU2051/2009/G9P[8]	94,8
38	JQ988894-RVA/Human-wt/Croatia/CR2006/2006/G8P[8]	94,6
39	JX195069-RVA/Human-wt/ITA/AV21/2010/G9P[8]	94,5
40	KC195764-RVA/Human-wt/AUS/CK00081/2007/G1P[8]	94,4
41	JX185764-RVA/Human-wt/ITA/ASTI23/2007/G9P[8]	94,4
42	KJ752853-RVA/Human-wt/ZWE/MRC-DPRU1850/2011/G4P[8]	94,3
43	HQ392454-RVA/Human-wt/BEL/BE00049/2009/G1P[8]	94,2
44	**RVA/Human-wt/RWA/UFS-NGS:MRC-DPRU8020/2015/G12P[8]**	**94,1**
45	**RVA/Human-wt/RWA/UFS-NGS:MRC-DPRU9995/2015/G12P[8]**	**94,1**
46	KU861385-RVA/Human-tc/USA/Wa/1974/G1P[8]	83,2
47	**RVA/Human-wt/RWA/UFS-NGS:MRC-DPRU6235/2014/G4P[4]**	**79,4**
48	HQ650120-RVA/Human-tc/USA/DS-1/1976/G2P[4]	70,1
49	JQ069421-RVA/Human-wt/CAN/RT125-07/2008/G2P[4]	69,6
50	KJ918866-RVA/Human-wt/HUN/ERN5635/2012/G2P[4]	69,6
51	LC469507-RVA/Human-wt/IDN/D13/2013/G3P[8]	69,2
52	KM660103-RVA/Human-wt/CMR/BA368/2010/G2P[4]	69,2
53	KC834696-RVA/Human-wt/AUS/V203/2009/G2P[4]	69,2
54	KC834697-RVA/Human-wt/AUS/336190/2004/G2P[4]	69,2
55	LC086731-RVA/Human-wt/THA/SKT-289/2013/G3P[8]	69,1
56	LC066656-RVA/Human-wt/THA/SKT-109/2013/G1P[8]	69,1
57	KU059772-RVA/Human-wt/AUS/D388/2013/G3P[8]	69,1
58	**RVA/Human-wt/RWA/UFS-NGS:MRC-DPRU6212/2014/G12P[8]**	**69,1**
59	KP007187-RVA/Human-wt/PHI/TGO12-012/2012/G1P[8]	69,0
60	LC066645-RVA/Human-wt/THA/PCB-180/2013/G1P[8]	69,0
61	LC086797-RVA/Human-wt/THA/SKT-138/2013/G2P[4]	68,9
62	LC469510-RVA/Human-wt/IDN/GRV60/2014/G1P[8]	68,9
63	LC086786-RVA/Human-wt/THA/NP-M51/2013/G2P[4]	68,8
64	LC173537-RVA/Human/JPN/SP025/2013/G1P[8]	68,8
65	AB792641-RVA/Human-tc/JPN/AU-1/1982/G3P[9]	41,1

	**RVA/Human-wt/RWA/UFS-NGS:MRC-DPRU8020/2015/G12P[8]**	
1	**RVA/Human-wt/RWA/UFS-NGS:MRC-DPRU9995/2015/G12P[8]**	**99,9**
2	KJ752853-RVA/Human-wt/ZWE/MRC-DPRU1850/2011/G4P[8]	99,2
3	JQ988894-RVA/Human-wt/Croatia/CR2006/2006/G8P[8]	99,1
4	KC195764-RVA/Human-wt/AUS/CK00081/2007/G1P[8]	98,9
5	JX185764-RVA/Human-wt/ITA/ASTI23/2007/G9P[8]	98,9
6	HQ392454-RVA/Human-wt/BEL/BE00049/2009/G1P[8]	98,5
7	KP752632-RVA/Human-wt/SEN/MRC-DPRU2051/2009/G9P[8]	98,3
8	LC439274-RVA/Human-wt/GHA/M0094/2010/G9P[8]	98,3
9	KJ559373-RVA/Human-wt/ARG/Mis864/1998/G4P[8]	98,3
10	KJ559070-RVA/Human-wt/ARG/Res1717/1998/G4P[8]	98,3
11	KJ559329-RVA/Human-wt/PRY/476/2000/G4P[8]	98,2
12	KJ559307-RVA/Human-wt/ARG/Tuc1662/1998/G4P[8]	98,2
13	KJ559120-RVA/Human-wt/PRY/88/1998/G4P[8]	98,2
14	KJ412877-RVA/Human-wt/PRY/1256A/2009/G3G4P[9]	98,2
15	JX195069-RVA/Human-wt/ITA/AV21/2010/G9P[8]	98,1
16	KJ626519-RVA/Human-wt/PRY/404/1999/G1P[8]	98,1
17	KJ559199-RVA/Human-wt/PRY/358/1999/G4P[8]	98,1
18	KC193618-RVA/Human-wt/Bel/BE00061/2000/G1P[8]	98,0
19	KJ559059-RVA/Human-wt/ARG/Res1730/1998/G4P[8]	98,0
20	KJ559132-RVA/Human-wt/ARG/Ush2037/1999/G4P[8]	98,0
21	KJ752411-RVA/Human-wt/ZAF/MRC-DPRU400/2003/G1P[8]	97,8
22	MG181431-RVA/Human-wt/MWI/MW2-1070/2004/G1P[8]	97,3
23	LC173562-RVA/Human/JPN/YM046/2013/G1P[8]	96,3
24	LC173555-RVA/Human/JPN/YM025/2013/G1P[8]	96,3
25	DQ146655-RVA/Human-wt/BGD/Dhaka25/2002/G12P[8]	96,2
26	DQ492675-RVA/Human-wt/BGD/Dhaka16/2003/G1P[8]	96,0
27	EF990709-RVA/Human-wt/BEL/B3458/2003/G9P[8]	95,8
28	KU048672-RVA/Human-wt/ITA/RG176/13/2013/G12P[8]	95,8
29	HQ657144-RVA/Human-wt/ZAF/3133WC/2009/G12P[4]	95,8
30	LC374165-RVA/Human-wt/NPL/09N3808/2009/G12P[6]	95,8
31	LC374187-RVA/Human-wt/NPL/10N4001/2010/G12P[6]	95,8
32	KU048673-RVA/Human-wt/ITA/RG177/13/2013/G12P[8]	95,7
33	LC368091-RVA/Human-wt/NPL/5N0014/2005/G12P[6]	95,7
34	MN067042-RVA/Human-wt/IND/CMC_00036/2010/G1P[8]	95,7
35	LC374198-RVA/Human-wt/NPL/10N4155/2010/G12P[6]	95,7
36	AB861962-RVA/Human-tc/KEN/KDH651/2010/G12P[8]	95,6
37	KU048678-RVA/Human-wt/ITA/PA525/14/2014/G12P[8]	95,5
38	KX655534-RVA/Cow-wt/UGA/BUW-14-A035/2014/G12P[8]	95,5
39	KM116052-RVA/Human-wt/USA/2013774166/2013/G12P[8]	95,5
40	MH171413-RVA/Human-wt/ESP/VI4816626/2012/G12P[8]	95,5
41	KU048659-RVA/Human-wt/ITA/PA93/12/2012/G12P[8]	95,5
42	KU048662-RVA/Human-wt/ITA/PA144/12/2012/G12P[8]	95,4
43	MH171402-RVA/Human-wt/ESP/SS257451/2012/G12P[8]	95,4
44	KU048677-RVA/Human-wt/ITA/PA417/14/2014/G12P[8]	95,4
45	**RVA/Human-wt/RWA/UFS-NGS:MRC-DPRU566/2013/G9P[4]**	**94,1**
46	KU861385-RVA/Human-tc/USA/Wa/1974/G1P[8]	82,1
47	**RVA/Human-wt/RWA/UFS-NGS:MRC-DPRU6235/2014/G4P[4]**	**79,1**
48	HQ650120-RVA/Human-tc/USA/DS-1/1976/G2P[4]	68,4
49	KM660103-RVA/Human-wt/CMR/BA368/2010/G2P[4]	65,6
50	**RVA/Human-wt/RWA/UFS-NGS:MRC-DPRU6212/2014/G12P[8]**	**65,6**
51	LC469507-RVA/Human-wt/IDN/D13/2013/G3P[8]	65,6
52	KC834696-RVA/Human-wt/AUS/V203/2009/G2P[4]	65,6
53	LC086731-RVA/Human-wt/THA/SKT-289/2013/G3P[8]	65,5
54	LC066656-RVA/Human-wt/THA/SKT-109/2013/G1P[8]	65,5
55	KU059772-RVA/Human-wt/AUS/D388/2013/G3P[8]	65,5
56	KP007187-RVA/Human-wt/PHI/TGO12-012/2012/G1P[8]	65,4
57	LC066645-RVA/Human-wt/THA/PCB-180/2013/G1P[8]	65,4
58	JQ069421-RVA/Human-wt/CAN/RT125-07/2008/G2P[4]	65,3
59	KJ918866-RVA/Human-wt/HUN/ERN5635/2012/G2P[4]	65,3
60	LC469510-RVA/Human-wt/IDN/GRV60/2014/G1P[8]	65,2
61	KC834697-RVA/Human-wt/AUS/336190/2004/G2P[4]	65,2
62	LC173537-RVA/Human/JPN/SP025/2013/G1P[8]	65,2
63	LC086797-RVA/Human-wt/THA/SKT-138/2013/G2P[4]	65,1
64	LC086786-RVA/Human-wt/THA/NP-M51/2013/G2P[4]	65,0
65	AB792641-RVA/Human-tc/JPN/AU-1/1982/G3P[9]	43,4

	**RVA/Human-wt/RWA/UFS-NGS:MRC-DPRU9995/2015/G12P[8]**	
1	**RVA/Human-wt/RWA/UFS-NGS:MRC-DPRU8020/2015/G12P[8]**	**99,9**
2	KJ752853-RVA/Human-wt/ZWE/MRC-DPRU1850/2011/G4P[8]	99,1
3	JQ988894-RVA/Human-wt/Croatia/CR2006/2006/G8P[8]	99,0
4	KC195764-RVA/Human-wt/AUS/CK00081/2007/G1P[8]	98,8
5	JX185764-RVA/Human-wt/ITA/ASTI23/2007/G9P[8]	98,8
6	HQ392454-RVA/Human-wt/BEL/BE00049/2009/G1P[8]	98,5
7	KP752632-RVA/Human-wt/SEN/MRC-DPRU2051/2009/G9P[8]	98,3
8	LC439274-RVA/Human-wt/GHA/M0094/2010/G9P[8]	98,3
9	KJ559373-RVA/Human-wt/ARG/Mis864/1998/G4P[8]	98,2
10	KJ559070-RVA/Human-wt/ARG/Res1717/1998/G4P[8]	98,2
11	KJ559329-RVA/Human-wt/PRY/476/2000/G4P[8]	98,1
12	KJ559307-RVA/Human-wt/ARG/Tuc1662/1998/G4P[8]	98,1
13	KJ559120-RVA/Human-wt/PRY/88/1998/G4P[8]	98,1
14	KJ412877-RVA/Human-wt/PRY/1256A/2009/G3G4P[9]	98,1
15	JX195069-RVA/Human-wt/ITA/AV21/2010/G9P[8]	98,0
16	KJ626519-RVA/Human-wt/PRY/404/1999/G1P[8]	98,0
17	KJ559199-RVA/Human-wt/PRY/358/1999/G4P[8]	98,0
18	KC193618-RVA/Human-wt/Bel/BE00061/2000/G1P[8]	98,0
19	KJ559059-RVA/Human-wt/ARG/Res1730/1998/G4P[8]	98,0
20	KJ559132-RVA/Human-wt/ARG/Ush2037/1999/G4P[8]	98,0
21	KJ752411-RVA/Human-wt/ZAF/MRC-DPRU400/2003/G1P[8]	97,8
22	MG181431-RVA/Human-wt/MWI/MW2-1070/2004/G1P[8]	97,3
23	LC173562-RVA/Human/JPN/YM046/2013/G1P[8]	96,2
24	LC173555-RVA/Human/JPN/YM025/2013/G1P[8]	96,2
25	DQ146655-RVA/Human-wt/BGD/Dhaka25/2002/G12P[8]	96,1
26	DQ492675-RVA/Human-wt/BGD/Dhaka16/2003/G1P[8]	96,0
27	EF990709-RVA/Human-wt/BEL/B3458/2003/G9P[8]	95,7
28	KU048672-RVA/Human-wt/ITA/RG176/13/2013/G12P[8]	95,7
29	HQ657144-RVA/Human-wt/ZAF/3133WC/2009/G12P[4]	95,7
30	LC374165-RVA/Human-wt/NPL/09N3808/2009/G12P[6]	95,7
31	LC374187-RVA/Human-wt/NPL/10N4001/2010/G12P[6]	95,7
32	KU048673-RVA/Human-wt/ITA/RG177/13/2013/G12P[8]	95,7
33	LC368091-RVA/Human-wt/NPL/5N0014/2005/G12P[6]	95,7
34	MN067042-RVA/Human-wt/IND/CMC_00036/2010/G1P[8]	95,6
35	LC374198-RVA/Human-wt/NPL/10N4155/2010/G12P[6]	95,6
36	AB861962-RVA/Human-tc/KEN/KDH651/2010/G12P[8]	95,5
37	KU048678-RVA/Human-wt/ITA/PA525/14/2014/G12P[8]	95,4
38	KX655534-RVA/Cow-wt/UGA/BUW-14-A035/2014/G12P[8]	95,4
39	KM116052-RVA/Human-wt/USA/2013774166/2013/G12P[8]	95,4
40	MH171413-RVA/Human-wt/ESP/VI4816626/2012/G12P[8]	95,4
41	KU048659-RVA/Human-wt/ITA/PA93/12/2012/G12P[8]	95,4
42	KU048662-RVA/Human-wt/ITA/PA144/12/2012/G12P[8]	95,3
43	MH171402-RVA/Human-wt/ESP/SS257451/2012/G12P[8]	95,3
44	KU048677-RVA/Human-wt/ITA/PA417/14/2014/G12P[8]	95,3
45	**RVA/Human-wt/RWA/UFS-NGS:MRC-DPRU566/2013/G9P[4]**	**94,1**
46	KU861385-RVA/Human-tc/USA/Wa/1974/G1P[8]	82,0
47	**RVA/Human-wt/RWA/UFS-NGS:MRC-DPRU6235/2014/G4P[4]**	**79,1**
48	HQ650120-RVA/Human-tc/USA/DS-1/1976/G2P[4]	68,3
49	KM660103-RVA/Human-wt/CMR/BA368/2010/G2P[4]	65,6
50	**RVA/Human-wt/RWA/UFS-NGS:MRC-DPRU6212/2014/G12P[8]**	**65,6**
51	LC469507-RVA/Human-wt/IDN/D13/2013/G3P[8]	65,5
52	KC834696-RVA/Human-wt/AUS/V203/2009/G2P[4]	65,5
53	LC086731-RVA/Human-wt/THA/SKT-289/2013/G3P[8]	65,5
54	LC066656-RVA/Human-wt/THA/SKT-109/2013/G1P[8]	65,5
55	KU059772-RVA/Human-wt/AUS/D388/2013/G3P[8]	65,5
56	KP007187-RVA/Human-wt/PHI/TGO12-012/2012/G1P[8]	65,4
57	LC066645-RVA/Human-wt/THA/PCB-180/2013/G1P[8]	65,4
58	JQ069421-RVA/Human-wt/CAN/RT125-07/2008/G2P[4]	65,3
59	KJ918866-RVA/Human-wt/HUN/ERN5635/2012/G2P[4]	65,3
60	LC469510-RVA/Human-wt/IDN/GRV60/2014/G1P[8]	65,2
61	KC834697-RVA/Human-wt/AUS/336190/2004/G2P[4]	65,2
62	LC173537-RVA/Human/JPN/SP025/2013/G1P[8]	65,1
63	LC086797-RVA/Human-wt/THA/SKT-138/2013/G2P[4]	65,1
64	LC086786-RVA/Human-wt/THA/NP-M51/2013/G2P[4]	64,9
65	AB792641-RVA/Human-tc/JPN/AU-1/1982/G3P[9]	43,3

	**RVA/Human-wt/RWA/UFS-NGS:MRC-DPRU6212/2014/G12P[8]**	
1	KU059772-RVA/Human-wt/AUS/D388/2013/G3P[8]	99,2
2	LC086786-RVA/Human-wt/THA/NP-M51/2013/G2P[4]	99,2
3	LC066656-RVA/Human-wt/THA/SKT-109/2013/G1P[8]	99,2
4	LC086731-RVA/Human-wt/THA/SKT-289/2013/G3P[8]	99,2
5	LC086797-RVA/Human-wt/THA/SKT-138/2013/G2P[4]	99,2
6	LC066645-RVA/Human-wt/THA/PCB-180/2013/G1P[8]	99,1
7	KP007187-RVA/Human-wt/PHI/TGO12-012/2012/G1P[8]	99,1
8	LC173537-RVA/Human/JPN/SP025/2013/G1P[8]	99,0
9	LC469507-RVA/Human-wt/IDN/D13/2013/G3P[8]	99,0
10	LC469510-RVA/Human-wt/IDN/GRV60/2014/G1P[8]	99,0
11	KC834697-RVA/Human-wt/AUS/336190/2004/G2P[4]	97,5
12	KC834696-RVA/Human-wt/AUS/V203/2009/G2P[4]	97,1
13	JQ069421-RVA/Human-wt/CAN/RT125-07/2008/G2P[4]	97,1
14	KJ918866-RVA/Human-wt/HUN/ERN5635/2012/G2P[4]	97,0
15	KM660103-RVA/Human-wt/CMR/BA368/2010/G2P[4]	96,5
16	HQ650120-RVA/Human-tc/USA/DS-1/1976/G2P[4]	92,6
17	**RVA/Human-wt/RWA/UFS-NGS:MRC-DPRU6235/2014/G4P[4]**	**90,5**
18	**RVA/Human-wt/RWA/UFS-NGS:MRC-DPRU566/2013/G9P[4]**	**69,1**
19	KU861385-RVA/Human-tc/USA/Wa/1974/G1P[8]	67,4
20	HQ657144-RVA/Human-wt/ZAF/3133WC/2009/G12P[4]	67,2
21	KU048677-RVA/Human-wt/ITA/PA417/14/2014/G12P[8]	67,1
22	KM116052-RVA/Human-wt/USA/2013774166/2013/G12P[8]	67,1
23	KU048678-RVA/Human-wt/ITA/PA525/14/2014/G12P[8]	67,0
24	KX655534-RVA/Cow-wt/UGA/BUW-14-A035/2014/G12P[8]	66,9
25	MN067042-RVA/Human-wt/IND/CMC_00036/2010/G1P[8]	66,9
26	KU048672-RVA/Human-wt/ITA/RG176/13/2013/G12P[8]	66,9
27	EF990709-RVA/Human-wt/BEL/B3458/2003/G9P[8]	66,9
28	MH171413-RVA/Human-wt/ESP/VI4816626/2012/G12P[8]	66,8
29	KU048659-RVA/Human-wt/ITA/PA93/12/2012/G12P[8]	66,8
30	KU048673-RVA/Human-wt/ITA/RG177/13/2013/G12P[8]	66,8
31	AB861962-RVA/Human-tc/KEN/KDH651/2010/G12P[8]	66,8
32	LC368091-RVA/Human-wt/NPL/5N0014/2005/G12P[6]	66,8
33	KU048662-RVA/Human-wt/ITA/PA144/12/2012/G12P[8]	66,7
34	LC374187-RVA/Human-wt/NPL/10N4001/2010/G12P[6]	66,7
35	MH171402-RVA/Human-wt/ESP/SS257451/2012/G12P[8]	66,7
36	DQ146655-RVA/Human-wt/BGD/Dhaka25/2002/G12P[8]	66,6
37	DQ492675-RVA/Human-wt/BGD/Dhaka16/2003/G1P[8]	66,6
38	LC173562-RVA/Human/JPN/YM046/2013/G1P[8]	66,6
39	LC173555-RVA/Human/JPN/YM025/2013/G1P[8]	66,6
40	LC374198-RVA/Human-wt/NPL/10N4155/2010/G12P[6]	66,5
41	LC374165-RVA/Human-wt/NPL/09N3808/2009/G12P[6]	66,4
42	KJ559373-RVA/Human-wt/ARG/Mis864/1998/G4P[8]	66,0
43	KJ559329-RVA/Human-wt/PRY/476/2000/G4P[8]	66,0
44	KJ559307-RVA/Human-wt/ARG/Tuc1662/1998/G4P[8]	66,0
45	KJ559070-RVA/Human-wt/ARG/Res1717/1998/G4P[8]	66,0
46	KJ626519-RVA/Human-wt/PRY/404/1999/G1P[8]	66,0
47	KJ559120-RVA/Human-wt/PRY/88/1998/G4P[8]	65,9
48	KJ559199-RVA/Human-wt/PRY/358/1999/G4P[8]	65,9
49	KJ559059-RVA/Human-wt/ARG/Res1730/1998/G4P[8]	65,8
50	KJ412877-RVA/Human-wt/PRY/1256A/2009/G3G4P[9]	65,8
51	KC193618-RVA/Human-wt/Bel/BE00061/2000/G1P[8]	65,8
52	KJ559132-RVA/Human-wt/ARG/Ush2037/1999/G4P[8]	65,7
53	JX185764-RVA/Human-wt/ITA/ASTI23/2007/G9P[8]	65,6
54	**RVA/Human-wt/RWA/UFS-NGS:MRC-DPRU8020/2015/G12P[8]**	**65,6**
55	**RVA/Human-wt/RWA/UFS-NGS:MRC-DPRU9995/2015/G12P[8]**	**65,6**
56	LC439274-RVA/Human-wt/GHA/M0094/2010/G9P[8]	65,6
57	KJ752411-RVA/Human-wt/ZAF/MRC-DPRU400/2003/G1P[8]	65,5
58	KC195764-RVA/Human-wt/AUS/CK00081/2007/G1P[8]	65,5
59	KP752632-RVA/Human-wt/SEN/MRC-DPRU2051/2009/G9P[8]	65,5
60	JQ988894-RVA/Human-wt/Croatia/CR2006/2006/G8P[8]	65,4
61	HQ392454-RVA/Human-wt/BEL/BE00049/2009/G1P[8]	65,3
62	MG181431-RVA/Human-wt/MWI/MW2-1070/2004/G1P[8]	65,3
63	KJ752853-RVA/Human-wt/ZWE/MRC-DPRU1850/2011/G4P[8]	65,3
64	JX195069-RVA/Human-wt/ITA/AV21/2010/G9P[8]	65,2
65	AB792641-RVA/Human-tc/JPN/AU-1/1982/G3P[9]	40,8

F. NSP2

	**RVA/Human-wt/RWA/UFS-NGS:MRC-DPRU6235/2014/G4P[4]**	
1	**RVA/Human-wt/RWA/UFS-NGS:MRC-DPRU6212/2014/G12P[8]**	**90,5**
2	LC086797-RVA/Human-wt/THA/SKT-138/2013/G2P[4]	90,2
3	KU059772-RVA/Human-wt/AUS/D388/2013/G3P[8]	90,2
4	LC086786-RVA/Human-wt/THA/NP-M51/2013/G2P[4]	90,2
5	LC086731-RVA/Human-wt/THA/SKT-289/2013/G3P[8]	90,1
6	LC066656-RVA/Human-wt/THA/SKT-109/2013/G1P[8]	90,0
7	LC066645-RVA/Human-wt/THA/PCB-180/2013/G1P[8]	89,9
8	KP007187-RVA/Human-wt/PHI/TGO12-012/2012/G1P[8]	89,9
9	LC173537-RVA/Human/JPN/SP025/2013/G1P[8]	89,8
10	LC469507-RVA/Human-wt/IDN/D13/2013/G3P[8]	89,8
11	LC469510-RVA/Human-wt/IDN/GRV60/2014/G1P[8]	89,8
12	KC834697-RVA/Human-wt/AUS/336190/2004/G2P[4]	88,9
13	KC834696-RVA/Human-wt/AUS/V203/2009/G2P[4]	88,6
14	KJ918866-RVA/Human-wt/HUN/ERN5635/2012/G2P[4]	88,5
15	JQ069421-RVA/Human-wt/CAN/RT125-07/2008/G2P[4]	88,2
16	KM660103-RVA/Human-wt/CMR/BA368/2010/G2P[4]	87,7
17	HQ650120-RVA/Human-tc/USA/DS-1/1976/G2P[4]	85,5
18	**RVA/Human-wt/RWA/UFS-NGS:MRC-DPRU566/2013/G9P[4]**	**79,4**
19	**RVA/Human-wt/RWA/UFS-NGS:MRC-DPRU8020/2015/G12P[8]**	**79,1**
20	**RVA/Human-wt/RWA/UFS-NGS:MRC-DPRU9995/2015/G12P[8]**	**79,1**
21	KJ752853-RVA/Human-wt/ZWE/MRC-DPRU1850/2011/G4P[8]	78,9
22	JQ988894-RVA/Human-wt/Croatia/CR2006/2006/G8P[8]	78,7
23	KC195764-RVA/Human-wt/AUS/CK00081/2007/G1P[8]	78,6
24	LC439274-RVA/Human-wt/GHA/M0094/2010/G9P[8]	78,5
25	JX185764-RVA/Human-wt/ITA/ASTI23/2007/G9P[8]	78,5
26	KP752632-RVA/Human-wt/SEN/MRC-DPRU2051/2009/G9P[8]	78,5
27	LC173562-RVA/Human/JPN/YM046/2013/G1P[8]	78,4
28	LC173555-RVA/Human/JPN/YM025/2013/G1P[8]	78,4
29	KJ559373-RVA/Human-wt/ARG/Mis864/1998/G4P[8]	78,3
30	KJ559329-RVA/Human-wt/PRY/476/2000/G4P[8]	78,3
31	KJ559070-RVA/Human-wt/ARG/Res1717/1998/G4P[8]	78,3
32	KJ626519-RVA/Human-wt/PRY/404/1999/G1P[8]	78,3
33	DQ492675-RVA/Human-wt/BGD/Dhaka16/2003/G1P[8]	78,3
34	HQ392454-RVA/Human-wt/BEL/BE00049/2009/G1P[8]	78,2
35	KJ559307-RVA/Human-wt/ARG/Tuc1662/1998/G4P[8]	78,2
36	KJ559120-RVA/Human-wt/PRY/88/1998/G4P[8]	78,2
37	KJ559199-RVA/Human-wt/PRY/358/1999/G4P[8]	78,2
38	KJ559059-RVA/Human-wt/ARG/Res1730/1998/G4P[8]	78,2
39	KJ412877-RVA/Human-wt/PRY/1256A/2009/G3G4P[9]	78,2
40	LC368091-RVA/Human-wt/NPL/5N0014/2005/G12P[6]	78,2
41	HQ657144-RVA/Human-wt/ZAF/3133WC/2009/G12P[4]	78,2
42	KC193618-RVA/Human-wt/Bel/BE00061/2000/G1P[8]	78,1
43	KJ752411-RVA/Human-wt/ZAF/MRC-DPRU400/2003/G1P[8]	78,1
44	EF990709-RVA/Human-wt/BEL/B3458/2003/G9P[8]	78,1
45	DQ146655-RVA/Human-wt/BGD/Dhaka25/2002/G12P[8]	78,1
46	KJ559132-RVA/Human-wt/ARG/Ush2037/1999/G4P[8]	78,0
47	JX195069-RVA/Human-wt/ITA/AV21/2010/G9P[8]	78,0
48	KU048672-RVA/Human-wt/ITA/RG176/13/2013/G12P[8]	78,0
49	MN067042-RVA/Human-wt/IND/CMC_00036/2010/G1P[8]	77,9
50	KU048673-RVA/Human-wt/ITA/RG177/13/2013/G12P[8]	77,9
51	KX655534-RVA/Cow-wt/UGA/BUW-14-A035/2014/G12P[8]	77,9
52	KU048678-RVA/Human-wt/ITA/PA525/14/2014/G12P[8]	77,8
53	KM116052-RVA/Human-wt/USA/2013774166/2013/G12P[8]	77,8
54	MH171413-RVA/Human-wt/ESP/VI4816626/2012/G12P[8]	77,8
55	KU048659-RVA/Human-wt/ITA/PA93/12/2012/G12P[8]	77,8
56	LC374165-RVA/Human-wt/NPL/09N3808/2009/G12P[6]	77,7
57	MG181431-RVA/Human-wt/MWI/MW2-1070/2004/G1P[8]	77,7
58	AB861962-RVA/Human-tc/KEN/KDH651/2010/G12P[8]	77,7
59	KU048662-RVA/Human-wt/ITA/PA144/12/2012/G12P[8]	77,7
60	MH171402-RVA/Human-wt/ESP/SS257451/2012/G12P[8]	77,6
61	LC374187-RVA/Human-wt/NPL/10N4001/2010/G12P[6]	77,6
62	KU048677-RVA/Human-wt/ITA/PA417/14/2014/G12P[8]	77,5
63	LC374198-RVA/Human-wt/NPL/10N4155/2010/G12P[6]	77,4
64	KU861385-RVA/Human-tc/USA/Wa/1974/G1P[8]	73,5
65	AB792641-RVA/Human-tc/JPN/AU-1/1982/G3P[9]	39,6

	**RVA/Human-wt/RWA/UFS-NGS:MRC-DPRU566/2013/G9P[4]**	
1	MN067042-RVA/Human-wt/IND/CMC_00036/2010/G1P[8]	97,7
2	HQ657144-RVA/Human-wt/ZAF/3133WC/2009/G12P[4]	97,4
3	KU048672-RVA/Human-wt/ITA/RG176/13/2013/G12P[8]	97,3
4	KX655534-RVA/Cow-wt/UGA/BUW-14-A035/2014/G12P[8]	97,3
5	KM116052-RVA/Human-wt/USA/2013774166/2013/G12P[8]	97,3
6	KU048677-RVA/Human-wt/ITA/PA417/14/2014/G12P[8]	97,3
7	KU048673-RVA/Human-wt/ITA/RG177/13/2013/G12P[8]	97,2
8	DQ146655-RVA/Human-wt/BGD/Dhaka25/2002/G12P[8]	97,2
9	MH171413-RVA/Human-wt/ESP/VI4816626/2012/G12P[8]	97,2
10	KU048659-RVA/Human-wt/ITA/PA93/12/2012/G12P[8]	97,2
11	KU048662-RVA/Human-wt/ITA/PA144/12/2012/G12P[8]	97,1
12	MH171402-RVA/Human-wt/ESP/SS257451/2012/G12P[8]	97,1
13	AB861962-RVA/Human-tc/KEN/KDH651/2010/G12P[8]	97,1
14	LC374187-RVA/Human-wt/NPL/10N4001/2010/G12P[6]	97,0
15	KU048678-RVA/Human-wt/ITA/PA525/14/2014/G12P[8]	97,0
16	LC374198-RVA/Human-wt/NPL/10N4155/2010/G12P[6]	96,9
17	LC374165-RVA/Human-wt/NPL/09N3808/2009/G12P[6]	96,9
18	LC368091-RVA/Human-wt/NPL/5N0014/2005/G12P[6]	96,8
19	DQ492675-RVA/Human-wt/BGD/Dhaka16/2003/G1P[8]	96,8
20	EF990709-RVA/Human-wt/BEL/B3458/2003/G9P[8]	96,7
21	LC173562-RVA/Human/JPN/YM046/2013/G1P[8]	96,6
22	LC173555-RVA/Human/JPN/YM025/2013/G1P[8]	96,6
23	KJ559307-RVA/Human-wt/ARG/Tuc1662/1998/G4P[8]	95,6
24	KJ559373-RVA/Human-wt/ARG/Mis864/1998/G4P[8]	95,5
25	KJ559070-RVA/Human-wt/ARG/Res1717/1998/G4P[8]	95,5
26	KJ559329-RVA/Human-wt/PRY/476/2000/G4P[8]	95,5
27	KJ559120-RVA/Human-wt/PRY/88/1998/G4P[8]	95,5
28	KJ626519-RVA/Human-wt/PRY/404/1999/G1P[8]	95,4
29	KJ559059-RVA/Human-wt/ARG/Res1730/1998/G4P[8]	95,4
30	KJ412877-RVA/Human-wt/PRY/1256A/2009/G3G4P[9]	95,4
31	KC193618-RVA/Human-wt/Bel/BE00061/2000/G1P[8]	95,3
32	KJ559132-RVA/Human-wt/ARG/Ush2037/1999/G4P[8]	95,3
33	KJ559199-RVA/Human-wt/PRY/358/1999/G4P[8]	95,3
34	KJ752411-RVA/Human-wt/ZAF/MRC-DPRU400/2003/G1P[8]	94,9
35	MG181431-RVA/Human-wt/MWI/MW2-1070/2004/G1P[8]	94,9
36	LC439274-RVA/Human-wt/GHA/M0094/2010/G9P[8]	94,8
37	KP752632-RVA/Human-wt/SEN/MRC-DPRU2051/2009/G9P[8]	94,8
38	JQ988894-RVA/Human-wt/Croatia/CR2006/2006/G8P[8]	94,6
39	JX195069-RVA/Human-wt/ITA/AV21/2010/G9P[8]	94,5
40	KC195764-RVA/Human-wt/AUS/CK00081/2007/G1P[8]	94,4
41	JX185764-RVA/Human-wt/ITA/ASTI23/2007/G9P[8]	94,4
42	KJ752853-RVA/Human-wt/ZWE/MRC-DPRU1850/2011/G4P[8]	94,3
43	HQ392454-RVA/Human-wt/BEL/BE00049/2009/G1P[8]	94,2
44	**RVA/Human-wt/RWA/UFS-NGS:MRC-DPRU8020/2015/G12P[8]**	**94,1**
45	**RVA/Human-wt/RWA/UFS-NGS:MRC-DPRU9995/2015/G12P[8]**	**94,1**
46	KU861385-RVA/Human-tc/USA/Wa/1974/G1P[8]	83,2
47	**RVA/Human-wt/RWA/UFS-NGS:MRC-DPRU6235/2014/G4P[4]**	**79,4**
48	HQ650120-RVA/Human-tc/USA/DS-1/1976/G2P[4]	70,1
49	JQ069421-RVA/Human-wt/CAN/RT125-07/2008/G2P[4]	69,6
50	KJ918866-RVA/Human-wt/HUN/ERN5635/2012/G2P[4]	69,6
51	LC469507-RVA/Human-wt/IDN/D13/2013/G3P[8]	69,2
52	KM660103-RVA/Human-wt/CMR/BA368/2010/G2P[4]	69,2
53	KC834696-RVA/Human-wt/AUS/V203/2009/G2P[4]	69,2
54	KC834697-RVA/Human-wt/AUS/336190/2004/G2P[4]	69,2
55	LC086731-RVA/Human-wt/THA/SKT-289/2013/G3P[8]	69,1
56	LC066656-RVA/Human-wt/THA/SKT-109/2013/G1P[8]	69,1
57	KU059772-RVA/Human-wt/AUS/D388/2013/G3P[8]	69,1
58	**RVA/Human-wt/RWA/UFS-NGS:MRC-DPRU6212/2014/G12P[8]**	**69,1**
59	KP007187-RVA/Human-wt/PHI/TGO12-012/2012/G1P[8]	69,0
60	LC066645-RVA/Human-wt/THA/PCB-180/2013/G1P[8]	69,0
61	LC086797-RVA/Human-wt/THA/SKT-138/2013/G2P[4]	68,9
62	LC469510-RVA/Human-wt/IDN/GRV60/2014/G1P[8]	68,9
63	LC086786-RVA/Human-wt/THA/NP-M51/2013/G2P[4]	68,8
64	LC173537-RVA/Human/JPN/SP025/2013/G1P[8]	68,8
65	AB792641-RVA/Human-tc/JPN/AU-1/1982/G3P[9]	41,1

	**RVA/Human-wt/RWA/UFS-NGS:MRC-DPRU8020/2015/G12P[8]**	
1	**RVA/Human-wt/RWA/UFS-NGS:MRC-DPRU9995/2015/G12P[8]**	**99,9**
2	KJ752853-RVA/Human-wt/ZWE/MRC-DPRU1850/2011/G4P[8]	99,2
3	JQ988894-RVA/Human-wt/Croatia/CR2006/2006/G8P[8]	99,1
4	KC195764-RVA/Human-wt/AUS/CK00081/2007/G1P[8]	98,9
5	JX185764-RVA/Human-wt/ITA/ASTI23/2007/G9P[8]	98,9
6	HQ392454-RVA/Human-wt/BEL/BE00049/2009/G1P[8]	98,5
7	KP752632-RVA/Human-wt/SEN/MRC-DPRU2051/2009/G9P[8]	98,3
8	LC439274-RVA/Human-wt/GHA/M0094/2010/G9P[8]	98,3
9	KJ559373-RVA/Human-wt/ARG/Mis864/1998/G4P[8]	98,3
10	KJ559070-RVA/Human-wt/ARG/Res1717/1998/G4P[8]	98,3
11	KJ559329-RVA/Human-wt/PRY/476/2000/G4P[8]	98,2
12	KJ559307-RVA/Human-wt/ARG/Tuc1662/1998/G4P[8]	98,2
13	KJ559120-RVA/Human-wt/PRY/88/1998/G4P[8]	98,2
14	KJ412877-RVA/Human-wt/PRY/1256A/2009/G3G4P[9]	98,2
15	JX195069-RVA/Human-wt/ITA/AV21/2010/G9P[8]	98,1
16	KJ626519-RVA/Human-wt/PRY/404/1999/G1P[8]	98,1
17	KJ559199-RVA/Human-wt/PRY/358/1999/G4P[8]	98,1
18	KC193618-RVA/Human-wt/Bel/BE00061/2000/G1P[8]	98,0
19	KJ559059-RVA/Human-wt/ARG/Res1730/1998/G4P[8]	98,0
20	KJ559132-RVA/Human-wt/ARG/Ush2037/1999/G4P[8]	98,0
21	KJ752411-RVA/Human-wt/ZAF/MRC-DPRU400/2003/G1P[8]	97,8
22	MG181431-RVA/Human-wt/MWI/MW2-1070/2004/G1P[8]	97,3
23	LC173562-RVA/Human/JPN/YM046/2013/G1P[8]	96,3
24	LC173555-RVA/Human/JPN/YM025/2013/G1P[8]	96,3
25	DQ146655-RVA/Human-wt/BGD/Dhaka25/2002/G12P[8]	96,2
26	DQ492675-RVA/Human-wt/BGD/Dhaka16/2003/G1P[8]	96,0
27	EF990709-RVA/Human-wt/BEL/B3458/2003/G9P[8]	95,8
28	KU048672-RVA/Human-wt/ITA/RG176/13/2013/G12P[8]	95,8
29	HQ657144-RVA/Human-wt/ZAF/3133WC/2009/G12P[4]	95,8
30	LC374165-RVA/Human-wt/NPL/09N3808/2009/G12P[6]	95,8
31	LC374187-RVA/Human-wt/NPL/10N4001/2010/G12P[6]	95,8
32	KU048673-RVA/Human-wt/ITA/RG177/13/2013/G12P[8]	95,7
33	LC368091-RVA/Human-wt/NPL/5N0014/2005/G12P[6]	95,7
34	MN067042-RVA/Human-wt/IND/CMC_00036/2010/G1P[8]	95,7
35	LC374198-RVA/Human-wt/NPL/10N4155/2010/G12P[6]	95,7
36	AB861962-RVA/Human-tc/KEN/KDH651/2010/G12P[8]	95,6
37	KU048678-RVA/Human-wt/ITA/PA525/14/2014/G12P[8]	95,5
38	KX655534-RVA/Cow-wt/UGA/BUW-14-A035/2014/G12P[8]	95,5
39	KM116052-RVA/Human-wt/USA/2013774166/2013/G12P[8]	95,5
40	MH171413-RVA/Human-wt/ESP/VI4816626/2012/G12P[8]	95,5
41	KU048659-RVA/Human-wt/ITA/PA93/12/2012/G12P[8]	95,5
42	KU048662-RVA/Human-wt/ITA/PA144/12/2012/G12P[8]	95,4
43	MH171402-RVA/Human-wt/ESP/SS257451/2012/G12P[8]	95,4
44	KU048677-RVA/Human-wt/ITA/PA417/14/2014/G12P[8]	95,4
45	**RVA/Human-wt/RWA/UFS-NGS:MRC-DPRU566/2013/G9P[4]**	**94,1**
46	KU861385-RVA/Human-tc/USA/Wa/1974/G1P[8]	82,1
47	**RVA/Human-wt/RWA/UFS-NGS:MRC-DPRU6235/2014/G4P[4]**	**79,1**
48	HQ650120-RVA/Human-tc/USA/DS-1/1976/G2P[4]	68,4
49	KM660103-RVA/Human-wt/CMR/BA368/2010/G2P[4]	65,6
50	**RVA/Human-wt/RWA/UFS-NGS:MRC-DPRU6212/2014/G12P[8]**	**65,6**
51	LC469507-RVA/Human-wt/IDN/D13/2013/G3P[8]	65,6
52	KC834696-RVA/Human-wt/AUS/V203/2009/G2P[4]	65,6
53	LC086731-RVA/Human-wt/THA/SKT-289/2013/G3P[8]	65,5
54	LC066656-RVA/Human-wt/THA/SKT-109/2013/G1P[8]	65,5
55	KU059772-RVA/Human-wt/AUS/D388/2013/G3P[8]	65,5
56	KP007187-RVA/Human-wt/PHI/TGO12-012/2012/G1P[8]	65,4
57	LC066645-RVA/Human-wt/THA/PCB-180/2013/G1P[8]	65,4
58	JQ069421-RVA/Human-wt/CAN/RT125-07/2008/G2P[4]	65,3
59	KJ918866-RVA/Human-wt/HUN/ERN5635/2012/G2P[4]	65,3
60	LC469510-RVA/Human-wt/IDN/GRV60/2014/G1P[8]	65,2
61	KC834697-RVA/Human-wt/AUS/336190/2004/G2P[4]	65,2
62	LC173537-RVA/Human/JPN/SP025/2013/G1P[8]	65,2
63	LC086797-RVA/Human-wt/THA/SKT-138/2013/G2P[4]	65,1
64	LC086786-RVA/Human-wt/THA/NP-M51/2013/G2P[4]	65,0
65	AB792641-RVA/Human-tc/JPN/AU-1/1982/G3P[9]	43,4

	**RVA/Human-wt/RWA/UFS-NGS:MRC-DPRU9995/2015/G12P[8]**	
1	**RVA/Human-wt/RWA/UFS-NGS:MRC-DPRU8020/2015/G12P[8]**	**99,9**
2	KJ752853-RVA/Human-wt/ZWE/MRC-DPRU1850/2011/G4P[8]	99,1
3	JQ988894-RVA/Human-wt/Croatia/CR2006/2006/G8P[8]	99,0
4	KC195764-RVA/Human-wt/AUS/CK00081/2007/G1P[8]	98,8
5	JX185764-RVA/Human-wt/ITA/ASTI23/2007/G9P[8]	98,8
6	HQ392454-RVA/Human-wt/BEL/BE00049/2009/G1P[8]	98,5
7	KP752632-RVA/Human-wt/SEN/MRC-DPRU2051/2009/G9P[8]	98,3
8	LC439274-RVA/Human-wt/GHA/M0094/2010/G9P[8]	98,3
9	KJ559373-RVA/Human-wt/ARG/Mis864/1998/G4P[8]	98,2
10	KJ559070-RVA/Human-wt/ARG/Res1717/1998/G4P[8]	98,2
11	KJ559329-RVA/Human-wt/PRY/476/2000/G4P[8]	98,1
12	KJ559307-RVA/Human-wt/ARG/Tuc1662/1998/G4P[8]	98,1
13	KJ559120-RVA/Human-wt/PRY/88/1998/G4P[8]	98,1
14	KJ412877-RVA/Human-wt/PRY/1256A/2009/G3G4P[9]	98,1
15	JX195069-RVA/Human-wt/ITA/AV21/2010/G9P[8]	98,0
16	KJ626519-RVA/Human-wt/PRY/404/1999/G1P[8]	98,0
17	KJ559199-RVA/Human-wt/PRY/358/1999/G4P[8]	98,0
18	KC193618-RVA/Human-wt/Bel/BE00061/2000/G1P[8]	98,0
19	KJ559059-RVA/Human-wt/ARG/Res1730/1998/G4P[8]	98,0
20	KJ559132-RVA/Human-wt/ARG/Ush2037/1999/G4P[8]	98,0
21	KJ752411-RVA/Human-wt/ZAF/MRC-DPRU400/2003/G1P[8]	97,8
22	MG181431-RVA/Human-wt/MWI/MW2-1070/2004/G1P[8]	97,3
23	LC173562-RVA/Human/JPN/YM046/2013/G1P[8]	96,2
24	LC173555-RVA/Human/JPN/YM025/2013/G1P[8]	96,2
25	DQ146655-RVA/Human-wt/BGD/Dhaka25/2002/G12P[8]	96,1
26	DQ492675-RVA/Human-wt/BGD/Dhaka16/2003/G1P[8]	96,0
27	EF990709-RVA/Human-wt/BEL/B3458/2003/G9P[8]	95,7
28	KU048672-RVA/Human-wt/ITA/RG176/13/2013/G12P[8]	95,7
29	HQ657144-RVA/Human-wt/ZAF/3133WC/2009/G12P[4]	95,7
30	LC374165-RVA/Human-wt/NPL/09N3808/2009/G12P[6]	95,7
31	LC374187-RVA/Human-wt/NPL/10N4001/2010/G12P[6]	95,7
32	KU048673-RVA/Human-wt/ITA/RG177/13/2013/G12P[8]	95,7
33	LC368091-RVA/Human-wt/NPL/5N0014/2005/G12P[6]	95,7
34	MN067042-RVA/Human-wt/IND/CMC_00036/2010/G1P[8]	95,6
35	LC374198-RVA/Human-wt/NPL/10N4155/2010/G12P[6]	95,6
36	AB861962-RVA/Human-tc/KEN/KDH651/2010/G12P[8]	95,5
37	KU048678-RVA/Human-wt/ITA/PA525/14/2014/G12P[8]	95,4
38	KX655534-RVA/Cow-wt/UGA/BUW-14-A035/2014/G12P[8]	95,4
39	KM116052-RVA/Human-wt/USA/2013774166/2013/G12P[8]	95,4
40	MH171413-RVA/Human-wt/ESP/VI4816626/2012/G12P[8]	95,4
41	KU048659-RVA/Human-wt/ITA/PA93/12/2012/G12P[8]	95,4
42	KU048662-RVA/Human-wt/ITA/PA144/12/2012/G12P[8]	95,3
43	MH171402-RVA/Human-wt/ESP/SS257451/2012/G12P[8]	95,3
44	KU048677-RVA/Human-wt/ITA/PA417/14/2014/G12P[8]	95,3
45	**RVA/Human-wt/RWA/UFS-NGS:MRC-DPRU566/2013/G9P[4]**	**94,1**
46	KU861385-RVA/Human-tc/USA/Wa/1974/G1P[8]	82,0
47	**RVA/Human-wt/RWA/UFS-NGS:MRC-DPRU6235/2014/G4P[4]**	**79,1**
48	HQ650120-RVA/Human-tc/USA/DS-1/1976/G2P[4]	68,3
49	KM660103-RVA/Human-wt/CMR/BA368/2010/G2P[4]	65,6
50	**RVA/Human-wt/RWA/UFS-NGS:MRC-DPRU6212/2014/G12P[8]**	**65,6**
51	LC469507-RVA/Human-wt/IDN/D13/2013/G3P[8]	65,5
52	KC834696-RVA/Human-wt/AUS/V203/2009/G2P[4]	65,5
53	LC086731-RVA/Human-wt/THA/SKT-289/2013/G3P[8]	65,5
54	LC066656-RVA/Human-wt/THA/SKT-109/2013/G1P[8]	65,5
55	KU059772-RVA/Human-wt/AUS/D388/2013/G3P[8]	65,5
56	KP007187-RVA/Human-wt/PHI/TGO12-012/2012/G1P[8]	65,4
57	LC066645-RVA/Human-wt/THA/PCB-180/2013/G1P[8]	65,4
58	JQ069421-RVA/Human-wt/CAN/RT125-07/2008/G2P[4]	65,3
59	KJ918866-RVA/Human-wt/HUN/ERN5635/2012/G2P[4]	65,3
60	LC469510-RVA/Human-wt/IDN/GRV60/2014/G1P[8]	65,2
61	KC834697-RVA/Human-wt/AUS/336190/2004/G2P[4]	65,2
62	LC173537-RVA/Human/JPN/SP025/2013/G1P[8]	65,1
63	LC086797-RVA/Human-wt/THA/SKT-138/2013/G2P[4]	65,1
64	LC086786-RVA/Human-wt/THA/NP-M51/2013/G2P[4]	64,9
65	AB792641-RVA/Human-tc/JPN/AU-1/1982/G3P[9]	43,3

	**RVA/Human-wt/RWA/UFS-NGS:MRC-DPRU6212/2014/G12P[8]**	
1	KU059772-RVA/Human-wt/AUS/D388/2013/G3P[8]	99,2
2	LC086786-RVA/Human-wt/THA/NP-M51/2013/G2P[4]	99,2
3	LC066656-RVA/Human-wt/THA/SKT-109/2013/G1P[8]	99,2
4	LC086731-RVA/Human-wt/THA/SKT-289/2013/G3P[8]	99,2
5	LC086797-RVA/Human-wt/THA/SKT-138/2013/G2P[4]	99,2
6	LC066645-RVA/Human-wt/THA/PCB-180/2013/G1P[8]	99,1
7	KP007187-RVA/Human-wt/PHI/TGO12-012/2012/G1P[8]	99,1
8	LC173537-RVA/Human/JPN/SP025/2013/G1P[8]	99,0
9	LC469507-RVA/Human-wt/IDN/D13/2013/G3P[8]	99,0
10	LC469510-RVA/Human-wt/IDN/GRV60/2014/G1P[8]	99,0
11	KC834697-RVA/Human-wt/AUS/336190/2004/G2P[4]	97,5
12	KC834696-RVA/Human-wt/AUS/V203/2009/G2P[4]	97,1
13	JQ069421-RVA/Human-wt/CAN/RT125-07/2008/G2P[4]	97,1
14	KJ918866-RVA/Human-wt/HUN/ERN5635/2012/G2P[4]	97,0
15	KM660103-RVA/Human-wt/CMR/BA368/2010/G2P[4]	96,5
16	HQ650120-RVA/Human-tc/USA/DS-1/1976/G2P[4]	92,6
17	**RVA/Human-wt/RWA/UFS-NGS:MRC-DPRU6235/2014/G4P[4]**	**90,5**
18	**RVA/Human-wt/RWA/UFS-NGS:MRC-DPRU566/2013/G9P[4]**	**69,1**
19	KU861385-RVA/Human-tc/USA/Wa/1974/G1P[8]	67,4
20	HQ657144-RVA/Human-wt/ZAF/3133WC/2009/G12P[4]	67,2
21	KU048677-RVA/Human-wt/ITA/PA417/14/2014/G12P[8]	67,1
22	KM116052-RVA/Human-wt/USA/2013774166/2013/G12P[8]	67,1
23	KU048678-RVA/Human-wt/ITA/PA525/14/2014/G12P[8]	67,0
24	KX655534-RVA/Cow-wt/UGA/BUW-14-A035/2014/G12P[8]	66,9
25	MN067042-RVA/Human-wt/IND/CMC_00036/2010/G1P[8]	66,9
26	KU048672-RVA/Human-wt/ITA/RG176/13/2013/G12P[8]	66,9
27	EF990709-RVA/Human-wt/BEL/B3458/2003/G9P[8]	66,9
28	MH171413-RVA/Human-wt/ESP/VI4816626/2012/G12P[8]	66,8
29	KU048659-RVA/Human-wt/ITA/PA93/12/2012/G12P[8]	66,8
30	KU048673-RVA/Human-wt/ITA/RG177/13/2013/G12P[8]	66,8
31	AB861962-RVA/Human-tc/KEN/KDH651/2010/G12P[8]	66,8
32	LC368091-RVA/Human-wt/NPL/5N0014/2005/G12P[6]	66,8
33	KU048662-RVA/Human-wt/ITA/PA144/12/2012/G12P[8]	66,7
34	LC374187-RVA/Human-wt/NPL/10N4001/2010/G12P[6]	66,7
35	MH171402-RVA/Human-wt/ESP/SS257451/2012/G12P[8]	66,7
36	DQ146655-RVA/Human-wt/BGD/Dhaka25/2002/G12P[8]	66,6
37	DQ492675-RVA/Human-wt/BGD/Dhaka16/2003/G1P[8]	66,6
38	LC173562-RVA/Human/JPN/YM046/2013/G1P[8]	66,6
39	LC173555-RVA/Human/JPN/YM025/2013/G1P[8]	66,6
40	LC374198-RVA/Human-wt/NPL/10N4155/2010/G12P[6]	66,5
41	LC374165-RVA/Human-wt/NPL/09N3808/2009/G12P[6]	66,4
42	KJ559373-RVA/Human-wt/ARG/Mis864/1998/G4P[8]	66,0
43	KJ559329-RVA/Human-wt/PRY/476/2000/G4P[8]	66,0
44	KJ559307-RVA/Human-wt/ARG/Tuc1662/1998/G4P[8]	66,0
45	KJ559070-RVA/Human-wt/ARG/Res1717/1998/G4P[8]	66,0
46	KJ626519-RVA/Human-wt/PRY/404/1999/G1P[8]	66,0
47	KJ559120-RVA/Human-wt/PRY/88/1998/G4P[8]	65,9
48	KJ559199-RVA/Human-wt/PRY/358/1999/G4P[8]	65,9
49	KJ559059-RVA/Human-wt/ARG/Res1730/1998/G4P[8]	65,8
50	KJ412877-RVA/Human-wt/PRY/1256A/2009/G3G4P[9]	65,8
51	KC193618-RVA/Human-wt/Bel/BE00061/2000/G1P[8]	65,8
52	KJ559132-RVA/Human-wt/ARG/Ush2037/1999/G4P[8]	65,7
53	JX185764-RVA/Human-wt/ITA/ASTI23/2007/G9P[8]	65,6
54	**RVA/Human-wt/RWA/UFS-NGS:MRC-DPRU8020/2015/G12P[8]**	**65,6**
55	**RVA/Human-wt/RWA/UFS-NGS:MRC-DPRU9995/2015/G12P[8]**	**65,6**
56	LC439274-RVA/Human-wt/GHA/M0094/2010/G9P[8]	65,6
57	KJ752411-RVA/Human-wt/ZAF/MRC-DPRU400/2003/G1P[8]	65,5
58	KC195764-RVA/Human-wt/AUS/CK00081/2007/G1P[8]	65,5
59	KP752632-RVA/Human-wt/SEN/MRC-DPRU2051/2009/G9P[8]	65,5
60	JQ988894-RVA/Human-wt/Croatia/CR2006/2006/G8P[8]	65,4
61	HQ392454-RVA/Human-wt/BEL/BE00049/2009/G1P[8]	65,3
62	MG181431-RVA/Human-wt/MWI/MW2-1070/2004/G1P[8]	65,3
63	KJ752853-RVA/Human-wt/ZWE/MRC-DPRU1850/2011/G4P[8]	65,3
64	JX195069-RVA/Human-wt/ITA/AV21/2010/G9P[8]	65,2
65	AB792641-RVA/Human-tc/JPN/AU-1/1982/G3P[9]	40,8

G. NSP3

	**RVA/Human-wt/RWA/UFS-NGS:MRC-DPRU6235/2014/G4P[4]**	
1	**RVA/Human-wt/RWA/UFS-NGS:MRC-DPRU566/2013/G9P[4]**	**96,8**
2	**RVA/Human-wt/RWA/UFS-NGS:MRC-DPRU8020/2015/G12P[8]**	**99,0**
3	**RVA/Human-wt/RWA/UFS-NGS:MRC-DPRU9995/2015/G12P[8]**	**99,0**
4	**RVA/Human-wt/RWA/UFS-NGS:MRC-DPRU6212/2014/G12P[8]**	**89,8**
5	KM230944-RVA/Human-xx/ITA/RC37/2009/G10P[8]	98,9
6	KJ752855-RVA/Human-wt/ZWE/MRC-DPRU1850/2011/G4P[8]	98,9
7	JN258918-RVA/Human-wt/Bel/BE00097/2009/G1P[8]	98,8
8	KJ559386-RVA/Human-wt/PRY/3/1998/G4P[8]	98,7
9	JN258334-RVA/Human-wt/USA/2007719720/2007/G1P[8]	98,6
10	HM773772-RVA/Human-wt/USA2007719698/2007/G1P[8]	98,4
11	JF490870-RVA/Human/USA/VU06-07-31/2006/G1P[8]	98,4
12	JN258381-RVA/Human-wt/USA/2008747288/2008/G1P[8]	98,4
13	JX185766-RVA/Human-wt/ITA/ASTI23/2007/G9P[8]	98,3
14	JX416224-RVA/Human-tc/AUS/McN13/1980/G3P2A[6]	98,2
15	JX943606-RVA/Human-TC/USA/Rotarix/2009/G1P[8]	98,2
16	KT695112-RVA/Human-wt/USA/DC5685/1991/G1P[8]	98,2
17	JX406753-RVA/Human-tc/USA/Wa/1974/G1P[8]	97,9
18	JN013980-RVA/Human-wt/ZAF/2371WC/2008/G9P[8]	97,9
19	MH171443-RVA/Human-wt/ESP/SS454877/2011/G1P[8]	98,0
20	KU048716-RVA/Human-wt/ITA/PA414/12/2012/G12P[8]	97,8
21	LC439278-RVA/Human-wt/GHA/M0094/2010/G9P[8]	97,8
22	LC439277-RVA/Human-wt/GHA/DC949/2010/G9P[8]	97,8
23	JX195082-RVA/Human-wt/ITA/AV28/2010/G9P[8]	97,9
24	JQ988896-RVA/Human-wt/HRV/CR2006/2006/G8P[8]	97,7
25	LC173793-RVA/Human-xx/JPN/NT064/2013/G1P[8]	98,0
26	JX629048-RVA/Human-tc/JPN/YO/1977/G3P1A[8]	97,8
27	KJ870919-RVA/Human-wt/COD/KisB521/2008/G12P[6]	97,9
28	KX632317-RVA/Human-wt/UGA/MUL-12-093/2012/G9P[8]	97,9
29	KU048703-RVA/Human-wt/ITA/PA93/12/2012/G12P[8]	96,9
30	KX655536-RVA/Cow-wt/UGA/BUW-14-A035/2014/G12P[8]	96,8
31	KM116054-RVA/Human-wt/USA/2013774166/2013/G12P[8]	96,9
32	KU048707-RVA/Human-wt/ITA/PA417/14/2014/G12P[8]	97,0
33	EF990711-RVA/Human-wt/BEL/B3458/2003/G9P[8]	97,0
34	LC374134-RVA/Human-wt/NPL/09N3140/2009/G12P[6]	97,1
35	MG181532-RVA/Human-wt/MWI/BID14A/2012/G1P[8]	97,0
36	LC019060-RVA/Human-tc/MMR/A23/2011/G12P[6]	97,0
37	AB861964-RVA/Human-tc/KEN/KDH651/2010/G12P[8]	96,9
38	MG926711-RVA/Human-wt/MOZ/0289/2012/G12P[6]	96,8
39	MH171437-RVA/Human-wt/ESP/SS65130987/2015/G12P[8]	96,8
40	KU048715-RVA/Human-wt/ITA/ME650/14/2014/G12P[8]	96,9
41	KU048714-RVA/Human-wt/ITA/PA525/14/2014/G12P[8]	96,9
42	MH171449-RVA/Human-wt/ESP/SS64751571/2016/G12P[8]	96,7
43	KU048708-RVA/Human-wt/ITA/ME659/14/2014/G12P[8]	96,8
44	JX195093-RVA/Human-wt/ITA/JES11/2010/G9P[8]	97,0
45	KX363069-RVA/Human-wt/VNM/12053_55/2012/GXP[X]	96,8
46	KF371989-RVA/Human-wt/CHN/Y106/2004/G3P[8]	97,6
47	LC173794-RVA/Human-xx/JPN/KN067/2013/G1P[8]	97,7
48	GU565049-RVA/Vaccine/USA/RotaTeq-WI79-4/1992/G6P1A[8]	91,0
49	GU565060-RVA/Vaccine/USA/RotaTeq-WI79-9/1992/G1P7[5]	91,0
50	GU565071-RVA/Vaccine/USA/RotaTeq-SC2-9/1992/G2P7[5]	91,0
51	GU565093-RVA/Vaccine/USA/RotaTeq-BrB-9/1996/G4P7[5]	91,0
52	GU565082-RVA/Vaccine/USA/RotaTeq-WI78-8/1992/G3P7[5]	91,0
53	KX212881-RVA/Cow-wt/TUR/Amasya-1/2015/G8P[5]	90,6
54	KU128900-RVA/Human-wt/Bel/BEF06018/2014/G29P[41]	90,5
55	KU317463-RVA/Human-tc/EGY/AS970/2012/G8P[14]	91,7
56	KJ751919-RVA/Cow-wt/ZAF/MRC-DPRU3005/2009/G6P[11]	91,3
57	KX655470-RVA/Human-wt/UGA/MUL-13-496/2013/G8P[4]	89,9
58	LC406827-RVA/Human-wt/KEN/KDH1255/2012/G8P[4]	89,8
59	KP752568-RVA/Human-wt/TZA/MRC-DPRU4570/2011/G8P[4]	90,0
60	KF636242-RVA/Human-wt/KEN/MRC-DPRU1606/2009/G8P[4]	89,8
61	KC834692-RVA/Human-wt/AUS/V233/1999/G2P[4]	89,8
62	KX655459-RVA/Human-wt/UGA/MUL-13-204/2013/G8P[6]	89,8
63	HQ657135-RVA/Human-wt/MWI/1473/2001/G8P[4]	89,8
64	DQ005117-RVA/Human-wt/COD/DRC86/2003/G8P[6]	90,0
65	DQ005106-RVA/Human-wt/COD/DRC88/2003/G8P[8]	90,0
66	DQ490542-RVA/Human-wt/BGD/RV161/2000/G12P[6]	89,7
67	DQ146679-RVA/Human-xx/BGD/Matlab13/2003/G12P[6]	89,7
68	KC571499-RVA/Human-wt/AUS/SA066/2010/G2P[4]	89,7
69	KU248380-RVA/Human-wt/BGN/J303/2010/G2P[4]	89,7
70	MG181928-RVA/Human-wt/MWI/BID1CT/2012/G2P[4]	89,8
71	JQ069253-RVA/Human-wt/CAN/RT125-07/2008/G2P[4]	90,1
72	KX362752-RVA/Human-wt/VNM/16020_81/2013/GXP[X]	90,0
73	JF460842-RVA/Human/BEL/F01498/2009/G3P[6]	89,9
74	JN013979-RVA/Human-wt/ZAF/2371WC/2008/G9P[8]	89,9
75	MG181829-RVA/Human-wt/MWI/BID11E/2012/G2P[4]	89,9
76	KC834691-RVA/Human-wt/AUS/336190/2004/G2P[4]	89,5
77	KX655514-RVA/Human-wt/UGA/MSK-13-048/2013/G9P[8]	90,0
78	MG181422-RVA/Human-wt/MWI/MW2-1057/2004/G1P[8]	90,1
79	EF554090-RVA/Human-wt/BEL/B1711/2002/G6P[6]	90,0
80	HQ650122-RVA/Human-tc/USA/DS-1/1976/G2P[4]	89,5
81	DQ490535-RVA/Human-tc/JPN/AU-1/1982/G3P3[9]	88,1

	**RVA/Human-wt/RWA/UFS-NGS:MRC-DPRU566/2013/G9P[4]**	
1	KU048703-RVA/Human-wt/ITA/PA93/12/2012/G12P[8]	98,0
2	KM116054-RVA/Human-wt/USA/2013774166/2013/G12P[8]	97,9
3	KX655536-RVA/Cow-wt/UGA/BUW-14-A035/2014/G12P[8]	97,9
4	KU048715-RVA/Human-wt/ITA/ME650/14/2014/G12P[8]	97,6
5	LC374134-RVA/Human-wt/NPL/09N3140/2009/G12P[6]	97,6
6	KU048707-RVA/Human-wt/ITA/PA417/14/2014/G12P[8]	97,6
7	EF990711-RVA/Human-wt/BEL/B3458/2003/G9P[8]	97,6
8	KU048714-RVA/Human-wt/ITA/PA525/14/2014/G12P[8]	97,5
9	AB861964-RVA/Human-tc/KEN/KDH651/2010/G12P[8]	97,5
10	LC019060-RVA/Human-tc/MMR/A23/2011/G12P[6]	97,5
11	MG181532-RVA/Human-wt/MWI/BID14A/2012/G1P[8]	97,5
12	MG926711-RVA/Human-wt/MOZ/0289/2012/G12P[6]	97,5
13	MH171449-RVA/Human-wt/ESP/SS64751571/2016/G12P[8]	97,5
14	KX363069-RVA/Human-wt/VNM/12053_55/2012/GXP[X]	97,5
15	KF371989-RVA/Human-wt/CHN/Y106/2004/G3P[8]	97,5
16	JX195093-RVA/Human-wt/ITA/JES11/2010/G9P[8]	97,4
17	KU048708-RVA/Human-wt/ITA/ME659/14/2014/G12P[8]	97,4
18	MH171437-RVA/Human-wt/ESP/SS65130987/2015/G12P[8]	97,4
19	LC173794-RVA/Human-xx/JPN/KN067/2013/G1P[8]	97,4
20	**RVA/Human-wt/RWA/UFS-NGS:MRC-DPRU6235/2014/G4P[4]**	**96,8**
21	JX416224-RVA/Human-tc/AUS/McN13/1980/G3P2A[6]	96,4
22	HM773772-RVA/Human-wt/USA2007719698/2007/G1P[8]	96,3
23	JF490870-RVA/Human/USA/VU06-07-31/2006/G1P[8]	96,3
24	JN258381-RVA/Human-wt/USA/2008747288/2008/G1P[8]	96,3
25	KT695112-RVA/Human-wt/USA/DC5685/1991/G1P[8]	96,3
26	JX629048-RVA/Human-tc/JPN/YO/1977/G3P1A[8]	96,3
27	JX943606-RVA/Human-TC/USA/Rotarix/2009/G1P[8]	96,3
28	MH171443-RVA/Human-wt/ESP/SS454877/2011/G1P[8]	96,3
29	LC439278-RVA/Human-wt/GHA/M0094/2010/G9P[8]	96,2
30	LC439277-RVA/Human-wt/GHA/DC949/2010/G9P[8]	96,2
31	JN013980-RVA/Human-wt/ZAF/2371WC/2008/G9P[8]	96,1
32	JX195082-RVA/Human-wt/ITA/AV28/2010/G9P[8]	96,1
33	KU048716-RVA/Human-wt/ITA/PA414/12/2012/G12P[8]	96,1
34	KJ559386-RVA/Human-wt/PRY/3/1998/G4P[8]	96,1
35	JN258334-RVA/Human-wt/USA/2007719720/2007/G1P[8]	96,1
36	**RVA/Human-wt/RWA/UFS-NGS:MRC-DPRU8020/2015/G12P[8]**	**96,0**
37	**RVA/Human-wt/RWA/UFS-NGS:MRC-DPRU9995/2015/G12P[8]**	**96,0**
38	JQ988896-RVA/Human-wt/HRV/CR2006/2006/G8P[8]	96,0
39	JX406753-RVA/Human-tc/USA/Wa/1974/G1P[8]	96,0
40	JN258918-RVA/Human-wt/Bel/BE00097/2009/G1P[8]	96,0
41	KM230944-RVA/Human-xx/ITA/RC37/2009/G10P[8]	95,9
42	KJ752855-RVA/Human-wt/ZWE/MRC-DPRU1850/2011/G4P[8]	95,9
43	LC173793-RVA/Human-xx/JPN/NT064/2013/G1P[8]	95,9
44	JX185766-RVA/Human-wt/ITA/ASTI23/2007/G9P[8]	95,8
45	KX632317-RVA/Human-wt/UGA/MUL-12-093/2012/G9P[8]	95,8
46	KJ870919-RVA/Human-wt/COD/KisB521/2008/G12P[6]	95,7
47	KX655470-RVA/Human-wt/UGA/MUL-13-496/2013/G8P[4]	91,2
48	EF554090-RVA/Human-wt/BEL/B1711/2002/G6P[6]	91,2
49	DQ005117-RVA/Human-wt/COD/DRC86/2003/G8P[6]	91,1
50	MG181422-RVA/Human-wt/MWI/MW2-1057/2004/G1P[8]	91,1
51	KX655514-RVA/Human-wt/UGA/MSK-13-048/2013/G9P[8]	91,0
52	**RVA/Human-wt/RWA/UFS-NGS:MRC-DPRU6212/2014/G12P[8]**	**91,0**
53	KX362752-RVA/Human-wt/VNM/16020_81/2013/GXP[X]	91,0
54	HQ657135-RVA/Human-wt/MWI/1473/2001/G8P[4]	91,0
55	DQ490542-RVA/Human-wt/BGD/RV161/2000/G12P[6]	91,0
56	DQ005106-RVA/Human-wt/COD/DRC88/2003/G8P[8]	91,0
57	KF636242-RVA/Human-wt/KEN/MRC-DPRU1606/2009/G8P[4]	90,9
58	KP752568-RVA/Human-wt/TZA/MRC-DPRU4570/2011/G8P[4]	90,9
59	MG181928-RVA/Human-wt/MWI/BID1CT/2012/G2P[4]	90,9
60	MG181829-RVA/Human-wt/MWI/BID11E/2012/G2P[4]	90,9
61	KC834692-RVA/Human-wt/AUS/V233/1999/G2P[4]	90,8
62	KX655459-RVA/Human-wt/UGA/MUL-13-204/2013/G8P[6]	90,8
63	KC571499-RVA/Human-wt/AUS/SA066/2010/G2P[4]	90,8
64	DQ146679-RVA/Human-xx/BGD/Matlab13/2003/G12P[6]	90,8
65	LC406827-RVA/Human-wt/KEN/KDH1255/2012/G8P[4]	90,8
66	KC834691-RVA/Human-wt/AUS/336190/2004/G2P[4]	90,7
67	KU248380-RVA/Human-wt/BGN/J303/2010/G2P[4]	90,7
68	JQ069253-RVA/Human-wt/CAN/RT125-07/2008/G2P[4]	90,7
69	KU317463-RVA/Human-tc/EGY/AS970/2012/G8P[14]	90,6
70	JF460842-RVA/Human/BEL/F01498/2009/G3P[6]	90,5
71	JN013979-RVA/Human-wt/ZAF/2371WC/2008/G9P[8]	90,5
72	KJ751919-RVA/Cow-wt/ZAF/MRC-DPRU3005/2009/G6P[11]	90,5
73	HQ650122-RVA/Human-tc/USA/DS-1/1976/G2P[4]	90,2
74	KU128900-RVA/Human-wt/Bel/BEF06018/2014/G29[P41]	90,1
75	GU565049-RVA/Vaccine/USA/RotaTeq-WI79-4/1992/G6P1A[8]	90,0
76	GU565060-RVA/Vaccine/USA/RotaTeq-WI79-9/1992/G1P7[5]	90,0
77	GU565071-RVA/Vaccine/USA/RotaTeq-SC2-9/1992/G2P7[5]	90,0
78	GU565093-RVA/Vaccine/USA/RotaTeq-BrB-9/1996/G4P7[]5	90,0
79	GU565082-RVA/Vaccine/USA/RotaTeq-WI78-8/1992/G3P7[5]	90,0
80	KX212881-RVA/Cow-wt/TUR/Amasya-1/2015/G8P[5]	89,5
81	DQ490535-RVA/Human-tc/JPN/AU-1/1982/G3P3[9]	87,3

	**RVA/Human-wt/RWA/UFS-NGS:MRC-DPRU8020/2015/G12P[8]**	
1	**RVA/Human-wt/RWA/UFS-NGS:MRC-DPRU9995/2015/G12P[8]**	**100,0**
2	KM230944-RVA/Human-xx/ITA/RC37/2009/G10P[8]	99,7
3	KJ752855-RVA/Human-wt/ZWE/MRC-DPRU1850/2011/G4P[8]	99,7
4	JN258918-RVA/Human-wt/Bel/BE00097/2009/G1P[8]	99,6
5	KJ559386-RVA/Human-wt/PRY/3/1998/G4P[8]	99,6
6	JN258334-RVA/Human-wt/USA/2007719720/2007/G1P[8]	99,5
7	HM773772-RVA/Human-wt/USA2007719698/2007/G1P[8]	99,2
8	JF490870-RVA/Human/USA/VU06-07-31/2006/G1P[8]	99,2
9	JN258381-RVA/Human-wt/USA/2008747288/2008/G1P[8]	99,2
10	JX185766-RVA/Human-wt/ITA/ASTI23/2007/G9P[8]	99,0
11	**RVA/Human-wt/RWA/UFS-NGS:MRC-DPRU6235/2014/G4P[4]**	**99,0**
12	JX943606-RVA/Human-TC/USA/Rotarix/2009/G1P[8]	98,9
13	LC173793-RVA/Human-xx/JPN/NT064/2013/G1P[8]	98,9
14	JX416224-RVA/Human-tc/AUS/McN13/1980/G3P2A[6]	98,8
15	KT695112-RVA/Human-wt/USA/DC5685/1991/G1P[8]	98,6
16	JX629048-RVA/Human-tc/JPN/YO/1977/G3P1A[8]	98,6
17	KX632317-RVA/Human-wt/UGA/MUL-12-093/2012/G9P[8]	98,5
18	JN013980-RVA/Human-wt/ZAF/2371WC/2008/G9P[8]	98,5
19	KJ870919-RVA/Human-wt/COD/KisB521/2008/G12P[6]	98,5
20	MH171443-RVA/Human-wt/ESP/SS454877/2011/G1P[8]	98,5
21	JX195082-RVA/Human-wt/ITA/AV28/2010/G9P[8]	98,5
22	JX406753-RVA/Human-tc/USA/Wa/1974/G1P[8]	98,5
23	LC439278-RVA/Human-wt/GHA/M0094/2010/G9P[8]	98,5
24	LC439277-RVA/Human-wt/GHA/DC949/2010/G9P[8]	98,5
25	KU048716-RVA/Human-wt/ITA/PA414/12/2012/G12P[8]	98,5
26	LC173794-RVA/Human-xx/JPN/KN067/2013/G1P[8]	98,4
27	KF371989-RVA/Human-wt/CHN/Y106/2004/G3P[8]	98,3
28	JQ988896-RVA/Human-wt/HRV/CR2006/2006/G8P[8]	98,3
29	LC374134-RVA/Human-wt/NPL/09N3140/2009/G12P[6]	98,0
30	KU048707-RVA/Human-wt/ITA/PA417/14/2014/G12P[8]	97,9
31	EF990711-RVA/Human-wt/BEL/B3458/2003/G9P[8]	97,9
32	JX195093-RVA/Human-wt/ITA/JES11/2010/G9P[8]	97,9
33	MG181532-RVA/Human-wt/MWI/BID14A/2012/G1P[8]	97,9
34	AB861964-RVA/Human-tc/KEN/KDH651/2010/G12P[8]	97,8
35	LC019060-RVA/Human-tc/MMR/A23/2011/G12P[6]	97,8
36	MG926711-RVA/Human-wt/MOZ/0289/2012/G12P[6]	97,7
37	KM116054-RVA/Human-wt/USA/2013774166/2013/G12P[8]	97,7
38	KU048715-RVA/Human-wt/ITA/ME650/14/2014/G12P[8]	97,7
39	KU048714-RVA/Human-wt/ITA/PA525/14/2014/G12P[8]	97,7
40	KU048703-RVA/Human-wt/ITA/PA93/12/2012/G12P[8]	97,7
41	KU048708-RVA/Human-wt/ITA/ME659/14/2014/G12P[8]	97,7
42	KX363069-RVA/Human-wt/VNM/12053_55/2012/GXP[X]	97,7
43	KX655536-RVA/Cow-wt/UGA/BUW-14-A035/2014/G12P[8]	97,6
44	MH171437-RVA/Human-wt/ESP/SS65130987/2015/G12P[8]	97,6
45	MH171449-RVA/Human-wt/ESP/SS64751571/2016/G12P[8]	97,6
46	**RVA/Human-wt/RWA/UFS-NGS:MRC-DPRU566/2013/G9P[4]**	**96,0**
47	KU317463-RVA/Human-tc/EGY/AS970/2012/G8P[14]	91,7
48	KJ751919-RVA/Cow-wt/ZAF/MRC-DPRU3005/2009/G6P[11]	91,4
49	GU565049-RVA/Vaccine/USA/RotaTeq-WI79-4/1992/G6P1A[8]	91,1
50	GU565060-RVA/Vaccine/USA/RotaTeq-WI79-9/1992/G1P7[5]	91,1
51	GU565071-RVA/Vaccine/USA/RotaTeq-SC2-9/1992/G2P7[5]	91,1
52	GU565093-RVA/Vaccine/USA/RotaTeq-BrB-9/1996/G4P7[5]	91,1
53	GU565082-RVA/Vaccine/USA/RotaTeq-WI78-8/1992/G3P7[5]	91,1
54	KX212881-RVA/Cow-wt/TUR/Amasya-1/2015/G8P[5]	90,7
55	KU128900-RVA/Human-wt/Bel/BEF06018/2014/G29P[41]	90,6
56	KX362752-RVA/Human-wt/VNM/16020_81/2013/GXP[X]	89,2
57	JQ069253-RVA/Human-wt/CAN/RT125-07/2008/G2P[4]	89,1
58	DQ005117-RVA/Human-wt/COD/DRC86/2003/G8P[6]	89,0
59	KP752568-RVA/Human-wt/TZA/MRC-DPRU4570/2011/G8P[4]	89,0
60	MG181422-RVA/Human-wt/MWI/MW2-1057/2004/G1P[8]	89,0
61	EF554090-RVA/Human-wt/BEL/B1711/2002/G6P[6]	89,0
62	DQ005106-RVA/Human-wt/COD/DRC88/2003/G8P[8]	89,0
63	KX655514-RVA/Human-wt/UGA/MSK-13-048/2013/G9P[8]	88,9
64	MG181829-RVA/Human-wt/MWI/BID11E/2012/G2P[4]	88,9
65	KX655459-RVA/Human-wt/UGA/MUL-13-204/2013/G8P[6]	88,9
66	JF460842-RVA/Human/BEL/F01498/2009/G3P[6]	88,9
67	HQ657135-RVA/Human-wt/MWI/1473/2001/G8P[4]	88,8
68	KC834692-RVA/Human-wt/AUS/V233/1999/G2P[]4	88,8
69	LC406827-RVA/Human-wt/KEN/KDH1255/2012/G8P[4]	88,8
70	JN013979-RVA/Human-wt/ZAF/2371WC/2008/G9P[8]	88,8
71	KF636242-RVA/Human-wt/KEN/MRC-DPRU1606/2009/G8P[4]	88,8
72	DQ490542-RVA/Human-wt/BGD/RV161/2000/G12P[6]	88,7
73	KU248380-RVA/Human-wt/BGN/J303/2010/G2P[4]	88,7
74	DQ146679-RVA/Human-xx/BGD/Matlab13/2003/G12P[6]	88,7
75	KC571499-RVA/Human-wt/AUS/SA066/2010/G2P[4]	88,7
76	MG181928-RVA/Human-wt/MWI/BID1CT/2012/G2P[4]	88,7
77	KX655470-RVA/Human-wt/UGA/MUL-13-496/2013/G8P[4]	88,5
78	KC834691-RVA/Human-wt/AUS/336190/2004/G2P[4]	88,5
79	HQ650122-RVA/Human-tc/USA/DS-1/1976/G2P[4]	88,5
80	**RVA/Human-wt/RWA/UFS-NGS:MRC-DPRU6212/2014/G12P[8]**	**88,5**
81	DQ490535-RVA/Human-tc/JPN/AU-1/1982/G3P3[9]	87,6

	**RVA/Human-wt/RWA/UFS-NGS:MRC-DPRU9995/2015/G12P[8]**	
1	**RVA/Human-wt/RWA/UFS-NGS:MRC-DPRU8020/2015/G12P[8]**	**100,0**
2	KM230944-RVA/Human-xx/ITA/RC37/2009/G10P[8]	99,7
3	KJ752855-RVA/Human-wt/ZWE/MRC-DPRU1850/2011/G4P[8]	99,7
4	JN258918-RVA/Human-wt/Bel/BE00097/2009/G1P[8]	99,6
5	KJ559386-RVA/Human-wt/PRY/3/1998/G4P[8]	99,6
6	JN258334-RVA/Human-wt/USA/2007719720/2007/G1P[8]	99,5
7	HM773772-RVA/Human-wt/USA2007719698/2007/G1P[8]	99,2
8	JF490870-RVA/Human/USA/VU06-07-31/2006/G1P[8]	99,2
9	JN258381-RVA/Human-wt/USA/2008747288/2008/G1P[8]	99,2
10	JX185766-RVA/Human-wt/ITA/ASTI23/2007/G9P[8]	99,0
11	**RVA/Human-wt/RWA/UFS-NGS:MRC-DPRU6235/2014/G4P[4]**	**99,0**
12	JX943606-RVA/Human-TC/USA/Rotarix/2009/G1P[8]	98,9
13	LC173793-RVA/Human-xx/JPN/NT064/2013/G1P[8]	98,9
14	JX416224-RVA/Human-tc/AUS/McN13/1980/G3P2A[6]	98,8
15	KT695112-RVA/Human-wt/USA/DC5685/1991/G1P[8]	98,6
16	JX629048-RVA/Human-tc/JPN/YO/1977/G3P1A[8]	98,6
17	KX632317-RVA/Human-wt/UGA/MUL-12-093/2012/G9P[8]	98,5
18	JN013980-RVA/Human-wt/ZAF/2371WC/2008/G9P[8]	98,5
19	KJ870919-RVA/Human-wt/COD/KisB521/2008/G12P[6]	98,5
20	MH171443-RVA/Human-wt/ESP/SS454877/2011/G1P[8]	98,5
21	JX195082-RVA/Human-wt/ITA/AV28/2010/G9P[8]	98,5
22	JX406753-RVA/Human-tc/USA/Wa/1974/G1P[8]	98,5
23	LC439278-RVA/Human-wt/GHA/M0094/2010/G9P[8]	98,5
24	LC439277-RVA/Human-wt/GHA/DC949/2010/G9P[8]	98,5
25	KU048716-RVA/Human-wt/ITA/PA414/12/2012/G12P[8]	98,5
26	LC173794-RVA/Human-xx/JPN/KN067/2013/G1P[8]	98,4
27	KF371989-RVA/Human-wt/CHN/Y106/2004/G3P[8]	98,3
28	JQ988896-RVA/Human-wt/HRV/CR2006/2006/G8P[8]	98,3
29	LC374134-RVA/Human-wt/NPL/09N3140/2009/G12P[6]	98,0
30	KU048707-RVA/Human-wt/ITA/PA417/14/2014/G12P[8]	97,9
31	EF990711-RVA/Human-wt/BEL/B3458/2003/G9P[8]	97,9
32	JX195093-RVA/Human-wt/ITA/JES11/2010/G9P[8]	97,9
33	MG181532-RVA/Human-wt/MWI/BID14A/2012/G1P[8]	97,9
34	AB861964-RVA/Human-tc/KEN/KDH651/2010/G12P[8]	97,8
35	LC019060-RVA/Human-tc/MMR/A23/2011/G12P[6]	97,8
36	MG926711-RVA/Human-wt/MOZ/0289/2012/G12P[6]	97,7
37	KM116054-RVA/Human-wt/USA/2013774166/2013/G12P[8]	97,7
38	KU048715-RVA/Human-wt/ITA/ME650/14/2014/G12P[8]	97,7
39	KU048714-RVA/Human-wt/ITA/PA525/14/2014/G12P[8]	97,7
40	KU048703-RVA/Human-wt/ITA/PA93/12/2012/G12P[8]	97,7
41	KU048708-RVA/Human-wt/ITA/ME659/14/2014/G12P[8]	97,7
42	KX363069-RVA/Human-wt/VNM/12053_55/2012/GXP[X]	97,7
43	KX655536-RVA/Cow-wt/UGA/BUW-14-A035/2014/G12P[8]	97,6
44	MH171437-RVA/Human-wt/ESP/SS65130987/2015/G12P[8]	97,6
45	MH171449-RVA/Human-wt/ESP/SS64751571/2016/G12P[8]	97,6
46	**RVA/Human-wt/RWA/UFS-NGS:MRC-DPRU566/2013/G9P[4]**	**96,0**
47	KU317463-RVA/Human-tc/EGY/AS970/2012/G8P[14]	91,7
48	KJ751919-RVA/Cow-wt/ZAF/MRC-DPRU3005/2009/G6P[11]	91,4
49	GU565049-RVA/Vaccine/USA/RotaTeq-WI79-4/1992/G6P1A[8]	91,1
50	GU565060-RVA/Vaccine/USA/RotaTeq-WI79-9/1992/G1P7[5]	91,1
51	GU565071-RVA/Vaccine/USA/RotaTeq-SC2-9/1992/G2P7[5]	91,1
52	GU565093-RVA/Vaccine/USA/RotaTeq-BrB-9/1996/G4P7[5]	91,1
53	GU565082-RVA/Vaccine/USA/RotaTeq-WI78-8/1992/G3P7[5]	91,1
54	KX212881-RVA/Cow-wt/TUR/Amasya-1/2015/G8P[5]	90,7
55	KU128900-RVA/Human-wt/Bel/BEF06018/2014/G29P[41]	90,6
56	KX362752-RVA/Human-wt/VNM/16020_81/2013/GXP[X]	89,2
57	JQ069253-RVA/Human-wt/CAN/RT125-07/2008/G2P[4]	89,1
58	DQ005117-RVA/Human-wt/COD/DRC86/2003/G8P[6]	89,0
59	KP752568-RVA/Human-wt/TZA/MRC-DPRU4570/2011/G8P[4]	89,0
60	MG181422-RVA/Human-wt/MWI/MW2-1057/2004/G1P[8]	89,0
61	EF554090-RVA/Human-wt/BEL/B1711/2002/G6P[6]	89,0
62	DQ005106-RVA/Human-wt/COD/DRC88/2003/G8P[8]	89,0
63	KX655514-RVA/Human-wt/UGA/MSK-13-048/2013/G9P[8]	88,9
64	MG181829-RVA/Human-wt/MWI/BID11E/2012/G2P[4]	88,9
65	KX655459-RVA/Human-wt/UGA/MUL-13-204/2013/G8P[6]	88,9
66	JF460842-RVA/Human/BEL/F01498/2009/G3P[6]	88,9
67	HQ657135-RVA/Human-wt/MWI/1473/2001/G8P[4]	88,8
68	KC834692-RVA/Human-wt/AUS/V233/1999/G2P[4]	88,8
69	LC406827-RVA/Human-wt/KEN/KDH1255/2012/G8P[4]	88,8
70	JN013979-RVA/Human-wt/ZAF/2371WC/2008/G9P[8]	88,8
71	KF636242-RVA/Human-wt/KEN/MRC-DPRU1606/2009/G8P[4]	88,8
72	DQ490542-RVA/Human-wt/BGD/RV161/2000/G12P[6]	88,7
73	KU248380-RVA/Human-wt/BGN/J303/2010/G2P[4]	88,7
74	DQ146679-RVA/Human-xx/BGD/Matlab13/2003/G12P[6]	88,7
75	KC571499-RVA/Human-wt/AUS/SA066/2010/G2P[4]	88,7
76	MG181928-RVA/Human-wt/MWI/BID1CT/2012/G2P[4]	88,7
77	KX655470-RVA/Human-wt/UGA/MUL-13-496/2013/G8P[4]	88,5
78	KC834691-RVA/Human-wt/AUS/336190/2004/G2P[4]	88,5
79	HQ650122-RVA/Human-tc/USA/DS-1/1976/G2P[4]	88,5
80	**RVA/Human-wt/RWA/UFS-NGS:MRC-DPRU6212/2014/G12P[8]**	**88,5**
81	DQ490535-RVA/Human-tc/JPN/AU-1/1982/G3P3[9]	87,6

	**RVA/Human-wt/RWA/UFS-NGS:MRC-DPRU6212/2014/G12P[8]**	
1	KX655470-RVA/Human-wt/UGA/MUL-13-496/2013/G8P[4]	99,7
2	KF636242-RVA/Human-wt/KEN/MRC-DPRU1606/2009/G8P[4]	99,6
3	KP752568-RVA/Human-wt/TZA/MRC-DPRU4570/2011/G8P[4]	99,4
4	LC406827-RVA/Human-wt/KEN/KDH1255/2012/G8P[4]	99,3
5	DQ005117-RVA/Human-wt/COD/DRC86/2003/G8P[6]	99,1
6	KX655459-RVA/Human-wt/UGA/MUL-13-204/2013/G8P[6]	99,1
7	KC834692-RVA/Human-wt/AUS/V233/1999/G2P[4]	99,1
8	KC571499-RVA/Human-wt/AUS/SA066/2010/G2P[4]	99,0
9	DQ490542-RVA/Human-wt/BGD/RV161/2000/G12P[6]	99,0
10	DQ005106-RVA/Human-wt/COD/DRC88/2003/G8P[8]	99,0
11	HQ657135-RVA/Human-wt/MWI/1473/2001/G8P[4]	99,0
12	KX362752-RVA/Human-wt/VNM/16020_81/2013/GXP[X]	99,0
13	KX655514-RVA/Human-wt/UGA/MSK-13-048/2013/G9P[8]	99,0
14	MG181422-RVA/Human-wt/MWI/MW2-1057/2004/G1P[8]	99,0
15	JQ069253-RVA/Human-wt/CAN/RT125-07/2008/G2P[4]	98,9
16	MG181829-RVA/Human-wt/MWI/BID11E/2012/G2P[4]	98,9
17	KU248380-RVA/Human-wt/BGN/J303/2010/G2P[4]	98,9
18	JN013979-RVA/Human-wt/ZAF/2371WC/2008/G9P[8]	98,9
19	JF460842-RVA/Human/BEL/F01498/2009/G3P[6]	98,9
20	KC834691-RVA/Human-wt/AUS/336190/2004/G2P[4]	98,9
21	EF554090-RVA/Human-wt/BEL/B1711/2002/G6P[6]	98,9
22	DQ146679-RVA/Human-xx/BGD/Matlab13/2003/G12P[6]	98,8
23	MG181928-RVA/Human-wt/MWI/BID1CT/2012/G2P[4]	98,8
24	HQ650122-RVA/Human-tc/USA/DS-1/1976/G2P[4]	97,5
25	**RVA/Human-wt/RWA/UFS-NGS:MRC-DPRU566/2013/G9P[4]**	91,0
26	DQ490535-RVA/Human-tc/JPN/AU-1/1982/G3P3[9]	90,8
27	**RVA/Human-wt/RWA/UFS-NGS:MRC-DPRU6235/2014/G4P[4]**	89,8
28	KU048703-RVA/Human-wt/ITA/PA93/12/2012/G12P[8]	88,8
29	KX655536-RVA/Cow-wt/UGA/BUW-14-A035/2014/G12P[8]	88,7
30	KT695112-RVA/Human-wt/USA/DC5685/1991/G1P[8]	88,5
31	JX185766-RVA/Human-wt/ITA/ASTI23/2007/G9P[8]	88,5
32	HM773772-RVA/Human-wt/USA2007719698/2007/G1P[8]	88,5
33	JF490870-RVA/Human/USA/VU06-07-31/2006/G1P[8]	88,5
34	JN258381-RVA/Human-wt/USA/2008747288/2008/G1P[8]	88,5
35	**RVA/Human-wt/RWA/UFS-NGS:MRC-DPRU9995/2015/G12P[8]**	88,5
36	**RVA/Human-wt/RWA/UFS-NGS:MRC-DPRU8020/2015/G12P[8]**	88,5
37	KM116054-RVA/Human-wt/USA/2013774166/2013/G12P[8]	88,4
38	JN258918-RVA/Human-wt/Bel/BE00097/2009/G1P[8]	88,4
39	JX416224-RVA/Human-tc/AUS/McN13/1980/G3P2A[6]	88,4
40	KJ870919-RVA/Human-wt/COD/KisB521/2008/G12P[6]	88,4
41	KX363069-RVA/Human-wt/VNM/12053_55/2012/GXP[X]	88,4
42	LC374134-RVA/Human-wt/NPL/09N3140/2009/G12P[6]	88,4
43	MG181532-RVA/Human-wt/MWI/BID14A/2012/G1P[8]	88,4
44	EF990711-RVA/Human-wt/BEL/B3458/2003/G9P[8]	88,4
45	KM230944-RVA/Human-xx/ITA/RC37/2009/G10P[8]	88,4
46	KJ752855-RVA/Human-wt/ZWE/MRC-DPRU1850/2011/G4P[8]	88,4
47	JN013980-RVA/Human-wt/ZAF/2371WC/2008/G9P[8]	88,4
48	MH171443-RVA/Human-wt/ESP/SS454877/2011/G1P[8]	88,4
49	KU048707-RVA/Human-wt/ITA/PA417/14/2014/G12P[8]	88,3
50	KF371989-RVA/Human-wt/CHN/Y106/2004/G3P[8]	88,3
51	JX629048-RVA/Human-tc/JPN/YO/1977/G3P1A[8]	88,3
52	LC019060-RVA/Human-tc/MMR/A23/2011/G12P[6]	88,3
53	KJ751919-RVA/Cow-wt/ZAF/MRC-DPRU3005/2009/G6P[11]	88,3
54	LC439278-RVA/Human-wt/GHA/M0094/2010/G9P[8]	88,3
55	LC439277-RVA/Human-wt/GHA/DC949/2010/G9P[8]	88,3
56	KX632317-RVA/Human-wt/UGA/MUL-12-093/2012/G9P[8]	88,3
57	LC173794-RVA/Human-xx/JPN/KN067/2013/G1P[8]	88,3
58	KU048716-RVA/Human-wt/ITA/PA414/12/2012/G12P[8]	88,2
59	JX195093-RVA/Human-wt/ITA/JES11/2010/G9P[8]	88,2
60	JX943606-RVA/Human-TC/USA/Rotarix/2009/G1P[8]	88,2
61	JQ988896-RVA/Human-wt/HRV/CR2006/2006/G8P[8]	88,2
62	KJ559386-RVA/Human-wt/PRY/3/1998/G4P[8]	88,2
63	AB861964-RVA/Human-tc/KEN/KDH651/2010/G12P[8]	88,2
64	KU048715-RVA/Human-wt/ITA/ME650/14/2014/G12P[8]	88,2
65	KU048714-RVA/Human-wt/ITA/PA525/14/2014/G12P[8]	88,2
66	KU317463-RVA/Human-tc/EGY/AS970/2012/G8P[14]	88,2
67	JX195082-RVA/Human-wt/ITA/AV28/2010/G9P[8]	88,2
68	JN258334-RVA/Human-wt/USA/2007719720/2007/G1P[8]	88,2
69	JX406753-RVA/Human-tc/USA/Wa/1974/G1P[8]	88,1
70	MH171437-RVA/Human-wt/ESP/SS65130987/2015/G12P[8]	88,1
71	MG926711-RVA/Human-wt/MOZ/0289/2012/G12P[6]	88,0
72	MH171449-RVA/Human-wt/ESP/SS64751571/2016/G12P[8]	88,0
73	KU048708-RVA/Human-wt/ITA/ME659/14/2014/G12P[8]	88,0
74	LC173793-RVA/Human-xx/JPN/NT064/2013/G1P[8]	87,9
75	GU565049-RVA/Vaccine/USA/RotaTeq-WI79-4/1992/G6P1A[8]	87,4
76	GU565060-RVA/Vaccine/USA/RotaTeq-WI79-9/1992/G1P7[5]	87,4
77	GU565071-RVA/Vaccine/USA/RotaTeq-SC2-9/1992/G2P7[5]	87,4
78	GU565093-RVA/Vaccine/USA/RotaTeq-BrB-9/1996/G4P7[5]	87,4
79	GU565082-RVA/Vaccine/USA/RotaTeq-WI78-8/1992/G3P7[5]	87,4
80	KU128900-RVA/Human-wt/Bel/BEF06018/2014/G29P4[1]	87,3
81	KX212881-RVA/Cow-wt/TUR/Amasya-1/2015/G8P[5]	87,3

H. NSP4

	**RVA/Human-wt/RWA/UFS-NGS:MRC-DPRU6235/2014/G4P[4]**	
1	KJ162417-RVA/Human-wt/RUS/NN1354-12/2012/G4P[8]	99,6
2	KP013461-RVA/Human-wt/DEN/S14953/2011/G9P[8]	99,6
3	LC173978-RVA/Human-wt/JPN/NT061/2013/G1P[8]	99,4
4	KU361019-RVA/Human-wt/BRA/QUI-108-F1/2009/G1P[8]	99,4
5	JQ988897-RVA/Human-wt/Croatia/CR2006/2006/G8P[8]	99,4
6	JX185767-RVA/Human-wt/ITA/ASTI23/2007/G9P[8]	99,2
7	MH171459-RVA/Human-wt/ESP/SS454877/2011/G1P[8]	99,2
8	JX195072-RVA/Human-wt/ITA/AV21/2010/G9P[8]	99,2
9	MH171460-RVA/Human-wt/ESP/SS455348/2011/G1P[8]	99,0
10	KP119548-RVA/Human-wt/ITA/AN13/2013/G4P[8]	99,0
11	JX195094-RVA/Human-wt/ITA/JES11/2010/G9P[8]	98,7
12	KU048746-RVA/Human-wt/ITA/PA417/14/2014/G12P[8]	98,5
13	KT694948-RVA/Human-wt/USA/Wa/1974/G1P[8]	98,0
14	JX943607-RVA/Human-tc/USA/Rotarix/2009/G1P[8]	97,2
15	MG181401-RVA/Human-wt/MWI/OP2-612/2002/G1P[8]	92,4
16	KJ752293-RVA/Human-wt/ZMB/MRC-DPRU3495/2009/G9P[6]	92,1
17	KP013457-RVA/Human-wt/DEN/L21920792/2011/G9P[8]	92,1
18	KJ752605-RVA/Human-wt/TGO/MRC-DPRU2229/xxxx/G9P[8]	92,1
19	MF469196-RVA/Human-wt/USA/SSCRTV_00006/2013/G12P[8]	91,8
20	HQ392315-RVA/Human-wt/BEL/BE00036/2008/G1P[8]	91,6
21	KC769358-RVA/Human-wt/AUS/CK00064/2007/G1P[8]	91,6
22	HQ392188-RVA/Human-wt/BEL/BE00023/2007/G1P[8]	91,1
23	GU199512-RVA/Human-wt/BGD/Matlab36/2002/G11P[8]	91,1
24	HQ392016-RVA/Human-wt/BEL/BE00006/2005/G1P[8]	90,9
25	JF490802-RVA/Human-wt/USA/VU05-06-69/2005/G1P[8]	90,9
26	JF790345-RVA/Human/USA/VU08-09-39/2008/G12P[8]	90,9
27	KX655537-RVA/Cow-wt/UGA/BUW-14-A035/2014/G12P[8]	90,6
28	KU048725-RVA/Human-wt/ITA/PA93/12/2012/G12P[8]	90,6
29	EF560711-RVA/Human/Dhaka6/BGD/2001/G11P[25]	90,6
30	LC105443-RVA/Human-wt/JPN/UR14-16/2014/G9P[8]	90,4
31	MH171450-RVA/Human-wt/ESP/SS257451/2012/G12P[8]	90,4
32	MN067067-RVA/Human-wt/IND/CMC_00001/2010/G1P[8]	90,4
33	HM773764-RVA/Human-wt/USA2007719739/2007/G1P[8]	90,4
34	**RVA/Human-wt/RWA/UFS-NGS:MRC-DPRU566/2013/G9P[4]**	**90,3**
35	LC374135-RVA/Human-wt/NPL/09N3140/2009/G12P[6]	90,2
36	KU361021-RVA/Human-wt/BRA/QUI-151-F1/2010/G1P[8]	90,2
37	LC374146-RVA/Human-wt/NPL/09N3294/2009/G12P[6]	90,2
38	DQ492678-RVA/Human-wt/BGD/Dhaka16/2003/G1P[8]	90,2
39	MN067008-RVA/Human-wt/IND/CMC_00042/2012/G9P[8]	90,1
40	**RVA/Human-wt/RWA/UFS-NGS:MRC-DPRU6212/2014/G12P[8**	**89,9**
41	MN067034-RVA/Human-wt/IND/CMC_00011/2011/G1P[8]	89,9
42	MN066911-RVA/Human-wt/IND/CMC_00010/2011/G1P[8]	89,9
43	LC019061-RVA/Human-tc/MMR/A23/2011/G12P[6]	89,6
44	HQ650125-RVA/Human-tc/USA/DS-1/1976/G2P[4]	70,5
45	D89873-RVA/Human-tc/JPN/AU-1/1982/G3P[9]	69,4
46	KC175256-RVA/Human-wt/IND/N259/2004/G10P[11]	68,8
47	MF184815-RVA/Human-wt/IND/N259/2004/G1G10P[8]	68,8
48	KM660197-RVA/Human-wt/CMR/BA368/2010/G2P[4]	67,5
49	AB853899-RVA/Cow-wt/JPN/Tottori-SG/2013/G15P1[4]	66,8
50	LC340019-RVA/Human-wt/JPN/12597/2014/G8P[14]	66,3
51	KU956015-RVA/Human-wt/HON/2011825363/2011/G10P[14]	66,1
52	**RVA/Human-wt/RWA/UFS-NGS:MRC-DPRU9995/2015/G12P[8**	**65,9**
53	**RVA/Human-wt/RWA/UFS-NGS:MRC-DPRU8020/2015/G12P[8**	**65,9**
54	GU565050-RVA/Vaccine/USA/RotaTeq-WI79-4/1992/G6P1A[8]	65,9
55	LC384339-RVA/Human-wt/JPN/HKD0825/2016/G1P[8]	65,9
56	KC443596-RVA/Human-wt/AUS/CK20039/2008/G1P[8]	65,9
57	GU565050-RVA/Vaccine/USA/RotaTeq-WI79-4/1992/G6P1A[8]	65,9
58	GU565083-RVA/Vaccine/USA/RotaTeq-WI78-8/1992/G3P7[5]	65,9
59	GU565094-RVA/Vaccine/USA/RotaTeq-BrB-9/1996/G4P7[5]	65,9
60	GU565061-RVA/Vaccine/USA/RotaTeq-WI79-9/1992/G1P7[5]	65,9
61	GU565072-RVA/Vaccine/USA/RotaTeq-SC2-9/1992/G2P7[5]	65,9
62	MF168103-RVA/Human-wt/USA/VU12-13-42/2013/G12P[8]	65,4
63	FJ422140-RVA/Rhesus-tc/USA/PTRV/1990/G8P[1]	65,3
64	LC074704-RVA/Cat-tc/JPN/FRV537/2004/G6P[5]	65,1
65	AB748604-RVA/Cow-tc/JPN/BRV106/1983/G6P[1]	65,0
66	MF940684-RVA/Bovine-tc/KOR/KJ19-2/2006/G6P[7]	64,8
67	AB748605-RVA/Cow-tc/IND/CR231/39/1994-1997/G6P[1]	64,3
68	AB748606-RVA/Human-tc/ISR/Ro8059/1995/G6P[1]	63,7
69	KT281129-RVA/Human-wt/USA/2012741499/2012/G24P[14]	63,7
70	AB908945-RVA/Horse-tc/JPN/JE29/1997/G3P[12]	62,6
71	AB908921-RVA/Horse-tc/JPN/No.32/2010/G3P[12]	61,8

	**RVA/Human-wt/RWA/UFS-NGS:MRC-DPRU566/2013/G9P[4]**	
1	MN067008-RVA/Human-wt/IND/CMC_00042/2012/G9P[8]	99,0
2	HQ392315-RVA/Human-wt/BEL/BE00036/2008/G1P[8]	98,7
3	HQ392188-RVA/Human-wt/BEL/BE00023/2007/G1P[8]	98,6
4	KX655537-RVA/Cow-wt/UGA/BUW-14-A035/2014/G12P[8]	98,6
5	KU048725-RVA/Human-wt/ITA/PA93/12/2012/G12P[8]	98,6
6	MH171450-RVA/Human-wt/ESP/SS257451/2012/G12P[8]	98,4
7	KC769358-RVA/Human-wt/AUS/CK00064/2007/G1P[8]	98,3
8	KJ752293-RVA/Human-wt/ZMB/MRC-DPRU3495/2009/G9P[6]	97,9
9	KP013457-RVA/Human-wt/DEN/L21920792/2011/G9P[8]	97,9
10	KJ752605-RVA/Human-wt/TGO/MRC-DPRU2229/xxxx/G9P[8]	97,9
11	GU199512-RVA/Human-wt/BGD/Matlab36/2002/G11P[8]	97,9
12	MF469196-RVA/Human-wt/USA/SSCRTV_00006/2013/G12P[8]	97,7
13	JF790345-RVA/Human/USA/VU08-09-39/2008/G12P[8]	97,6
14	EF560711-RVA/Human/Dhaka6/BGD/2001/G11P[25]	97,4
15	HQ392016-RVA/Human-wt/BEL/BE00006/2005/G1P[8]	97,2
16	JF490802-RVA/Human-wt/USA/VU05-06-69/2005/G1P[8]	97,2
17	MG181401-RVA/Human-wt/MWI/OP2-612/2002/G1P[8]	97,2
18	MN067067-RVA/Human-wt/IND/CMC_00001/2010/G1P[8]	97,2
19	HM773764-RVA/Human-wt/USA2007719739/2007/G1P[8]	97,2
20	LC374135-RVA/Human-wt/NPL/09N3140/2009/G12P[6]	97,0
21	KU361021-RVA/Human-wt/BRA/QUI-151-F1/2010/G1P[8]	97,0
22	LC374146-RVA/Human-wt/NPL/09N3294/2009/G12P[6]	97,0
23	DQ492678-RVA/Human-wt/BGD/Dhaka16/2003/G1P[8]	97,0
24	LC105443-RVA/Human-wt/JPN/UR14-16/2014/G9P[8]	96,8
25	MN067034-RVA/Human-wt/IND/CMC_00011/2011/G1P[8]	96,8
26	MN066911-RVA/Human-wt/IND/CMC_00010/2011/G1P[8]	96,8
27	LC019061-RVA/Human-tc/MMR/A23/2011/G12P[6]	96,6
28	**RVA/Human-wt/RWA/UFS-NGS:MRC-DPRU6212/2014/G12P[8]**	**96,4**
29	KT694948-RVA/Human-wt/USA/Wa/1974/G1P[8]	91,7
30	KU361019-RVA/Human-wt/BRA/QUI-108-F1/2009/G1P[8]	91,0
31	JQ988897-RVA/Human-wt/Croatia/CR2006/2006/G8P[8]	91,0
32	KJ162417-RVA/Human-wt/RUS/NN1354-12/2012/G4P[8]	90,8
33	KP013461-RVA/Human-wt/DEN/S14953/2011/G9P[8]	90,8
34	JX185767-RVA/Human-wt/ITA/ASTI23/2007/G9P[8]	90,8
35	MH171459-RVA/Human-wt/ESP/SS454877/2011/G1P[8]	90,8
36	JX195072-RVA/Human-wt/ITA/AV21/2010/G9P[8]	90,8
37	KU048746-RVA/Human-wt/ITA/PA417/14/2014/G12P[8]	90,8
38	JX943607-RVA/Human-tc/USA/Rotarix/2009/G1P[8]	90,8
39	LC173978-RVA/Human-wt/JPN/NT061/2013/G1P[8]	90,6
40	MH171460-RVA/Human-wt/ESP/SS455348/2011/G1P[8]	90,6
41	KP119548-RVA/Human-wt/ITA/AN13/2013/G4P[8]	90,6
42	JX195094-RVA/Human-wt/ITA/JES11/2010/G9P[8]	90,5
43	**RVA/Human-wt/RWA/UFS-NGS:MRC-DPRU6235/2014/G4P[4]**	**90,3**
44	KU956015-RVA/Human-wt/HON/2011825363/2011/G10P[14]	71,8
45	KC175256-RVA/Human-wt/IND/N259/2004/G10P[11]	69,4
46	MF184815-RVA/Human-wt/IND/N259/2004/G1G10P[8]	69,4
47	HQ650125-RVA/Human-tc/USA/DS-1/1976/G2P[4]	69,4
48	FJ422140-RVA/Rhesus-tc/USA/PTRV/1990/G8P[1]	68,7
49	LC074704-RVA/Cat-tc/JPN/FRV537/2004/G6P[5]	68,5
50	D89873-RVA/Human-tc/JPN/AU-1/1982/G3P[9]	68,3
51	MF940684-RVA/Bovine-tc/KOR/KJ19-2/2006/G6P[7]	68,0
52	AB853899-RVA/Cow-wt/JPN/Tottori-SG/2013/G15P[14]	67,6
53	LC340019-RVA/Human-wt/JPN/12597/2014/G8P[14]	67,4
54	AB748604-RVA/Cow-tc/JPN/BRV106/1983/G6P[1]	67,0
55	AB748605-RVA/Cow-tc/IND/CR231/39/1994-1997/G6P[1]	66,3
56	AB908945-RVA/Horse-tc/JPN/JE29/1997/G3P[12]	66,3
57	AB908921-RVA/Horse-tc/JPN/No.32/2010/G3P[12]	66,3
58	**RVA/Human-wt/RWA/UFS-NGS:MRC-DPRU9995/2015/G12P[8]**	**65,9**
59	**RVA/Human-wt/RWA/UFS-NGS:MRC-DPRU8020/2015/G12P[8]**	**65,9**
60	GU565050-RVA/Vaccine/USA/RotaTeq-WI79-4/1992/G6P1A[8]	65,9
61	LC384339-RVA/Human-wt/JPN/HKD0825/2016/G1P[8]	65,9
62	KC443596-RVA/Human-wt/AUS/CK20039/2008/G1P[8]	65,9
63	GU565050-RVA/Vaccine/USA/RotaTeq-WI79-4/1992/G6P1A[8]	65,9
64	GU565083-RVA/Vaccine/USA/RotaTeq-WI78-8/1992/G3P7[5]	65,9
65	GU565094-RVA/Vaccine/USA/RotaTeq-BrB-9/1996/G4P7[5]	65,9
66	GU565061-RVA/Vaccine/USA/RotaTeq-WI79-9/1992/G1P7[5]	65,9
67	GU565072-RVA/Vaccine/USA/RotaTeq-SC2-9/1992/G2P7[5]	65,9
68	AB748606-RVA/Human-tc/ISR/Ro8059/1995/G6P[1]	65,8
69	KT281129-RVA/Human-wt/USA/2012741499/2012/G24P[14]	65,8
70	MF168103-RVA/Human-wt/USA/VU12-13-42/2013/G12P[8]	65,4
71	KM660197-RVA/Human-wt/CMR/BA368/2010/G2P[4]	62,3

	**RVA/Human-wt/RWA/UFS-NGS:MRC-DPRU8020/2015/G12P[8]**	
1	**RVA/Human-wt/RWA/UFS-NGS:MRC-DPRU9995/2015/G12P[8]**	**100,0**
2	GU565050-RVA/Vaccine/USA/RotaTeq-WI79-4/1992/G6P1A[8]	100,0
3	LC384339-RVA/Human-wt/JPN/HKD0825/2016/G1P[8]	100,0
4	KC443596-RVA/Human-wt/AUS/CK20039/2008/G1P[8]	100,0
5	GU565050-RVA/Vaccine/USA/RotaTeq-WI79-4/1992/G6P1A[8]	100,0
6	GU565083-RVA/Vaccine/USA/RotaTeq-WI78-8/1992/G3P7[5]	100,0
7	GU565094-RVA/Vaccine/USA/RotaTeq-BrB-9/1996/G4P7[5]	100,0
8	GU565061-RVA/Vaccine/USA/RotaTeq-WI79-9/1992/G1P7[5]	100,0
9	GU565072-RVA/Vaccine/USA/RotaTeq-SC2-9/1992/G2P7[5]	100,0
10	MF168103-RVA/Human-wt/USA/VU12-13-42/2013/G12P[8]	99,8
11	AB748606-RVA/Human-tc/ISR/Ro8059/1995/G6P[1]	97,7
12	AB748604-RVA/Cow-tc/JPN/BRV106/1983/G6P[1]	97,5
13	AB748605-RVA/Cow-tc/IND/CR231/39/1994-1997/G6P[1]	97,4
14	FJ422140-RVA/Rhesus-tc/USA/PTRV/1990/G8P[1]	97,2
15	LC074704-RVA/Cat-tc/JPN/FRV537/2004/G6P[5]	96,8
16	LC340019-RVA/Human-wt/JPN/12597/2014/G8P[14]	96,6
17	KT281129-RVA/Human-wt/USA/2012741499/2012/G24P[14]	95,8
18	AB853899-RVA/Cow-wt/JPN/Tottori-SG/2013/G15P[14]	94,9
19	MF940684-RVA/Bovine-tc/KOR/KJ19-2/2006/G6P[7]	92,1
20	AB908945-RVA/Horse-tc/JPN/JE29/1997/G3P[12]	91,9
21	AB908921-RVA/Horse-tc/JPN/No.32/2010/G3P[12]	91,4
22	KC175256-RVA/Human-wt/IND/N259/2004/G10P[11]	90,6
23	MF184815-RVA/Human-wt/IND/N259/2004/G1G10P[8]	90,6
24	KU956015-RVA/Human-wt/HON/2011825363/2011/G10P[14]	88,1
25	HQ650125-RVA/Human-tc/USA/DS-1/1976/G2P[4]	83,3
26	KM660197-RVA/Human-wt/CMR/BA368/2010/G2P[4]	80,8
27	MG181401-RVA/Human-wt/MWI/OP2-612/2002/G1P[8]	69,1
28	GU199512-RVA/Human-wt/BGD/Matlab36/2002/G11P[8]	68,5
29	HQ392188-RVA/Human-wt/BEL/BE00023/2007/G1P[8]	68,1
30	KC769358-RVA/Human-wt/AUS/CK00064/2007/G1P[8]	68,1
31	KJ752293-RVA/Human-wt/ZMB/MRC-DPRU3495/2009/G9P[6]	68,0
32	KP013457-RVA/Human-wt/DEN/L21920792/2011/G9P[8]	68,0
33	KJ752605-RVA/Human-wt/TGO/MRC-DPRU2229/xxxx/G9P[8]	68,0
34	JF790345-RVA/Human/USA/VU08-09-39/2008/G12P[8]	68,0
35	KT694948-RVA/Human-wt/USA/Wa/1974/G1P[8]	67,8
36	EF560711-RVA/Human/Dhaka6/BGD/2001/G11P[25]	67,7
37	LC105443-RVA/Human-wt/JPN/UR14-16/2014/G9P[8]	67,7
38	HQ392315-RVA/Human-wt/BEL/BE00036/2008/G1P[8]	67,6
39	JF490802-RVA/Human-wt/USA/VU05-06-69/2005/G1P[8]	67,6
40	**RVA/Human-wt/RWA/UFS-NGS:MRC-DPRU6212/2014/G12P[8]**	**67,4**
41	MF469196-RVA/Human-wt/USA/SSCRTV_00006/2013/G12P[8]	67,4
42	LC374135-RVA/Human-wt/NPL/09N3140/2009/G12P[6]	67,4
43	KU361021-RVA/Human-wt/BRA/QUI-151-F1/2010/G1P[8]	67,4
44	LC374146-RVA/Human-wt/NPL/09N3294/2009/G12P[6]	67,4
45	KX655537-RVA/Cow-wt/UGA/BUW-14-A035/2014/G12P[8]	67,2
46	KU048725-RVA/Human-wt/ITA/PA93/12/2012/G12P[8]	67,2
47	HQ392016-RVA/Human-wt/BEL/BE00006/2005/G1P[8]	67,2
48	MH171459-RVA/Human-wt/ESP/SS454877/2011/G1P8]	67,1
49	JX195072-RVA/Human-wt/ITA/AV21/2010/G9P[8]	67,1
50	MH171460-RVA/Human-wt/ESP/SS455348/2011/G1P[8]	67,1
51	MN067067-RVA/Human-wt/IND/CMC_00001/2010/G1P[8]	67,0
52	HM773764-RVA/Human-wt/USA2007719739/2007/G1P[8]	67,0
53	MN067034-RVA/Human-wt/IND/CMC_00011/2011/G1P[8]	66,8
54	MN066911-RVA/Human-wt/IND/CMC_00010/2011/G1P[8]	66,8
55	LC019061-RVA/Human-tc/MMR/A23/2011/G12P[6]	66,8
56	KU048746-RVA/Human-wt/ITA/PA417/14/2014/G12P[8]	66,8
57	MN067008-RVA/Human-wt/IND/CMC_00042/2012/G9P[8]	66,8
58	JX943607-RVA/Human-tc/USA/Rotarix/2009/G1P[8]	66,7
59	KU361019-RVA/Human-wt/BRA/QUI-108-F1/2009/G1P[8]	66,7
60	JQ988897-RVA/Human-wt/Croatia/CR2006/2006/G8P[8]	66,7
61	KP119548-RVA/Human-wt/ITA/AN13/2013/G4P[8]	66,7
62	MH171450-RVA/Human-wt/ESP/SS257451/2012/G12P[8]	66,6
63	DQ492678-RVA/Human-wt/BGD/Dhaka16/2003/G1P[8]	66,6
64	KJ162417-RVA/Human-wt/RUS/NN1354-12/2012/G4P[8]	66,3
65	KP013461-RVA/Human-wt/DEN/S14953/2011/G9P[8]	66,3
66	JX185767-RVA/Human-wt/ITA/ASTI23/2007/G9P[8]	66,3
67	**RVA/Human-wt/RWA/UFS-NGS:MRC-DPRU566/2013/G9P[4]**	**65,9**
68	**RVA/Human-wt/RWA/UFS-NGS:MRC-DPRU6235/2014/G4P[4]**	**65,9**
69	LC173978-RVA/Human-wt/JPN/NT061/2013/G1P[8]	65,9
70	JX195094-RVA/Human-wt/ITA/JES11/2010/G9P[8]	65,3
71	D89873-RVA/Human-tc/JPN/AU-1/1982/G3P[9]	60,8

	**RVA/Human-wt/RWA/UFS-NGS:MRC-DPRU9995/2015/G12P[8]**	
1	**RVA/Human-wt/RWA/UFS-NGS:MRC-DPRU8020/2015/G12P[8]**	**100,0**
2	GU565050-RVA/Vaccine/USA/RotaTeq-WI79-4/1992/G6P1A[8]	100,0
3	LC384339-RVA/Human-wt/JPN/HKD0825/2016/G1P[8]	100,0
4	KC443596-RVA/Human-wt/AUS/CK20039/2008/G1P[8]	100,0
5	GU565050-RVA/Vaccine/USA/RotaTeq-WI79-4/1992/G6P1A[8]	100,0
6	GU565083-RVA/Vaccine/USA/RotaTeq-WI78-8/1992/G3P7[5]	100,0
7	GU565094-RVA/Vaccine/USA/RotaTeq-BrB-9/1996/G4P7[5]	100,0
8	GU565061-RVA/Vaccine/USA/RotaTeq-WI79-9/1992/G1P7[5]	100,0
9	GU565072-RVA/Vaccine/USA/RotaTeq-SC2-9/1992/G2P7[5]	100,0
10	MF168103-RVA/Human-wt/USA/VU12-13-42/2013/G12P[8]	99,8
11	AB748606-RVA/Human-tc/ISR/Ro8059/1995/G6P[1]	97,7
12	AB748604-RVA/Cow-tc/JPN/BRV106/1983/G6P[1]	97,5
13	AB748605-RVA/Cow-tc/IND/CR231/39/1994-1997/G6P[1]	97,4
14	FJ422140-RVA/Rhesus-tc/USA/PTRV/1990/G8P[1]	97,2
15	LC074704-RVA/Cat-tc/JPN/FRV537/2004/G6P[5]	96,8
16	LC340019-RVA/Human-wt/JPN/12597/2014/G8P[14]	96,6
17	KT281129-RVA/Human-wt/USA/2012741499/2012/G24P[14]	95,8
18	AB853899-RVA/Cow-wt/JPN/Tottori-SG/2013/G15P[14]	94,9
19	MF940684-RVA/Bovine-tc/KOR/KJ19-2/2006/G6P[7]	92,1
20	AB908945-RVA/Horse-tc/JPN/JE29/1997/G3P[12]	91,9
21	AB908921-RVA/Horse-tc/JPN/No.32/2010/G3P[12]	91,4
22	KC175256-RVA/Human-wt/IND/N259/2004/G10P[11]	90,6
23	MF184815-RVA/Human-wt/IND/N259/2004/G1G10P[8]	90,6
24	KU956015-RVA/Human-wt/HON/2011825363/2011/G10P[14]	88,1
25	HQ650125-RVA/Human-tc/USA/DS-1/1976/G2P[4]	83,3
26	KM660197-RVA/Human-wt/CMR/BA368/2010/G2P[4]	80,8
27	MG181401-RVA/Human-wt/MWI/OP2-612/2002/G1P[8]	69,1
28	GU199512-RVA/Human-wt/BGD/Matlab36/2002/G11P[8]	68,5
29	HQ392188-RVA/Human-wt/BEL/BE00023/2007/G1P[8]	68,1
30	KC769358-RVA/Human-wt/AUS/CK00064/2007/G1P[8]	68,1
31	KJ752293-RVA/Human-wt/ZMB/MRC-DPRU3495/2009/G9P[6]	68,0
32	KP013457-RVA/Human-wt/DEN/L21920792/2011/G9P[8]	68,0
33	KJ752605-RVA/Human-wt/TGO/MRC-DPRU2229/xxxx/G9P[8]	68,0
34	JF790345-RVA/Human/USA/VU08-09-39/2008/G12P[8]	68,0
35	KT694948-RVA/Human-wt/USA/Wa/1974/G1P[8]	67,8
36	EF560711-RVA/Human/Dhaka6/BGD/2001/G11P[25]	67,7
37	LC105443-RVA/Human-wt/JPN/UR14-16/2014/G9P[8]	67,7
38	HQ392315-RVA/Human-wt/BEL/BE00036/2008/G1P[8]	67,6
39	JF490802-RVA/Human-wt/USA/VU05-06-69/2005/G1P[8]	67,6
40	**RVA/Human-wt/RWA/UFS-NGS:MRC-DPRU6212/2014/G12P[8]**	**67,4**
41	MF469196-RVA/Human-wt/USA/SSCRTV_00006/2013/G12P[8]	67,4
42	LC374135-RVA/Human-wt/NPL/09N3140/2009/G12P[6]	67,4
43	KU361021-RVA/Human-wt/BRA/QUI-151-F1/2010/G1P[8]	67,4
44	LC374146-RVA/Human-wt/NPL/09N3294/2009/G12P[6]	67,4
45	KX655537-RVA/Cow-wt/UGA/BUW-14-A035/2014/G12P[8]	67,2
46	KU048725-RVA/Human-wt/ITA/PA93/12/2012/G12P[8]	67,2
47	HQ392016-RVA/Human-wt/BEL/BE00006/2005/G1P[8]	67,2
48	MH171459-RVA/Human-wt/ESP/SS454877/2011/G1P[8]	67,1
49	JX195072-RVA/Human-wt/ITA/AV21/2010/G9P[8]	67,1
50	MH171460-RVA/Human-wt/ESP/SS455348/2011/G1P[8]	67,1
51	MN067067-RVA/Human-wt/IND/CMC_00001/2010/G1P[8]	67,0
52	HM773764-RVA/Human-wt/USA2007719739/2007/G1P[8]	67,0
53	MN067034-RVA/Human-wt/IND/CMC_00011/2011/G1P[8]	66,8
54	MN066911-RVA/Human-wt/IND/CMC_00010/2011/G1P[8]	66,8
55	LC019061-RVA/Human-tc/MMR/A23/2011/G12P[6]	66,8
56	KU048746-RVA/Human-wt/ITA/PA417/14/2014/G12P[8]	66,8
57	MN067008-RVA/Human-wt/IND/CMC_00042/2012/G9P[8]	66,8
58	JX943607-RVA/Human-tc/USA/Rotarix/2009/G1P[8]	66,7
59	KU361019-RVA/Human-wt/BRA/QUI-108-F1/2009/G1P[8]	66,7
60	JQ988897-RVA/Human-wt/Croatia/CR2006/2006/G8P[8]	66,7
61	KP119548-RVA/Human-wt/ITA/AN13/2013/G4P[8]	66,7
62	MH171450-RVA/Human-wt/ESP/SS257451/2012/G12P[8]	66,6
63	DQ492678-RVA/Human-wt/BGD/Dhaka16/2003/G1P[8]	66,6
64	KJ162417-RVA/Human-wt/RUS/NN1354-12/2012/G4P[8]	66,3
65	KP013461-RVA/Human-wt/DEN/S14953/2011/G9P[8]	66,3
66	JX185767-RVA/Human-wt/ITA/ASTI23/2007/G9P[8]	66,3
67	**RVA/Human-wt/RWA/UFS-NGS:MRC-DPRU566/2013/G9P[4]**	**65,9**
68	**RVA/Human-wt/RWA/UFS-NGS:MRC-DPRU6235/2014/G4P[4]**	**65,9**
69	LC173978-RVA/Human-wt/JPN/NT061/2013/G1P[8]	65,9
70	JX195094-RVA/Human-wt/ITA/JES11/2010/G9P[8]	65,3
71	D89873-RVA/Human-tc/JPN/AU-1/1982/G3P[9]	60,8

	**RVA/Human-wt/RWA/UFS-NGS:MRC-DPRU6212/2014/G12P[8]**	
1	LC374135-RVA/Human-wt/NPL/09N3140/2009/G12P[6]	99,0
2	KU361021-RVA/Human-wt/BRA/QUI-151-F1/2010/G1P[8]	99,0
3	LC374146-RVA/Human-wt/NPL/09N3294/2009/G12P[6]	99,0
4	HQ392016-RVA/Human-wt/BEL/BE00006/2005/G1P[8]	98,8
5	JF490802-RVA/Human-wt/USA/VU05-06-69/2005/G1P[8]	98,8
6	JF790345-RVA/Human/USA/VU08-09-39/2008/G12P[8]	98,8
7	MN067067-RVA/Human-wt/IND/CMC_00001/2010/G1P[8]	98,8
8	HM773764-RVA/Human-wt/USA2007719739/2007/G1P[8]	98,8
9	LC105443-RVA/Human-wt/JPN/UR14-16/2014/G9P[8]	98,8
10	MN067034-RVA/Human-wt/IND/CMC_00011/2011/G1P[8]	98,8
11	MN066911-RVA/Human-wt/IND/CMC_00010/2011/G1P[8]	98,8
12	GU199512-RVA/Human-wt/BGD/Matlab36/2002/G11P[8]	98,7
13	EF560711-RVA/Human/Dhaka6/BGD/2001/G11P[25]	98,7
14	DQ492678-RVA/Human-wt/BGD/Dhaka16/2003/G1P[8]	98,7
15	LC019061-RVA/Human-tc/MMR/A23/2011/G12P[6]	98,7
16	HQ392188-RVA/Human-wt/BEL/BE00023/2007/G1P[8]	97,9
17	KC769358-RVA/Human-wt/AUS/CK00064/2007/G1P[8]	97,9
18	HQ392315-RVA/Human-wt/BEL/BE00036/2008/G1P[8]	97,9
19	MG181401-RVA/Human-wt/MWI/OP2-612/2002/G1P[8]	97,7
20	KX655537-RVA/Cow-wt/UGA/BUW-14-A035/2014/G12P[8]	97,5
21	KJ752293-RVA/Human-wt/ZMB/MRC-DPRU3495/2009/G9P[6]	97,5
22	KP013457-RVA/Human-wt/DEN/L21920792/2011/G9P[8]	97,5
23	KJ752605-RVA/Human-wt/TGO/MRC-DPRU2229/xxxx/G9P[8]	97,5
24	MH171450-RVA/Human-wt/ESP/SS257451/2012/G12P[8]	97,3
25	MF469196-RVA/Human-wt/USA/SSCRTV_00006/2013/G12P[8]	97,3
26	KU048725-RVA/Human-wt/ITA/PA93/12/2012/G12P[8]	97,1
27	MN067008-RVA/Human-wt/IND/CMC_00042/2012/G9P[8]	97,0
28	**RVA/Human-wt/RWA/UFS-NGS:MRC-DPRU566/2013/G9P[4]**	**96,4**
29	KT694948-RVA/Human-wt/USA/Wa/1974/G1P[8]	91,3
30	LC173978-RVA/Human-wt/JPN/NT061/2013/G1P[8]	90,6
31	KU361019-RVA/Human-wt/BRA/QUI-108-F1/2009/G1P[8]	90,6
32	JQ988897-RVA/Human-wt/Croatia/CR2006/2006/G8P[8]	90,6
33	KJ162417-RVA/Human-wt/RUS/NN1354-12/2012/G4P[8]	90,4
34	KP013461-RVA/Human-wt/DEN/S14953/2011/G9P[8]	90,4
35	JX185767-RVA/Human-wt/ITA/ASTI23/2007/G9P[8]	90,4
36	MH171459-RVA/Human-wt/ESP/SS454877/2011/G1P[8]	90,4
37	JX195072-RVA/Human-wt/ITA/AV21/2010/G9P[8]	90,4
38	KU048746-RVA/Human-wt/ITA/PA417/14/2014/G12P[8]	90,4
39	JX943607-RVA/Human-tc/USA/Rotarix/2009/G1P[8]	90,3
40	MH171460-RVA/Human-wt/ESP/SS455348/2011/G1P[8]	90,2
41	KP119548-RVA/Human-wt/ITA/AN13/2013/G4P[8]	90,2
42	JX195094-RVA/Human-wt/ITA/JES11/2010/G9P[8]	90,1
43	**RVA/Human-wt/RWA/UFS-NGS:MRC-DPRU6235/2014/G4P[4]**	**89,9**
44	HQ650125-RVA/Human-tc/USA/DS-1/1976/G2P[4]	70,8
45	KC175256-RVA/Human-wt/IND/N259/2004/G10P[11]	69,0
46	MF184815-RVA/Human-wt/IND/N259/2004/G1G10P[8]	69,0
47	D89873-RVA/Human-tc/JPN/AU-1/1982/G3P[9]	68,2
48	LC340019-RVA/Human-wt/JPN/12597/2014/G8P[14]	68,1
49	FJ422140-RVA/Rhesus-tc/USA/PTRV/1990/G8P[1]	68,0
50	LC074704-RVA/Cat-tc/JPN/FRV537/2004/G6P[5]	67,8
51	KU956015-RVA/Human-wt/HON/2011825363/2011/G10P[14]	67,5
52	GU565050-RVA/Vaccine/USA/RotaTeq-WI79-4/1992/G6P1A[8]	67,4
53	GU565083-RVA/Vaccine/USA/RotaTeq-WI78-8/1992/G3P7[5]	67,4
54	GU565094-RVA/Vaccine/USA/RotaTeq-BrB-9/1996/G4P7[5]	67,4
55	GU565061-RVA/Vaccine/USA/RotaTeq-WI79-9/1992/G1P7[5]	67,4
56	GU565072-RVA/Vaccine/USA/RotaTeq-SC2-9/1992/G2P7[5]	67,4
57	**RVA/Human-wt/RWA/UFS-NGS:MRC-DPRU9995/2015/G12P[8]**	**67,4**
58	**RVA/Human-wt/RWA/UFS-NGS:MRC-DPRU8020/2015/G12P[8]**	**67,4**
59	GU565050-RVA/Vaccine/USA/RotaTeq-WI79-4/1992/G6P1A[8]	67,4
60	LC384339-RVA/Human-wt/JPN/HKD0825/2016/G1P[8]	67,4
61	KC443596-RVA/Human-wt/AUS/CK20039/2008/G1P[8]	67,4
62	MF168103-RVA/Human-wt/USA/VU12-13-42/2013/G12P[8]	67,0
63	AB748604-RVA/Cow-tc/JPN/BRV106/1983/G6P[1]	66,9
64	MF940684-RVA/Bovine-tc/KOR/KJ19-2/2006/G6P[7]	66,4
65	AB853899-RVA/Cow-wt/JPN/Tottori-SG/2013/G15P[14]	66,4
66	AB748605-RVA/Cow-tc/IND/CR231/39/1994-1997/G6P[1]	66,3
67	AB748606-RVA/Human-tc/ISR/Ro8059/1995/G6P[1]	65,7
68	KT281129-RVA/Human-wt/USA/2012741499/2012/G24P[14]	65,1
69	AB908945-RVA/Horse-tc/JPN/JE29/1997/G3P[12]	64,5
70	AB908921-RVA/Horse-tc/JPN/No.32/2010/G3P[12]	63,6
71	KM660197-RVA/Human-wt/CMR/BA368/2010/G2P[4]	61,9

I. NSP5

	**RVA/Human-wt/RWA/UFS-NGS:MRC-DPRU6235/2014/G4P[4]**	
1	KF636356-RVA/Human-wt/ZMB/MRC-DPRU1621/2008/G8P[4]	99,8
2	LC406840-RVA/Human-wt/KEN/KDH1629/2013/G8P[4]	99,7
3	KX655527-RVA/Human-wt/UGA/MUL-13-427/2013/G8P[4]	99,7
4	KF636244-RVA/Human-wt/KEN/MRC-DPRU1606/2009/G8P[4]	99,7
5	KJ752846-RVA/Human-wt/ZAF/MRC-DPRU3477/xxxx/G8P[4]	99,5
6	KJ752661-RVA/Human-wt/UGA/MRC-DPRU1922/2008/G8P[4]	99,5
7	LC406829-RVA/Human-wt/KEN/KDH1255/2012/G8P[4]	99,3
8	KJ751612-RVA/Human-wt/TZA/MRC-DPRU4568/2011/G8P[4]	99,1
9	LC174281-RVA/Human-wt/JPN/SP015/2013/G2P[4]	99,0
10	HQ657137-RVA/Human-wt/MWI/1473/2001/G8P[4]	98,8
11	EF554092-RVA/Human-wt/BEL/B1711/2002/G6P[6]	98,8
12	LC215272-RVA/Human-wt/VNM/SP193/2013/G1P[4]	98,8
13	LC169895-RVA/Human-wt/THA/PCB-103/2013/G8P[8]	98,8
14	DQ490561-RVA/Human-wt/BGD/RV176/2000/G12P[6]	98,8
15	LC174298-RVA/Human-wt/JPN/SP034/2013/G1P[8]	98,6
16	LC174336-RVA/Human-wt/JPN/YM090/2014/G2P[4]	98,6
17	LC477666-RVA/Human-wt/JPN/Tokyo18-25/2018/G3P[8]	98,6
18	HQ650126-RVA/Human-tc/USA/DS-1/1976/G2P[4]	96,5
19	MG926766-RVA/Human-wt/MOZ/0060b/2012/G12P8P[14]	84,1
20	FJ495136-RVA/Antelope-wt/ZAF/RC-18-08/G6P[14]	84,0
21	KC020048-RVA/Human-wt/RUS/O1180/2011/G3P[9]	83,3
22	GU565062-RVA/Vaccine/USA/RotaTeq-WI79-9/1992/G1P7[5]	83,3
23	GU565073-RVA/Vaccine/USA/RotaTeq-SC2-9/1992/G2P7[5]	83,3
24	MF168105-RVA/Human-wt/USA/VU12-13-42/2013/G12P[8]	83,3
25	GU565095-RVA/Vaccine/USA/RotaTeq-BrB-9/1996/G4P7[5]	83,3
26	GU565084-RVA/Vaccine/USA/RotaTeq-WI78-8/1992/G3P7[5]	83,3
27	KF500518-RVA/Human-wt/USA/12US1134/2012/G3P[9]	83,3
28	GU565051-RVA/Vaccine/USA/RotaTeq-WI79-4/1992/G6P1A[8]	83,1
29	**RVA/Human-wt/RWA/UFS-NGS:MRC-DPRU9995/2015/G12P[8]**	**83,1**
30	FJ347121-RVA/Cow-wt/ARG/B383/1998/G15P[11]	82,8
31	**RVA/Human-wt/RWA/UFS-NGS:MRC-DPRU8020/2015/G12P[8]**	**82,8**
32	EF554103-RVA/Human-tc/AUS/MG6/1993/G6P[14]	82,8
33	**RVA/Human-wt/RWA/UFS-NGS:MRC-DPRU6212/2014/G12P[8]**	**81,9**
34	KX212877-RVA/Cow-wt/TUR/Amasya-1/2015/G8P[5]	81,7
35	HQ657148-RVA/Human-wt/ZAF/3133WC/2009/G12P[4]	81,6
36	DQ146670-RVA/Human-wt/BGD/Dhaka12/2003/G12P[6]	81,6
37	LC174316-RVA/Human-wt/JPN/YM051/2013/G9P[8]	81,4
38	KU361041-RVA/Human-wt/BRA/QUI-89-F4/2010/G1P[8]	81,3
39	LC105065-RVA/Human-wt/JPN/HK14-10/2014/G1P[8]	81,3
40	MG926713-RVA/Human-wt/MOZ/0289/2012/G12P[6]	81,3
41	LC174330-RVA/Human-wt/JPN/KN165/2014/G1P[8]	81,3
42	LC374191-RVA/Human-wt/NPL/10N4001/2010/G12P[6]	81,3
43	LC374136-RVA/Human-wt/NPL/09N3140/2009/G12P[6]	81,3
44	KM116058-RVA/Human-wt/USA/2013774166/2013/G12P[8]	81,3
45	KT695147-RVA/Human-tc/USA/DC5685-40-AG/1991/G1P[8]	81,3
46	**RVA/Human-wt/RWA/UFS-NGS:MRC-DPRU566/2013/G9P[4]**	**81,3**
47	KT918731-RVA/Human-wt/USA/VU11-12-192/2012/G12P[8]	81,1
48	KU048760-RVA/Human-wt/ITA/RG176/13/2013/G12P[8]	81,1
49	LC019062-RVA/Human-tc/MMR/A23/2011/G12P[6]	81,1
50	DQ146659-RVA/Human-wt/BGD/Dhaka25/2002/G12P[8]	81,1
51	MG926757-RVA/Human-wt/MOZ/0060a/2012/G12[P8]P[14]	81,1
52	LC477663-RVA/Human-wt/JPN/Tokyo17-21/2017/G3P[8]	81,1
53	LC368117-RVA/Human-wt/NPL/06N0359/2006/G12P[6]	81,0
54	KT920892-RVA/Human-wt/IND/VR11281/2003/G1[P8]	81,0
55	LC372912-RVA/Human-wt/NPL/08N2066/2008/G12P[6]	81,0
56	KT694949-RVA/Human-tc/USA/Wa/1974/G1P[8]	81,0
57	JX943608-RVA/Human-TC/USA/Rotarix/2009/G1P[8]	80,9
58	MK417795-RVA/Human-wt/ESP/SS96099331/2018/G12P[8]	80,8
59	KU048768-RVA/Human-wt/ITA/ME659/14/2014/G12P[8]	80,8
60	MG181479-RVA/Human-wt/MWI/MW2-1274/2005/G1P[8]	80,8
61	LC469596-RVA/Human-wt/IDN/STM457/2018/G1P[8]	80,8
62	LC367288-RVA/Human-wt/NPL/08N2868/2008/G12P[6]	80,5

	**RVA/Human-wt/RWA/UFS-NGS:MRC-DPRU566/2013/G9P[4]**	
1	KM116058-RVA/Human-wt/USA/2013774166/2013/G12P[8]	99,5
2	DQ146659-RVA/Human-wt/BGD/Dhaka25/2002/G12P[8]	99,5
3	KU361041-RVA/Human-wt/BRA/QUI-89-F4/2010/G1P[8]	99,5
4	LC105065-RVA/Human-wt/JPN/HK14-10/2014/G1P[8]	99,5
5	MG926713-RVA/Human-wt/MOZ/0289/2012/G12P[6]	99,3
6	HQ657148-RVA/Human-wt/ZAF/3133WC/2009/G12P[4]	99,3
7	KT918731-RVA/Human-wt/USA/VU11-12-192/2012/G12P[8]	99,3
8	KU048760-RVA/Human-wt/ITA/RG176/13/2013/G12P[8]	99,3
9	MG926757-RVA/Human-wt/MOZ/0060a/2012/G12P8P[14]	99,3
10	LC174330-RVA/Human-wt/JPN/KN165/2014/G1P[8]	99,1
11	MK417795-RVA/Human-wt/ESP/SS96099331/2018/G12P[8]	99,1
12	KT695147-RVA/Human-tc/USA/DC5685-40-AG/1991/G1P[8]	99,1
13	LC174316-RVA/Human-wt/JPN/YM051/2013/G9P[8]	99,1
14	DQ146670-RVA/Human-wt/BGD/Dhaka12/2003/G12P[6]	99,1
15	LC368117-RVA/Human-wt/NPL/06N0359/2006/G12P[6]	98,8
16	MG181479-RVA/Human-wt/MWI/MW2-1274/2005/G1P[8]	98,6
17	LC374191-RVA/Human-wt/NPL/10N4001/2010/G12P[6]	98,6
18	LC374136-RVA/Human-wt/NPL/09N3140/2009/G12P[6]	98,6
19	LC469596-RVA/Human-wt/IDN/STM457/2018/G1P[8]	98,6
20	KT920892-RVA/Human-wt/IND/VR11281/2003/G1P[8]	98,6
21	LC477663-RVA/Human-wt/JPN/Tokyo17-21/2017/G3P[8]	98,5
22	LC019062-RVA/Human-tc/MMR/A23/2011/G12P[6]	98,5
23	LC367288-RVA/Human-wt/NPL/08N2868/2008/G12P[6]	98,5
24	LC372912-RVA/Human-wt/NPL/08N2066/2008/G12P[6]	98,4
25	**RVA/Human-wt/RWA/UFS-NGS:MRC-DPRU6212/2014/G12P[8]**	**98,3**
26	KU048768-RVA/Human-wt/ITA/ME659/14/2014/G12P[8]	98,1
27	KT694949-RVA/Human-tc/USA/Wa/1974/G1P[8]	93,4
28	JX943608-RVA/Human-TC/USA/Rotarix/2009/G1P[8]	92,9
29	KF500518-RVA/Human-wt/USA/12US1134/2012/G3P[9]	86,5
30	EF554103-RVA/Human-tc/AUS/MG6/1993/G6P[14]	86,4
31	GU565062-RVA/Vaccine/USA/RotaTeq-WI79-9/1992/G1P7[5]	86,4
32	GU565073-RVA/Vaccine/USA/RotaTeq-SC2-9/1992/G2P7[5]	86,4
33	MF168105-RVA/Human-wt/USA/VU12-13-42/2013/G12P[8]	86,4
34	GU565095-RVA/Vaccine/USA/RotaTeq-BrB-9/1996/G4P7[5]	86,4
35	GU565084-RVA/Vaccine/USA/RotaTeq-WI78-8/1992/G3P7[5]	86,4
36	MG926766-RVA/Human-wt/MOZ/0060b/2012/G12P8P[14]	86,3
37	GU565051-RVA/Vaccine/USA/RotaTeq-WI79-4/1992/G6P1A[8]	86,1
38	**RVA/Human-wt/RWA/UFS-NGS:MRC-DPRU9995/2015/G12P[8]**	**86,1**
39	**RVA/Human-wt/RWA/UFS-NGS:MRC-DPRU8020/2015/G12P[8]**	**86,1**
40	FJ347121-RVA/Cow-wt/ARG/B383/1998/G15P[11]	85,9
41	FJ495136-RVA/Antelope-wt/ZAF/RC-18-08/G6P[14]	85,8
42	KX212877-RVA/Cow-wt/TUR/Amasya-1/2015/G8P[5]	85,7
43	KC020048-RVA/Human-wt/RUS/O1180/2011/G3P[9]	85,6
44	**RVA/Human-wt/RWA/UFS-NGS:MRC-DPRU6235/2014/G4P[4]**	**81,3**
45	KJ752661-RVA/Human-wt/UGA/MRC-DPRU1922/2008/G8P[4]	81,1
46	KF636356-RVA/Human-wt/ZMB/MRC-DPRU1621/2008/G8P[4]	81,1
47	LC215272-RVA/Human-wt/VNM/SP193/2013/G1P[4]	81,1
48	LC169895-RVA/Human-wt/THA/PCB-103/2013/G8P[8]	81,1
49	HQ657137-RVA/Human-wt/MWI/1473/2001/G8P[4]	80,8
50	EF554092-RVA/Human-wt/BEL/B1711/2002/G6P[6]	80,8
51	LC406840-RVA/Human-wt/KEN/KDH1629/2013/G8P[4]	80,8
52	KX655527-RVA/Human-wt/UGA/MUL-13-427/2013/G8P[4]	80,8
53	KF636244-RVA/Human-wt/KEN/MRC-DPRU1606/2009/G8P[4]	80,8
54	KJ751612-RVA/Human-wt/TZA/MRC-DPRU4568/2011/G8P[4]	80,8
55	LC174298-RVA/Human-wt/JPN/SP034/2013/G1P[8]	80,8
56	LC477666-RVA/Human-wt/JPN/Tokyo18-25/2018/G3P[8]	80,8
57	LC174281-RVA/Human-wt/JPN/SP015/2013/G2P[4]	80,8
58	LC174336-RVA/Human-wt/JPN/YM090/2014/G2P[4]	80,8
59	KJ752846-RVA/Human-wt/ZAF/MRC-DPRU3477/xxxx/G8P[4]	80,6
60	DQ490561-RVA/Human-wt/BGD/RV176/2000/G12P[6]	80,4
61	LC406829-RVA/Human-wt/KEN/KDH1255/2012/G8P[4]	80,2
62	HQ650126-RVA/Human-tc/USA/DS-1/1976/G2P[4]	79,7

	**RVA/Human-wt/RWA/UFS-NGS:MRC-DPRU8020/2015/G12P[8]**	
1	**RVA/Human-wt/RWA/UFS-NGS:MRC-DPRU9995/2015/G12P[8]**	**99,8**
2	GU565062-RVA/Vaccine/USA/RotaTeq-WI79-9/1992/G1P7[5]	99,7
3	GU565073-RVA/Vaccine/USA/RotaTeq-SC2-9/1992/G2P7[5]	99,7
4	MF168105-RVA/Human-wt/USA/VU12-13-42/2013/G12P[8]	99,7
5	GU565095-RVA/Vaccine/USA/RotaTeq-BrB-9/1996/G4P7[5]	99,7
6	GU565084-RVA/Vaccine/USA/RotaTeq-WI78-8/1992/G3P7[5]	99,7
7	GU565051-RVA/Vaccine/USA/RotaTeq-WI79-4/1992/G6P1A[8]	99,5
8	KF500518-RVA/Human-wt/USA/12US1134/2012/G3P[9]	97,0
9	KC020048-RVA/Human-wt/RUS/O1180/2011/G3P[9]	96,9
10	EF554103-RVA/Human-tc/AUS/MG6/1993/G6P[14]	96,5
11	FJ495136-RVA/Antelope-wt/ZAF/RC-18-08/G6P[14]	96,5
12	FJ347121-RVA/Cow-wt/ARG/B383/1998/G15P[11]	96,3
13	KX212877-RVA/Cow-wt/TUR/Amasya-1/2015/G8P[5]	95,7
14	MG926766-RVA/Human-wt/MOZ/0060b/2012/G12P8P[14]	95,7
15	**RVA/Human-wt/RWA/UFS-NGS:MRC-DPRU566/2013/G9P[4]**	**86,1**
16	KT918731-RVA/Human-wt/USA/VU11-12-192/2012/G12P[8]	85,8
17	KU048760-RVA/Human-wt/ITA/RG176/13/2013/G12P[8]	85,8
18	KT695147-RVA/Human-tc/USA/DC5685-40-AG/1991/G1P[8]	85,8
19	DQ146670-RVA/Human-wt/BGD/Dhaka12/2003/G12P[6]	85,8
20	**RVA/Human-wt/RWA/UFS-NGS:MRC-DPRU6212/2014/G12P[8]**	**85,6**
21	LC174316-RVA/Human-wt/JPN/YM051/2013/G9P[8]	85,6
22	DQ146659-RVA/Human-wt/BGD/Dhaka25/2002/G12P[8]	85,6
23	KU361041-RVA/Human-wt/BRA/QUI-89-F4/2010/G1P[8]	85,6
24	LC105065-RVA/Human-wt/JPN/HK14-10/2014/G1P[8]	85,6
25	MG926713-RVA/Human-wt/MOZ/0289/2012/G12P[6]	85,6
26	MK417795-RVA/Human-wt/ESP/SS96099331/2018/G12P[8]	85,6
27	LC374191-RVA/Human-wt/NPL/10N4001/2010/G12P[6]	85,6
28	LC374136-RVA/Human-wt/NPL/09N3140/2009/G12P[6]	85,6
29	LC469596-RVA/Human-wt/IDN/STM457/2018/G1P[8]	85,6
30	LC477663-RVA/Human-wt/JPN/Tokyo17-21/2017/G3P[8]	85,5
31	KU048768-RVA/Human-wt/ITA/ME659/14/2014/G12P[8]	85,4
32	KM116058-RVA/Human-wt/USA/2013774166/2013/G12P[8]	85,3
33	HQ657148-RVA/Human-wt/ZAF/3133WC/2009/G12P[4]	85,3
34	LC019062-RVA/Human-tc/MMR/A23/2011/G12P[6]	85,3
35	LC367288-RVA/Human-wt/NPL/08N2868/2008/G12P[6]	85,3
36	MG926757-RVA/Human-wt/MOZ/0060a/2012/G12P[8]P[14]	85,3
37	LC368117-RVA/Human-wt/NPL/06N0359/2006/G12P[6]	85,3
38	KT920892-RVA/Human-wt/IND/VR11281/2003/G1P[8]	85,3
39	LC174330-RVA/Human-wt/JPN/KN165/2014/G1P[8]	85,1
40	MG181479-RVA/Human-wt/MWI/MW2-1274/2005/G1P[8]	85,1
41	LC372912-RVA/Human-wt/NPL/08N2066/2008/G12P[6]	84,8
42	KT694949-RVA/Human-tc/USA/Wa/1974/G1P[8]	83,9
43	JX943608-RVA/Human-TC/USA/Rotarix/2009/G1P[8]	83,8
44	KJ752661-RVA/Human-wt/UGA/MRC-DPRU1922/2008/G8P[4]	83,0
45	KF636356-RVA/Human-wt/ZMB/MRC-DPRU1621/2008/G8P[4]	83,0
46	LC215272-RVA/Human-wt/VNM/SP193/2013/G1P[4]	83,0
47	LC169895-RVA/Human-wt/THA/PCB-103/2013/G8P[8]	83,0
48	HQ657137-RVA/Human-wt/MWI/1473/2001/G8P[4]	82,8
49	EF554092-RVA/Human-wt/BEL/B1711/2002/G6P6	82,8
50	**RVA/Human-wt/RWA/UFS-NGS:MRC-DPRU6235/2014/G4P4**	**82,8**
51	LC406840-RVA/Human-wt/KEN/KDH1629/2013/G8P[4]	82,8
52	KX655527-RVA/Human-wt/UGA/MUL-13-427/2013/G8P[4]	82,8
53	KF636244-RVA/Human-wt/KEN/MRC-DPRU1606/2009/G8P[4]	82,8
54	LC174298-RVA/Human-wt/JPN/SP034/2013/G1P[8]	82,8
55	LC174281-RVA/Human-wt/JPN/SP015/2013/G2P[4]	82,7
56	LC174336-RVA/Human-wt/JPN/YM090/2014/G2P[4]	82,7
57	KJ752846-RVA/Human-wt/ZAF/MRC-DPRU3477/xxxx/G8P[4]	82,5
58	LC477666-RVA/Human-wt/JPN/Tokyo18-25/2018/G3P[8]	82,5
59	DQ490561-RVA/Human-wt/BGD/RV176/2000/G12P[6]	82,4
60	KJ751612-RVA/Human-wt/TZA/MRC-DPRU4568/2011/G8P[4]	82,2
61	LC406829-RVA/Human-wt/KEN/KDH1255/2012/G8P[4]	82,2
62	HQ650126-RVA/Human-tc/USA/DS-1/1976/G2P[4]	81,7

	**RVA/Human-wt/RWA/UFS-NGS:MRC-DPRU9995/2015/G12P[8]**	
1	GU565062-RVA/Vaccine/USA/RotaTeq-WI79-9/1992/G1P7[5]	99,8
2	GU565073-RVA/Vaccine/USA/RotaTeq-SC2-9/1992/G2P7[5]	99,8
3	MF168105-RVA/Human-wt/USA/VU12-13-42/2013/G12P[8]	99,8
4	GU565095-RVA/Vaccine/USA/RotaTeq-BrB-9/1996/G4P7[5]	99,8
5	GU565084-RVA/Vaccine/USA/RotaTeq-WI78-8/1992/G3P7[5]	99,8
6	**RVA/Human-wt/RWA/UFS-NGS:MRC-DPRU8020/2015/G12P[8]**	**99,8**
7	GU565051-RVA/Vaccine/USA/RotaTeq-WI79-4/1992/G6P1A[8]	99,7
8	KF500518-RVA/Human-wt/USA/12US1134/2012/G3P[9]	97,2
9	KC020048-RVA/Human-wt/RUS/O1180/2011/G3P[9]	97,0
10	EF554103-RVA/Human-tc/AUS/MG6/1993/G6P[14]	96,7
11	FJ495136-RVA/Antelope-wt/ZAF/RC-18-08/G6P[14]	96,7
12	FJ347121-RVA/Cow-wt/ARG/B383/1998/G15P[11]	96,5
13	KX212877-RVA/Cow-wt/TUR/Amasya-1/2015/G8P[5]	95,9
14	MG926766-RVA/Human-wt/MOZ/0060b/2012/G12P[8]P[14]	95,9
15	**RVA/Human-wt/RWA/UFS-NGS:MRC-DPRU566/2013/G9P[4]**	**86,1**
16	KT918731-RVA/Human-wt/USA/VU11-12-192/2012/G12P[8]	85,8
17	KU048760-RVA/Human-wt/ITA/RG176/13/2013/G12P[8]	85,8
18	KT695147-RVA/Human-tc/USA/DC5685-40-AG/1991/G1P[8]	85,8
19	DQ146670-RVA/Human-wt/BGD/Dhaka12/2003/G12P[6]	85,8
20	**RVA/Human-wt/RWA/UFS-NGS:MRC-DPRU6212/2014/G12P[8]**	**85,6**
21	LC174316-RVA/Human-wt/JPN/YM051/2013/G9P[8]	85,6
22	DQ146659-RVA/Human-wt/BGD/Dhaka25/2002/G12P[8]	85,6
23	KU361041-RVA/Human-wt/BRA/QUI-89-F4/2010/G1P[8]	85,6
24	LC105065-RVA/Human-wt/JPN/HK14-10/2014/G1P[8]	85,6
25	MG926713-RVA/Human-wt/MOZ/0289/2012/G12P[6]	85,6
26	MK417795-RVA/Human-wt/ESP/SS96099331/2018/G12P[8]	85,6
27	LC374191-RVA/Human-wt/NPL/10N4001/2010/G12P[6]	85,6
28	LC374136-RVA/Human-wt/NPL/09N3140/2009/G12P[6]	85,6
29	LC469596-RVA/Human-wt/IDN/STM457/2018/G1P[8]	85,6
30	LC477663-RVA/Human-wt/JPN/Tokyo17-21/2017/G3P[8]	85,6
31	KU048768-RVA/Human-wt/ITA/ME659/14/2014/G12P[8]	85,4
32	KM116058-RVA/Human-wt/USA/2013774166/2013/G12P[8]	85,4
33	HQ657148-RVA/Human-wt/ZAF/3133WC/2009/G12P[4]	85,4
34	LC019062-RVA/Human-tc/MMR/A23/2011/G12P[6]	85,4
35	LC367288-RVA/Human-wt/NPL/08N2868/2008/G12P[6]	85,4
36	MG926757-RVA/Human-wt/MOZ/0060a/2012/G12P8P[14]	85,4
37	LC368117-RVA/Human-wt/NPL/06N0359/2006/G12P[6]	85,3
38	KT920892-RVA/Human-wt/IND/VR11281/2003/G1P[8]	85,3
39	LC174330-RVA/Human-wt/JPN/KN165/2014/G1P[8]	85,2
40	MG181479-RVA/Human-wt/MWI/MW2-1274/2005/G1P[8]	85,1
41	LC372912-RVA/Human-wt/NPL/08N2066/2008/G12P[6]	84,9
42	KT694949-RVA/Human-tc/USA/Wa/1974/G1P[8]	84,0
43	JX943608-RVA/Human-TC/USA/Rotarix/2009/G1P[8]	83,8
44	KJ752661-RVA/Human-wt/UGA/MRC-DPRU1922/2008/G8P[4]	83,3
45	KF636356-RVA/Human-wt/ZMB/MRC-DPRU1621/2008/G8P[4]	83,3
46	LC215272-RVA/Human-wt/VNM/SP193/2013/G1P[4]	83,3
47	LC169895-RVA/Human-wt/THA/PCB-103/2013/G8P[8]	83,3
48	HQ657137-RVA/Human-wt/MWI/1473/2001/G8P[4]	83,1
49	EF554092-RVA/Human-wt/BEL/B1711/2002/G6P[6]	83,1
50	**RVA/Human-wt/RWA/UFS-NGS:MRC-DPRU6235/2014/G4P[4]**	**83,1**
51	LC406840-RVA/Human-wt/KEN/KDH1629/2013/G8P[4]	83,1
52	KX655527-RVA/Human-wt/UGA/MUL-13-427/2013/G8P[4]	83,1
53	KF636244-RVA/Human-wt/KEN/MRC-DPRU1606/2009/G8P[4]	83,1
54	LC174298-RVA/Human-wt/JPN/SP034/2013/G1P[8]	83,1
55	LC174281-RVA/Human-wt/JPN/SP015/2013/G2P[4]	83,0
56	LC174336-RVA/Human-wt/JPN/YM090/2014/G2P[4]	83,0
57	KJ752846-RVA/Human-wt/ZAF/MRC-DPRU3477/xxxx/G8P[4]	82,8
58	LC477666-RVA/Human-wt/JPN/Tokyo18-25/2018/G3P[8]	82,8
59	DQ490561-RVA/Human-wt/BGD/RV176/2000/G12P[6]	82,7
60	KJ751612-RVA/Human-wt/TZA/MRC-DPRU4568/2011/G8P[4]	82,5
61	LC406829-RVA/Human-wt/KEN/KDH1255/2012/G8P[4]	82,5
62	HQ650126-RVA/Human-tc/USA/DS-1/1976/G2P[4]	82,0

	**RVA/Human-wt/RWA/UFS-NGS:MRC-DPRU6212/2014/G12P[8]**	
1	LC374191-RVA/Human-wt/NPL/10N4001/2010/G12P[6]	99,3
2	LC374136-RVA/Human-wt/NPL/09N3140/2009/G12P[6]	99,3
3	LC019062-RVA/Human-tc/MMR/A23/2011/G12P[6]	99,1
4	DQ146670-RVA/Human-wt/BGD/Dhaka12/2003/G12P[6]	99,1
5	MG181479-RVA/Human-wt/MWI/MW2-1274/2005/G1P[8]	99,0
6	LC469596-RVA/Human-wt/IDN/STM457/2018/G1P[8]	99,0
7	LC477663-RVA/Human-wt/JPN/Tokyo17-21/2017/G3P[8]	98,8
8	KU048768-RVA/Human-wt/ITA/ME659/14/2014/G12P[8]	98,8
9	DQ146659-RVA/Human-wt/BGD/Dhaka25/2002/G12P[8]	98,8
10	KT695147-RVA/Human-tc/USA/DC5685-40-AG/1991/G1P[8]	98,8
11	LC368117-RVA/Human-wt/NPL/06N0359/2006/G12P[6]	98,8
12	LC372912-RVA/Human-wt/NPL/08N2066/2008/G12P[6]	98,8
13	KU361041-RVA/Human-wt/BRA/QUI-89-F4/2010/G1P[8]	98,6
14	LC105065-RVA/Human-wt/JPN/HK14-10/2014/G1P[8]	98,6
15	KT920892-RVA/Human-wt/IND/VR11281/2003/G1P[8]	98,6
16	KM116058-RVA/Human-wt/USA/2013774166/2013/G12P[8]	98,5
17	MG926713-RVA/Human-wt/MOZ/0289/2012/G12P[6]	98,5
18	HQ657148-RVA/Human-wt/ZAF/3133WC/2009/G12P[4]	98,5
19	KT918731-RVA/Human-wt/USA/VU11-12-192/2012/G12P[8]	98,5
20	KU048760-RVA/Human-wt/ITA/RG176/13/2013/G12P[8]	98,5
21	LC367288-RVA/Human-wt/NPL/08N2868/2008/G12P[6]	98,5
22	MG926757-RVA/Human-wt/MOZ/0060a/2012/G12P8P[14]	98,5
23	LC174330-RVA/Human-wt/JPN/KN165/2014/G1P[8]	98,3
24	MK417795-RVA/Human-wt/ESP/SS96099331/2018/G12P[8]	98,3
25	LC174316-RVA/Human-wt/JPN/YM051/2013/G9P[8]	98,3
26	**RVA/Human-wt/RWA/UFS-NGS:MRC-DPRU566/2013/G9P[4]**	**98,3**
27	KT694949-RVA/Human-tc/USA/Wa/1974/G1P[8]	92,8
28	JX943608-RVA/Human-TC/USA/Rotarix/2009/G1P[8]	92,7
29	EF554103-RVA/Human-tc/AUS/MG6/1993/G6P[14]	85,9
30	GU565062-RVA/Vaccine/USA/RotaTeq-WI79-9/1992/G1P7[5]	85,9
31	GU565073-RVA/Vaccine/USA/RotaTeq-SC2-9/1992/G2P7[5]	85,9
32	MF168105-RVA/Human-wt/USA/VU12-13-42/2013/G12P[8]	85,9
33	GU565095-RVA/Vaccine/USA/RotaTeq-BrB-9/1996/G4P7[5]	85,9
34	GU565084-RVA/Vaccine/USA/RotaTeq-WI78-8/1992/G3P7[5]	85,9
35	MG926766-RVA/Human-wt/MOZ/0060b/2012/G12P8P[14]	85,8
36	FJ347121-RVA/Cow-wt/ARG/B383/1998/G15P[11]	85,7
37	GU565051-RVA/Vaccine/USA/RotaTeq-WI79-4/1992/G6P1A[8]	85,6
38	**RVA/Human-wt/RWA/UFS-NGS:MRC-DPRU9995/2015/G12P[8**	**85,6**
39	**RVA/Human-wt/RWA/UFS-NGS:MRC-DPRU8020/2014/G12P[8**	**85,6**
40	KF500518-RVA/Human-wt/USA/12US1134/2012/G3P[9]	85,6
41	FJ495136-RVA/Antelope-wt/ZAF/RC-18-08/G6P[14]	85,3
42	KX212877-RVA/Cow-wt/TUR/Amasya-1/2015/G8P[5]	85,2
43	KC020048-RVA/Human-wt/RUS/O1180/2011/G3P[9]	85,1
44	**RVA/Human-wt/RWA/UFS-NGS:MRC-DPRU6235/2015/G4P[4]**	**81,9**
45	KJ752661-RVA/Human-wt/UGA/MRC-DPRU1922/2008/G8P[4]	81,6
46	KF636356-RVA/Human-wt/ZMB/MRC-DPRU1621/2008/G8P[4]	81,6
47	LC215272-RVA/Human-wt/VNM/SP193/2013/G1P[4]	81,6
48	LC169895-RVA/Human-wt/THA/PCB-103/2013/G8P[8]	81,6
49	HQ657137-RVA/Human-wt/MWI/1473/2001/G8P[4]	81,4
50	EF554092-RVA/Human-wt/BEL/B1711/2002/G6P[6]	81,4
51	LC406840-RVA/Human-wt/KEN/KDH1629/2013/G8P[4]	81,4
52	KX655527-RVA/Human-wt/UGA/MUL-13-427/2013/G8P[4]	81,4
53	KF636244-RVA/Human-wt/KEN/MRC-DPRU1606/2009/G8P[4]	81,4
54	KJ751612-RVA/Human-wt/TZA/MRC-DPRU4568/2011/G8P[4]	81,4
55	LC174298-RVA/Human-wt/JPN/SP034/2013/G1P[8]	81,4
56	LC477666-RVA/Human-wt/JPN/Tokyo18-25/2018/G3P[8]	81,4
57	LC174281-RVA/Human-wt/JPN/SP015/2013/G2P[4]	81,3
58	LC174336-RVA/Human-wt/JPN/YM090/2014/G2P[4]	81,3
59	KJ752846-RVA/Human-wt/ZAF/MRC-DPRU3477/xxxx/G8P[4]	81,1
60	DQ490561-RVA/Human-wt/BGD/RV176/2000/G12P[6]	81,0
61	HQ650126-RVA/Human-tc/USA/DS-1/1976/G2P[4]	80,9
62	LC406829-RVA/Human-wt/KEN/KDH1255/2012/G8P[4]	80,8

Supplementary 2 (S2): Nucleotide Homology tables for all the 11 segments based on A) VP6 B) VP1 C) VP2 D) VP3 E) NSP1 F) NSP2 G) NSP3 H) NSP4 I) NSP5 of the study strains from Rwanda.
